# Gas‐Phase Transformation of Fluorinated Benzoporphyrins to Porphyrin‐Embedded Conical Nanocarbons

**DOI:** 10.1002/chem.202002638

**Published:** 2020-09-02

**Authors:** Dominik Lungerich, Jakob Felix Hitzenberger, Michael Ruppel, Tibor Döpper, Matthias Witt, Ivana Ivanović‐Burmazović, Andreas Görling, Norbert Jux, Thomas Drewello

**Affiliations:** ^1^ Department of Chemistry and Pharmacy, & Interdisciplinary Center for Molecular Materials (ICMM) Organic Chemistry II Friedrich-Alexander-University Erlangen-Nuernberg Nikolaus-Fiebiger-Str. 10 91058 Erlangen Germany; ^2^ Center for Nanomedicine Institute for Basic Science (IBS) Seoul 03722 Republic of Korea; ^3^ Graduate Program of Nano Biomedical Engineering (NanoBME) Advanced Science Institute Yonsei University Seoul 03722 Republic of Korea; ^4^ Department of Chemistry and Pharmacy Physical Chemistry I Friedrich-Alexander-University Erlangen-Nuernberg Egerlandstrasse 3 91058 Erlangen Germany; ^5^ Department of Chemistry and Pharmacy Theoretical Chemistry Friedrich-Alexander-University Erlangen-Nuernberg Egerlandstrasse 3 91058 Erlangen Germany; ^6^ Bruker Daltonics GmbH Fahrenheitstrasse 4 28359 Bremen Germany; ^7^ Department of Chemistry and Pharmacy Bioinorganic Chemistry Friedrich-Alexander-University Erlangen-Nuernberg Egerlandstrasse 1 91058 Erlangen Germany

**Keywords:** annulenes, C−F activation, collision-induced dissociation, mass spectrometry, polycyclic aromatic hydrocarbons, porphyrinoids

## Abstract

Geodesic nitrogen‐containing graphene fragments are interesting candidates for various material applications, but the available synthetic protocols, which need to overcome intrinsic strain energy during the formation of the bowl‐shaped skeletons, are often incompatible with heteroatom‐embedded structures. Through this mass spectrometry‐based gas‐phase study, we show by means of collision‐induced dissociation experiments and supported by density functional theory calculations, the first evidence for the formation of a porphyrin‐embedded conical nanocarbon. The influences of metalation and functionalization of the used tetrabenzoporphyrins have been investigated, which revealed different cyclization efficiencies, different ionization possibilities, and a variation of the dissociation pathway. Our results suggest a stepwise process for HF elimination from the *fjord* region, which supports a selective pathway towards bent nitrogen‐containing graphene fragments.

## Graphical abstract

**Porphyrin in a cone**! The preparation of a porphin at the tip of a carbon nanocone has been achieved by the transformation of fluorinated tetraaryltetrabenzoporphyrins in the gas phase through collision‐induced dissociation. In this process, a remarkable selectivity of *fjord*‐region C−F activation was found. These findings allow considering novel strategies to access this type of nitrogen‐containing carbon allotrope and to apply them in novel hybrid materials.

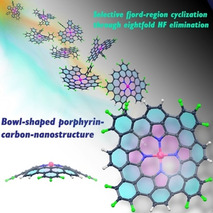

## Introduction

**Figure 1:**
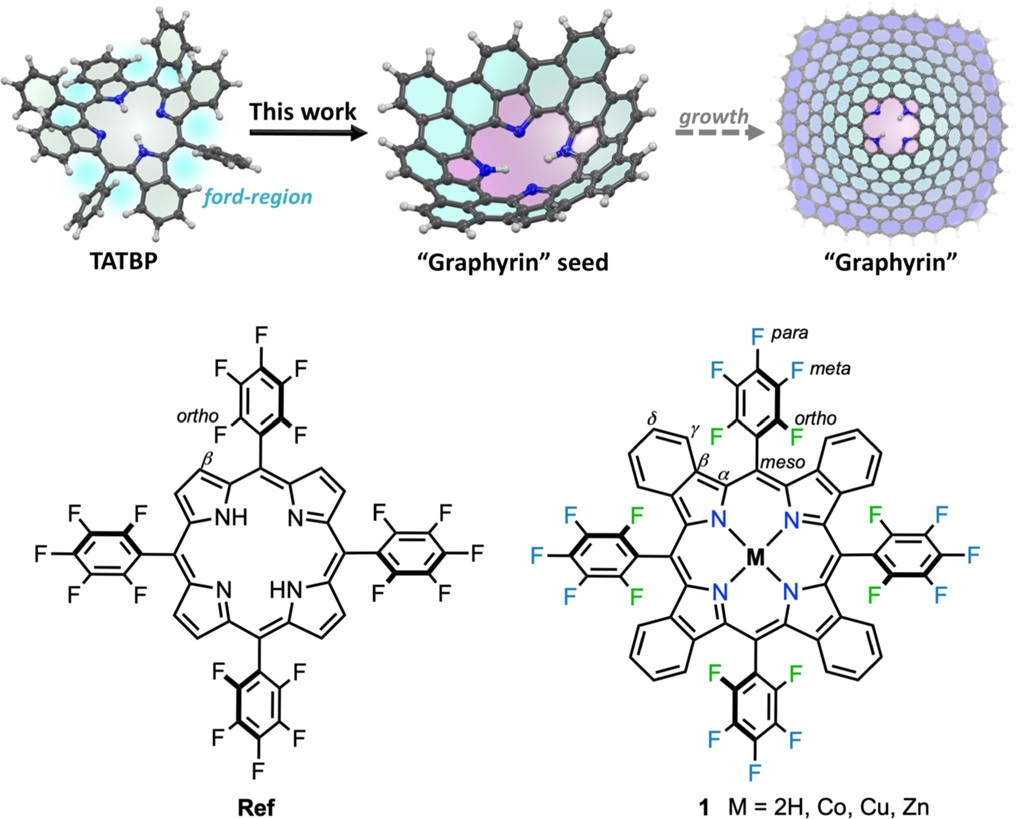
Scope and inspiration of this work.

The positive curvature‐induced distortion of polycyclic aromatic hydrocarbons (PAHs) from planar to bowl‐shaped geometries can be traced back to the 1960s, when Barth and Lawton first synthesized corannulene in a tour de force of 17 steps.^1^ However, the development of synthetic strategies such as flash vacuum pyrolysis (FVP) for the preparation of tailor‐made precursors, which was widely exploited by Scott et al. in the 1990s, allowed for the preparation of corannulene in only three steps in an overall yield of 24 %.^2^ Finally, the contributions of Sygula, Rabideau, and Siegel and co‐workers catapulted corannulene from a high‐end molecule to a starting material, these days available in kilogram quantities.^3^ That said, the rising interest in unprecedented curved PAHs in materials applications^4^ has accelerated the development of novel synthetic methodologies immensely.^5^ Nowadays, to avoid temperatures of up to 1100 °C, which are often required for FVP,^6^ milder strategies towards strained PAHs dominate the synthetic landscape. They encompass, for example, palladium‐catalyzed C−H activation^7^, ^8^ and oxidative C−C bond formation (Scholl oxidation).^9^ Also, the activation of the C−F bond has emerged as a powerful tool and has been demonstrated in the gas phase,^10^ via silylium carboranes,^11^ and on activated alumina.^12^ Although very efficient for all‐carbon skeletons, the latter C−F activation strategies unfortunately show severe incompatibilities with heteroatom‐containing precursors, which leaves the fraction of newly discovered nitrogen‐containing buckybowls rather small.^8^, ^13^ So far, the strategy of surface‐assisted formation of nitrogen‐containing buckybowls appears to be one of the few promising pathways to follow.^14^ However, if we consider one of the most prominent classes of nitrogen‐containing molecules, namely the porphyrinoids, geodesic structures have been realized for inherently bowl‐shaped subporphyrins as well as four‐fold *o*‐phenyl‐β‐porphyrin five‐membered‐ring fused porphyrins.^15^ With the porphyrin core embedded in the center of the carbon‐rich architecture, however, only planar derivatives, which were either attached^16^ or fused to larger PAHs,^17^ were realized. However, complete embedment of the porphin core into a graphene‐like carbon sheet, as depicted in Figure 1, has never been achieved, not even through on‐surface‐assisted syntheses.^18^

Due to the intrinsic structure of tetraaryltetrabenzoporphyrins (**TATBP**s), it is tempting to use this skeleton as a precursor for the bottom‐up preparation of a porphyrin‐incorporated conical graphene fragment (“graphyrin”). Due to its periphery, which comprises six‐membered rings, the structure can serve as a seed for growth into large graphene flakes (see Figure 1 top). Further, a rich metal complexation chemistry could produce a large variety of electronic and chemical fine‐tuning options. Hence, such a material would allow us to contemplate several potential applications, ranging from nano(opto)electronics to catalysis.^19^ Yet, unlike the reactivity of the β position in porphyrins,^20^ the activation of the respective γ position in **TATBP**s turns out to be rather difficult. All our attempts to cyclize **TATBP**s under common conditions, such as oxidative coupling,^21^ which has been successfully applied to porphyrins before, have failed.^22^ Even surface‐assisted methods on Cu(111) did not yield completely cyclized products.^23^ Thus, inspired by the work in the gas phase of Brückner and co‐workers, who reported *o*‐phenyl–β‐porphyrin five‐membered ring fusion in 5,10,15,20‐tetrakis(pentafluorophenyl)porphyrin (**Ref**),^24^ we intended to overcome the challenging activation of the γ position by using pentafluorophenyl‐substituted tetrabenzoporphyrin **1** (see Figure 1 bottom). In this work, we examined the possible formation of a conical porphyrin‐embedded nanocarbon by means of collision‐induced dissociation (CID) experiments in a mass spectrometer at ultrahigh resolution. Our findings were supported by density functional theory (DFT) calculations and additional mechanistical investigations performed by varying the substituents.

## Results and Discussion

### General information

**Figure 2:**
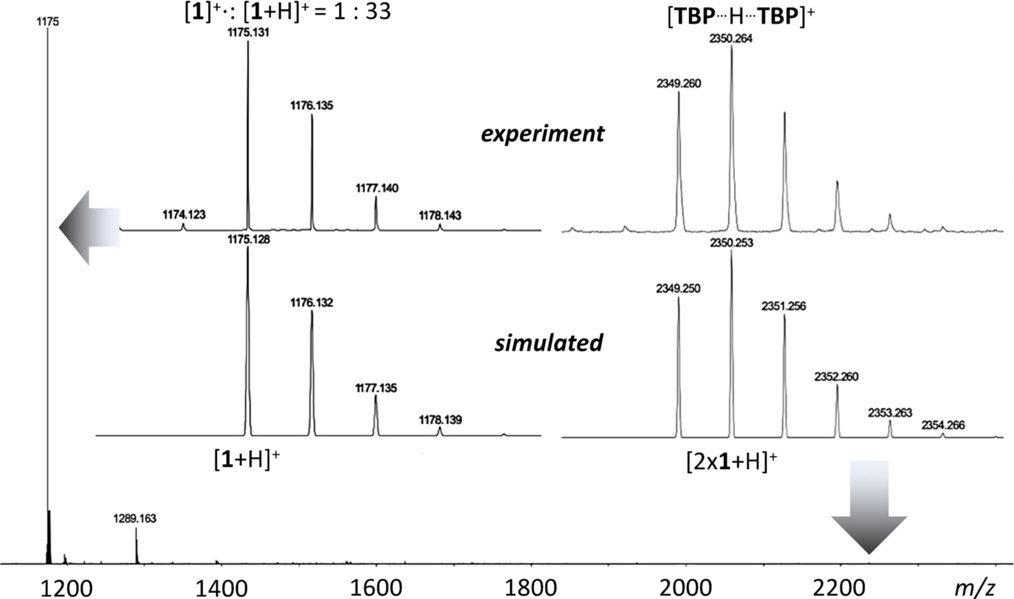
Nature of the ions produced from free‐base tetrabenzoporphyrins upon ESI.

Prior to the cyclization experiments, we clarify here the nature of the precursor ion, which is of importance for the dissociation behavior. In electrospray ionization (ESI) mode, free‐base tetrabenzoporphyrins (**TBP**s) mainly form protonated quasi‐molecular ions, accompanied by a significantly less abundant radical cation and a proton‐bridged dimer of the type [**TBP**⋅⋅⋅H⋅⋅⋅**TBP**]^+^, as demonstrated for **1** in Figure 2. Negligible traces of ions with alkali metals as charge carriers, such as Na^+^ and K^+^, highlight the preference for competitive proton uptake. The high basicity of the inner nitrogen atoms results from the increased density of π electrons in the pyrrole rings, caused by the distortion of the otherwise planar porphyrin macrocycle.^25^

By contrast, metalated benzoporphyrins are ionized to yield radical cations [**TBP**]^+.^ (see the Supporting Information). In the following, a dissociation leading to the desired C−C bond formation is termed “cyclization”, and undesired side reactions are referred to as “fragmentation”. After ionization, the benzoporphyrin parent ion is selected by a quadrupole mass analyzer, with further activation of the parent ions triggered by collision‐induced energy transfer in a collision quadrupole inside the mass spectrometer. Such tandem mass spectrometric (MS^2^) experiments allow quantitative comparison of the ion ratios. The fragmentation energy is provided by multiple collisions of the precursor ion and resulting fragment ions with the neutral collision gas (nitrogen). Accelerating the precursor ion to 80–110 eV prior to entering the collision quadrupole increases the energy of the collision between the precursor ion and collision gas. For ultrahigh mass spectrometric analysis of the dissociation products, we used a Fourier‐transform ion cyclotron resonance mass spectrometer (FT‐ICR‐MS) with a maximum resolution of 2 000 000 and sub‐ppm mass accuracy.

### Collision‐induced dissociation of 1

**Scheme 1:**
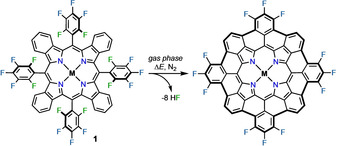
Reaction of **1** to yield bowl‐shaped **TBP**s by HF elimination in the gas phase. M=2H, Co, Cu, Zn.

**Figure 3:**
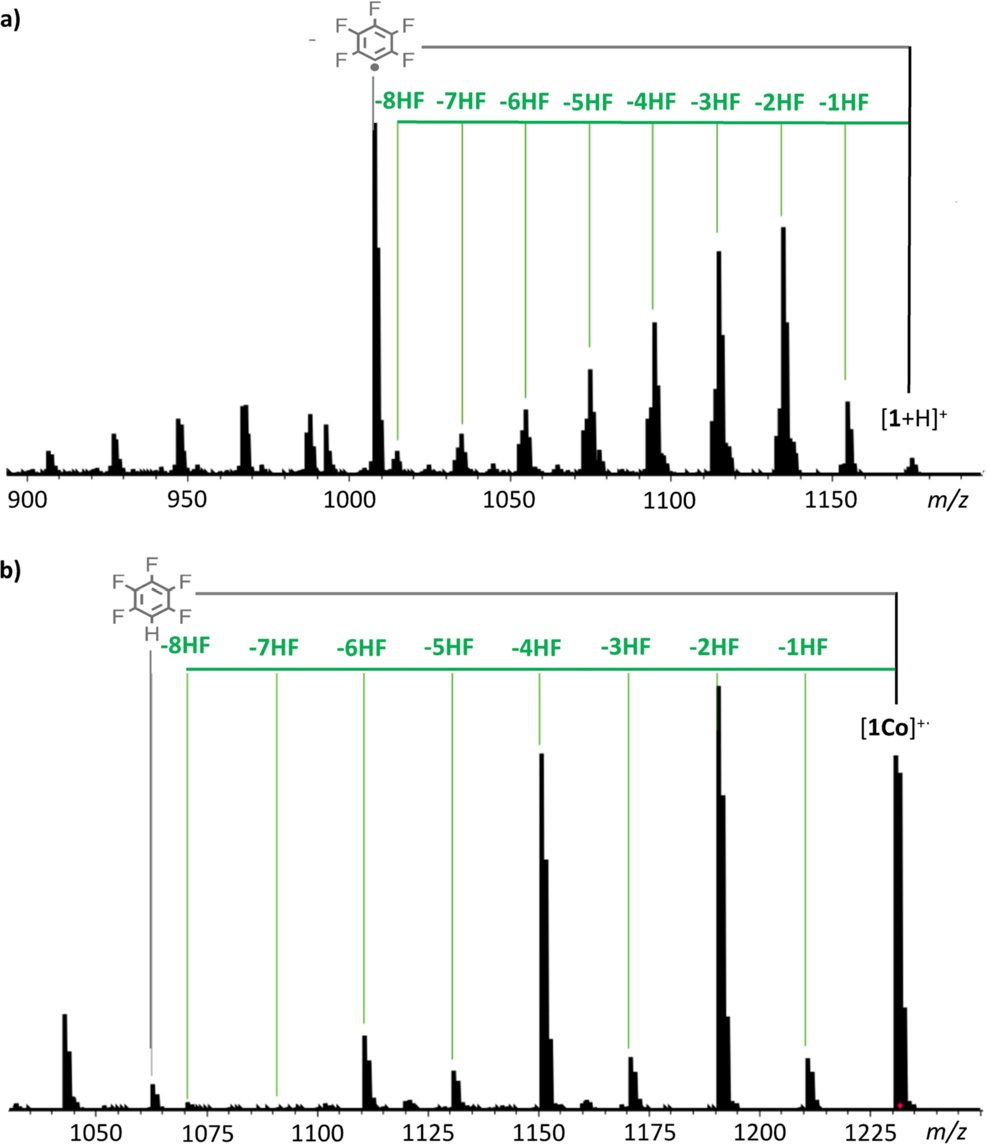
Positive‐ion CID (MS^2^) mass spectra of a) [**1**+H]^+^ and b) [**1Co**]^+.^.

To investigate the eight‐fold γ–*ortho* cyclization to the cone‐shaped benzoporphyrin, we chose pentafluorophenyl‐substituted **1** as candidate (see Scheme 1). In the following, the dissociation of **1** is generic for all the investigated cyclization reactions and shall be discussed in detail for the protonated free‐base [**1**+H]^+^ and the radical cation of the cobalt‐complexed benzoporphyrin [**1Co**]^+⋅^. As depicted in Figure 3 a, the behavior of [**1**+H]^+^ follows two major reaction pathways. On the one hand, cyclization occurs to various degrees by stepwise elimination of hydrogen fluoride. On the other hand, the loss of a pentafluorophenyl radical (^.^C_6_F_5_) is also observed.^24^, ^26^ The intensity of the signal arising from this side reaction is rather high in comparison with that from the fragmentation of the fluorinated porphyrin **Ref**, which serves as a reference in our study (see the Supporting Information), under similar conditions.^24^ Still, the sum of the peak intensities for the stepwise cyclization of [**1**+H]^+^ remains higher than the intensity of the fragmentation peak (see below).^27^ The nature of the cyclization process requires, for the case of eight consecutive reactions, high energies to be provided. In this context, the extent to which the cyclization is preferred is astonishing, and that an eight‐fold cyclization can be observed at all, is even more so (see Figure 3). Importantly, due to the ultrahigh resolution of FT‐ICR‐MS, the dissociation processes can be clearly distinguished from each other, which allows the quantification of the peak ratios. The loss of a second ^.^C_6_F_5_ fragment does not occur. Instead, a combination of ^.^C_6_F_5_ loss and stepwise cyclization is observed. Additional side reactions that produce peaks of minor intensity include the loss of multiple hydrogen atoms. These originate most likely from the protonated inner nitrogen atoms.^28^ The loss of F^.^ radicals does not take place;^29^ only at very high energies does the complete breakdown of the molecule occur.

By contrast, the pathway followed by [**1Co**]^+.^ predominantly features the desired cyclization reaction, and, contradictory to the literature,^30^ the loss of pentafluorobenzene (C_6_F_5_H) is negligible (Figure 3 b). The loss of one or two hydrogen atoms is not observed for the metalated species. This supports the hypothesis made earlier for [**1**+H]^+^, that the hydrogen losses stem from the inner N*H*. A second, even more pronounced difference in the fragmentation behavior of the metalated species lies in the observed intensity pattern of the cyclization products. Although for the free‐base **TBP**, C−C bond formation proceeds stepwise, for the metalated **TBP**, the cyclization occurs predominantly by the preference for even‐numbered eliminations of HF molecules. The latter behavior can be explained by slow odd‐numbered HF elimination steps (*n*=1, 3, 5, 7) followed by fast even‐numbered eliminations (*n*=2, 4, 6, 8). The same behavior was observed for the metalated derivatives [**1Cu**]^+.^ and [**1Zn**]^+.^ (see Table 1 and Chapter S3 in the Supporting Information).

**Table 1:** Summary of the maximum number of hydrogen halide losses from the investigated **TATBP**s.

Ionized monomer	Type of X at phenyl	HX loss	Ionized monomer	Type of X at phenyl	HX loss
[**1**+H]^+^	F_5_‐phenyl	8	[**5**+H]^+^	4 *p‐*F	0
[**1Co**]^+.^	F_5_‐phenyl	8	[**6**+H]^+^	4 *o‐*F	4
[**1Cu]** ^+.^	F_5_‐phenyl	8	[**6Pd**]^+.^	4 *o‐*F	1
[**1Zn**]^+.^	F_5_‐phenyl	8	[**7**+H]^+^	8 *o*‐F, 4 *p*‐Br	5
[**2**+H]^+^	8 *o‐*F	7	[**8**+H]^+^	8 *o‐*Cl	7
[**3**+H]^+^	8 *m‐*F	0	[**8Pd**]^+.^	8 *o‐*Cl	5
[**4**+H]^+^	12 *m*,*p‐*F	0	[**Ref**+H]^+^	F_5_‐phenyl	8

### Computational analysis

**Figure 4:**
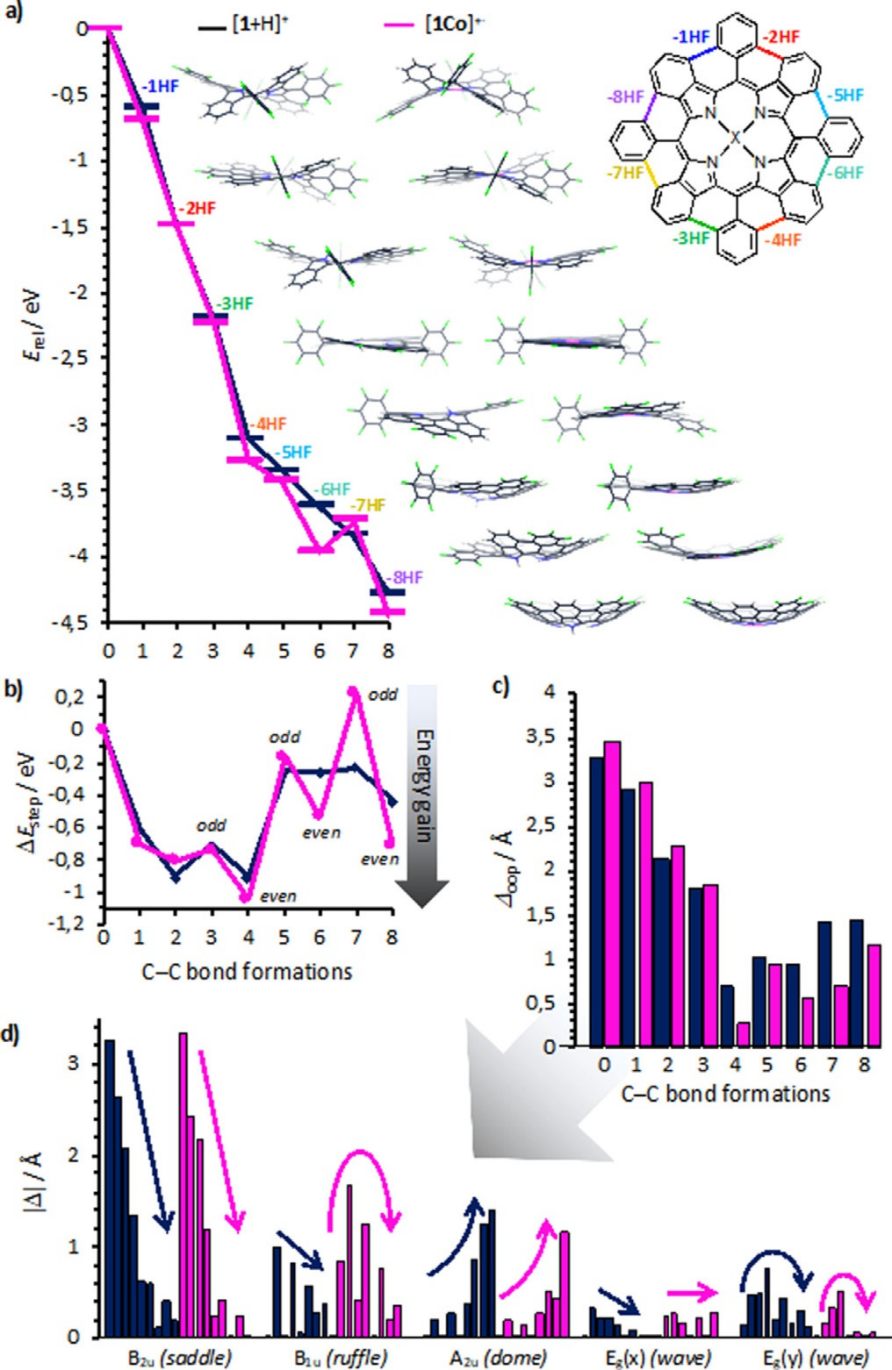
Summary of the computational analysis of the stepwise C−C bond formation through HF elimination for the ions [**1**+H]^+^ (dark blue), [**1Co**]^+.^ (magenta) and their intermediates performed at the DFT ωB97X‐D/6‐311G(d,p) level of theory. a) Relative reaction enthalpies of the geometry‐optimized intermediates. The energy of the formed HF is included. b) Relative energy difference for each reaction step (1–8). c) Summary of the NSD analysis for the overall out‐of‐plane distortion of each structure. d) Selected out‐of‐plane modes; arrows indicate the trend for 0 to 8 C−C bond formations.

To shed light on this phenomenon, we performed DFT calculations on the ionic species at the ωB97X‐D/6‐311G(d,p) level of theory; the results are summarized in Figure 4.^31^ From the calculated reaction enthalpies shown in Figure 4 a, it is evident that the overall cyclization by HF elimination is energetically favored; particularly upto the formation of the nearly planar −4 HF intermediate (for enlarged structures, see Figures S82 and S83 in the Supporting Information). The subsequent C−C bond formations proceed, albeit energetically less favorably due to increasing internal strain energy, still exothermically until completion of eight‐fold γ–*ortho* cyclization. For the metalated species, only the odd‐numbered step to [**1Co**−7 HF]^+.^ is endothermic, which is surprisingly well reflected in the virtual absence of the corresponding signal in the mass spectrum (see Figure 3 b). Internal strain, due to steric repulsion between the isoindole moiety and the in‐plane‐forced aryl moiety of the odd‐numbered cyclization steps, is also well reflected in the relative energy difference per step, as depicted in Figure 4 b. Thus, the odd‐numbered C−C bond formations show less energy gain than the even‐numbered eliminations. This feature is particularly true for the metalated derivatives, which mirrors very well the experimental observations of more favorable even‐numbered elimination steps (see Figure 3) and strongly supports the formation of the proposed bowl‐shaped structure rather than skeletal rearrangements. The origin of this behavior is ascribed to the increased internal strain in the metalated derivatives, which disfavors greater rotations of the isoindole moieties in the porphyrin skeleton due to metal complexation.

From normal‐coordinate structural decomposition (NSD) analysis, which was introduced by Shelnutt and co‐workers as a means to quantitatively analyze the various vibrational modes of the porphyrin skeleton,^32^ it becomes evident that the overall out‐of‐plane distortion Δ_oop_ steadily decreases from Δ_oop_([**1**+H]^+^)=3.258 Å and Δ_oop_([**1Co**]^+.^)=3.449 Å until the −4 HF intermediate, and then increases again to Δ_oop_([**1**−8 HF+H]^+^)=1.424 Å and Δ_oop_([**1Co**−8 HF]^+.^)=1.170 Å, respectively (Figure 4 c). As indicated by the trend arrows in Figure 4 d, for both species, the major contribution to distortion initially stems from the B_2u_ mode (saddling), whereas the major contribution to the distortion of the final intermediates and product stems from the A_2u_ mode (doming). However, the intermediate structures of [**1**−*x* HF+H]^+^ also show strong contributions from the E_g_(*y*) mode (waving), whereas [**1Co**−*x* HF]^+.^ exhibit a stronger tendency for ruffling (B_1u_ mode). Detailed information on the complete NSD analysis can be found in the Supporting Information.

### Mechanistic investigation

**Figure 5:**
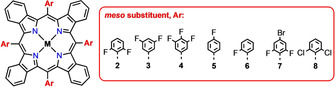
Investigated fluorinated **TBP 2**–**7** and chlorinated **TBP 8**.

The mechanism for the cyclization process cannot be drawn from the observed dissociation of **1** or **Ref**;^24^ possible HF eliminations of δ‐hydrogen atoms or *m*/*p*‐fluorine atoms, or skeletal rearrangements, cannot be ruled out. Thus, we also prepared **TBP 2**–**7** (Figure 5) bearing precisely chosen F_*n*_‐phenyls (*n*=1–3), with the fluorine atoms in strategically placed positions (*ortho*, *meta*, *para*).^33^

**Figure 6:**
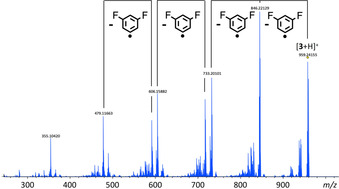
Positive‐ion CID (MS^2^) mass spectrum of [**3**+H]^+^.

**Figure 7:**
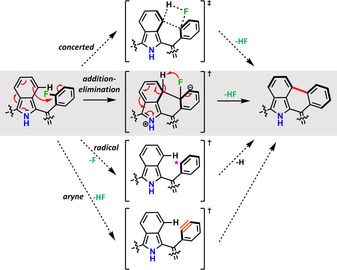
Plausible mechanisms for the *fjord*‐region γ–*ortho* cyclization by HF elimination.

Briefly, [**2**+H]^+^ mainly shows, as in the case of [**1**+H]^+^, the desired cyclization reaction by HF elimination, however only up to seven times. With respect to the mechanism, fragmentation by loss of F^.^ radicals was not observed, which makes a mechanism by radical formation unlikely, although the formation of arynes by means of 1,2 elimination of HF, which could undergo the desired γ–*ortho* cyclization, remains possible. The formation of arynes is also formally possible for compounds **3**, **4** and **5**, while only **3** and **4** can form an “*ortho*‐aryne”; however, the outcomes of CID of [**3**+H]^+^, [**4**+H]^+^, and [**5**+H]^+^ contrast starkly with the observations made for [**1**+H]^+^ and [**2**+H]^+^. As demonstrated in Figure 6, CID of [**3**+H]^+^ leads almost exclusively to fragmentation, with virtually no yield of the desired cyclization products. Again, the loss of F^.^ radicals is not observed; however, up to four distinct ion signals corresponding to fragmentation by loss of the respective aryl radicals are observed, which reflects the total decomposition of the parent ion. The same is true for [**4**+H]^+^ and [**5**+H]^+^, as shown in Chapter S4 in the Supporting Information. With **3**–**5** all lacking fluorine atoms in the *ortho* positions, the need for *o*‐fluorine atoms becomes evident, and would support a mechanism by a concerted transition state. However, concerted mechanisms are rather unfavored in gas‐phase reactions. Therefore, a two‐step mechanism involving first C−C bond formation by a nucleophilic aromatic addition reaction from the electron‐rich benzo ring to the electron poor F_*n*_‐aryl ring, followed by 1,2‐elimination of HF becomes the most plausible pathway (see Figure 7). Further experiments with monofluorophenyl‐substituted [**6**+H]^+^ show, as expected, four cyclization steps, which is the maximum possible HF eliminations in this scaffold, whereas [**6Pd**]^+.^ shows only one successful cyclization. A likely explanation for this observation is the lack of flexibility in [**6Pd**]^+.^, due to tight metal complexation (as discussed for [**1Co**]^+.^), compared with [**6**+H]^+^; for symmetry reasons, flexibility is necessary for the formation of the energetically less favorable structures that result from the monofluoro substitution pattern. Finally, the selectivity for *fjord*‐region HF elimination is underpinned by [**7**+H]^+^, which includes four *p*‐bromine substituents. Even though fragmentation by loss of one Br^.^ radical occurs, it is not the major dissociation pathway, and the cyclization of up to five C−C bonds could be identified. The reduced number of HF eliminations can be ascribed to the energy loss incurred by Br^.^ fragmentation (see Figure S69 in the Supporting Information).

To identify the effect of the nature of the halogen, we carried out CID experiments with *o*,*o*‐dichlorinated derivative **8**. For [**8**+H]^+^, a maximum of up to seven C−C bonds were formed (−7 HCl), but also a significant number of intermediates featuring the loss of Cl^.^ (see Chapter S2 in the Supporting Information). Apparently, the soft character of the C−Cl bonds leads to a less selective dissociation pathway. Even though [**2**+H]^+^ also underwent only seven C−C bond formations, there is a major difference between [**8**+H]^+^ and [**2**+H]^+^: in the case of [**8**+H]^+^ bearing chloride atoms, the cyclization is 15 % less abundant than the fragmentation by loss of the *meso* substituent (^.^C_6_H_3_Cl_2_). In contrast, [**2**+H]^+^ shows mainly the desired cyclization reaction by HF elimination, resulting in a yield of 34 % per step.^27^

Energy‐dependent CID experiments not only provide additional information on the different energy demands of the transformations, but also quantification of the individual yields of the cyclizations and the side reactions. Thus, the so‐called survival yields (SY) were plotted as a function of the collision energy in the center‐of‐mass frame (*E*_COM_), which is generated from the measured laboratory frame collision energy (*E*_lab_) and the masses of the collision gas and the precursor ion according to Equation (1).^34^

(1) $$E_{COM}=E_{lab}\frac{m_{collision\;gas}}{m_{collision\;gas}+m_{precursor\;ion}}$$

**Figure 8:**
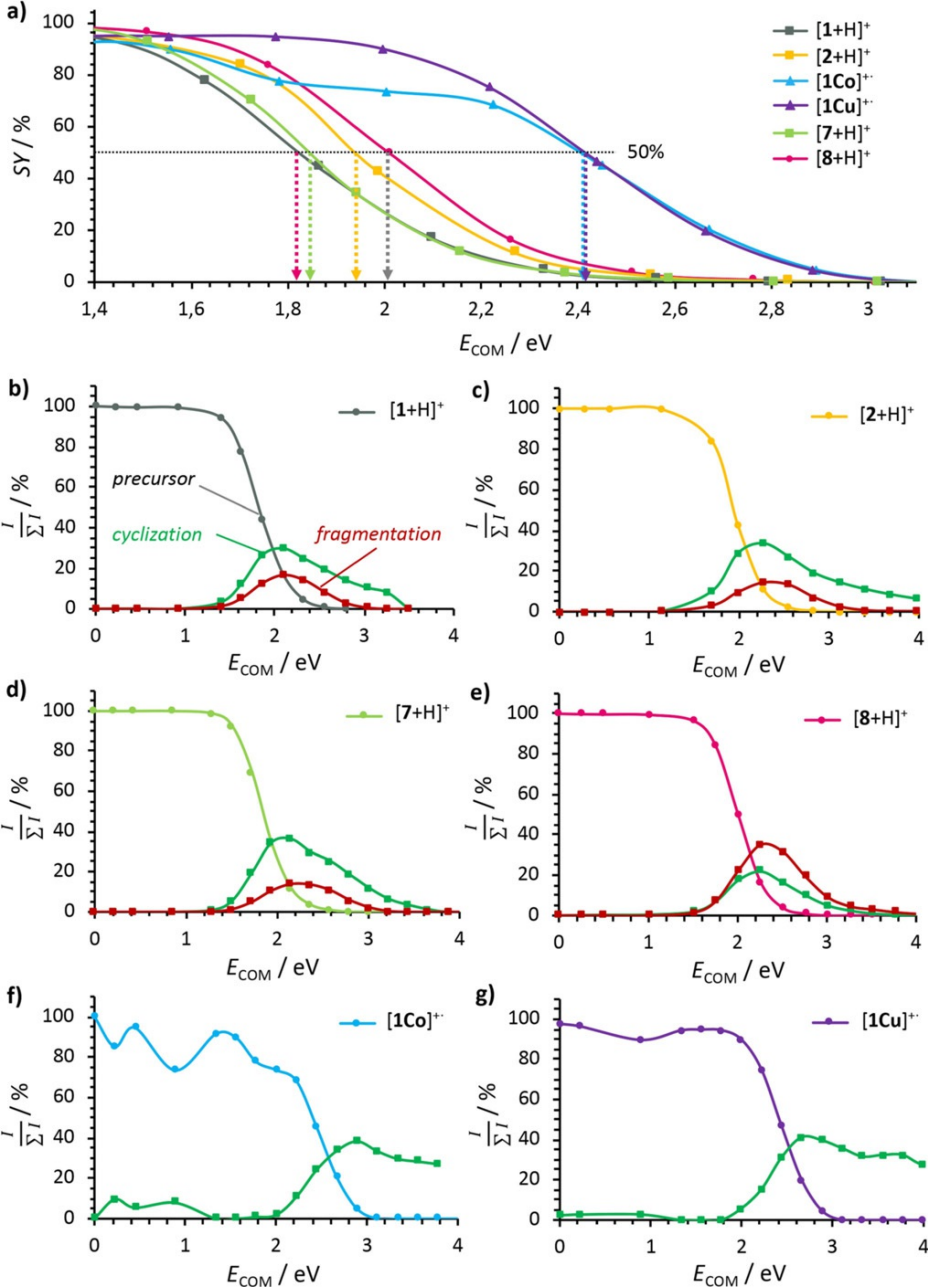
Energy‐dependent CID breakdown graphs: a) Survival yield (SY) in the precursor ion region and relative intensity ratio for b) [**1**+H]^+^, c) [**2**+H]^+^, d) [**7**+H]^+^, e) [**8**+H]^+^, f) [**1Co**]^+.^, and g) [**1Cu**]^+.^.The precursors in b–g are depicted according to the respective color in (a), the products of cyclization steps 1–8 in green, and the side reaction of the aryl radical fragmentation in red. The *x* axis represents the center‐of‐mass collision energy (*E*_COM_) and the *y* axis the ratio of the precursor or fragment ion intensity over the sum of the intensities of all signals (I/∑I ).

The breakdown graphs of the energy‐dependent CID experiments are displayed in Figure 8. They were obtained under multiple collision conditions and provide relative rather than absolute data on the energy demands of the CID processes. The decay reactions of the free‐base porphyrin precursor ions, summarized in Figure 8 a, are identified as the least‐energy‐demanding processes. The decay of the pentafluorophenyl‐ and 2,6‐difluoro‐4‐bromophenyl‐substituted **TBP**s [**1**+H]^+^ and [**7**+H]^+^ commences at the lowest collision energy (*E*_COM_=1.8 eV at 50 % SY), closely followed by the dissociation of [**2**+H]^+^ and [**8**+H]^+^ (*E*_COM_=1.9 and 2.0 eV at 50 % SY, respectively). We attribute the slightly lower energy requirement of [**1**+H]^+^ and [**7**+H]^+^ to the electron‐poor nature of the aryl group, which reduces the energy demand for the cyclization reaction compared with the di‐*ortho*‐substituted derivatives [**2**+H]^+^ and [**8**+H]^+^. A marked shift towards higher collision energies is evident for the decay of the metalated precursor ions [**1Co**]^+.^ and [**1Cu**]^+.^ (*E*_COM_=2.4 eV at 50 % SY). We attribute this to the fact that 1) metalation rigidifies the backbone, thereby making the necessary conformational changes energetically more demanding and 2) the cyclization of metalated porphyrins favors even‐numbered eliminations, that is, a first slow, energy‐demanding step for generating a rather strained species, followed by a second, fast, likely strain‐energy‐releasing elimination at the same aryl ring (generating a picene). In each individual case, the cyclization and side reaction (fragmentation) start to proceed at matching energies, as depicted in Figure 8 b–g. However, as discussed earlier, the significance of the side reaction is higher for [**8**+H]^+^ (Figure 8 e), whereas the fluorinated derivatives prefer the desired cyclization (Figure 8 b–d). Fragmentation is virtually suppressed for metalated derivatives, as depicted in Figure 8 f,g.

Interestingly, despite the dramatically high homolytic bond dissociation enthalpy of the C−F bond (C_6_H_5_−F: 533 kJ mol^−1^) compared with the C−C bond (C_6_H_5_−C_6_H_5_: 494 kJ mol^−1^),^35^ the cyclization reaction is preferred in the fluorinated system over the fragmentation by loss of the aryl radical, which further underpins the exclusion of a radical pathway and supports an addition‐elimination reaction mechanism.^22^ A further driving force for this process can be attributed to the high standard heat of formation for hydrogen fluoride (Δ_f_ *H*⊖(HF)=273 kJ mol^−1^) and the gain in entropy.^36^ On the other hand, the low homolytic bond dissociation enthalpy of C−Cl (C_6_H_5_−Cl: 407 kJ mol^−1^), paired with the observation of Cl^.^ radical losses and a higher aryl radical fragmentation ratio, make a radical pathway for [**8**+H]^+^ appear to be likely.

Hence, in view of the data in Table 1, it becomes clear that the maximum number of halide losses is dependent on the nature of functionalization, that is, the type and position of the halide as well as the state of the inner nitrogen cavity (free‐base/metalated).

### Wet‐chemical approach with 8

**Figure 9:**
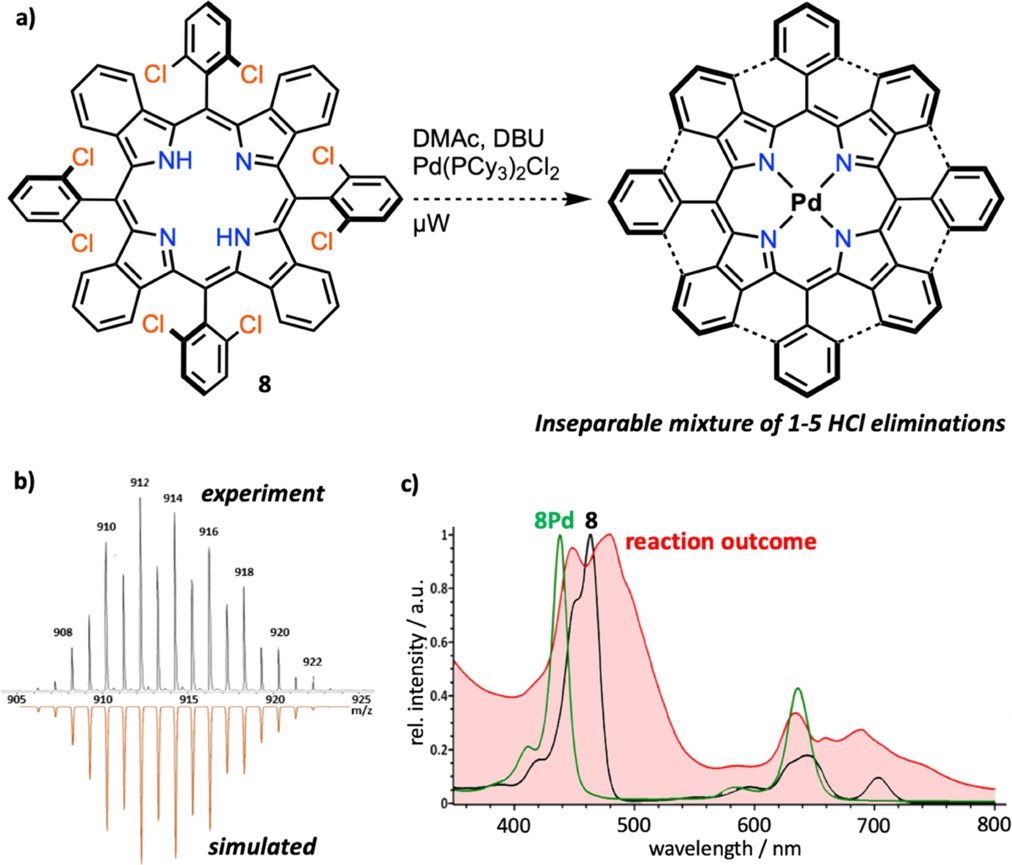
Outcome of wet‐chemical approach towards eight‐fold γ–*ortho* cyclization. a) Best result achieved in a microwave reactor with **8**, [Pd(PCy_3_)_2_Cl_2_] (8 equiv), DMAc/DBU (4:1, v:v), N_2_, 180 °C, 4 h; then silica gel plug filtration. b) Enlargement of the mass spectrum of the reaction outcome (simulation suggests a mixture of [**8Pd**−5 HCl−3 Cl+3 H]^+^/[**8Pd**−4 HCl−4 Cl+H]^+^/[**8Pd**−3 HCl−5 Cl+5 H]^+^/[**8Pd**−2 HCl−6 Cl+6 H]^+^/[**8Pd**−1 HCl−7 Cl+7 H]^+^=1:7:5:4:4. c) Normalized UV/Vis spectra of **8**, **8Pd**, and the isolated reaction outcome.

To translate our gas‐phase studies to the preparative scale, we attempted to cyclize fluorinated derivatives **1** and **2** by transition‐metal‐catalyzed methods using, for example, [Ni(dppp)Cl_2_] (dppp=1,3‐bis(diphenylphosphino)propane) as catalyst.^37^ However, aside from metalation of the porphyrin core, none of the fluorinated derivatives showed any reactivity. Therefore, we focused our investigations on 2,6‐dichlorophenyl‐substituted benzoporphyrin **8**, because the C−H activation of chlorinated polycyclic aromatic hydrocarbons has been successfully applied in the past to the synthesis of strained molecules, such as indenocorannulenes (see also Chapter S1 in the Supporting Information).7a, 38 We screened several catalytic systems, including Pd(OAc)_2_, [Pd(PPh_3_)_2_Cl_2_], and [Pd(PCy_3_)_2_Cl_2_] (Cy=cyclohexyl). The best outcome was observed by using the conditions published by Scott and co‐workers, namely with [Pd(PCy_3_)_2_Cl_2_] as catalyst in dry *N*,*N*‐dimethylacetamide (DMAc) and 1,8‐diazabicyclo[5.4.0]undec‐1‐ene (DBU) as base heated at 180 °C in a microwave reactor for 4 h (Figure 9 a).^38^ The mass spectrum shows several reaction features that could be identified (compare Figure 9 b): palladium complexation occurred quantitatively when free‐base **8** was used, and successful C−C bond formation was observed by the dehydrochlorination of up to five bonds; residual chlorine atoms were substituted by hydrogen atoms to give completely dehalogenated products. However, chromatographic separation of the reaction products could not be achieved. In the UV/Vis absorption spectrum of the reaction products, the π extension of the porphyrin core is evident by significant broadening and a bathochromic shift of the absorption characteristics, as depicted in Figure 9 c. Virtually the same results were obtained when starting from **8Pd**. Interestingly, in addition to the clear advantages of studying reactions in the gas phase,^39^ such as solvent‐free conditions, suppression of polymerization, and direct identification of the reaction products, the results obtained from the gas‐phase CID experiments of [**8Pd**]^+.^ are equivalent to the palladium‐catalyzed wet‐chemical approach (see Table 1). From this we can conclude that palladium complexation occurs faster than the respective catalytic C−H activation reaction, and that chlorinated species **8** and **8Pd** are not suitable candidates for the eight‐fold cyclization. That said, it is important to find a suitable wet‐chemical method that is able to activate C−F bonds in the presence of a porphyrin core in order to prepare the graphyrin seed on a preparative scale.

## Conclusions

In summary, we have shown strong experimental evidence for the successful formation of a porphyrin‐embedded conical graphene fragment by means of *fjord*‐region selective C−C bond formations mediated by eight‐fold HF elimination. To our surprise, we found in our MS^2^ experiments, despite the unfavorable situation of mere singly bonded *meso*‐aryl substituents, that the desired cyclization process is preferred over the fragmentation of peripheral aryls. From all the evidence obtained, we conclude that the bond‐forming mechanism most likely involves C−C bond formation by nucleophilic addition followed by 1,2‐elimination of HF, rather than a concerted mechanism or a two‐step process comprising the formation of reactive aryl radicals or arynes. Even though metal complexation leads to an increased demand for collision energy in the dissociation process, the cyclization of the respective radical cations proceeds, unlike in the case of protonated free‐base derivatives, with high selectivity and suppressed tendency for *meso*‐aryl fragmentation. The higher demand for collision energy in metalated species originates from the fact that the cyclization is more favorable for even‐numbered than for odd‐numbered HF elimination steps. Although in the past the HF elimination strategy has been successfully applied to precursors with rigid, annulated F_*n*_‐benzo moieties for the formation of strained all‐carbon architectures, this study represents the first example of its application to heteroatom‐containing molecules, which furthermore tolerates singly bonded F_*n*_‐aryls. The similar outcomes for the CID and palladium‐catalyzed wet‐chemical experiments of **8** suggest that the results obtained in the mass spectrometer are not only gas‐phase specific, but reflect well the suitability of each precursor benzoporphyrin for preparative approaches. Hence, the successful detection of a bowl‐shaped graphene–porphyrin hybrid (“graphyrin”) raises the expectations of preparing this structural motif on a preparative scale. We believe that the development of novel synthetic strategies that are able to activate the C−F bond in the presence of heteroatoms will allow the development of these systems for future nanotechnological and materials applications.

## Conflict of interest

The authors declare no conflict of interest.

## Supporting information

As a service to our authors and readers, this journal provides supporting information supplied by the authors. Such materials are peer reviewed and may be re‐organized for online delivery, but are not copy‐edited or typeset. Technical support issues arising from supporting information (other than missing files) should be addressed to the authors.

Supplementary

## References

[1a] W. E. Barth , R. G. Lawton , J. Am. Chem. Soc. 1966, 88, 380–381;

[1b] R. G. Lawton , W. E. Barth , J. Am. Chem. Soc. 1971, 93, 1730–1745.

[2] L. T. Scott , P. C. Cheng , M. M. Hashemi , M. S. Bratcher , D. T. Meyer , H. B. Warren , J. Am. Chem. Soc. 1997, 119, 10963–10968.

[3a] A. Sygula , P. W. Rabideau , J. Am. Chem. Soc. 2000, 122, 6323–6324;

[3b] A. Sygula , P. W. Rabideau , J. Am. Chem. Soc. 1999, 121, 7800–7803;

[3c] T. J. Seiders , E. L. Elliott , G. H. Grube , J. S. Siegel , J. Am. Chem. Soc. 1999, 121, 7804–7813;

[3d] M. A. Petrukhina , L. T. Scott , Fragments of Fullerenes and Carbon Nanotubes: Designed Synthesis Unusual Reactions, and Coordination Chemistry, Wiley, 2011;

[3e] A. M. Butterfield , B. Gilomen , J. S. Siegel , Org. Process Res. Dev. 2012, 16, 664–676.

[4] T. A. Schaub , Angew. Chem. Int. Ed. 2020, 59, 4620–4622; 10.1002/anie.201914830 31994343

[5] M. A. Majewski , M. Stępień , Angew. Chem. Int. Ed. 2019, 58, 86–116; 10.1002/anie.201807004 30006951

[6a] C. Wentrup , Angew. Chem. Int. Ed. 2017, 56, 14808–14835; 10.1002/anie.201705118 28675675

[6b] L. T. Scott , J. Org. Chem. 2016, 81, 11535–11547. 27934463 10.1021/acs.joc.6b02113

[7a] B. D. Steinberg , E. A. Jackson , A. S. Filatov , A. Wakamiya , M. A. Petrukhina , L. T. Scott , J. Am. Chem. Soc. 2009, 131, 10537–10545; 19722628 10.1021/ja9031852

[7b] K. Shoyama , F. Würthner , J. Am. Chem. Soc. 2019, 141, 13008–13012. 31380635 10.1021/jacs.9b06617

[8] K. Shoyama , D. Schmidt , M. Mahl , F. Würthner , Org. Lett. 2017, 19, 5328–5331. 28901772 10.1021/acs.orglett.7b02618

[9a] K. Kawasumi , Q. Zhang , Y. Segawa , L. T. Scott , K. Itami , Nat. Chem. 2013, 5, 739–744; 23965674 10.1038/nchem.1704

[9b] Z. Z. Zhu , Z. C. Chen , Y. R. Yao , C. H. Cui , S. H. Li , X. J. Zhao , Q. Zhang , H. R. Tian , P. Y. Xu , F. F. Xie , X.-M. Xie , Y.-Z. Tan , S.-L. Deng , J. M. Quimby , L. T. Scott , et al., Sci. Adv. 2019, 5, eaaw0982. 31467971 10.1126/sciadv.aaw0982 PMC6707775

[10a] M. Kabdulov , M. Jansen , K. Y. Amsharov , Chem. Eur. J. 2013, 19, 17262–17266; 24273113 10.1002/chem.201303838

[10b] J.-F. Greisch , K. Y. Amsharov , J. Weippert , P. Weis , A. Böttcher , M. M. Kappes , J. Am. Chem. Soc. 2016, 138, 11254–11263; 27501376 10.1021/jacs.6b06205

[10c] S. Ulas , J. Weippert , K. Y. Amsharov , M. Jansen , M. L. Pop , M. V. Diudea , D. Strelnikov , A. Böttcher , M. M. Kappes , J. Phys. Chem. C 2015, 119, 7308–7318;

[10d] K. Y. Amsharov , M. A. Kabdulov , M. Jansen , Chem. Eur. J. 2010, 16, 5868–5871. 20391586 10.1002/chem.201000374

[11a] O. Allemann , S. Duttwyler , P. Romanato , K. K. Baldridge , J. S. Siegel , Science 2011, 332, 574–577; 21527709 10.1126/science.1202432

[11b] S. Duttwyler , C. Douvris , N. L. P. Fackler , F. S. Tham , C. A. Reed , K. K. Baldridge , J. S. Siegel , Angew. Chem. Int. Ed. 2010, 49, 7519–7522; 10.1002/anie.201003762 20818636

[12a] V. Akhmetov , A. Förtsch , M. Feofanov , S. Troyanov , K. Y. Amsharov , Org. Chem. Front. 2020, 7, 1271–1275;

[12b] D. Sharapa , A.-K. Steiner , K. Y. Amsharov , Phys. Status Solidi B 2018, 255, 1800189;

[12c] A.-K. Steiner , K. Y. Amsharov , Angew. Chem. Int. Ed. 2017, 56, 14732–14736; 10.1002/anie.201707272 28857380

[12d] K. Y. Amsharov , M. A. Kabdulov , M. Jansen , Angew. Chem. Int. Ed. 2012, 51, 4594–4597; 10.1002/anie.201200516 22473916

[13a] Q. Tan , S. Higashibayashi , S. Karanjit , H. Sakurai , Nat. Commun. 2012, 3, 891; 22692534 10.1038/ncomms1896

[13b] H. Yokoi , S. Hiroto , D. Sakamaki , S. Seki , H. Shinokubo , Chem. Sci. 2018, 9, 819–824; 29629149 10.1039/c7sc04453d PMC5872494

[13c] H. Yokoi , Y. Hiraoka , S. Hiroto , D. Sakamaki , S. Seki , H. Shinokubo , Nat. Commun. 2015, 6, 8215. 26337912 10.1038/ncomms9215 PMC4569845

[14a] M. Krzeszewski , T. Kodama , E. M. Espinoza , V. I. Vullev , T. Kubo , D. T. Gryko , Chem. Eur. J. 2016, 22, 16478–16488; 27659591 10.1002/chem.201603282

[14b] S. Mishra , M. Krzeszewski , C. A. Pignedoli , P. Ruffieux , R. Fasel , D. T. Gryko , Nat. Commun. 2018, 9, 1714; 29712921 10.1038/s41467-018-04144-5 PMC5928119

[14c] G. Otero , G. Biddau , C. Sánchez-Sánchez , R. Caillard , M. F. López , C. Rogero , F. J. Palomares , N. Cabello , M. A. Basanta , J. Ortega , J. Méndez , A. M. Echavarren , R. Pérez , B. Gómez-Lor , J. A. Martín-Gago , Nature 2008, 454, 865–868. 18704082 10.1038/nature07193

[15a] Y. Inokuma , A. Osuka , Dalton Trans. 2008, 2517–2526; 18443691 10.1039/b719808f

[15b] Y. Saegusa , T. Ishizuka , K. Komamura , S. Shimizu , H. Kotani , N. Kobayashi , T. Kojima , Phys. Chem. Chem. Phys. 2015, 17, 15001–15011; 25986941 10.1039/c5cp01420d

[15c] T. Ishizuka , Y. Saegusa , Y. Shiota , K. Ohtake , K. Yoshizawa , T. Kojima , Chem. Commun. 2013, 49, 5939–5941. 10.1039/c3cc42831a 23715087

[16a] D. Lungerich , J. F. Hitzenberger , M. Marcia , F. Hampel , T. Drewello , N. Jux , Angew. Chem. Int. Ed. 2014, 53, 12231–12235; 10.1002/anie.201407053 25244699

[16b] D. Lungerich , J. F. Hitzenberger , W. Donaubauer , T. Drewello , N. Jux , Chem. Eur. J. 2016, 22, 16755–16759; 27661059 10.1002/chem.201603789

[16c] D. Lungerich , J. F. Hitzenberger , F. Hampel , T. Drewello , N. Jux , Chem. Eur. J. 2018, 24, 15818–15824; 30151869 10.1002/chem.201803684

[16d] M. M. Martin , D. Lungerich , P. Haines , F. Hampel , N. Jux , Angew. Chem. Int. Ed. 2019, 58, 8932–8937; 10.1002/anie.201903654 30968516

[16e] M. M. Martin , D. Lungerich , F. Hampel , J. Langer , T. K. Ronson , N. Jux , Chem. Eur. J. 2019, 25, 15083–15090. 31429504 10.1002/chem.201903113 PMC6899994

[17a] V. V. Diev , C. W. Schlenker , K. Hanson , Q. Zhong , J. D. Zimmerman , S. R. Forrest , M. E. Thompson , J. Org. Chem. 2012, 77, 143–159; 22077105 10.1021/jo201490y

[17b] N. K. S. Davis , A. L. Thompson , H. L. Anderson , J. Am. Chem. Soc. 2011, 133, 30–31. 21141995 10.1021/ja109671f

[18a] Y. He , M. Garnica , F. Bischoff , J. Ducke , M.-L. Bocquet , M. Batzill , W. Auwärter , J. V. Barth , Nat. Chem. 2017, 9, 33–38; 27995925 10.1038/nchem.2600

[18b] W. Perkins , F. R. Fischer , Chem. Eur. J. 2017, 23, 17687–17691; 29108109 10.1002/chem.201705252

[18c] L. M. Mateo , Q. Sun , S.-X. Liu , J. J. Bergkamp , K. Eimre , C. A. Pignedoli , P. Ruffieux , S. Decurtins , G. Bottari , R. Fasel , T. Torres , Angew. Chem. Int. Ed. 2020, 59, 1334–1339; 10.1002/anie.201913024 31729821

[19a] H. M. Jeong , J. W. Lee , W. H. Shin , Y. J. Choi , H. J. Shin , J. K. Kang , J. W. Choi , Nano Lett. 2011, 11, 2472–2477; 21595452 10.1021/nl2009058

[19b] Z. Fei , J. Dong , M. J. Arellano-Jiménez , G. Ye , N. D. Kim , E. L. G. Samuel , Z. Peng , Z. Zhu , F. Qin , J. Bao , M. J. Yacaman , P. M. Ajayan , D. Chen , J. M. Tour , Nat. Commun. 2015, 6, 8668; 26487368 10.1038/ncomms9668 PMC4639894

[19c] C. Zhang , J. Sha , H. Fei , M. Liu , S. Yazdi , J. Zhang , Q. Zhong , X. Zou , N. Zhao , H. Yu , Z. Jiang , E. Ringe , B. I. Yakobson , J. Dong , D. Chen , J. M. Tour , ACS Nano 2017, 11, 6930–6941; 28656759 10.1021/acsnano.7b02148

[19d] X. Wang , X. Li , L. Zhang , Y. Yoon , P. K. Weber , H. Wang , J. Guo , H. Dai , Science 2009, 324, 768–771; 19423822 10.1126/science.1170335

[19e] Z. Wang , J. Zhao , Q. Cai , Phys. Chem. Chem. Phys. 2017, 19, 23113–23121; 28820201 10.1039/c7cp04299j

[19f] S. Wei , A. Li , J.-C. Liu , Z. Li , W. Chen , Y. Gong , Q. Zhang , W.-C. Cheong , Y. Wang , L. Zheng , H. Xiao , C. Chen , D. Wang , Q. Peng , L. Gu , et al., Nat. Nanotechnol. 2018, 13, 856–861. 30013217 10.1038/s41565-018-0197-9

[20a] M. Stępień , E. Gońka , M. Żyła , N. Sprutta , Chem. Rev. 2017, 117, 3479–3716; 27258218 10.1021/acs.chemrev.6b00076

[20b] N. Fukui , W.-Y. Cha , S. Lee , S. Tokuji , D. Kim , H. Yorimitsu , A. Osuka , Angew. Chem. Int. Ed. 2013, 52, 9728–9732; 10.1002/anie.201304794 23913359

[20c] K. Ota , T. Tanaka , A. Osuka , Org. Lett. 2014, 16, 2974–2977; 24818531 10.1021/ol501115m

[20d] J. P. Lewtak , D. T. Gryko , Chem. Commun. 2012, 48, 10069–10086. 10.1039/c2cc31279d 22649792

[21a] M. Grzybowski , B. Sadowski , H. Butenschön , D. Gryko , Angew. Chem. Int. Ed. 2020, 59, 2998–3027; 10.1002/anie.201904934 PMC7027897 31342599

[21b] M. Grzybowski , K. Skonieczny , H. Butenschön , D. T. Gryko , Angew. Chem. Int. Ed. 2013, 52, 9900–9930; 10.1002/anie.201210238 23852649

[22] Taken in parts from: D. Lungerich, Fragments of Graphyrin, Friedrich-Alexander-Universität Erlangen-Nürnberg (FAU), **2017**.

[23a] L. Zhang , M. Lepper , M. Stark , D. Lungerich , N. Jux , W. Hieringer , H.-P. Steinrück , H. Marbach , Phys. Chem. Chem. Phys. 2015, 17, 13066–13073; 25912831 10.1039/c5cp01490e

[23b] M. Lepper , L. Zhang , M. Stark , S. Ditze , D. Lungerich , N. Jux , W. Hieringer , H.-P. Steinrück , H. Marbach , J. Phys. Chem. C 2015, 119, 19897–19905; 10.1039/c5cp01490e 25912831

[23c] L. Zhang , M. Lepper , M. Stark , T. Menzel , D. Lungerich , N. Jux , W. Hieringer , H.-P. Steinrück , H. Marbach , Phys. Chem. Chem. Phys. 2017, 19, 20281–20289. 28726947 10.1039/c7cp03731g

[24] K. S. F. Lau , M. Sadilek , M. Gouterman , G. E. Khalil , C. Brückner , J. Am. Soc. Mass Spectrom. 2006, 17, 1306–1314. 16857382 10.1016/j.jasms.2006.06.004

[25] O. S. Finikova , A. V. Cheprakov , I. P. Beletskaya , P. J. Carroll , S. A. Vinogradov , J. Org. Chem. 2004, 69, 522–535. 14725469 10.1021/jo0350054

[26] B. A. Iglesias , J. F. B. Barata , M. R. M. Domingues , M. G. P. M. S. Neves , J. A. S. Cavaleiro , Int. J. Mass Spectrom. 2014, 363, 1–7.

[27] The integration of signals gives the same result as the summing up of the peak intensities.

[28] M. Rosario , M. Domingues , O. V. Nemirovskiy , M. Graço , O. S. Marques , M. Graça Neves , J. A. S. Cavaleiro , A. J. Ferrer-Correia , M. L. Gross , J. Am. Soc. Mass Spectrom. 1998, 9, 767–774. 9692252 10.1016/s1044-0305(98)00048-8

[29] D. J. Douglas , J. Phys. Chem. 1982, 86, 185–191.

[30] E. Mishra , J. L. Worlinsky , C. Brückner , V. Ryzhov , J. Am. Soc. Mass Spectrom. 2014, 25, 18–29. 24135805 10.1007/s13361-013-0750-6

[31a] Y. Minenkov , Å. Singstad , G. Occhipinti , V. R. Jensen , Dalton Trans. 2012, 41, 5526–5541; 22430848 10.1039/c2dt12232d

[31b] Y. Shao , L. F. Molnar , Y. Jung , J. Kussmann , C. Ochsenfeld , S. T. Brown , A. T. B. Gilbert , L. V. Slipchenko , S. V. Levchenko , D. P. O'Neill , R. A. DiStasio, Jr. , R. C. Lochan , T. Wang , G. J. O. Beran , N. A. Besley , et al., Phys. Chem. Chem. Phys. 2006, 8, 3172–3191. 16902710 10.1039/b517914a

[32a] W. Jentzen , X.-Z. Song , J. A. Shelnutt , J. Phys. Chem. B 1997, 101, 1684–1699;

[32b] W. Jentzen , J.-G. Ma , J. A. Shelnutt , Biophys. J. 1998, 74, 753–763; 9533688 10.1016/S0006-3495(98)74000-7 PMC1302556

[32c] J. A. Shelnutt , J. Porphyrins Phthalocyanines 2001, 5, 300–311.

[33] M. Ruppel , D. Lungerich , S. Sturm , R. Lippert , F. Hampel , N. Jux , Chem. Eur. J. 2020, 26, 3287–3296. 31846109 10.1002/chem.201904718 PMC7154557

[34] R. W. Kirschbaum , M. Hausmann , O. V. Boltalina , S. H. Strauss , T. Drewello , Phys. Chem. Chem. Phys. 2015, 17, 23052–23058. 26272592 10.1039/c5cp03112e

[35] S. J. Blanksby , G. B. Ellison , Acc. Chem. Res. 2003, 36, 255–263. 12693923 10.1021/ar020230d

[36] B. Ruscic , R. E. Pinzon , M. L. Morton , G. Von Laszevski , S. J. Bittner , S. G. Nijsure , K. A. Amin , M. Minkoff , A. F. Wagner , J. Phys. Chem. A 2004, 108, 9979–9997.

[37a] L. Cronin , C. L. Higgitt , R. Karch , R. N. Perutz , Organometallics 1997, 16, 4920–4928;

[37b] M. Ohashi , M. Shibata , H. Saijo , T. Kambara , S. Ogoshi , Organometallics 2013, 32, 3631–3639;

[37c] A. D. Sun , K. Leung , A. D. Restivo , N. A. LaBerge , H. Takasaki , J. A. Love , Chem. Eur. J. 2014, 20, 3162–3168. 24522982 10.1002/chem.201303809

[38a] E. A. Jackson , B. D. Steinberg , M. Bancu , A. Wakamiya , L. T. Scott , J. Am. Chem. Soc. 2007, 129, 484–485. 17226999 10.1021/ja067487h

[39a] L. Zhao , R. I. Kaiser , B. Xu , U. Ablikim , W. Lu , M. Ahmed , M. M. Evseev , E. K. Bashkirov , V. N. Azyazov , M. V. Zagidullin , A. N. Morozov , A. H. Howlader , S. F. Wnuk , A. M. Mebel , D. Joshi , Nat. Commun. 2019, 10, 1510; 30944302 10.1038/s41467-019-09224-8 PMC6447558

[39b] L. Zhao , B. Xu , U. Ablikim , W. Lu , M. Ahmed , M. M. Evseev , E. K. Bashkirov , V. N. Azyazov , A. H. Howlader , S. F. Wnuk , A. N. Morozov , A. H. Howlader , S. F. Wnuk , A. M. Mebel , D. Joshi , ChemPhysChem 2019, 20, 791–797; 30710434 10.1002/cphc.201801154

[39c] A. M. Thomas , L. Zhao , C. He , G. R. Galimova , A. M. Mebel , R. I. Kaiser , Angew. Chem. Int. Ed. 2019, 58, 15488–15495; 10.1002/anie.201908039 31368202

