# Supplementary material for: Gas‐Phase Transformation of Fluorinated Benzoporphyrins to Porphyrin‐Embedded Conical Nanocarbons
Source: Chemistry. 2020 Sep 2;26(53):12180–7. doi: 10.1002/chem.202002638 (PMC7540561; doi:10.1002/chem.202002638)
Supplement: Supplementary file 1 — Supplementary [file CHEM-26-12180-s001.pdf]

Chemistry–A European Journal

Supporting Information

Gas-Phase Transformation of Fluorinated Benzoporphyrins to Porphyrin-Embedded Conical Nanocarbons

Dominik Lungerich,*[a, b, c] Jakob Felix Hitzenberger,[d] Michael Ruppel,[a] Tibor Döpper,[e] Matthias Witt,[f] Ivana Ivanović-Burmazović,[g] Andreas Görling,[e] Norbert Jux,*[a] and Thomas Drewello*[d]

Author Contributions

D.L. Data curation: Lead; Formal analysis: Lead; Investigation: Equal; Methodology: Supporting; Project administration: Equal; Visualization: Lead; Writing - Original Draft: Lead; Writing - Review & Editing: Lead; Synthesis, Computations: Lead
J.H. Conceptualization: Supporting; Data curation: Equal; Formal analysis: Equal; Methodology: Equal; Visualization: Supporting; Writing - Original Draft: Supporting; Mass spectrometry: Lead
M.R. Conceptualization: Supporting; Data curation: Supporting; Formal analysis: Supporting; Investigation: Supporting; Visualization: Supporting; Writing - Original Draft: Supporting; Writing - Review & Editing: Supporting
T.D. Investigation: Supporting; Computation: Supporting
M.W. Writing - Review & Editing: Supporting; Mass Spectrometry: Supporting
I.I. Resources: Equal
A.G. Funding acquisition: Equal; Resources: Supporting
N.J. Conceptualization: Supporting; Funding acquisition: Equal; Resources: Equal; Supervision: Equal; Writing - Review & Editing: Supporting
T.D. Conceptualization: Equal; Formal analysis: Supporting; Funding acquisition: Lead; Project administration: Equal; Supervision: Equal; Writing - Review & Editing: Equal.

TABLE OF CONTENT

1. SYNTHETIC SECTION S2

1.1 General information S2

1.2 Synthesis of molecules S3

1.3 Chapter S1 – wet-chemical approaches to graphyrin S37

Wet-chemical palladium-catalyzed C–H activation S37

Wet-chemical intramolecular oxidative cyclodehydrogenation S41

2. MASS SPECTROMETRIC SECTION S43

2.1 General information S43

2.2 Chapter S2 – Dissociation Experiments of 8 and 8Pd S44

QTOF experiments with 8 S44

FT-ICR experiments with 8 S45

FT-ICR experiments with 8Pd S50

2.3 Chapter S3 – Dissociation Experiments of 1, 1Co, 1Cu, 1Zn, 2, 7, and Ref S51

FT-ICR experiments with 2 S52

QTOF experiments with 1 S54

FT-ICR experiments with 1 S56

QTOF experiments with 1Co S58

FT-ICR experiments with 1Co S59

QTOF and FT-ICR experiments with 1Cu and 1Zn S61

QTOF Experiments with 7 **S63**

FT-ICR experiments with Ref (5,10,15,20-tetrakis(pentafluorophenyl)-porphyrin) S65

2.4 Chapter S4 – Dissociation Experiments of 3, 4, 5, 6, and 6Pd S66

QTOF experiments with 3 S66

FT-ICR experiments with 3 S66

QTOF experiments with 4 S67

FT-ICR experiments with 4 S67

QTOF experiments with 5 **S68**

QTOF experiments with 6 **S68**

QTOF experiments with 6Pd S69

3. COMPUTATIONAL ANALYSIS S70

3.1 General information S70

3.2 Energy data on cyclization of $[1+H]^+$ and $[1Co]^{+\bullet}$ S71

3.3 Energy data on cyclization of $[1+H]^+$ and $[1Cu]^{+\bullet}$ S72

3.4 Normal-coordinate structural decomposition analysis S74

3.5 XYZ Coordinates of geometry optimized structures S83

4. REFERENCES S108

1. SYNTHETIC SECTION

1.1 General information

All chemicals were purchased from Sigma-Aldrich and used without any further purification. Solvents were distilled prior to usage. Dichloromethane and chloroform were neutralized with K_2CO_3 before distillation. Microwave–assisted reactions were carried out in the respective vials of a Biotage initiator$^+$ monomode microwave reactor. Standard stir-rate was 600 rpm, fixed hold time (FHT) was on and no external cooling was applied. Thin layer chromatography (TLC) was performed on Merck silica gel 60 F524, detected by UV-light (254nm, 366nm). Column chromatography was performed on Macherey-Nagel silica gel 60 M (230-400 mesh, 0.04–0.063 mm). NMR spectroscopy was performed on a Bruker Avance Neo CryoProbe DCH (^{1}H: 600 MHz, ^{13}C: 150 MHz), Bruker Avance 400 (^{1}H: 400 MHz, ^{13}C: 100 MHz), Bruker Avance 300 (^{1}H: 300 MHz, ^{13}C: 75 MHz) or Jeol EX400 (^{1}H: 400 MHz, ^{13}C: 100 MHz). Deuterated solvents were purchased from Sigma Aldrich and used as received. ^{1}H NMR and ^{13}C NMR chemical shifts δ are given in parts per million [ppm] and are referenced to residual protic impurities in the solvent (^{1}H NMR), or to the deuterated solvent itself (^{13}C NMR). The resonance multiplicities are indicated as “s“ (singlet), “d” (doublet), “t” (triplet), “q” (quartet) and “m” (multiplet). Signals referred to as “bs” (broad singlet) are not clearly resolved or significantly broadened. LDI/MALDI-ToF (nitrogen UV-laser, 337 nm) mass spectra were obtained by using a Bruker ultrafleXreme spectrometer with 2,5-dihydroxybenzoic acid (DHB) or (*E*)-2-(3-(4-(*tert*-butyl)phenyl)-2-methylallylidene)malononitrile (dctb) as matrices. ESI/APPI-ToF mass spectrometry was carried out on a Bruker maXis 4G UHR TOF MS/MS-spectromter or a Bruker micrOTOF II focus TOF MS-spectrometer. IR spectroscopy was performed on a Bruker FT-IR Tensor 27 and Pike MIRacle ATR unit. The ATR unit was equipped with a diamond crystal plate and high-pressure clamp. Spectra were recorded as solid samples directly from the diamond crystal. All absorptions $\tilde{\nu}$ are given in wave numbers [cm^{-1}]. UV/vis spectroscopy was carried out on a Varian Cary 5000 UV-Vis-NIR spectrometer. Spectra were recorded at room temperature using quartz cuvettes with a path length of 1 cm. Fluorescence spectra were recorded on a Shimadzu RF–5301PC spectrofluorophotometer.

1.2 Synthesis of molecules

General Procedure to free-base derivatives

Herein discussed molecules are published and characterized structurally, spectroscopically and electrochemically in detail in our recent publication (M. Ruppel et al. *Chem. Eur. J.* **2020**, *26*, 3287–3296). They were synthesized according to the reported literature procedure and spectroscopic data were in agreement with reported values.[1]

General information on metalated derivatives

Metalated derivatives were used for MS analysis without further spectroscopic characterization. Fluorinated benzoporphyrins show very poor solubility in common organic solvents, in particular if they are present as metalated species. However, whereas free-base species can be easily solubilized by addition of trifluoroacetic acid, this strategy does not apply for metalated derivatives.[1]

***meso*-Tetrakis(2,3,4,5,6-pentafluorophenyl)tetrabenzoporphyrin 1**

Purified by filtration over silica gel (CH_2Cl_2 + 1% TFA) and subsequent recrystallization from CH_2Cl_2/NEt_3 with MeOH, 706 mg, 57%. **^{1}H NMR** (400 MHz, $CDCl_3$/TFA-d_1, rt) δ [ppm] 7.76–7.74 (m, 16H). **HRMS** (APPI, toluene) m/z calcd for $C_{60}H_{18}F_{20}N_4$ $[M]^+$ 1174.1207, found 1174.1218.

***meso*-Tetrakis(2,3,4,5,6-pentafluorophenyl)tetrabenzoporphyrins-Co$^{II/III}$ 1Co**

A 100 mL Schlenk-RBF equipped with a magnetic stir bar and a condenser was charged with *meso*-tetrakis(2,3,4,5,6-pentafluorophenyl)tetrabenzoporphyrin **3** (20 mg, 17 µmol), $Co(OAc)_2 \cdot 4H_2O$ (50 mg, 200 µmol), and dissolved in 1:1 CH_2Cl_2/DMF (80 mL) under N_2 atmosphere. The mixture was heated at reflux for 4 h in darkness, until all starting material was consumed by TLC. After cooling to rt, the CH_2Cl_2 was removed in vacuo and the remaining mixture diluted with H_2O (50 mL). The remaining product was filtered and dried in vacuo, giving a crystalline dark blue solid (19 mg). *Further purification was carried out in-situ in the mass spectrometer by selective ion filtration in the quadrupole. NMR characterization was neglected because of the*

paramagnetic character of Co^{III}-porphyrinoids combined with its poor solubility. **HRMS** (APPI, toluene) m/z calcd for $C_{60}H_{16}CoF_{20}N_4$ $[M]^+$ 1231.03821, found 1231.03929.

meso-Tetrakis(2,3,4,5,6-pentafluorophenyl)tetrabenzoporphyrins-Cu^{II} 1Cu

A 20 mL Schlenk-tube equipped with a magnetic stir bar was charged with the *meso*-tetrakis(2,3,4,5,6-pentafluorophenyl)tetrabenzoporphyrin **3** (20 mg, 17 µmol) and dissolved in CH_2Cl_2 (15 mL) and NEt_3 (3 drops). Then, a saturated solution of $Cu(OAc)_2$ in MeOH (2.5 mL) was added and the mixture was stirred at rt for 18 h in darkness. The mixture was concentrated and the residue was purified by plug filtration (SiO_2, 4x8 cm; CH_2Cl_2). The bright green fraction was concentrated and the product was reprecipitated from CH_2Cl_2 and MeOH, giving a dark green/blue solid (17 mg). *Further purification was carried out in-situ in the mass spectrometer by selective ion filtration in the quadrupole. NMR characterization was neglected because of the paramagnetic character of Cu^{II}-porphyrinoids combined with its poor solubility.* **HRMS** (APPI, toluene) m/z calcd for $C_{60}H_{16}CuF_{20}N_4$ $[M]^+$ 1235.03461, found 1231.03587.

meso-Tetrakis(2,3,4,5,6-pentafluorophenyl)tetrabenzoporphyrins-Zn^{II} 1Zn

A 100 mL Schlenk-RBF equipped with a magnetic stir bar and a condenser was charged with the *meso*-tetrakis(2,3,4,5,6-pentafluorophenyl)tetrabenzoporphyrin **3** (20 mg, 17 µmol), $Zn(OAc)_2 \cdot 2H_2O$ (50 mg, 228 µmol) and dissolved in THF (50 mL) under N_2 atmosphere. The mixture was heated at reflux for 18 h in darkness. After cooling to rt, the mixture was diluted with H_2O (50 mL), extracted with CH_2Cl_2 and dried over $MgSO_4$. The bright green organic layer was concentrated and the product was re-precipitated from CH_2Cl_2 and MeOH, giving a dark green solid (20 mg). *Further purification was dispensed, because Zn-TATBPs are prone to demetallation in even 1% HCl. Therefore, purification was carried out in-situ in the mass spectrometer by selective ion filtration in the quadrupole. NMR characterization was neglected because of the poor solubility and instant demetallation upon addition of solubilizing TFA.* **HRMS** (APPI, toluene) m/z calcd for $C_{60}H_{16}F_{20}N_4Zn$ $[M]^+$ 1236.03416, found 1236.03640.

meso-Tetrakis(2,6-difluorophenyl)tetrabenzoporphyrins 2

Purified by filtration over silica gel (CH_2Cl_2 + 1% TFA) and subsequent recrystallization from CH_2Cl_2/NEt_3 with MeOH, 588 mg, 58%. **^{1}H NMR** (600 MHz, $CDCl_3$/TFA-d_1, rt) δ [ppm] 8.01–7.97 (m, 4H), 7.71–7.69 (AA'BB', 8H), 7.61–7.60 (AA'BB', 8H), 7.51–7.49 (m, 8H). **HRMS** (APPI, THF) m/z calcd for $C_{60}H_{31}F_8N_4$ $[M+H]^+$ 959.2415, found 959.2412.

meso-Tetrakis(3,5-difluorophenyl)tetrabenzoporphyrins 3

Purified by filtration over silica gel (CH_2Cl_2 + 1% TFA) and subsequent recrystallization from CH_2Cl_2/NEt_3 with MeOH, 708 mg, 70%. **^{1}H NMR** (600 MHz, $CDCl_3$/TFA-d_1, rt) δ [ppm] 8.09–8.08 (m, 8H), 7.60–7.58 (AA'BB', 8H), 7.56–7.54 (AA'BB', 8H), 7.50 (tt, $^3J_{HF}$= 8.4 Hz, $^4J_{HH}$= 2.2 Hz, 4H). **HRMS** (APPI, toluene) m/z calcd for $C_{60}H_{30}F_8N_4$ $[M]^+$ 958.2337, found 958.2325.

meso-Tetrakis(3,4,5-trifluorophenyl)tetrabenzoporphyrin 4

Purified by filtration over silica gel (CH_2Cl_2 + 1% TFA) and subsequent recrystallization from CH_2Cl_2/NEt_3 with MeOH, 642 mg, 59%. **^{1}H NMR** (600 MHz, $CDCl_3$/TFA-d_1, rt) δ [ppm] 8.19 (t, $^3J_{HF}$= 6.2 Hz, 8 H), 7.65–7.63 (AA'BB', 8H), 7.55–7.53 (AA'BB', 8H). **HRMS** (APPI, toluene) m/z calcd for $C_{60}H_{26}F_{12}N_4$ $[M]^+$ 1030.1960, found 1030.1951

meso-Tetrakis(4-fluorophenyl)tetrabenzoporphyrins 5

Purified by filtration over silica gel (CH_2Cl_2 + 1% TFA) and subsequent recrystallization from CH_2Cl_2/NEt_3 with MeOH, 805 mg, 86%. **^{1}H NMR** (600 MHz, $CDCl_3$/TFA-d_1, rt) δ [ppm] 8.50–8.48 (m, 8H), 7.66–7.63 (m, 8H), 7.52–7.49 (AA'BB', 8H), 7.48–7.46 (AA'BB', 8H). **HRMS** (APPI, MeCN/toluene) m/z calcd for $C_{60}H_{34}F_4N_4$ $[M]^+$ 886.2714, found 886.2703.

meso-Tetrakis(2-fluorophenyl)tetrabenzoporphyrins 6

Purified by filtration over silica gel (CH_2Cl_2 + 1% TFA) and subsequent recrystallization from CH_2Cl_2/NEt_3 with MeOH, 749 mg, 80% (*mixture of atropisomers*). **^{1}H NMR** (400

MHz, CD_2Cl_2/TFA-d_1, rt) δ [ppm] 8.59–8.45 (m, 4H), 8.10–8.04 (m, 4H), 7.82–7.76 (m, 4H), 7.72–7.66 (m, 4H), 7.62–7.55 (m, 16H). **HRMS** (ESI, MeCN/toluene) m/z calcd for $C_{60}H_{35}F_4N_4$ $[M+H]^+$ 887.2792, found 887.2776.

meso-Tetrakis(2-fluorophenyl)tetrabenzoporphyrin-Pd^{II} 6Pd

A 50 mL Schlenk-RBF equipped with a magnetic stir bar and a condenser was charged with 15 equiv. $PdCl_2$ (9 mg, 507 µmol) in PhCN (10 mL) and heated to 180 °C under N_2 atmosphere. After all inorganics were dissolved, *meso*-tetrakis(2-fluorophenyl)-tetrabenzoporphyrin **8** (30 mg, 34 µmol) was added and the mixture was stirred at 180 °C for 1 h. The solvent was removed in vacuo and the residue was purified by plug filtration (SiO_2, 3x8 cm; CH_2Cl_2). The bright green fraction was concentrated and the product was re-precipitated from CH_2Cl_2 and MeOH. *NMR characterization was neglected because of the poor solubility in common solvents.* **HRMS** (ESI, CH_2Cl_2/MeCN) m/z calcd for $C_{60}H_{28}Cl_8N_4Pd$ $[M]^+$ 990.16122, found 990.15871.

meso-Tetrakis(4-bromo-2,6-difluorophenyl)tetrabenzoporphyrin 7

Purified by filtration over silica gel (CH_2Cl_2 + 1% TFA) and subsequent recrystallization from CH_2Cl_2/NEt_3 with MeOH, 578 mg, 43%. **^{1}H NMR** (600 MHz, CD_2Cl_2/TFA-d_1, rt) δ [ppm] 7.81–7.80 (AA'BB', 8H), 7.78 (d, $^3J_{HF}$= 6.7 Hz, 8H), 7.73–7.71 (AA'BB', 8H). **^{13}C{^{1}H} NMR** (150 MHz, CD_2Cl_2/TFA-d_1, rt) δ [ppm] 162.11 (dd, $^1J_{CF}$= 257.2 Hz, $^3J_{CF}$= 5.8 Hz), 141.5, 141.3, 131.7, 130.7, 127.5 (t, $^3J_{CF}$= 12.1 Hz), 123.4, 117.9 (dd, $^2J_{CF}$= 24.2 Hz, $^4J_{CF}$= 3.2 Hz), 115.4 (t, $^2J_{CF}$= 17.8 Hz), 99.1, 99.0. **^{19}F{^{1}H} NMR** (470 MHz, CD_2Cl_2/TFA-d_1, rt) δ [ppm] -109.6. **HRMS** (ESI, CH_2Cl_2/MeCN) m/z calcd for $C_{60}H_{27}Br_4F_8N_4$ $[M+H]^+$ 1270.8836, found 1270.8831.

meso-Tetrakis(2,6-dichlorophenyl)tetrabenzoporphyrin 8

Purified by filtration over silica gel (CH_2Cl_2 + 1% TFA) and subsequent recrystallization from CH_2Cl_2/NEt_3 with MeOH, 654 mg, 57%. **^{1}H NMR** (400 MHz, $CDCl_3$/TFA-d_1, rt) δ [ppm] 7.94–7.86 (m, 12H), 7.62–7.59 (AA'BB', 8H), 7.54–7.50 (AA'BB', 8H). **HRMS** (APPI, toluene) m/z calcd for $C_{60}H_{30}Cl_8N_4$ $[M]^+$ 1085.9973, found 1085.9972

***meso*-Tetrakis(2,6-dichlorophenyl)tetrabenzoporphyrin-Pd^{II} 8Pd**

A 50 mL Schlenk-RBF equipped with a magnetic stir bar and a condenser was charged with 15 equiv. $PdCl_2$ (12 mg, 690 µmol) in PhCN (10 mL) and heated to 180 °C under N_2 atmosphere. After all inorganics were dissolved, *meso*-tetrakis(2,6-dichlorophenyl)-tetrabenzoporphyrin **1** (50 mg, 46 µmol) was added and the mixture was stirred at 180 °C for 1.5 h. The solvent was removed in vacuo and the residue was purified by plug filtration (SiO_2, 3x8 cm; CH_2Cl_2). The bright green fraction was concentrated and the product was re-precipitated from CH_2Cl_2 and MeOH. *NMR characterization was neglected because of the poor solubility in common solvents.* **HRMS** (ESI, CH_2Cl_2/MeCN) m/z calcd for $C_{60}H_{28}Cl_8N_4Pd$ $[M]^+$ 1189.88570, found 1089.99149.

***meso*-Tetrakis(2,6-dibromophenyl)tetrabenzoporphyrin S1**

Purified by filtration over silica gel (CH_2Cl_2 + 1% TFA) and subsequent recrystallization from CH_2Cl_2/NEt_3 with MeOH, 664 mg, 44%. **^{1}H NMR** (600 MHz, CD_2Cl_2/TFA-d_1, rt) δ [ppm] 8.20 (d, $^3J_{HH}$= 8.1 Hz, 8H), 7.76 (t, $^3J_{HH}$= 8.1 Hz, 4H), 7.69–7.67 (AA'BB', 8H), 7.60–7.58 (AA'BB', 8H). **HRMS** (ESI) m/z calcd for $C_{60}H_{31}Br_8N_4$ $[M+H]^+$ 1438.6010, found 1438.6022.

***meso*-Tetrakis(3,4,5-trimethoxyphenyl)tetrabenzoporphyrin S2**

Purified by filtration over silica gel (CH_2Cl_2/THF, 1:2, v:v) and subsequent recrystallization from CH_2Cl_2/NEt_3 with MeOH, 702 mg, 56%. **^{1}H NMR** (400 MHz, acetone-d_6/TFA-d_1, rt) δ [ppm] 8.11 (s, 8H), 7.71–7.66 (AA'BB', 8H), 7.57–7.54 (AA'BB', 8H), 4.11 (s, 12H), 3.94 (s, 24H). **HRMS** (APPI, toluene/CH_2Cl_2) m/z calcd for $C_{72}H_{62}N_4O_{12}$ $[M]^+$ 1174.4359, found 1174.4363.

***meso*-Tetraphenyltetrabenzoporphyrin S3**

Purified by filtration over silica gel (CH_2Cl_2) and subsequent recrystallization from CH_2Cl_2/NEt_3 with MeOH, 645 mg, 75%. **^{1}H NMR** (400 MHz, CD_2Cl_2, rt) δ [ppm] 8.36–8.33 (m, 8H), 7.99–7.88 (m, 12H), 7.26 (br, 16H), -1.21 (br, 2H). **HRMS** (APPI, toluene) m/z calcd for $C_{60}H_{39}N_4$ $[M+H]^+$ 815.3169, found 815.3184.

DL10 (D. Lungerich) in C2/c

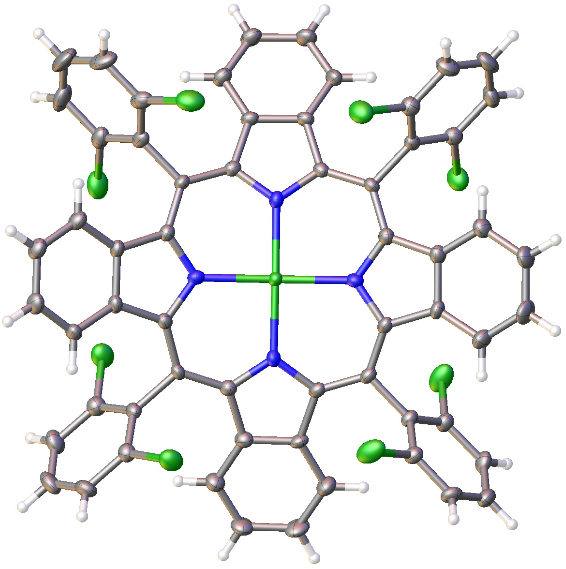

Figure S1. Single crystal X-ray structure of **8Pd**. Thermal ellipsoids are drawn at 50% probability level; CCDC: 1950258. For more information, refer to: Ruppel et al. *Chem. Eur. J.* **2020**, *26*, 3287–3296.

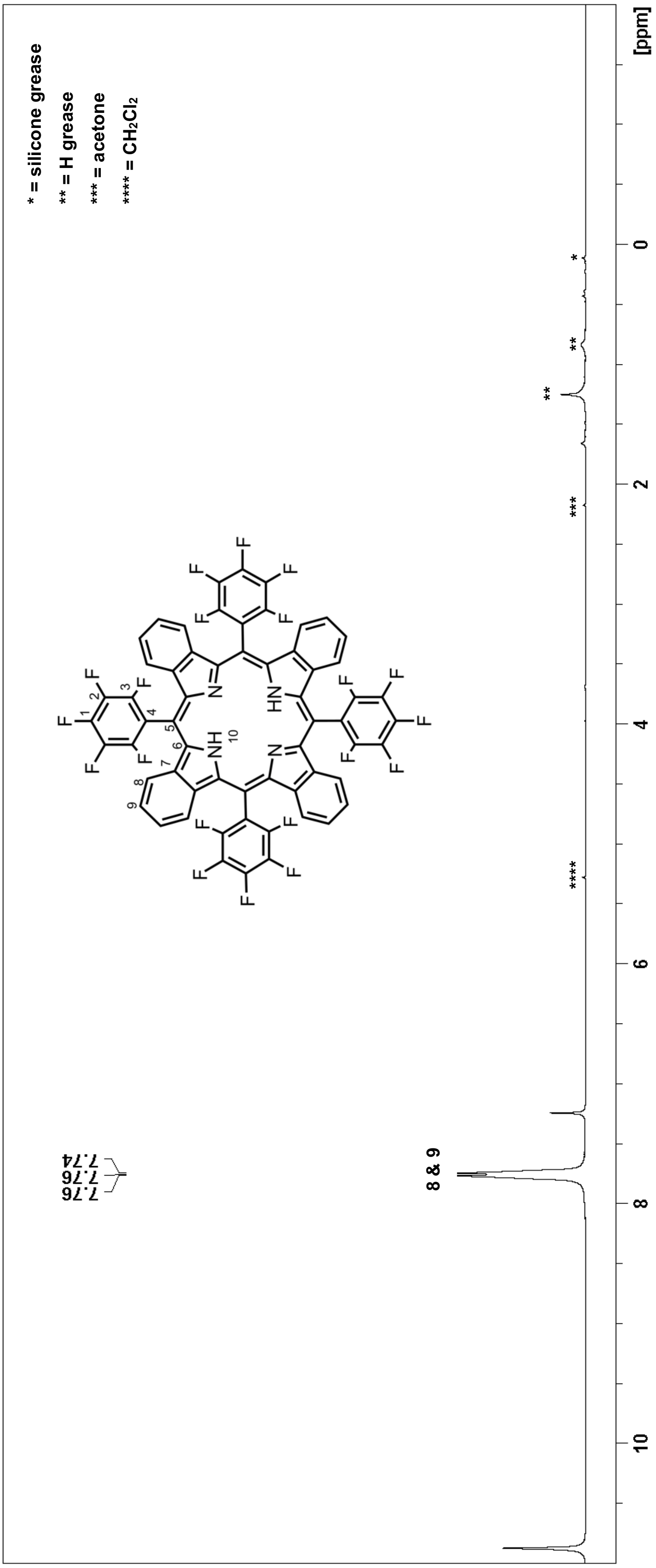

Figure S2. ^{1}H NMR (400 MHz, $CDCl_3$/TFA-d_1, rt) of **1**.

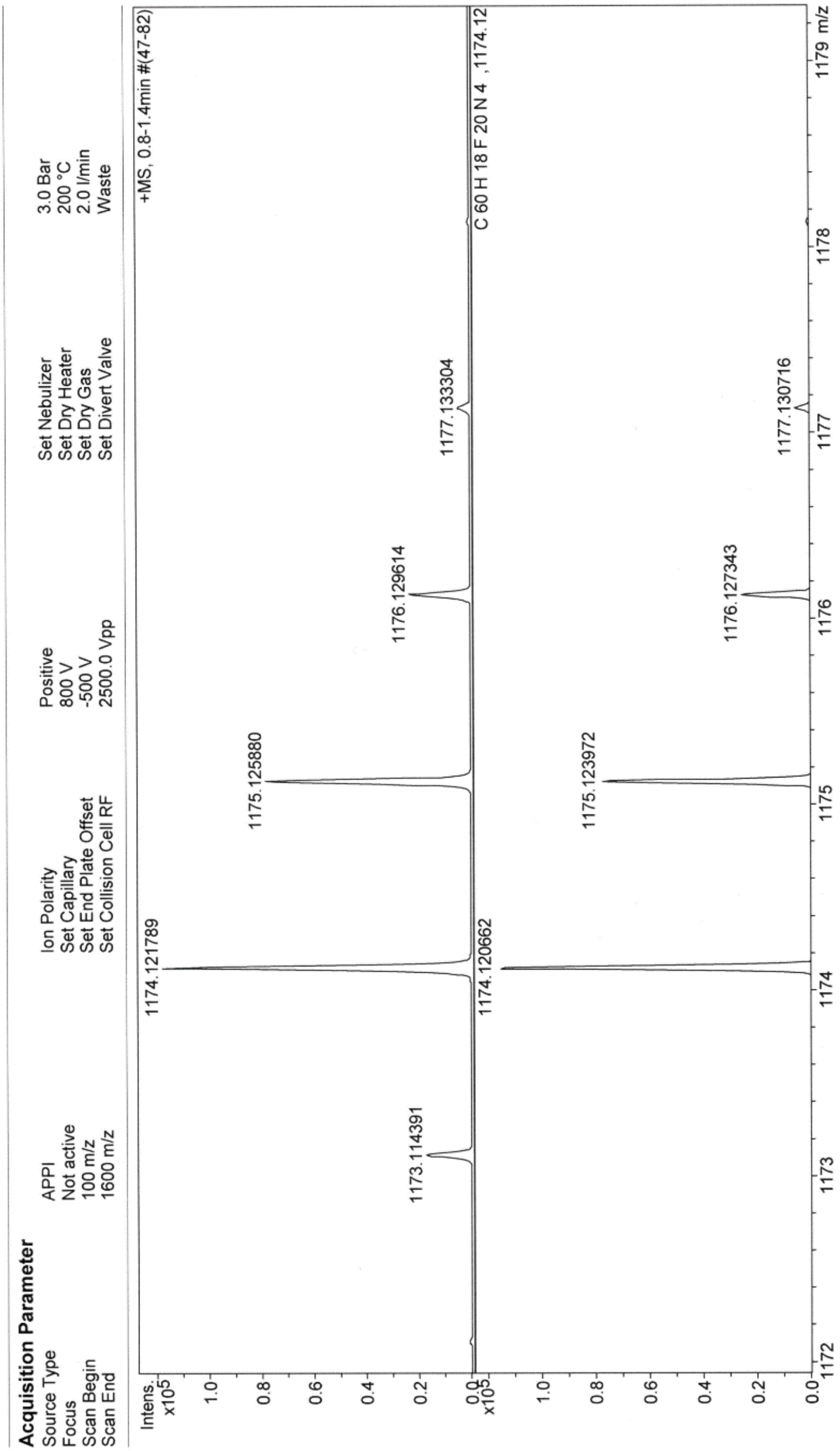

Figure S3. HRMS (APPI, toluene) of **1**.

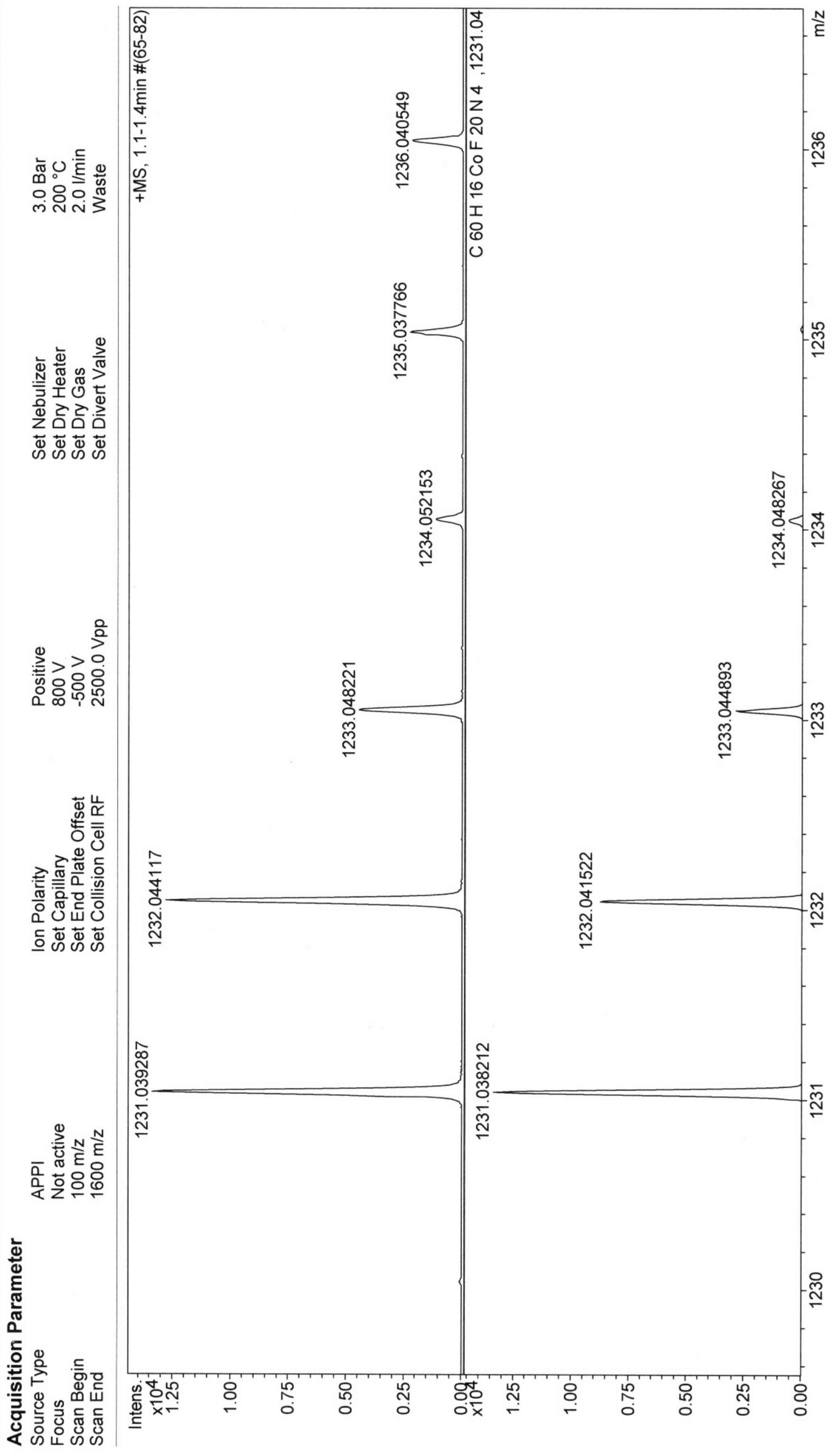

Figure S4. HRMS (APPI, toluene) of **1Co**.

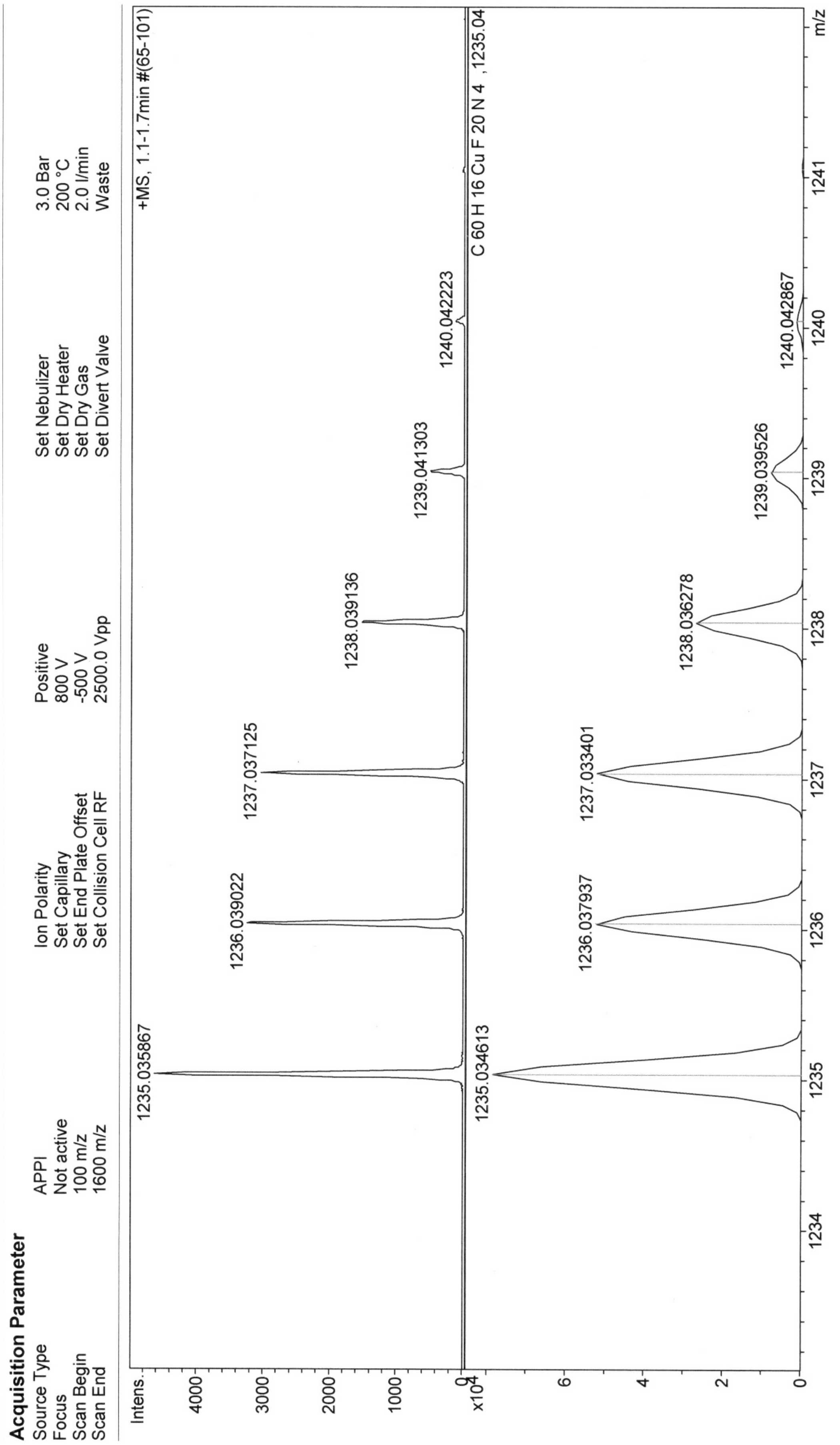

Figure S5. HRMS (APPI, toluene) of **1Cu**.

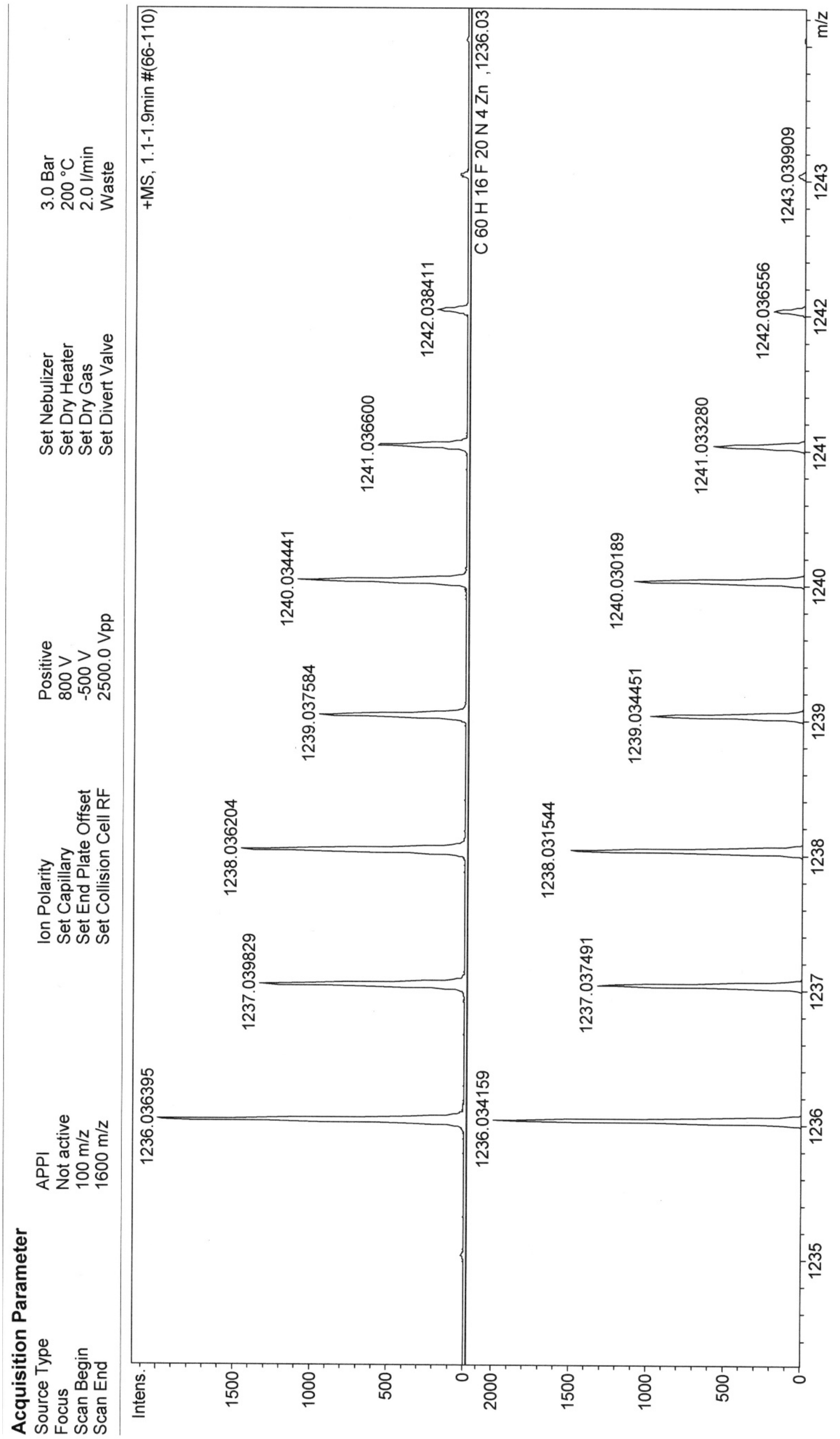

Figure S6. HRMS (APPI, toluene) of **1Zn**.

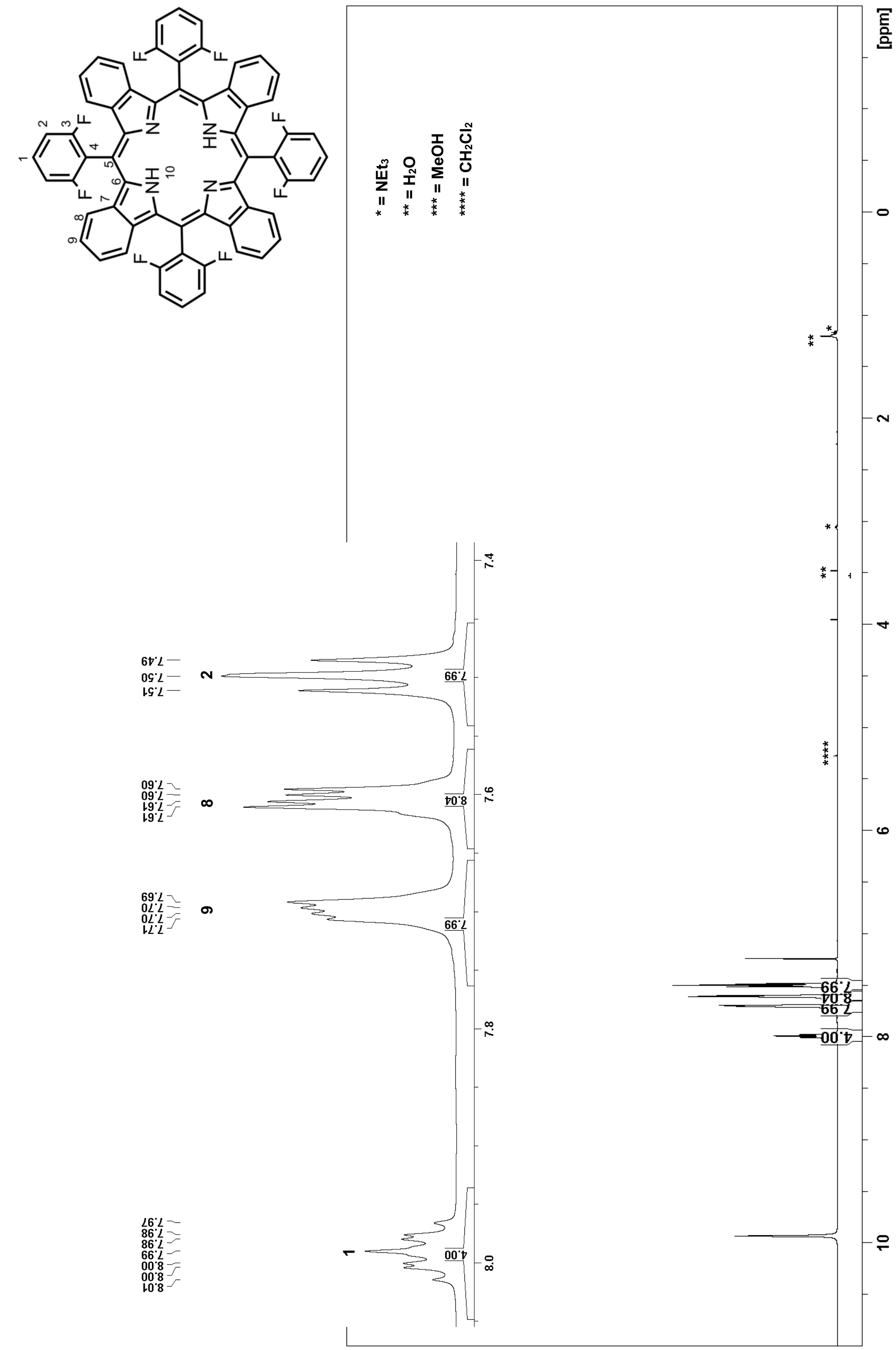

Figure S7. ^{1}H NMR (600 MHz, $CDCl_3$/TFA-d_1, rt) of **2**.

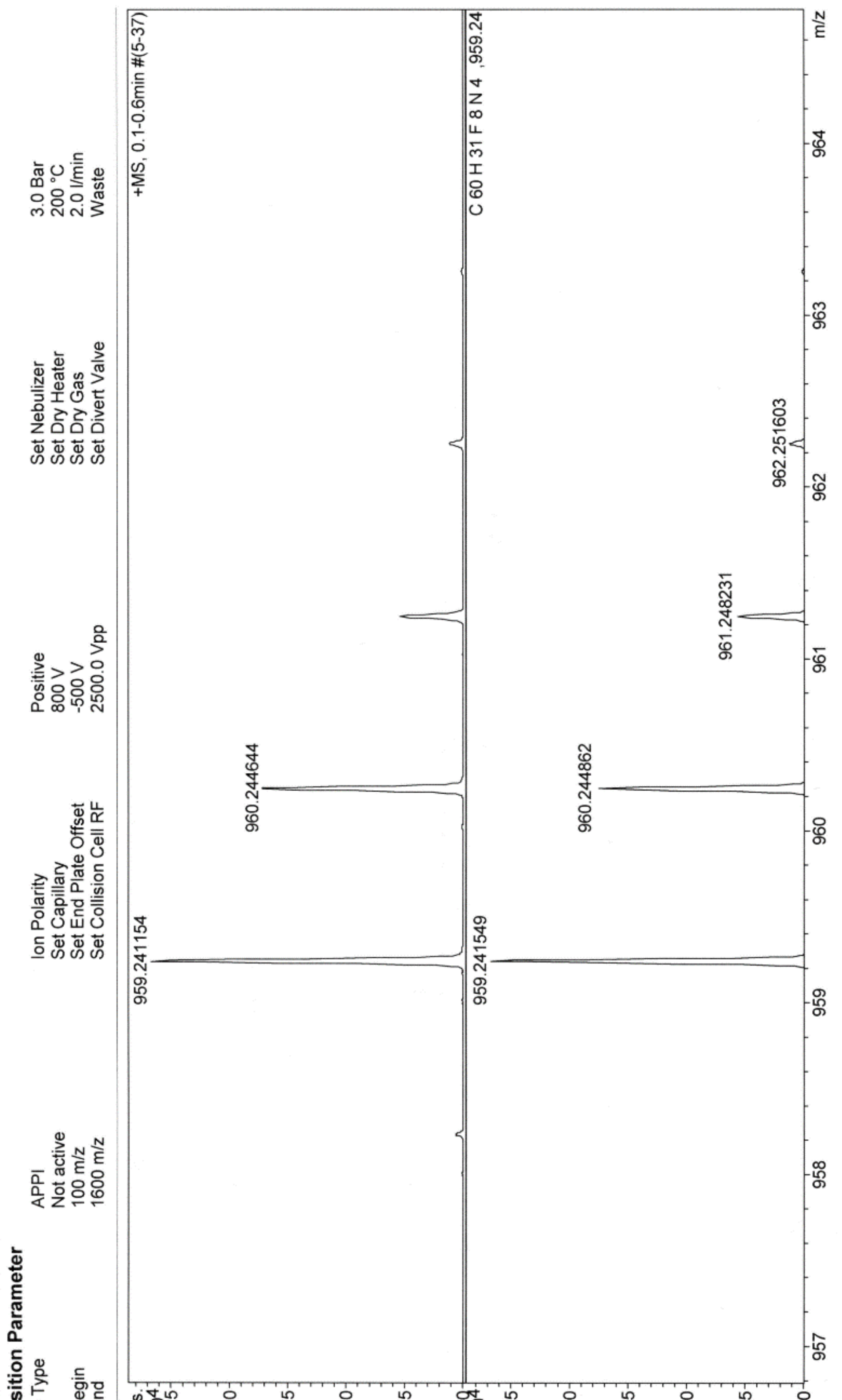

Figure S8. HRMS (APPI, THF) of **2**.

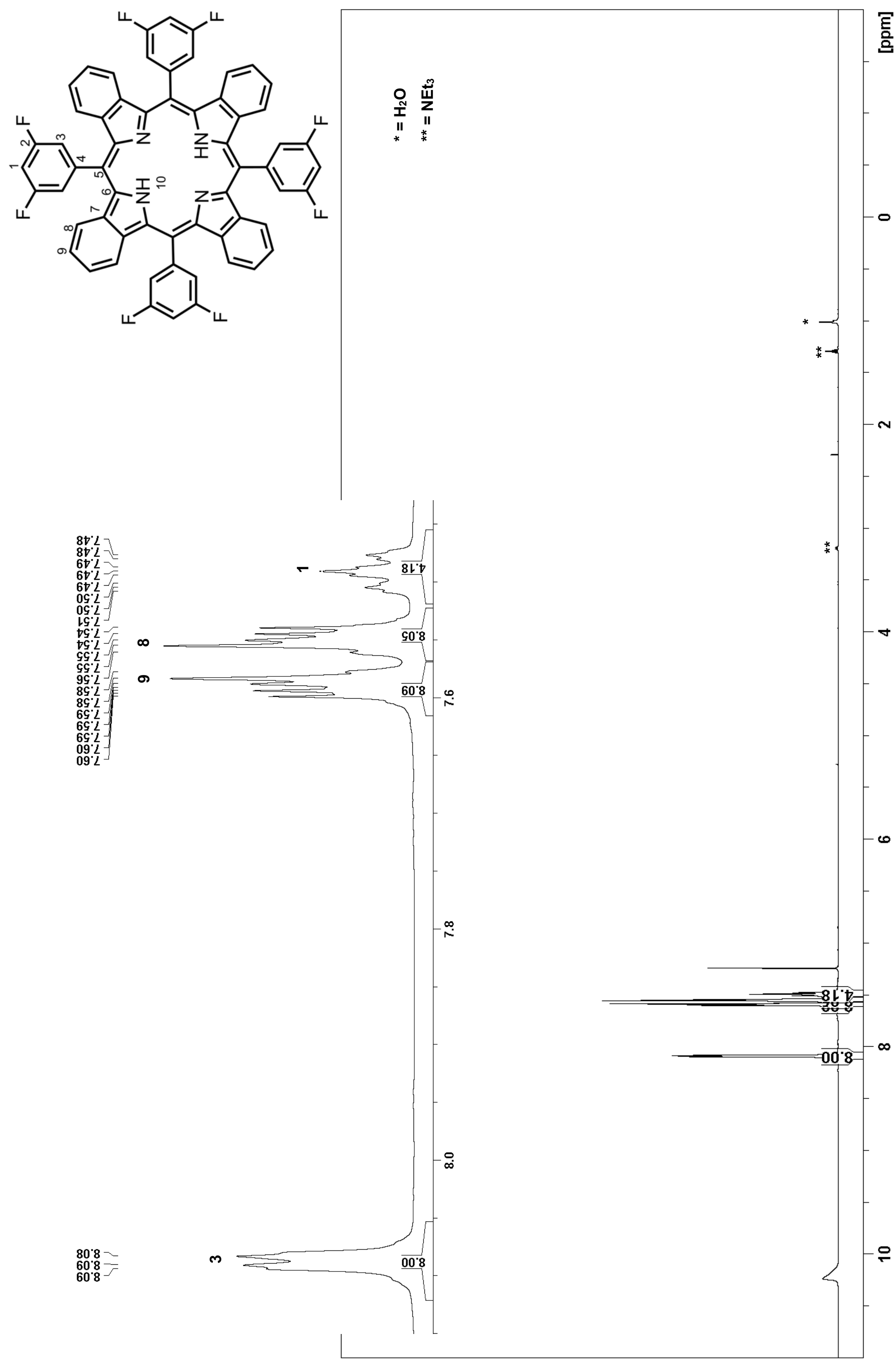

Figure S9. ^{1}H NMR (600 MHz, $CDCl_3$/TFA-d_1, rt) of **3**.

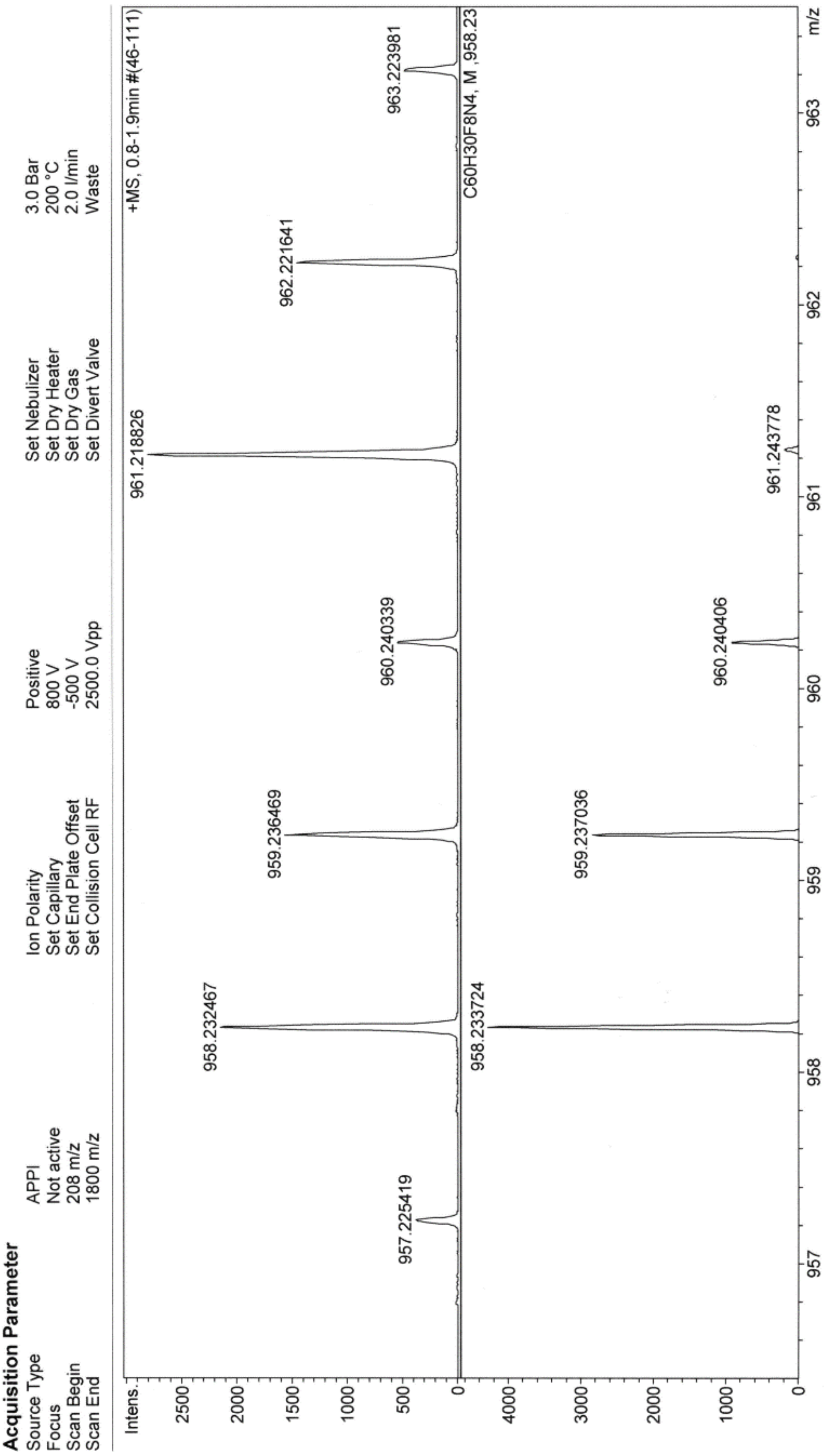

Figure S10. HRMS (APPI, toluene) of **3**.

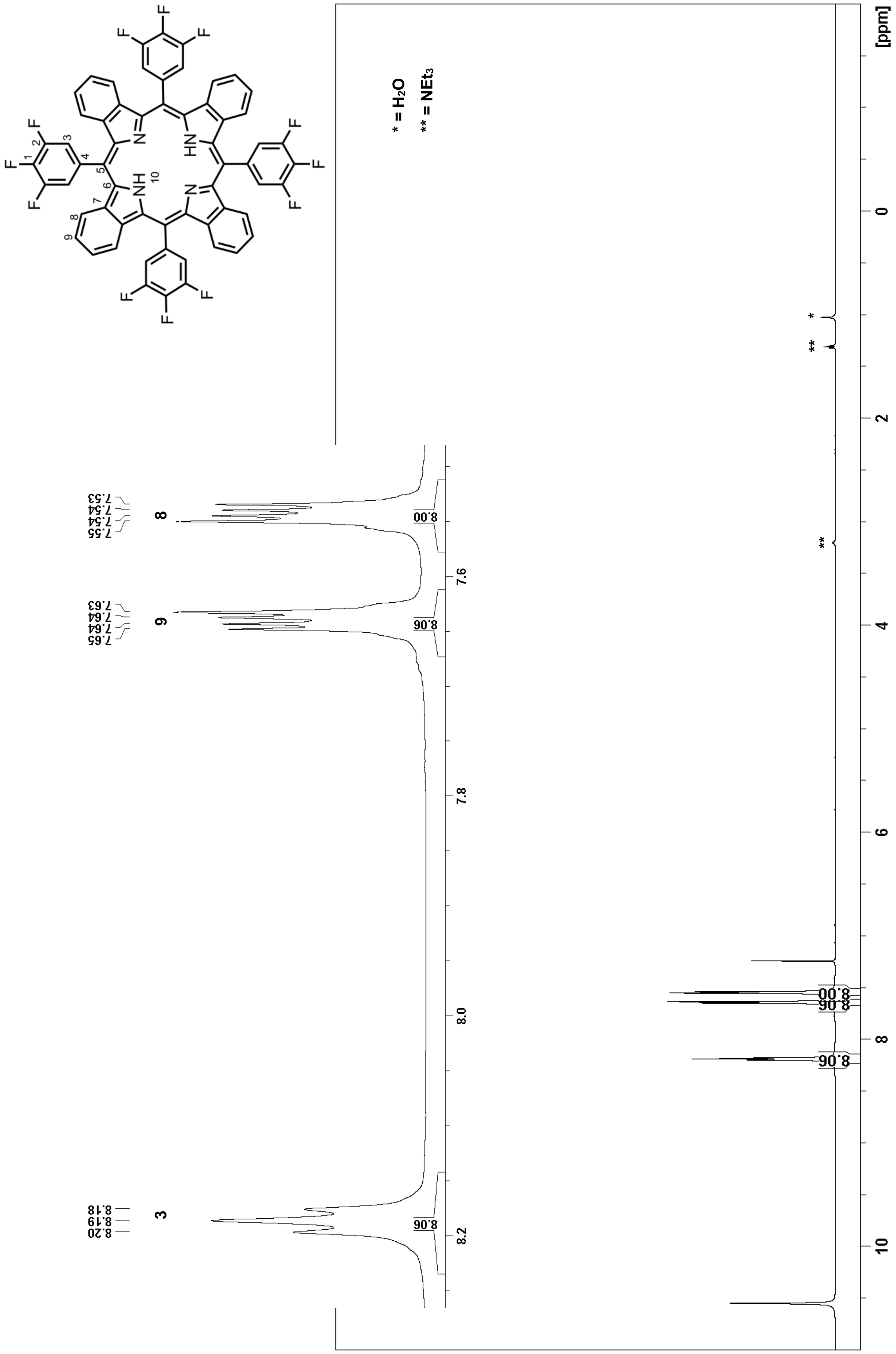

Figure S11. ^{1}H NMR (600 MHz, $CDCl_3$/TFA-d_1, rt) of **4**.

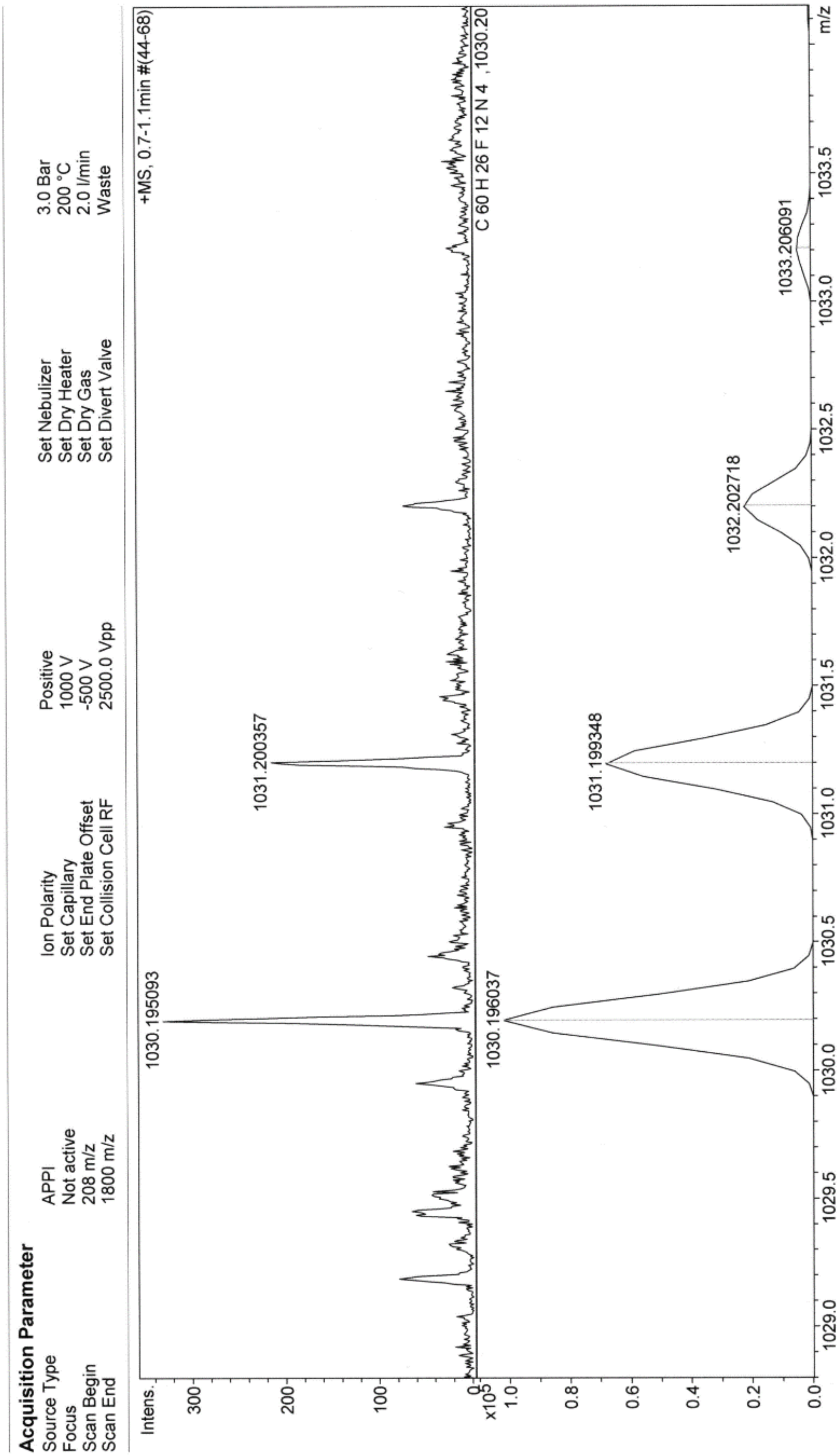

Figure S12. HRMS (APPI, toluene) of **4**.

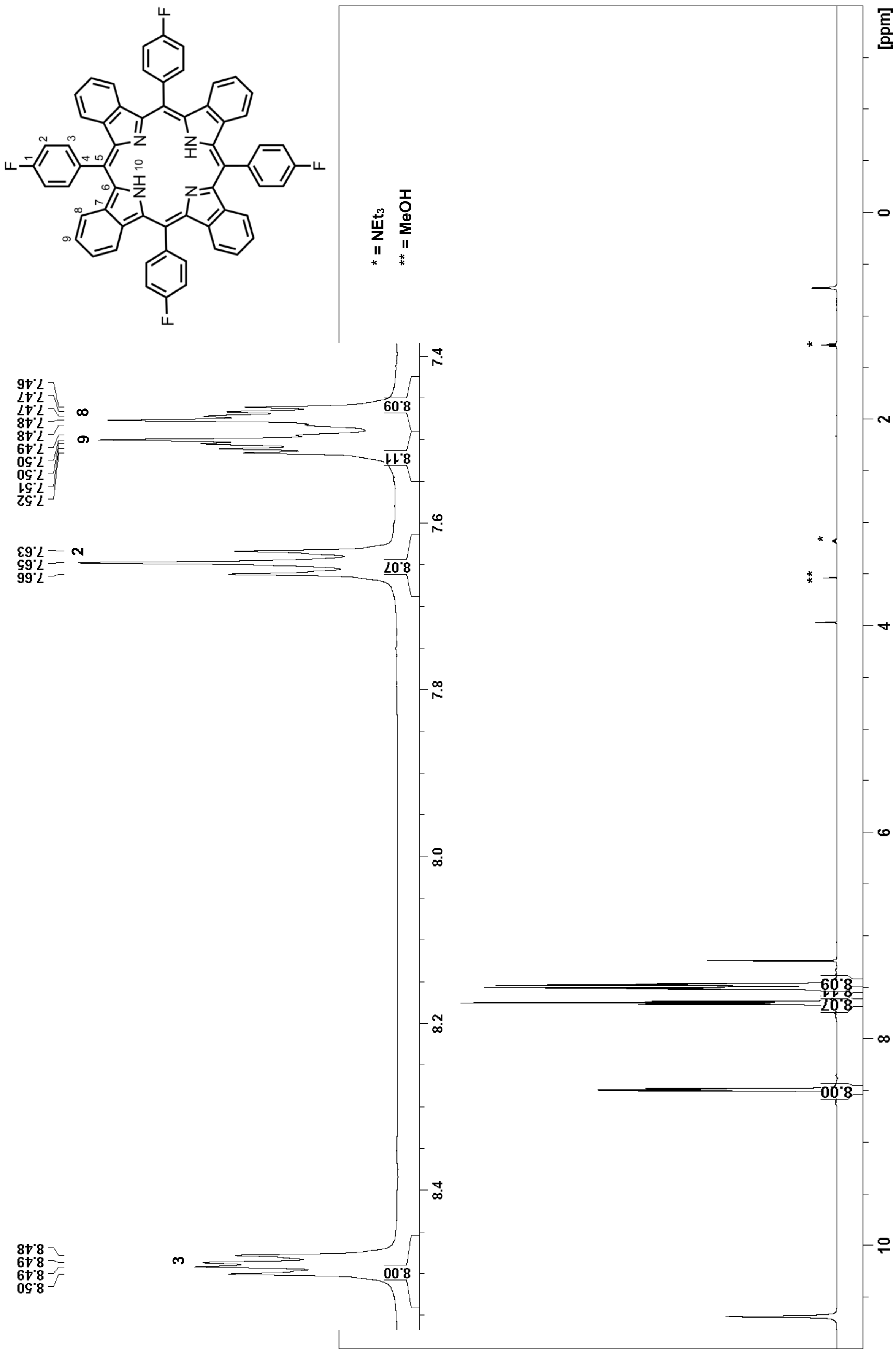

Figure S13. ^{1}H NMR (600 MHz, $CDCl_3$/TFA-d_1, rt) of **5**.

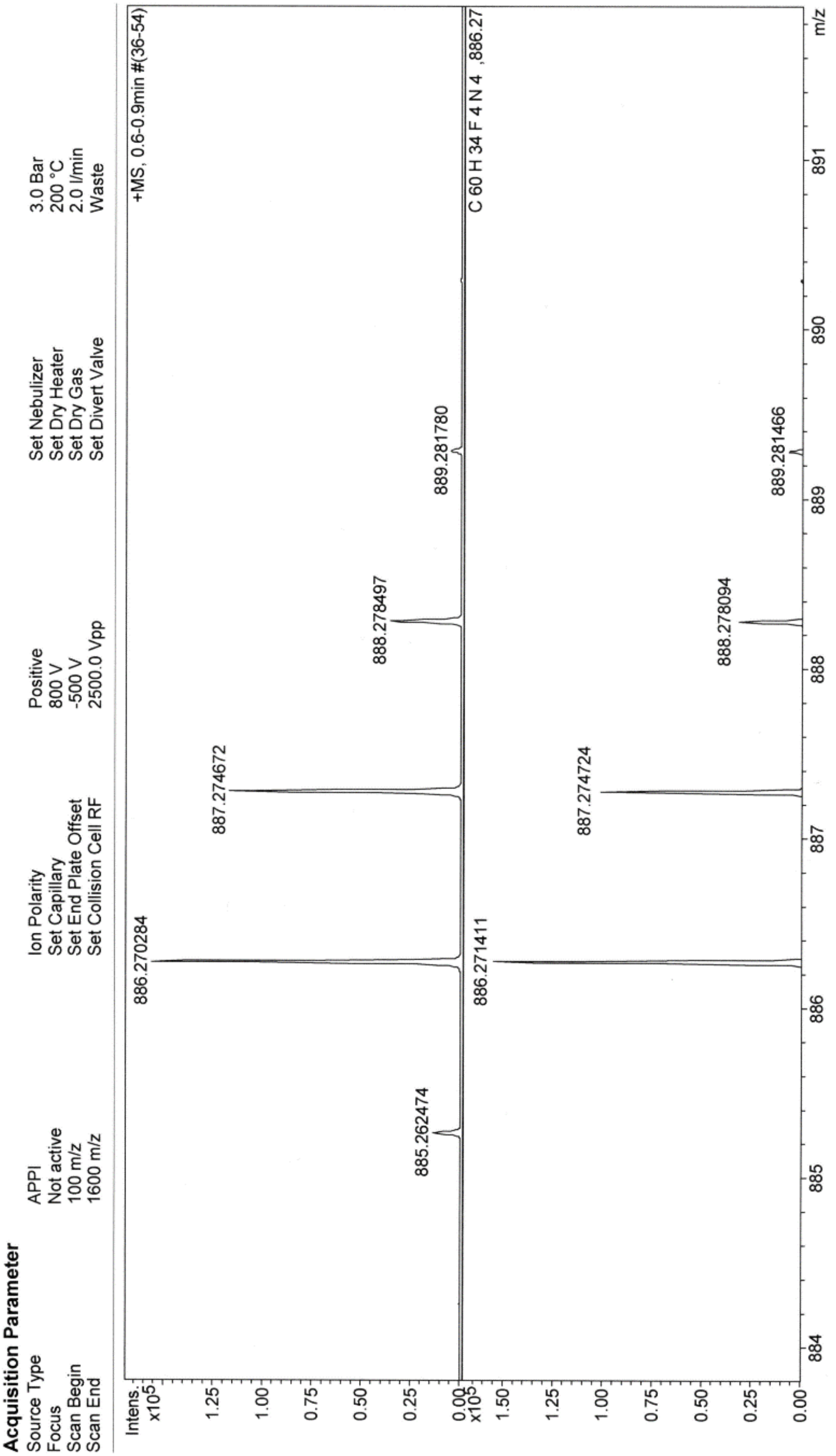

Figure S14. HRMS (APPI, MeCN/toluene) of **5**.

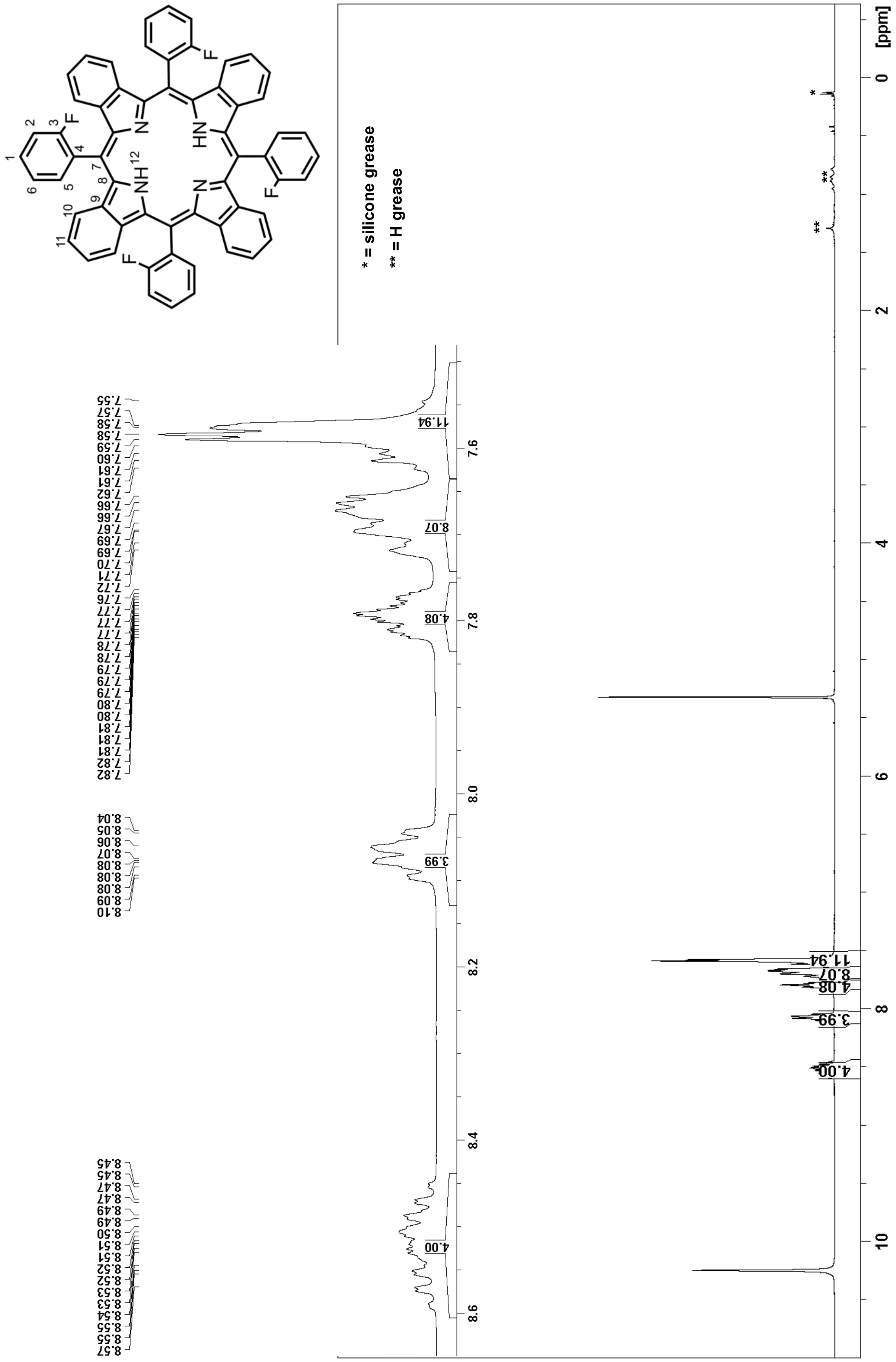

Figure S15. ^{1}H NMR (400 MHz, CD_2Cl_2/TFA-d_1, rt) of **6** (mixture of four atropisomers).

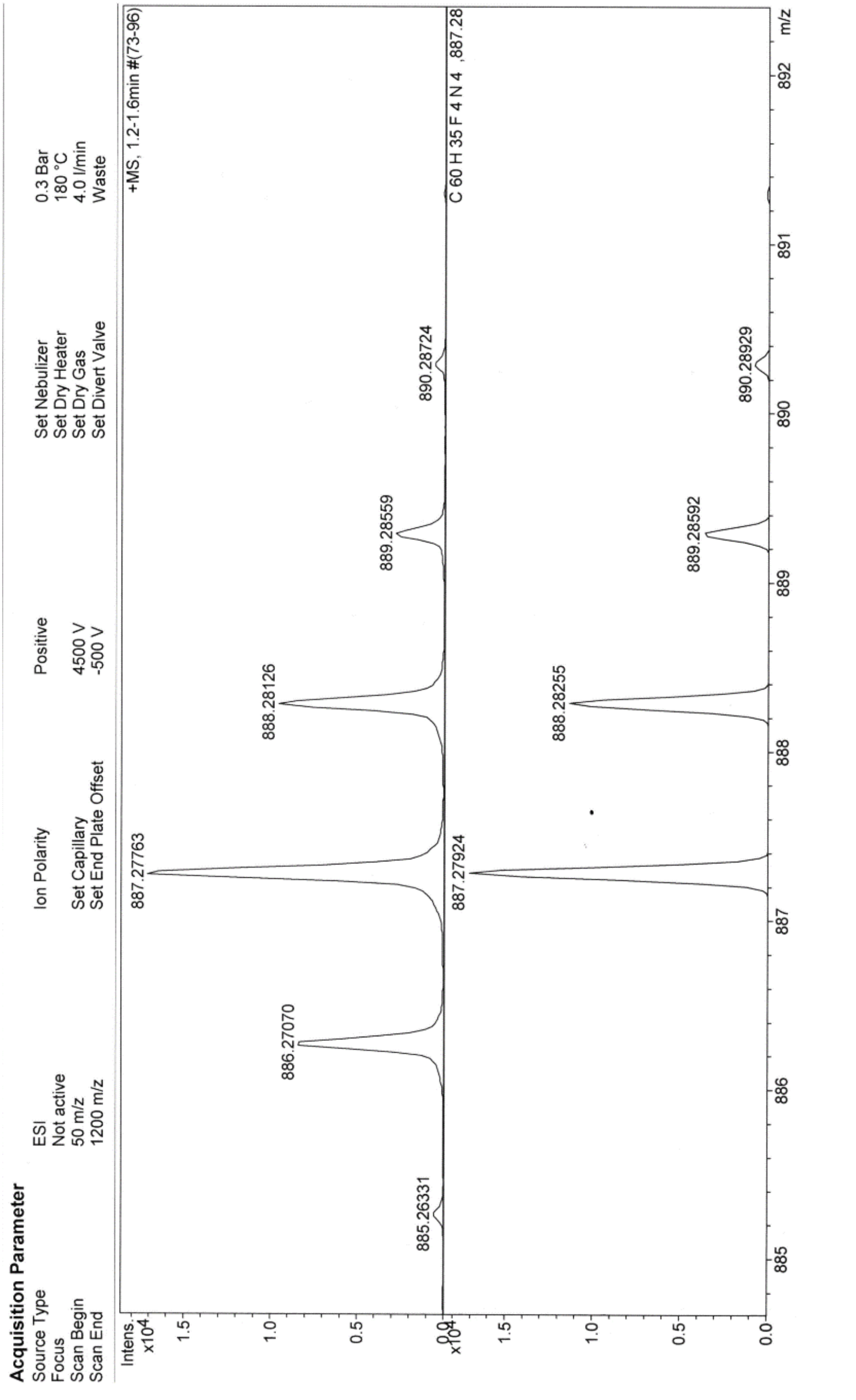

Figure S16. HRMS (ESI, MeCN/toluene) of **6**.

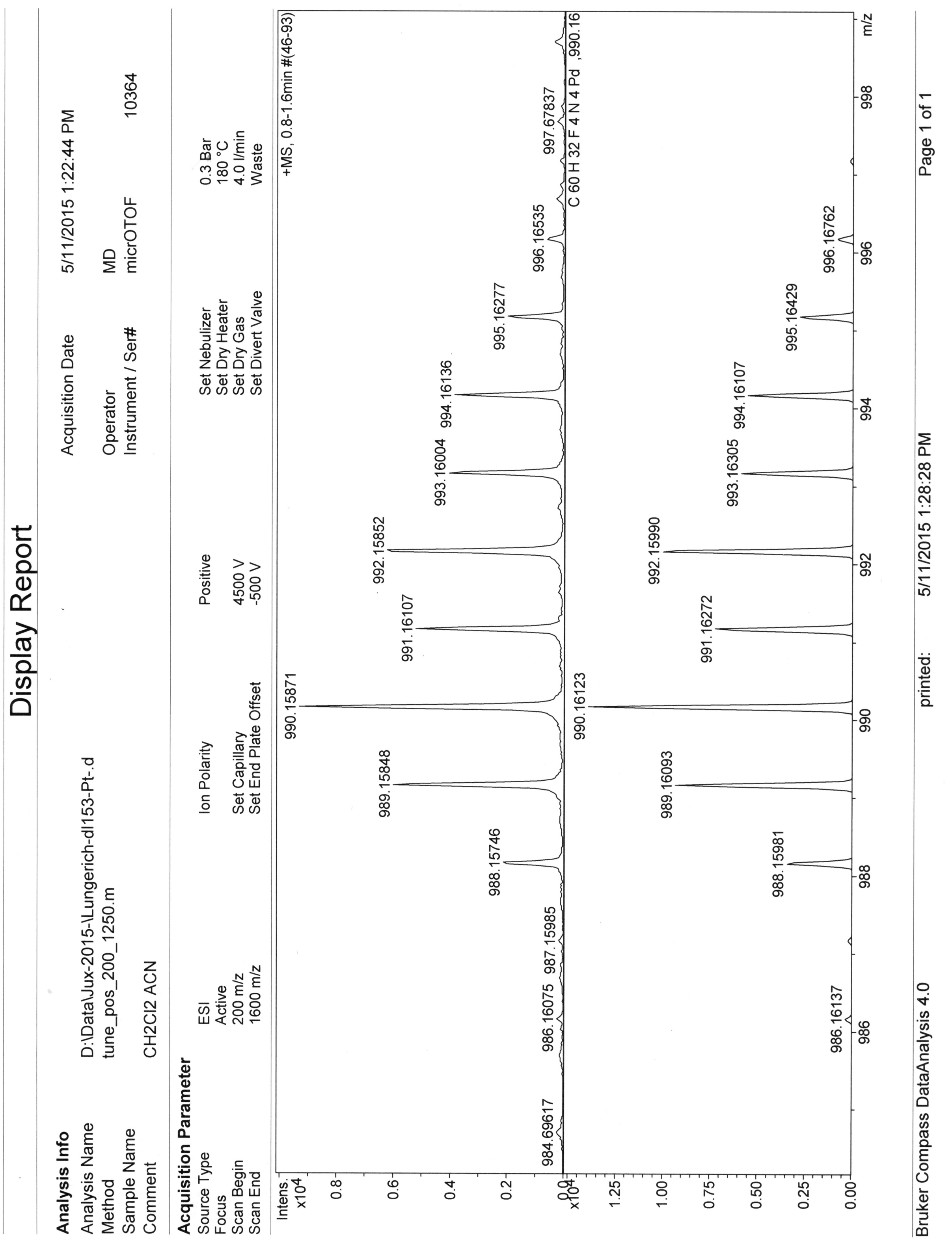

Figure S17. HRMS (ESI, CH_2Cl_2/MeCN) of **6Pd**.

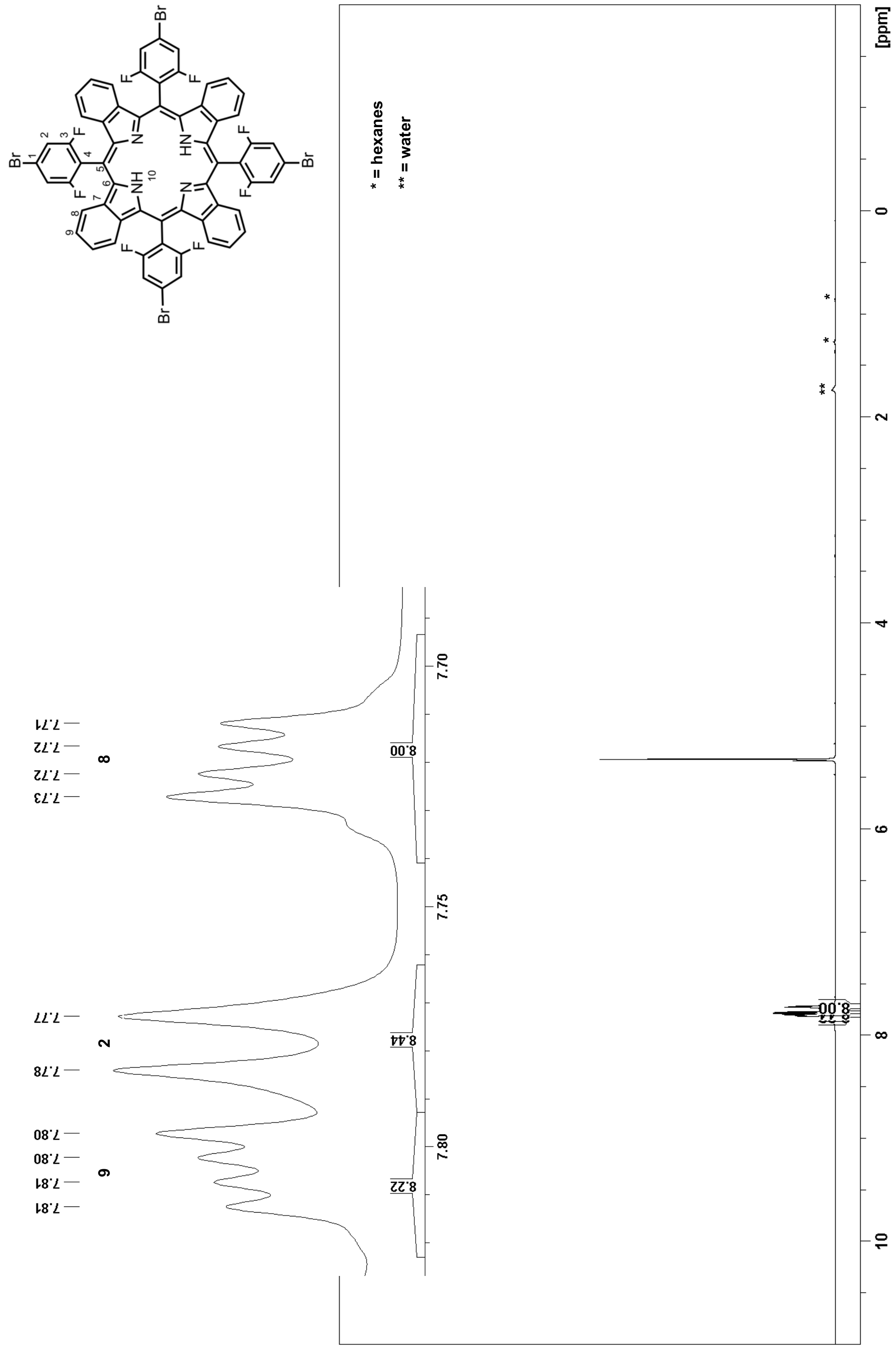

Figure S18. ^{1}H NMR (600 MHz, CD_2Cl_2/TFA-d_1, rt) of **7**.

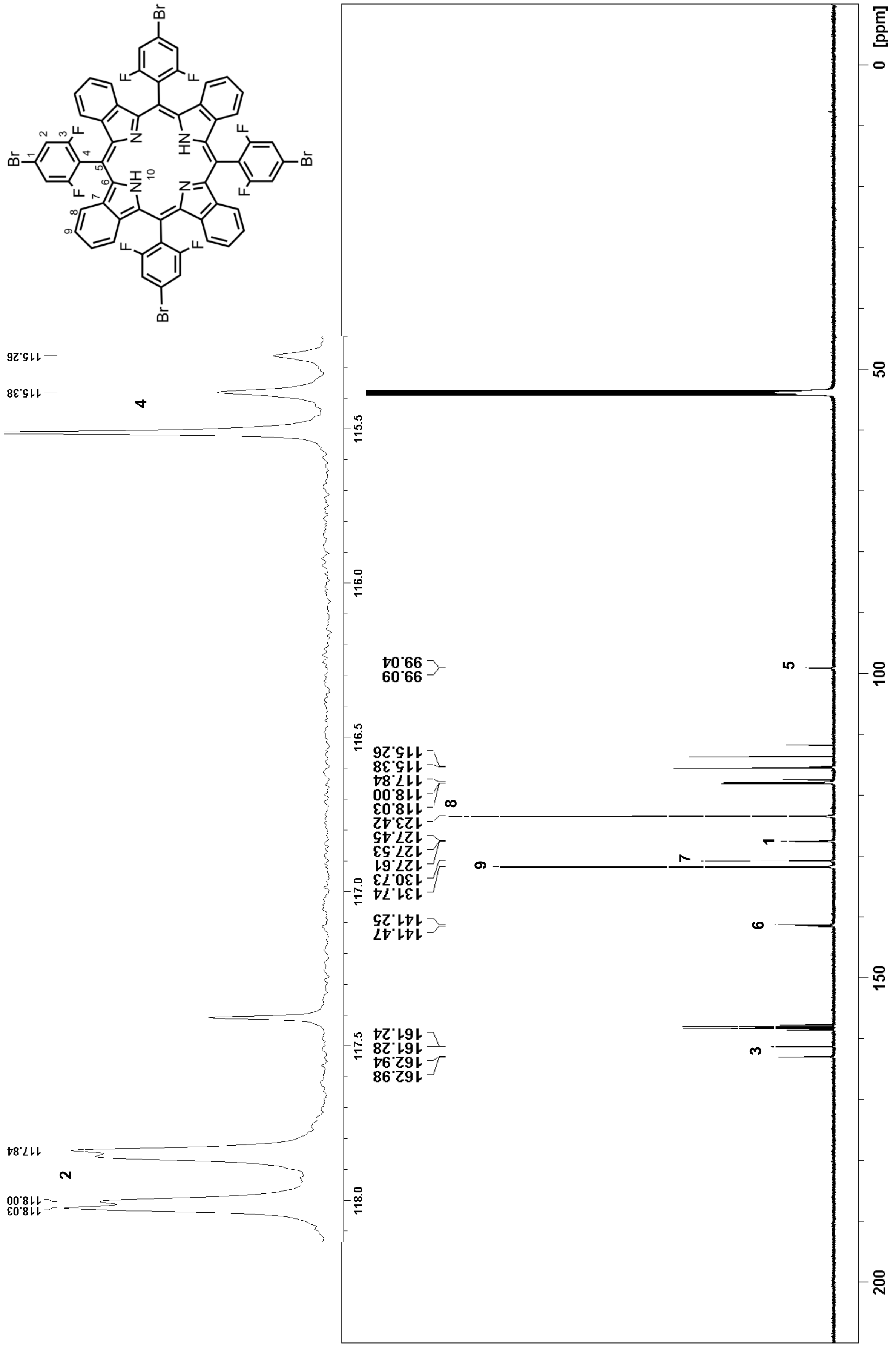

Figure S19. $^{13}C\{^1H\}$ NMR (150 MHz, CD_2Cl_2/TFA-d_1, rt) of **7**.

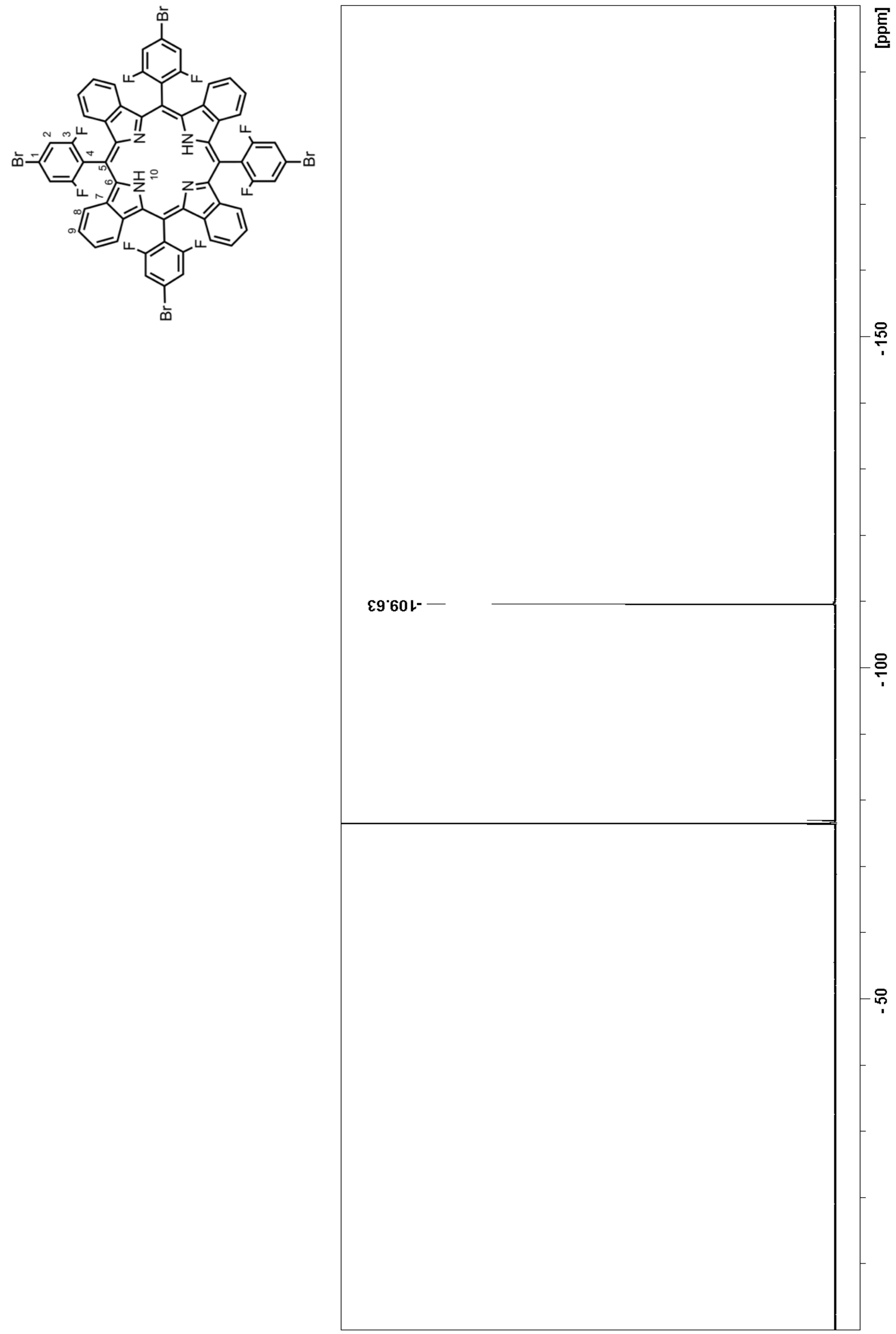

Figure S20. $^{19}F\{^{1}H\}$ NMR (470 MHz, CD_2Cl_2/TFA-d_1, rt) of **7**.

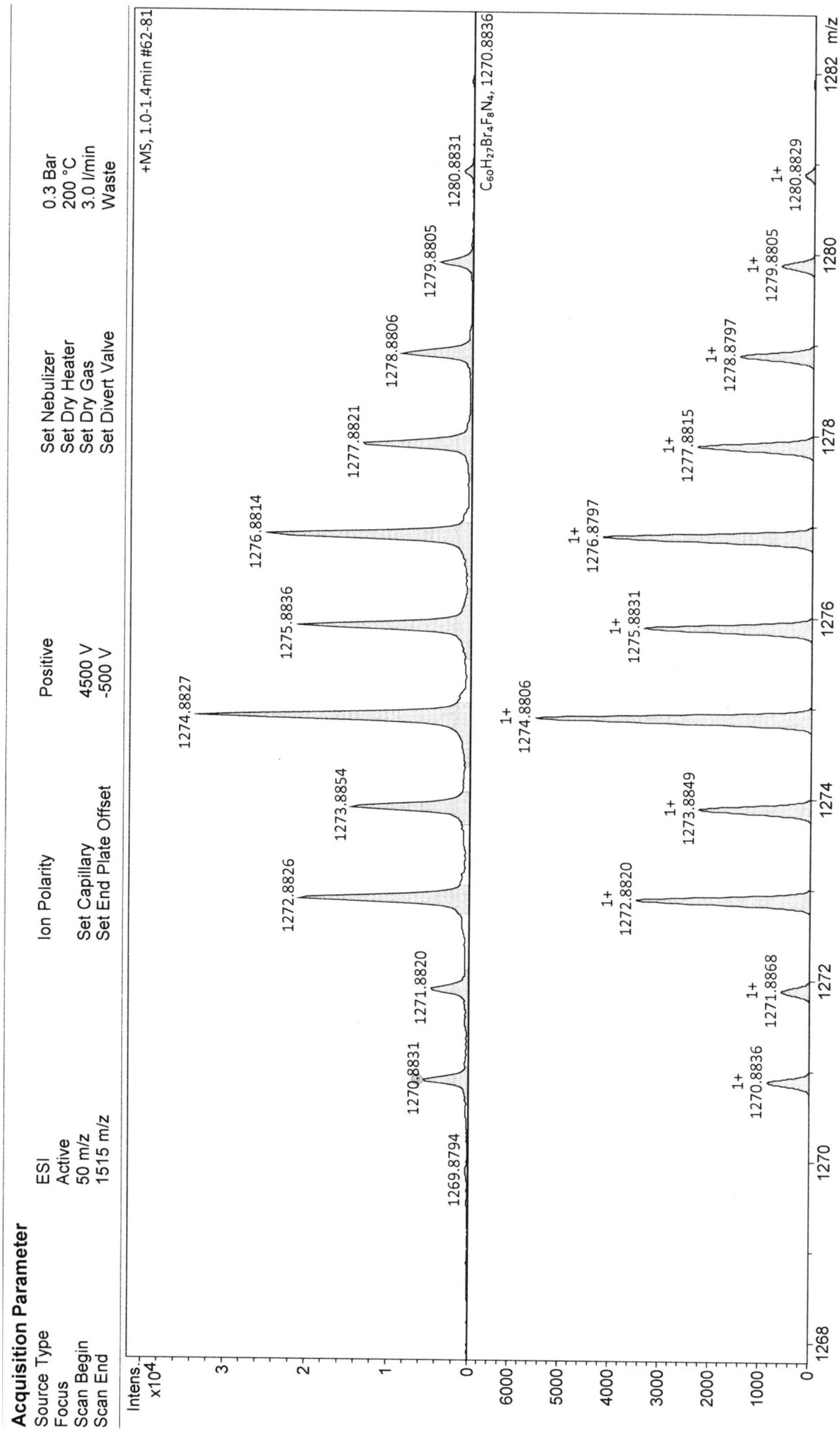

Figure S21. HRMS (ESI, CH_2Cl_2/MeCN) of **7**.

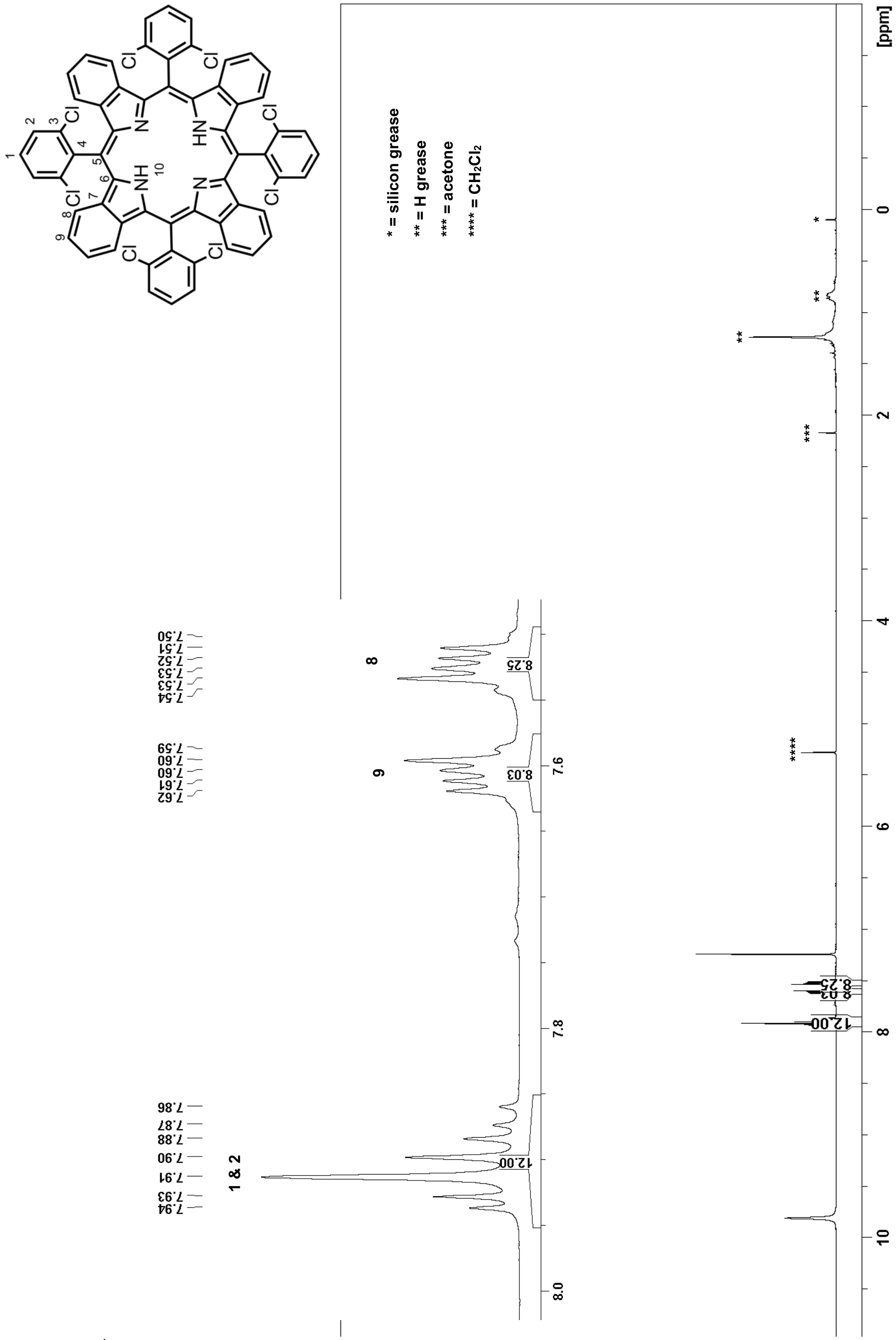

Figure S22. ^{1}H NMR (400 MHz, $CDCl_3$/TFA-d_1) of **8**.

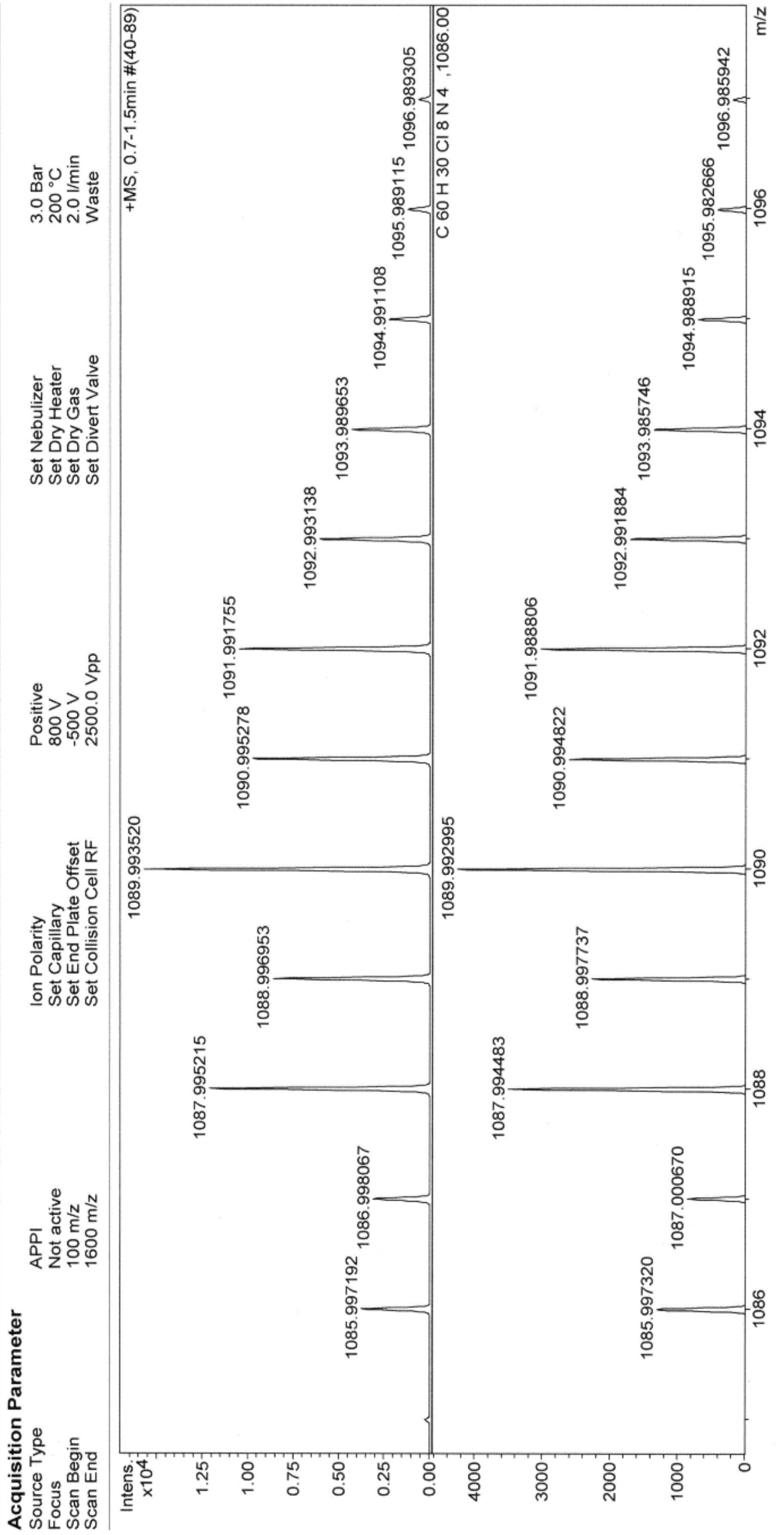

Figure S23. HRMS (APPI, toluene) of **8**.

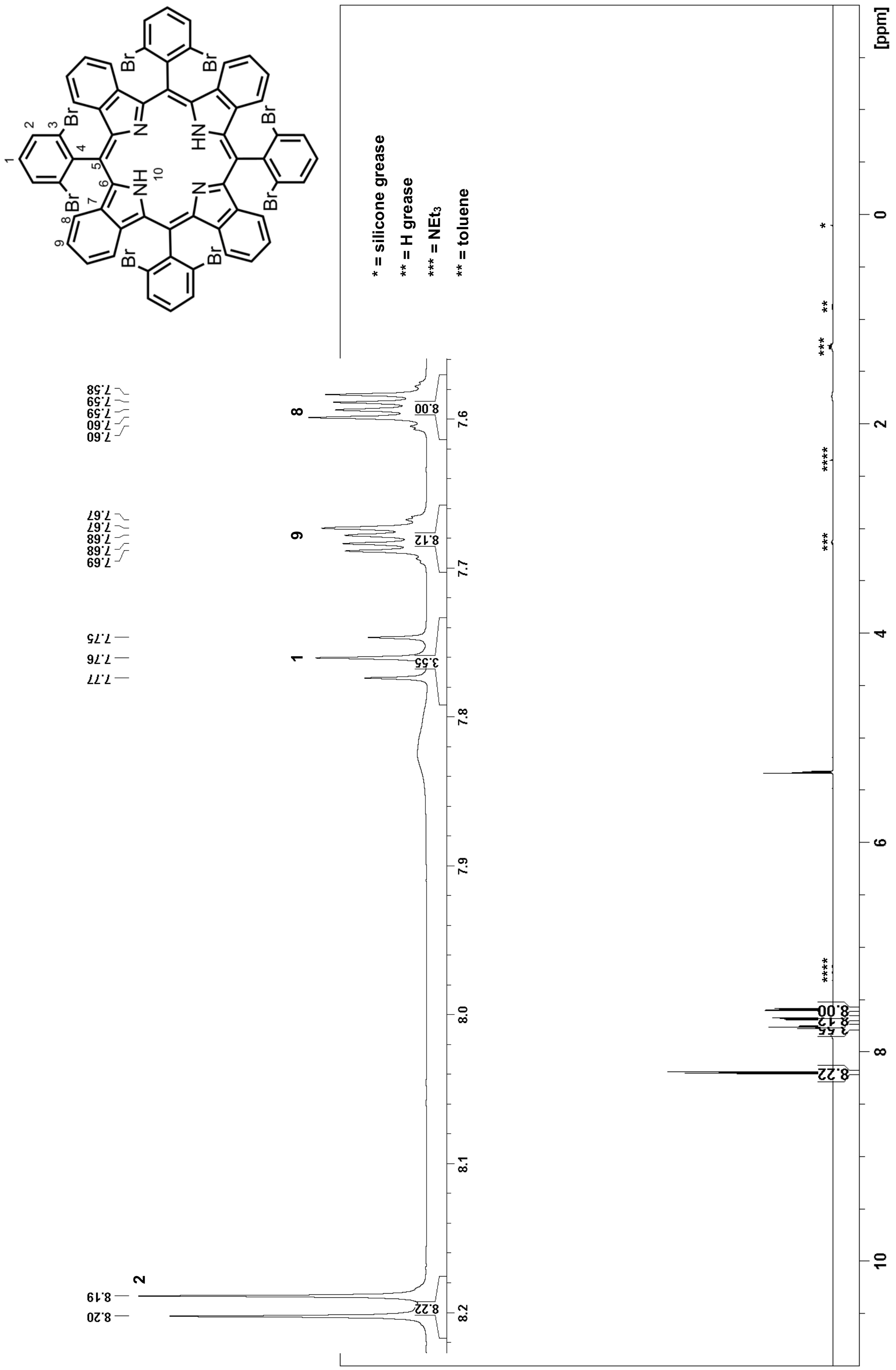

Figure S24. ^1H NMR (600 MHz, CD_2Cl_2/TFA-d_1, rt) of **S1**.

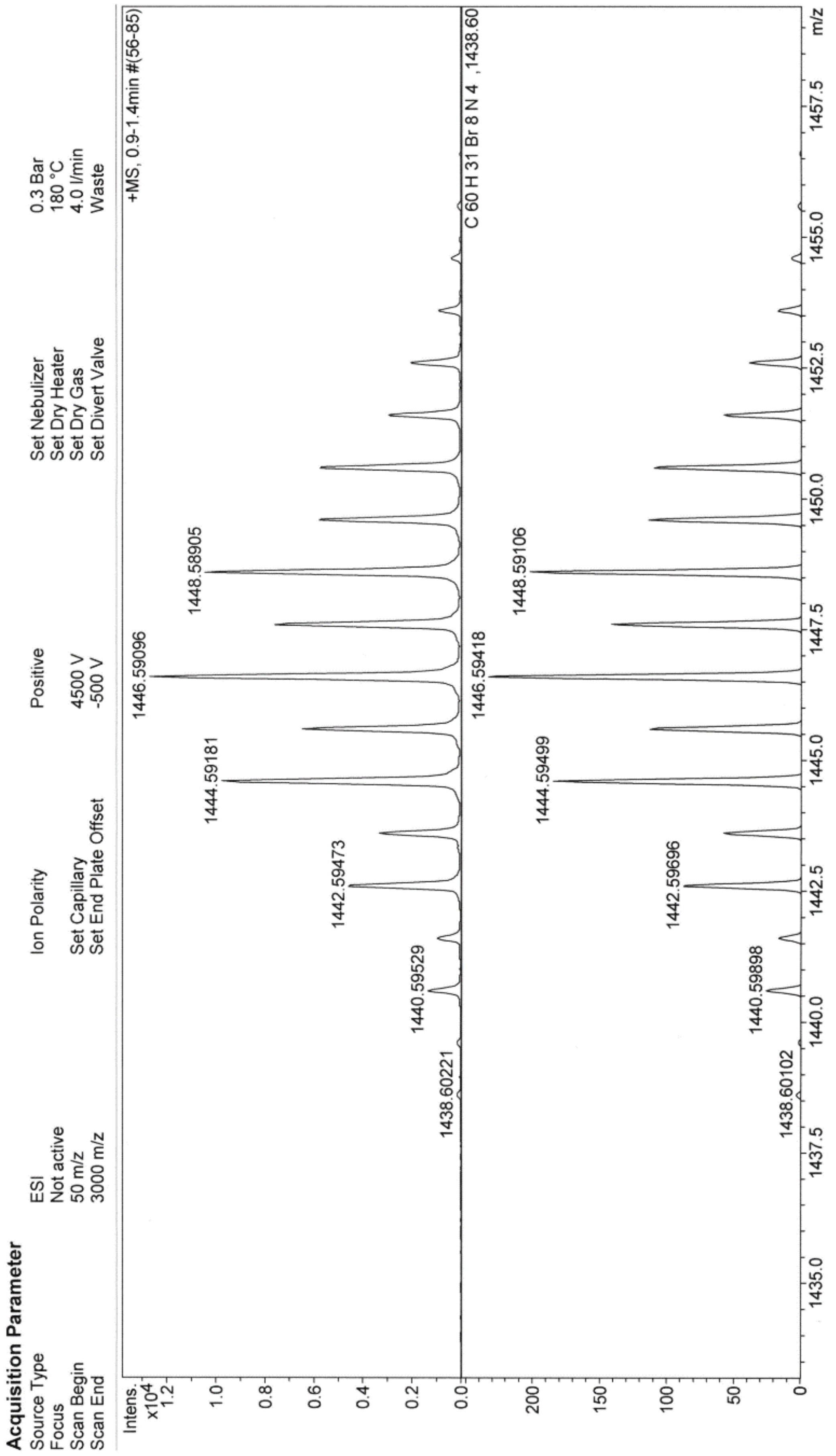

Figure S25. HRMS (ESI) of **S1**.

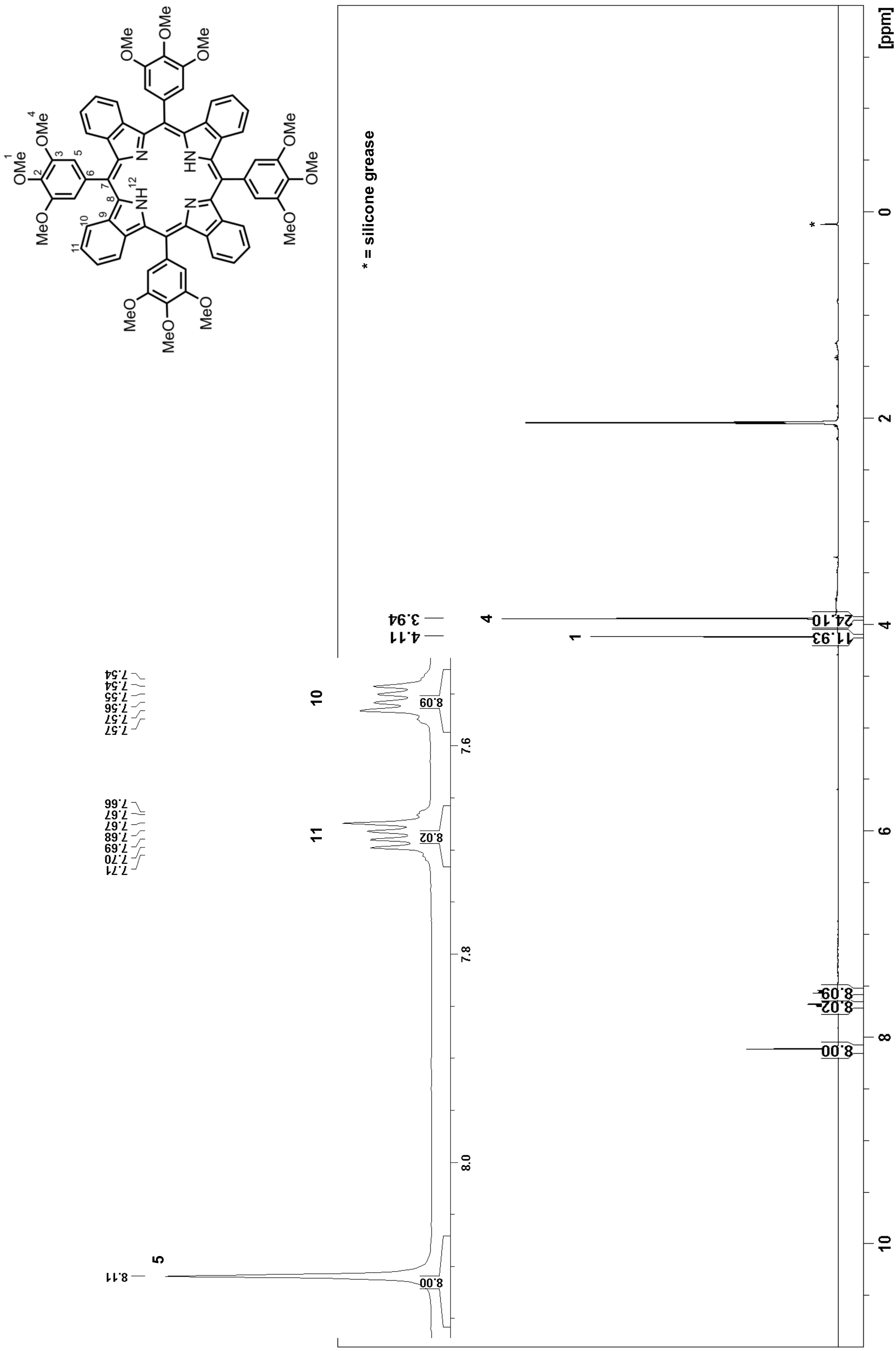

Figure S26. ^{1}H NMR (400 MHz, acetone-d_6/TFA-d_1) of **S2**.

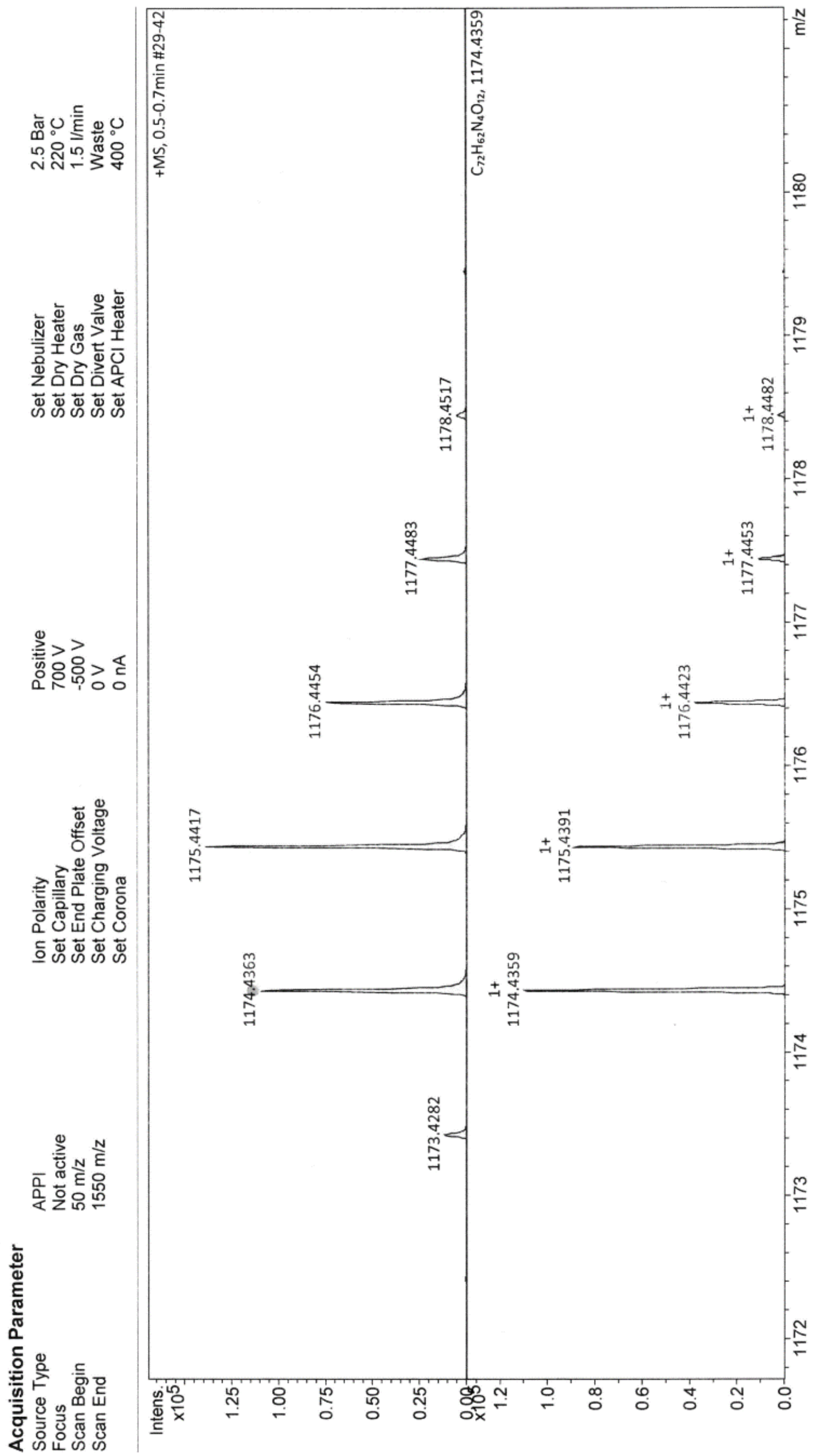

Figure S27. HRMS (APPI, toluene/CH_2Cl_2) of **S2**.

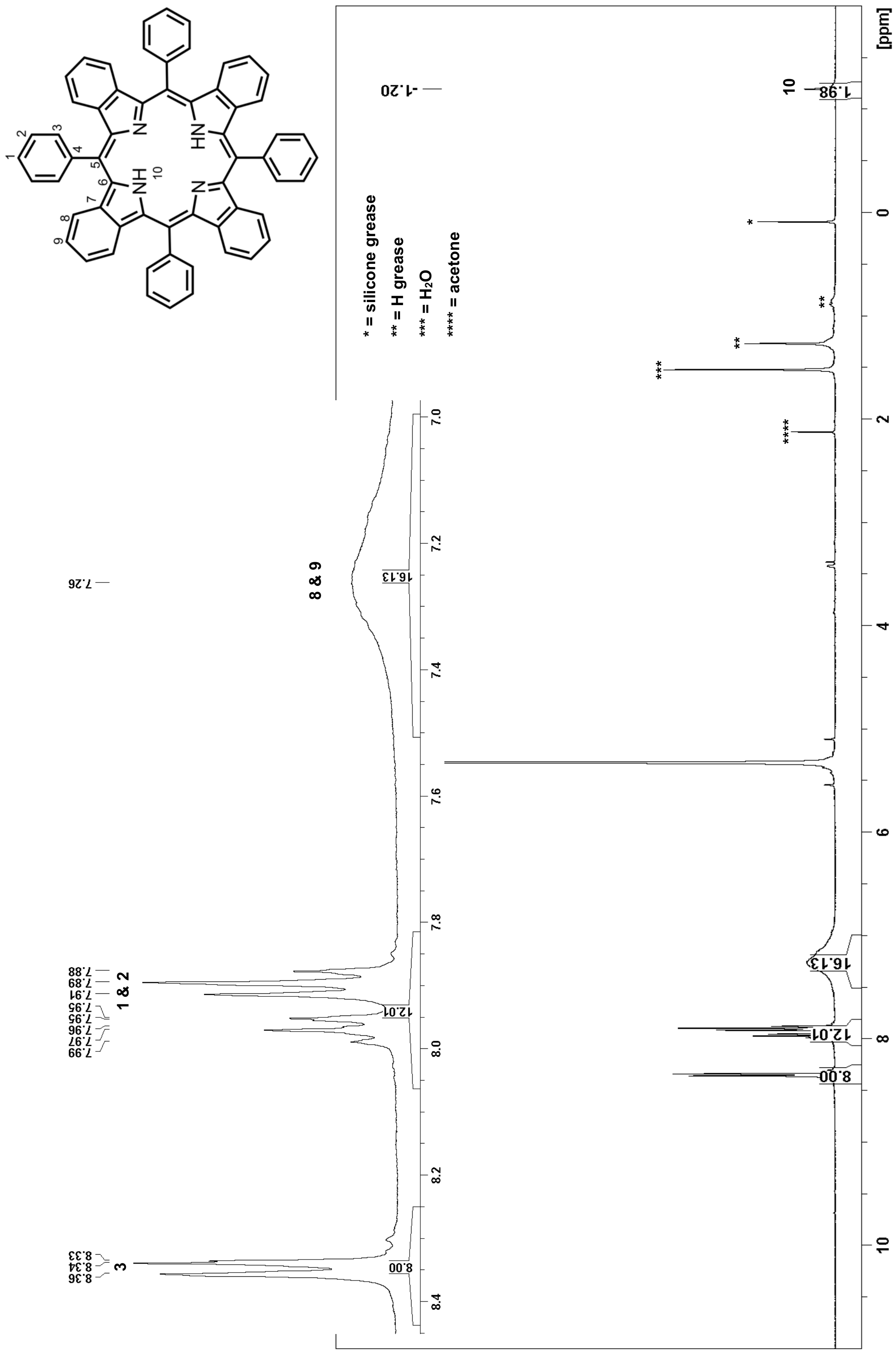

Figure S28. ^{1}H NMR (400 MHz, CD_2Cl_2, rt) of **S3**.

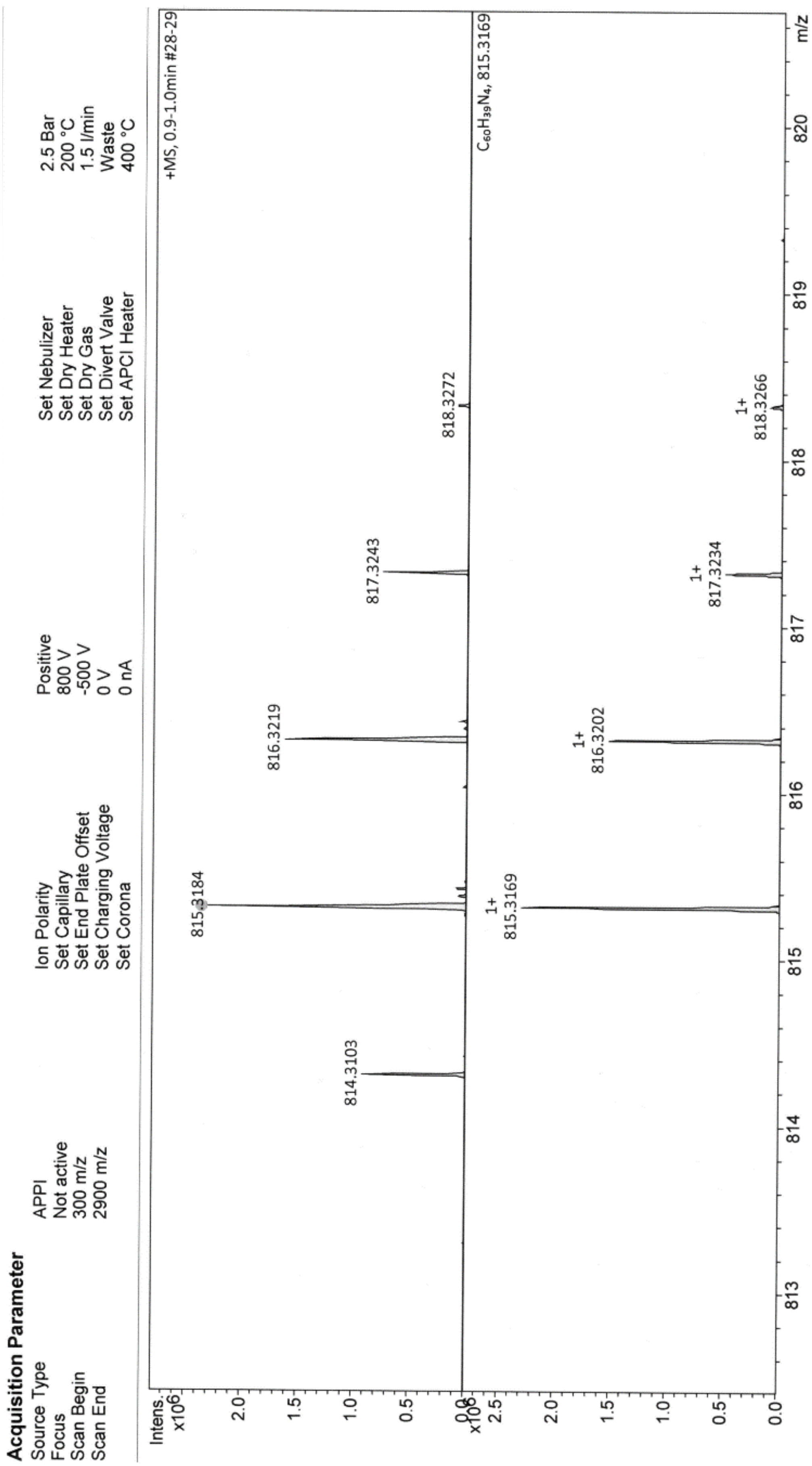

Figure S29. HRMS (APPI, toluene) of **S3**.

1.3 Chapter S1 – wet-chemical approaches to graphyrin

Wet-chemical palladium-catalyzed C–H activation

As a starting point for the conditions of the Pd-catalyzed CH-activation we were inspired by the work of L. T. Scott on indeno-fused corannulenes, who utilized $Pd(PCy_3)_2Cl_2$ as catalyst, DBU as base and DMAc as solvent.[2] We applied this conditions to **8** and analyzed the reaction outcome by mass spectrometry as well as absorption spectroscopy. It is important to mention, that much higher concentration of the catalyst as well as longer reaction times were necessary to initiate the cyclization (see Table S1).

A significant broadening of all absorption features is observed for the reaction outcome in comparison with the reactant as depicted in Figure S30a. Furthermore, a red-shit of the Soret band and the Q-bands is found, which can be interpreted as an indicator for the π-extension of the TATBP macrocycle. According to mass spectrometry, three reaction events could be recognized: 1. C-C bond formation via HCl elimination, 2. Pd-insertion and 3. exchange of residual Cl substituents by hydrogens (hydrodehalogenation). Based on the isotopic pattern, it is assumed that the observed ion peak consists of several species, which differ in the number of formed C-C bonds. If the different constitutional isomers are also considered, it is not surprising that all attempts to separate the mixture by means of column chromatography or HPLC were unsuccessful. Even if the exact product distribution cannot be determined, since all derived ions might differ in their ionization ability, a rough estimation is achieved by simulation of the ion peak. As shown in Figure S30b, the measured spectrum is well represented by the simulated mixture of **8Pd**-5HCl, **8Pd**-4HCl, **8Pd**-3HCl, **8Pd**-2HCl and **8Pd**-1HCl with a distribution of 1 : 7 : 5 : 4 : 4. Furthermore, the same product distribution was found when **8Pd** instead of **8** was used as starting material, which led to the conclusion that palladium insertion occurs early during the reaction.

Moreover, we found an interesting trend of the reaction outcome screening the reaction of **8** in a temperature range from 150 to 300 °C. For temperatures below 180 °C, neither HCl elimination, Pd-insertion nor hydrodehalogenation took place, while a shift of signals to higher *m/z* values is found with increasing temperature. This observation suggests that higher temperatures favor the undesired hydrodehalogenation reaction, which is demonstrated in Figure S31 by simulating the mass spectra of the reaction outcome at 180, 240 and 300 °C, respectively.

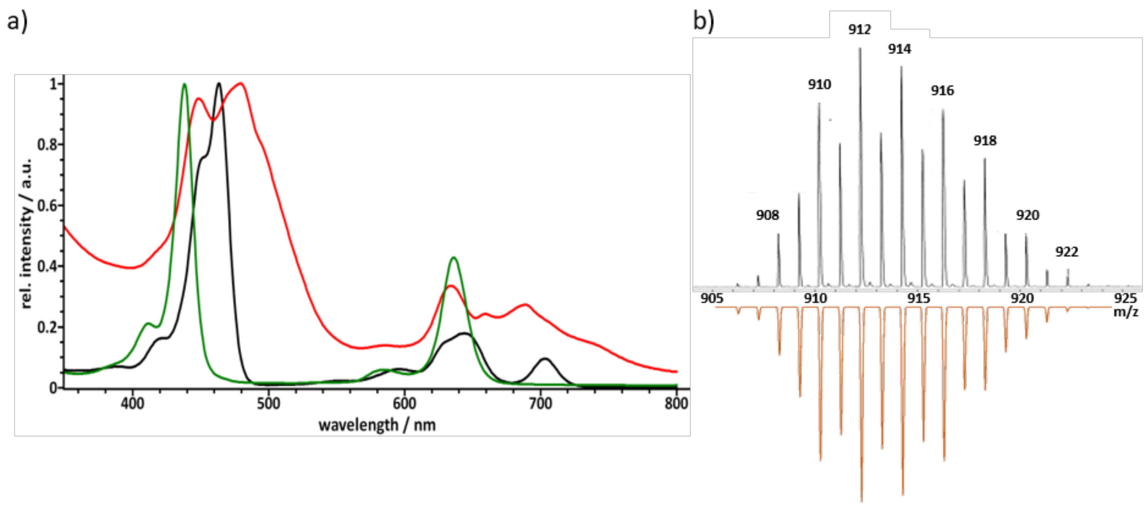

Figure S30. Exemplified UV/Vis and MS analysis of a reaction outcome: a) normalized absorption spectrum of **8** (black line), **8Pd** (green line) and the **reaction outcome** (red line); b) measured (top) and simulated (bottom) mass spectrum of the **reaction outcome**.

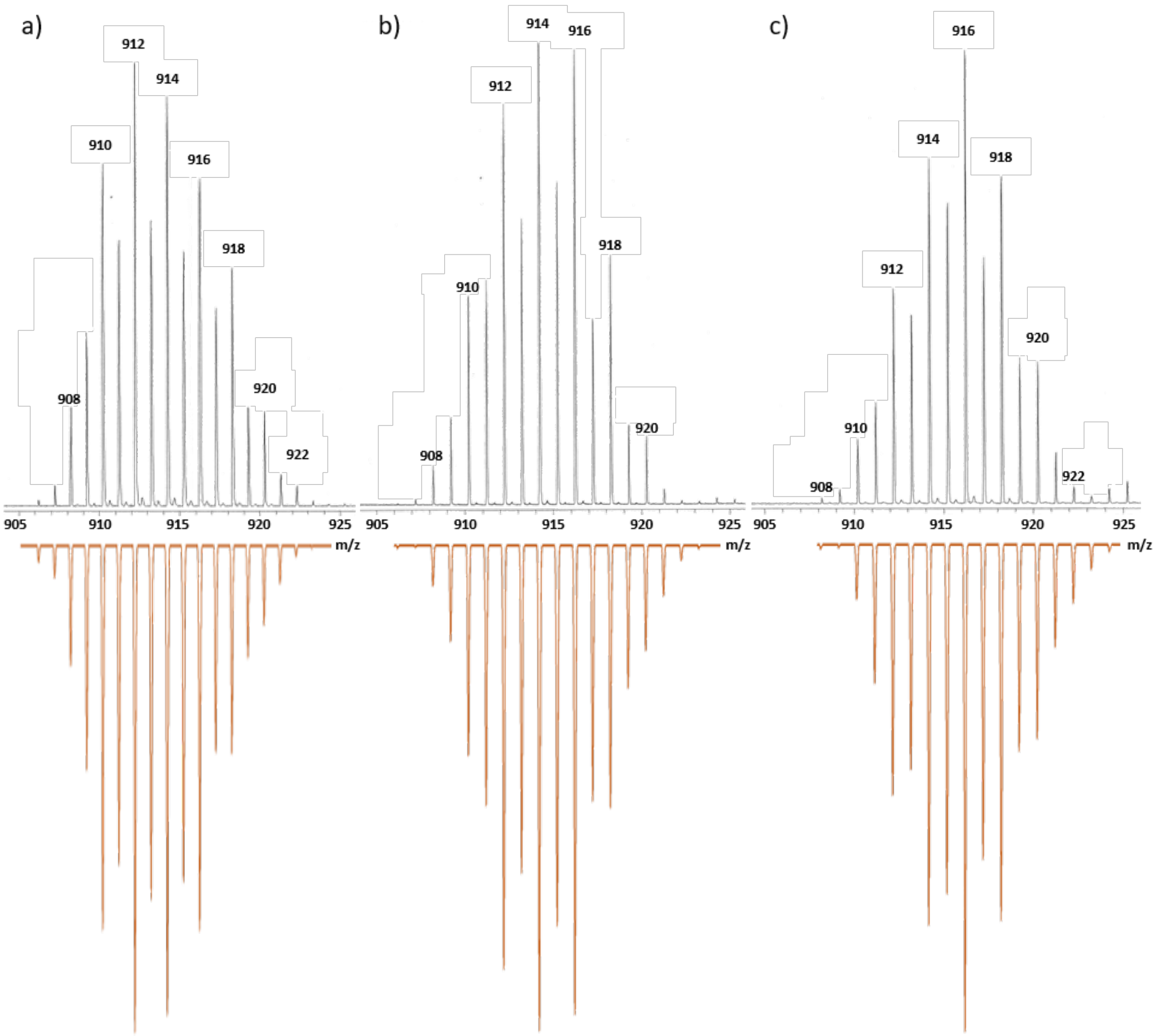

Figure S31. Measured (top) and simulated (bottom) mass spectra after Pd-catalyzed CH-activation of **8** at different reaction temperatures: a) 180 °C; b) 240 °C; c) 300 °C.

Table S1. Experimental conditions for Pd-catalyzed C–H activation: A 5 mL MW vial equipped with a magnetic stir bar was charged with **8** (10 mg), $Pd(PCy_3)_2Cl_2$ (8 eq), and dissolved in DMAc/DBU (2.5 mL, 4:1, v:v). The mixture was deoxygenated and reacted in the microwave reactor: elevated temperatures, 4 h, high absorption level). CH_2Cl_2 (50 mL) was added and the organic layer was washed with 10% aqueous HCl (2x 20 mL), 10% aqueous Na_2CO_3 (2x 20 mL) and brine (20 mL). After drying over $MgSO_4$, the solvent was removed, and the residue was filtered over silica gel (THF). After evaporation of the solvent a dark green solid was obtained.

N	**Temperature**	**Observations**
1	150 °C	No reaction
2	160 °C	No reaction
3	170 °C	No reaction
4	180 °C	Color change to olive, Pd-insertion (metalation), mixture of different cyclization, hydrodehalogenation side reaction.
5	200 °C	Color change to olive, Pd-insertion (metalation), mixture of different cyclization, hydrodehalogenation side reaction.
6	220 °C	Color change to olive, Pd-insertion (metalation), mixture of different cyclization, hydrodehalogenation side reaction.
7	240 °C	Color change to olive, Pd-insertion (metalation), mixture of different cyclization, hydrodehalogenation side reaction.
8	260 °C	Color change to olive, Pd-insertion (metalation), mixture of different cyclization, hydrodehalogenation side reaction.
9	280 °C	Color change to olive, Pd-insertion (metalation), mixture of different cyclization, hydrodehalogenation side reaction.
10	300 °C	Color change to olive, Pd-insertion (metalation), mixture of different cyclization, hydrodehalogenation side reaction.

Since aryl bromides usually show a better reactivity in Pd-catalyzed reactions, we exchanged **8** for its brominated derivate **S1**, but no significant difference was observed as summarized in Table S2.

Table S2. Experimental conditions for Pd-catalyzed C–H activation: A 5 mL MW vial equipped with a magnetic stir bar was charged with **S1** (10 mg), $Pd(PCy_3)_2Cl_2$, and dissolved in DMAc/DBU (2.5 mL, 4:1, v:v). The mixture was deoxygenated and reacted in the microwave reactor: elevated temperatures, 4 h, high absorption level). CH_2Cl_2 (50 mL) was added and the organic layer was washed with 10% aqueous HCl (2x 20 mL), 10% aqueous Na_2CO_3 (2x 20 mL) and brine (20 mL). After drying over $MgSO_4$, the solvent was removed, and the residue was filtered over silica gel (THF). After evaporation of the solvent a dark green solid was obtained.

N	**conditions**	**observations**
11	[Pd] (8 eq), 180 °C	Color change to olive, Pd-insertion (metalation), mixture of different cyclization, hydrodehalogenation side reaction
12	[Pd] (8 eq), 130 °C	No reaction
13	[Pd] (8 eq), 140 °C	No reaction
14	[Pd] (8 eq), 150 °C	No reaction
13	[Pd] (8 eq), 160 °C	Color change to olive, Pd-insertion (metalation), mixture of different cyclization, hydrodehalogenation side reaction
15	[Pd] (8 eq), 170 °C	Color change to olive, Pd-insertion (metalation), mixture of different cyclization, hydrodehalogenation side reaction
17	[Pd] (8 eq), 200 °C	Color change to olive, Pd-insertion (metalation), mixture of different cyclization, hydrodehalogenation side reaction
18	[Pd] (10 eq), 120 °C, 14 d	Color change to olive, Pd-insertion (metalation), mixture of different cyclization, hydrodehalogenation side reaction
19	[Pd] (10 eq), DMAc (2.5 mL), Cs_2CO_3, 150 °C	No reaction
20	[Pd] (10 eq), DMAc (2.5 mL), P_1-*t*Bu, 150 °C	No reaction
21	[Pd] (9 eq), DMF/DBU (2.5 mL, 4:1, v:v), 160 °C, 4 d	Color change to olive, Pd-insertion (metalation), mixture of different cyclization, hydrodehalogenation side reaction
22	[Pd] (9 eq), Dioxane/DBU (2.5 mL, 4:1, v:v), 160 °C, 4 d	Color change to olive, Pd-insertion (metalation), mixture of different cyclization, hydrodehalogenation side reaction
23	[Pd] (9 eq), THF/DBU (2.5 mL, 4:1, v:v), 160 °C, 4 d	Color change to olive, Pd-insertion (metalation), mixture of different cyclization, hydrodehalogenation side reaction
24	[Pd] (9 eq), MeCN/DBU (2.5 mL, 4:1, v:v), 160 °C, 4 d	No reaction

Wet-chemical intramolecular oxidative cyclodehydrogenation

In addition to the Pd-catalyzed cyclization attempts, the intramolecular oxidative cyclodehydrogenation pathway was tested (see examples in Table S3). It has been demonstrated that this approach is a powerful tool for fusing aromatic units at the *meso*-β–position of the porphyrin macrocycle via 5- or 6-membered ring formation.[3,4] Therefore, **S2** as well as **S2Ni** or **S3** were tested under standard conditions using $FeCl_3$ in $MeNO_2$, but no reaction was observed and only starting material was recovered. Even harsher reaction conditions, like e.g. DDQ and Trifluoromethanesulfonic acid did not lead to any C-C bond formation, however partial hydrolysis of the methyl ether was detected by mass spectrometry.

Experimental procedures for oxidative cyclodehydrogenation attempts

$FeCl_3$ route: A 250 mL Schlenk-RBF equipped with a magnetic stir bar, a rubber septum and a glass tube was charged with **S2** (50 mg, 42.5 µmol) [or **S2Ni** (52 mg, 42.2 µmol) or **S3** (35 mg, 42.9 µmol)] and dissolved in CH_2Cl_2 (150 mL). The mixture was cooled to -20 °C and deoxygenated by bubbling N_2 through the solution for 20 min. A solution of $FeCl_3$ (148 mg, 910 µmol) in $MeNO_2$ (0.5 mL) was added slowly while bubbling vigorously N_2 into the reaction mixture at -20 °C. After 60 minutes, the N_2 bubbling was stopped and the mixture was stirred under N_2 atmosphere for 16 h, while the temperature slowly raised to rt. The reaction was quenched by addition of MeOH (5 mL). The organic layer was washed with saturated aqueous $NaHCO_3$ (2x 100 mL) and brine (100 mL). After drying over $MgSO_4$, the solvent was removed and the residue was filtered over silica gel (THF/CH_2Cl_2, 2:1, v:v). After evaporation of the solvent a green solid was obtained.

DDQ/TfOH route: A 250 mL Schlenk-RBF equipped with a magnetic stir bar, a rubber septum and a glass tube was charged with **S2** (50 mg, 42.5 µmol [or **S3** (40 mg, 49.1 µmol)], DDQ (106 mg, 468 µmol) and dissolved in CH_2Cl_2 (150 mL). The mixture was cooled to -20 °C and deoxygenated by bubbling N_2 through the solution for 20 min. Trifluoromethanesulfonic acid (83 µL, 141 mg, 936 µmol) was added and the mixture was stirred under N_2 atmosphere for 16 h, while the temperature slowly raised to rt. The reaction was quenched by addition of MeOH (5 mL). The organic layer was washed with 10% aqueous Na_2SO_3 (150 mL), 10% aqueous Na_2CO_3 (150 mL) and brine (100 mL). After drying over $MgSO_4$, the solvent was removed and the residue

was filtered over silica gel (THF/CH_2Cl_2, 2:1, v:v). After evaporation of the solvent a green solid was obtained.

Table S3. Summary of distinct examples of intramolecular cyclodehydrogenation attempts of different TATBPs.

N	**Compound**	**Conditions**	**Observations**
1	**S2**	$FeCl_3$, $MeNO_2$, CH_2Cl_2	No reaction, starting material recovered
2	**S2Ni**	$FeCl_3$, $MeNO_2$, CH_2Cl_2	No reaction, starting material recovered
3	**S2**	DDQ, TfOH	Ether hydrolysis
4	**S3**	$FeCl_3$, $MeNO_2$, CH_2Cl_2	No reaction, starting material recovered
5	**S3**	DDQ, TfOH	No reaction, starting material recovered

2. MASS SPECTROMETRIC SECTION

2.1 General information

ESI experiments were conducted in positive ion-mode and with two different ESI mass spectrometers.

The first instrument was an ultra-high resolution Fourier transform ion cyclotron resonance (FT-ICR) mass spectrometer (solariX, Bruker, Bremen, Germany). The following settings were applied. Flow rate of the sample solution by syringe pump infusion 5.0 µLmin^{-1}, nebuliser nitrogen pressure 400 hPa, capillary entrance voltage -5.0 kV, spray shield voltage -4.5 kV, nitrogen dry gas temperature 453 K, dry gas flow rate 4.0 L/min. Energy-resolved collision-induced dissociations (MS^2, CID) were conducted in a collision cell following a mass selecting quadrupole and preceding the high-resolution daughter ion analysis in the Paracell^{TM} analyser. Argon (Ar) served as the collision gas. For the measurements at the solariX mass spectrometer, the samples were dissolved separately in DCM and then diluted individually in acetonitrile (ACN) with 0.2% formic acid. The solution (0.005 mg/mL) was introduced to the ESI-MS ionization chamber by direct infusion.

The second instrument was an ESI-quadrupole time-of-flight (qToF) mass spectrometer (maXis, Bruker, Bremen, Germany). The following settings were applied. Flow rate of the sample solution by syringe pump infusion 3.0 µLmin^{-1}, nebuliser nitrogen pressure 400 hPa, capillary entrance voltage -4.3 kV, spray shield voltage -3.8 kV, nitrogen dry gas temperature 473 K, dry gas flow rate 4.0 L/min. Energy-resolved collision-induced dissociations (MS^2, CID) were conducted under multiple collision conditions in a collision quadrupole following a mass selecting quadrupole and preceding the high-resolution daughter ion analysis in the TOF analyser. Nitrogen (N_2) served as the collision gas.

For the maXis qToF instrument, the porphyrins were dissolved separately in dichloromethane (DCM) and then diluted individually with a mixture of MeOH:DCM (Volume ratio 2:1). After thorough mixing, the 10^{-5} M solution was introduced to the ESI-MS ionization chamber by direct infusion.

2.2 Chapter S2 – Dissociation Experiments of 8 and 8Pd

QTOF experiments with 8

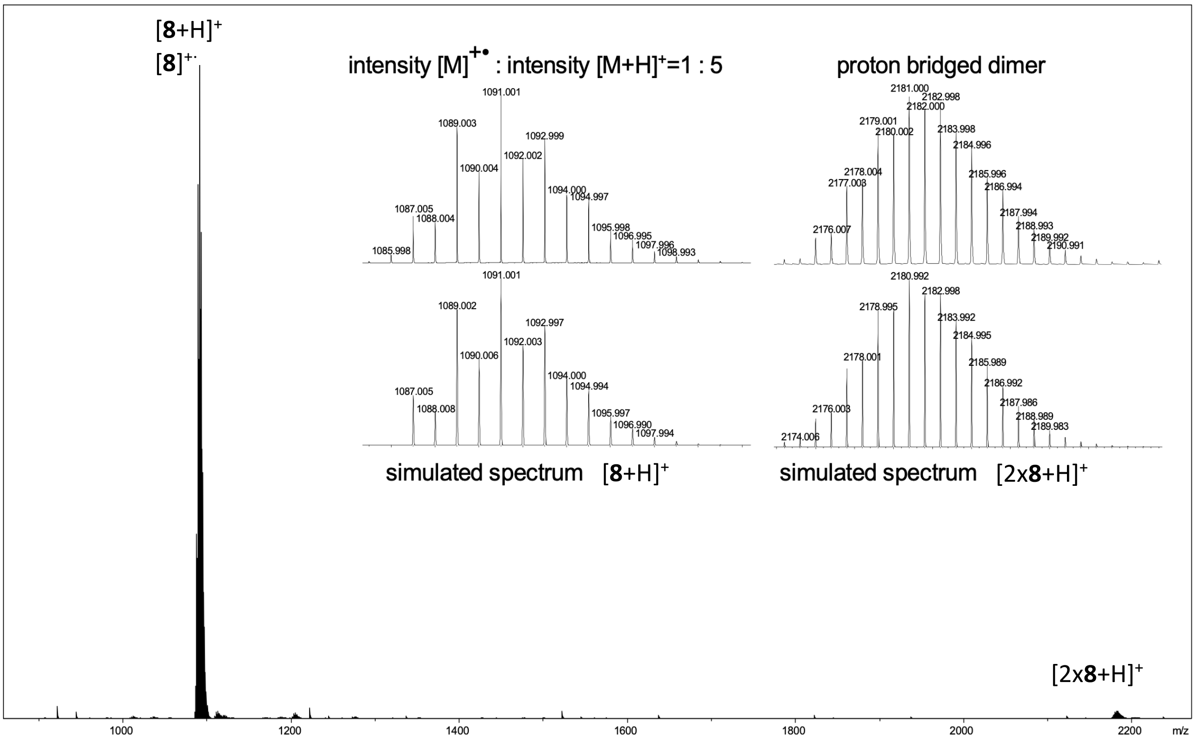

Figure S32. Analysis of precursor ion nature.

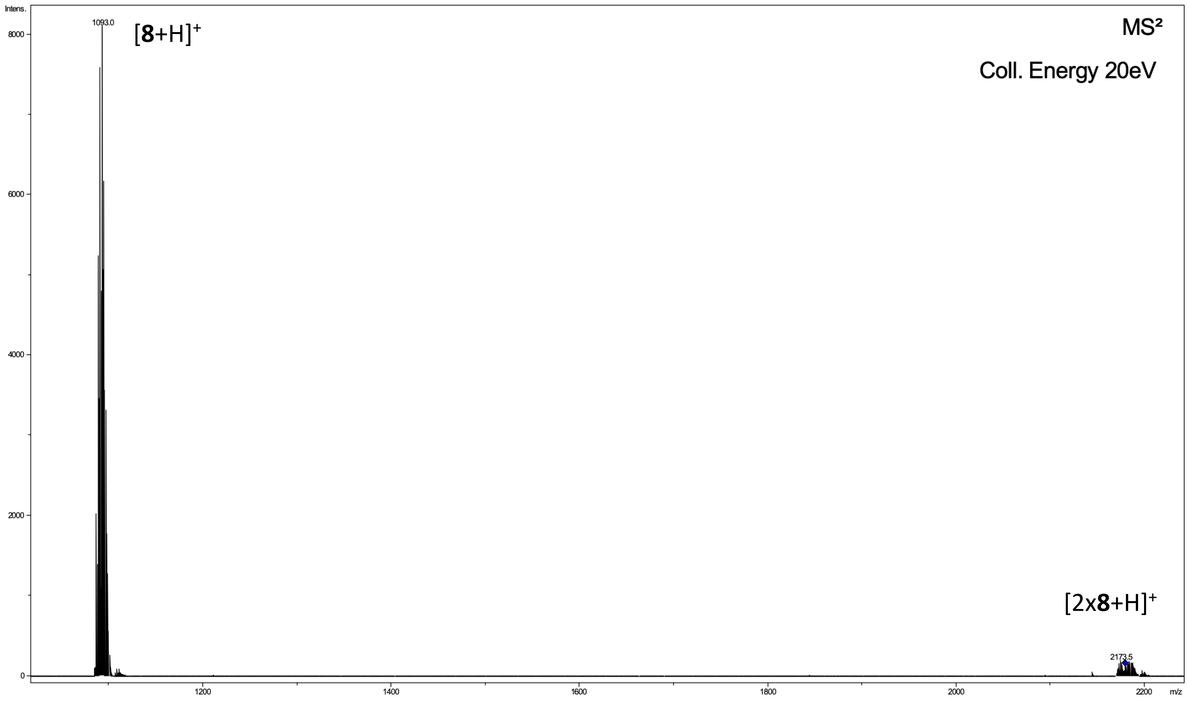

Figure S33. MS^2 of proton bridged dimer.

FT-ICR experiments with 8

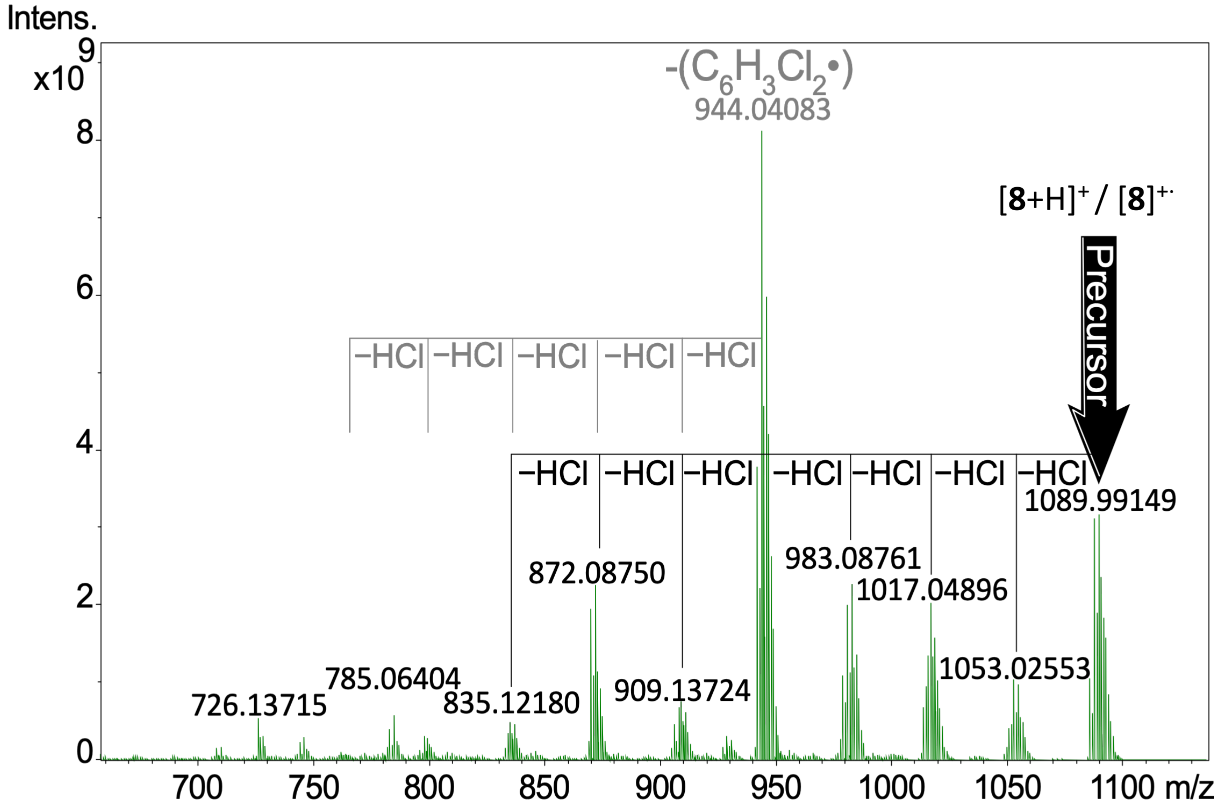

Figure S34. Overview on monomer [**8**+H]$^+$ MS^2 experiments with FT-ICR.

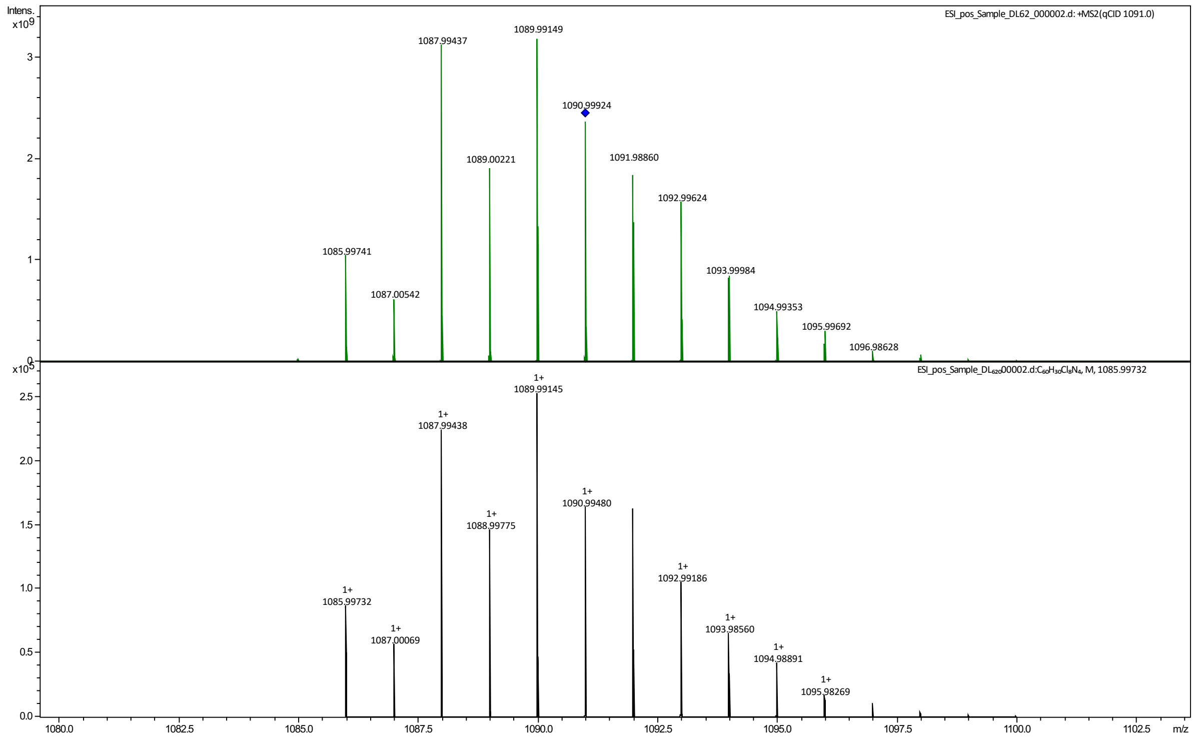

Figure S35. HRMS spectrum (top) and simulated spectrum of [**8**]$^{+\bullet}$ (bottom); protonated species is already dissociated.

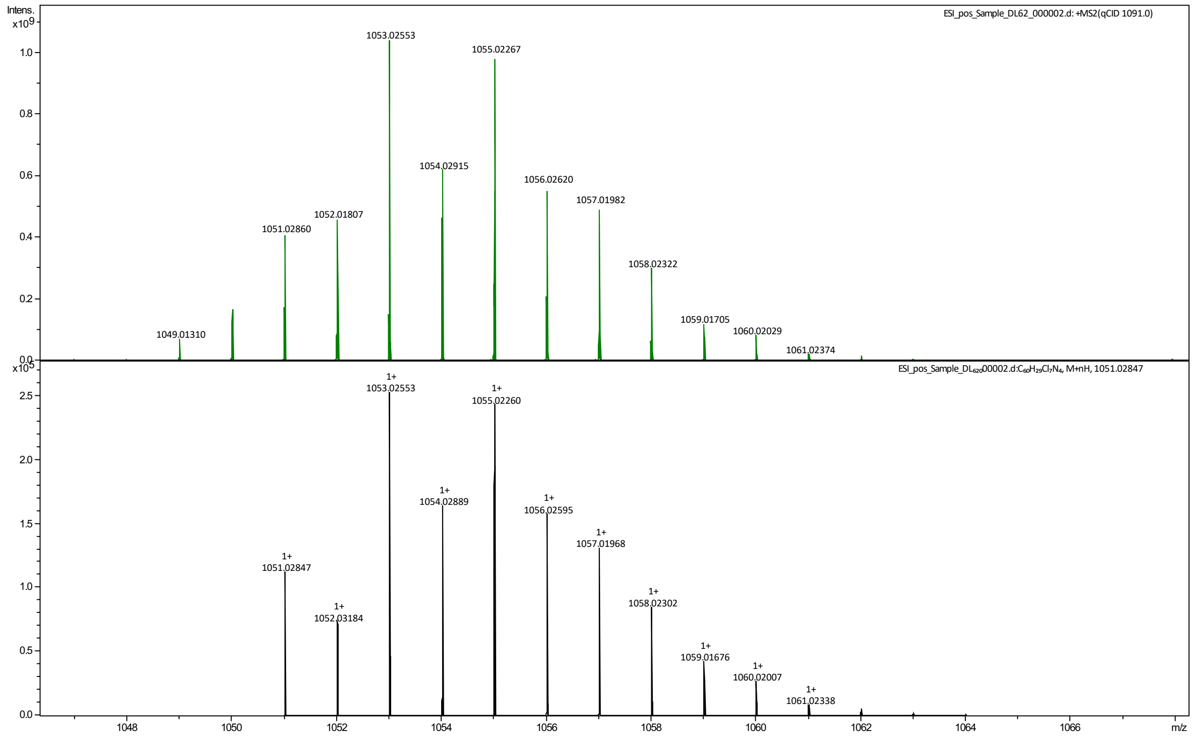

Figure S36. HRMS spectrum (top) and simulated spectrum of [**8**+H-HCl]$^+$ (bottom).

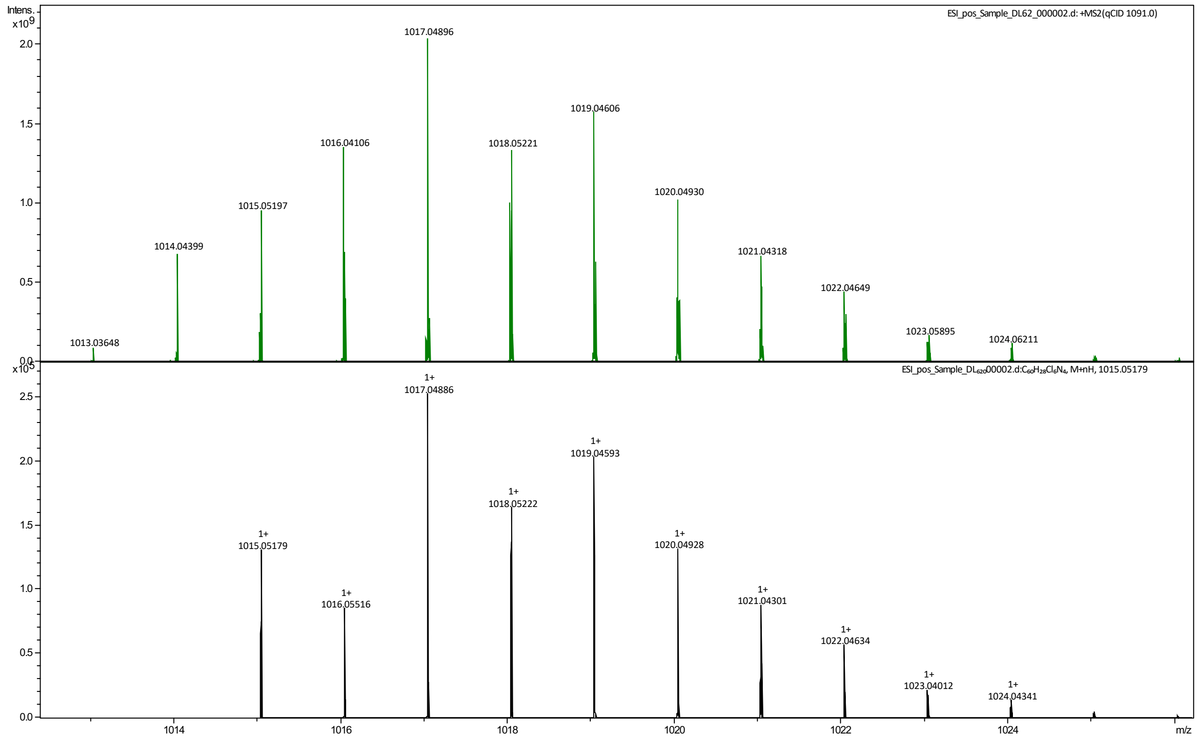

Figure S37. HRMS spectrum (top) and simulated spectrum of [**8**+H-2HCl]$^+$ (bottom).

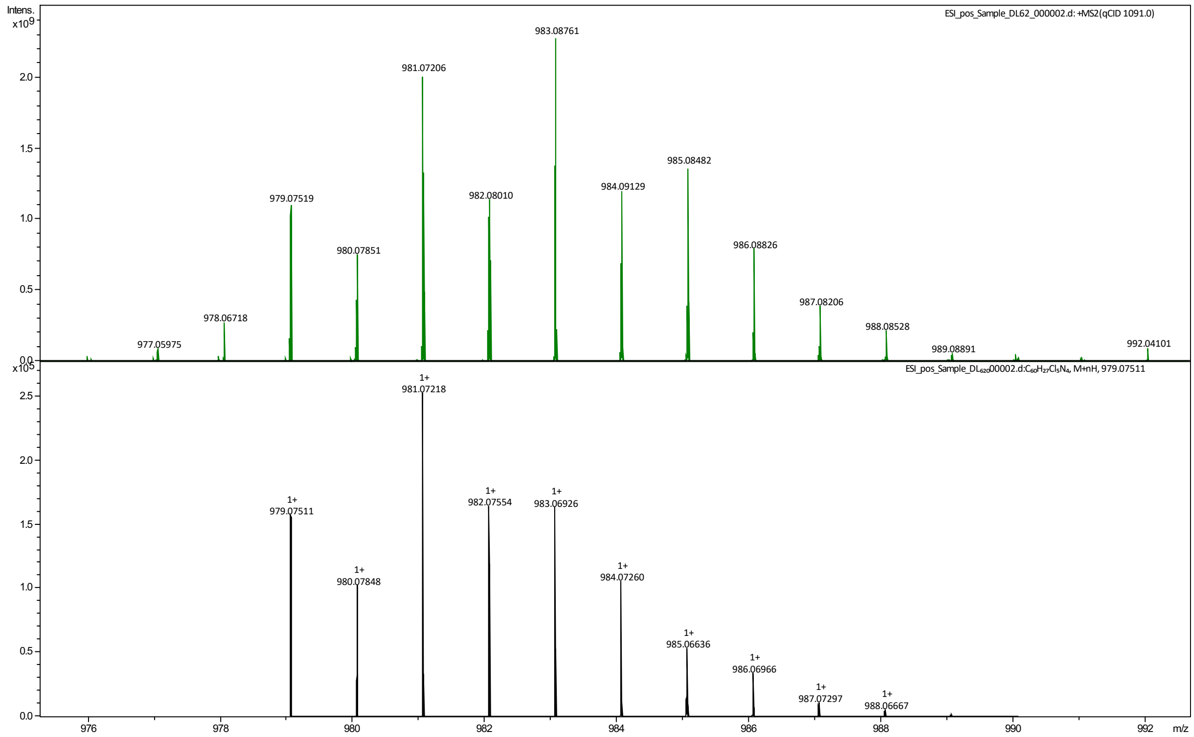

Figure S38. HRMS spectrum (top) and simulated spectrum of [**8**+H-3HCl]$^{+}$ (bottom).

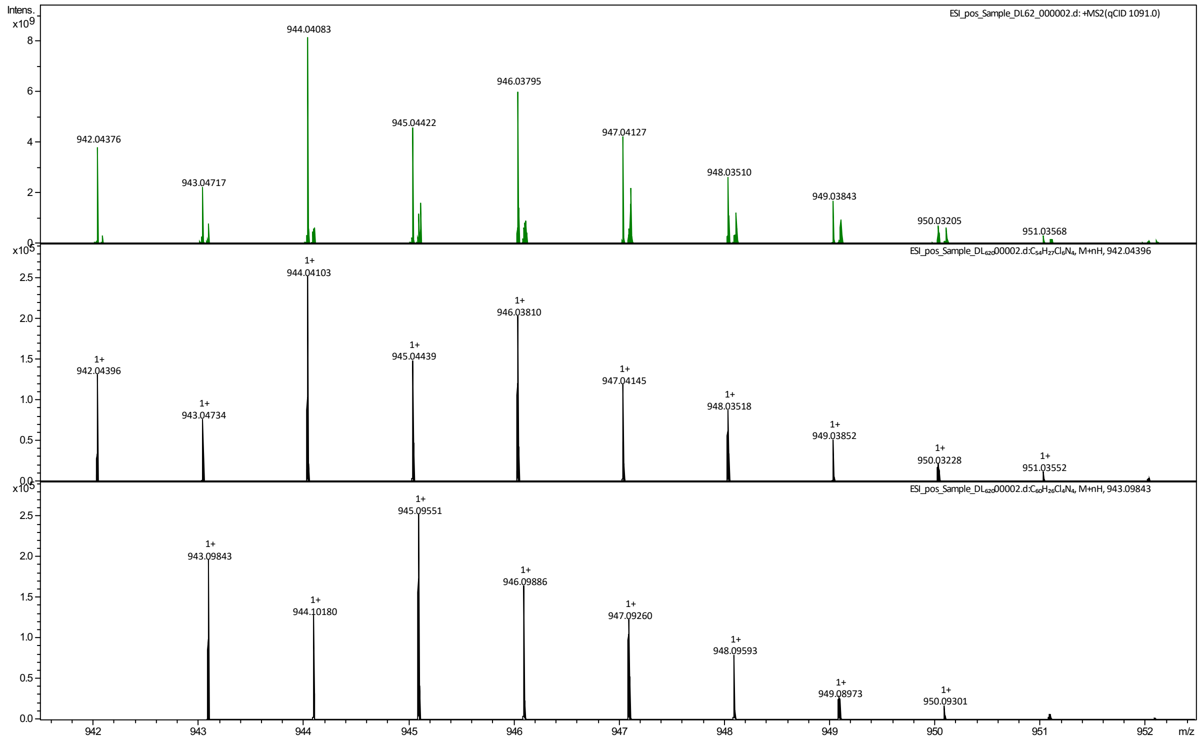

Figure S39. HRMS spectrum (top), simulated spectrum of [**8**+H-4HCl]$^{+}$ (middle), and simulated spectrum of [**8**+H-$C_6H_3Cl_2$]$^{+}$ (bottom).

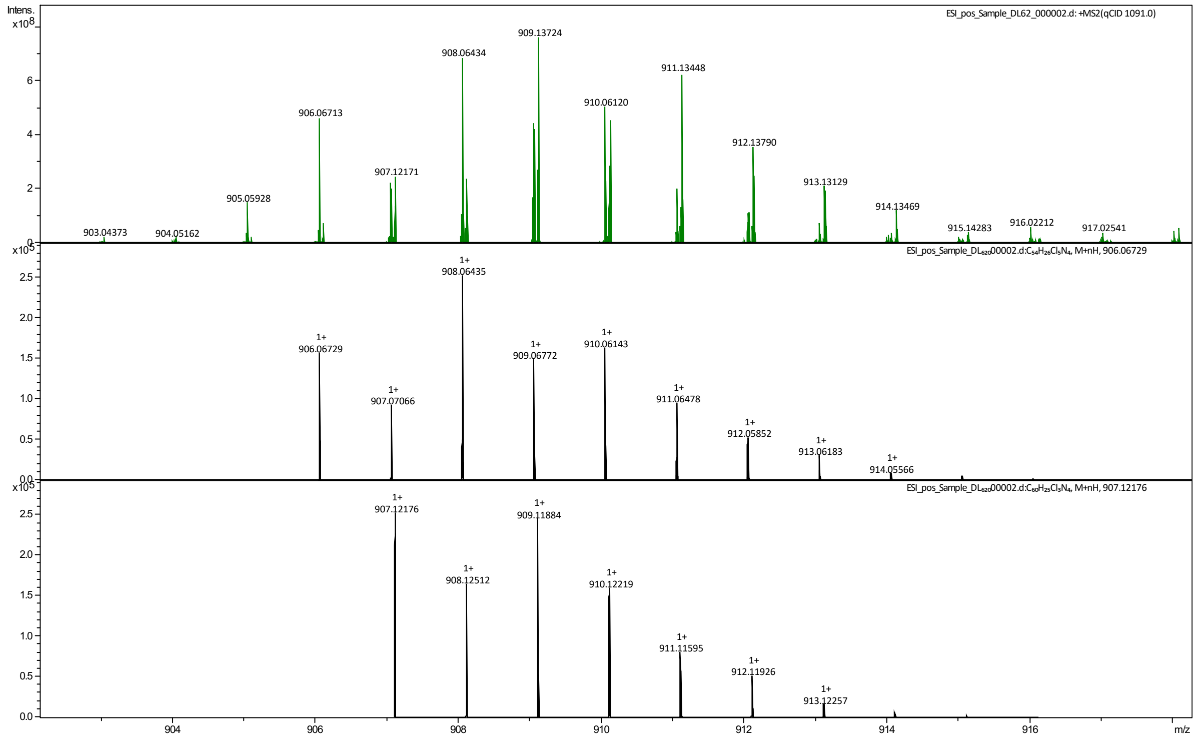

Figure S40. HRMS spectrum (top), simulated spectrum of [**8**+H-5HCl]$^+$ (middle), and simulated spectrum of [**8**+H-$C_6H_3Cl_2$-HCl]$^+$ (bottom).

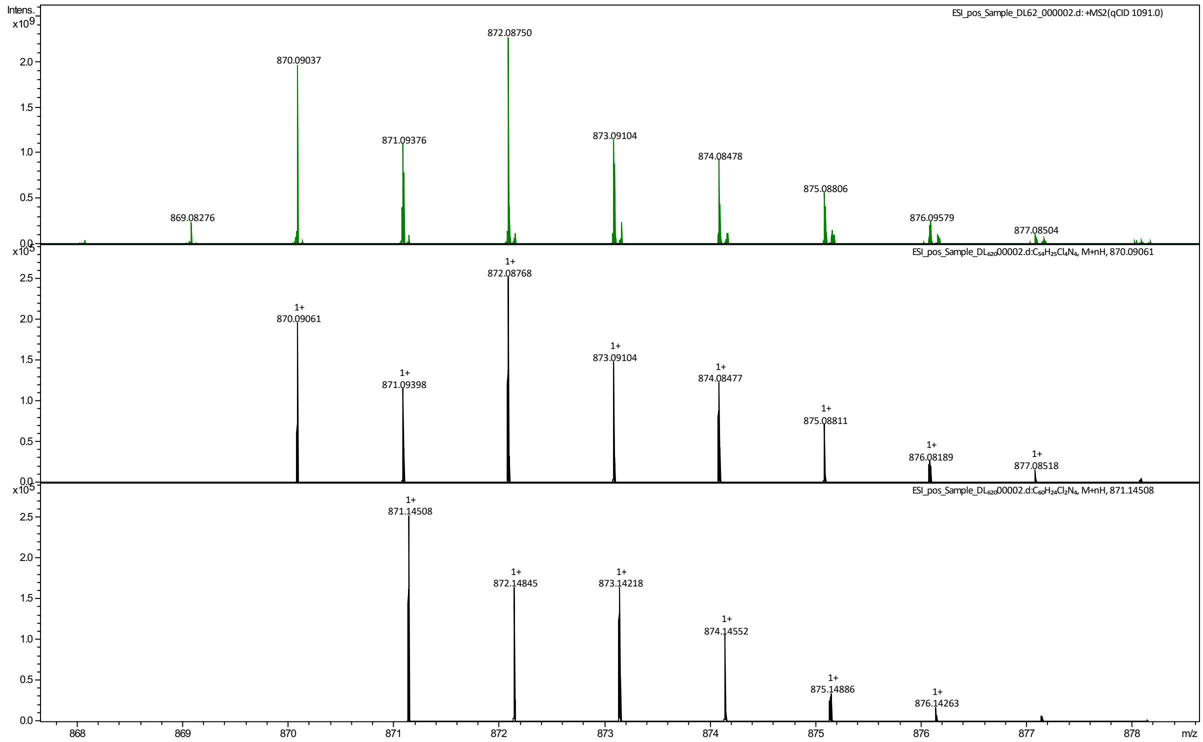

Figure S41. HRMS spectrum (top), simulated spectrum of [**8**+H-6HCl]$^+$ (middle), and simulated spectrum of [**8**+H-$C_6H_3Cl_2$-2HCl]$^+$ (bottom).

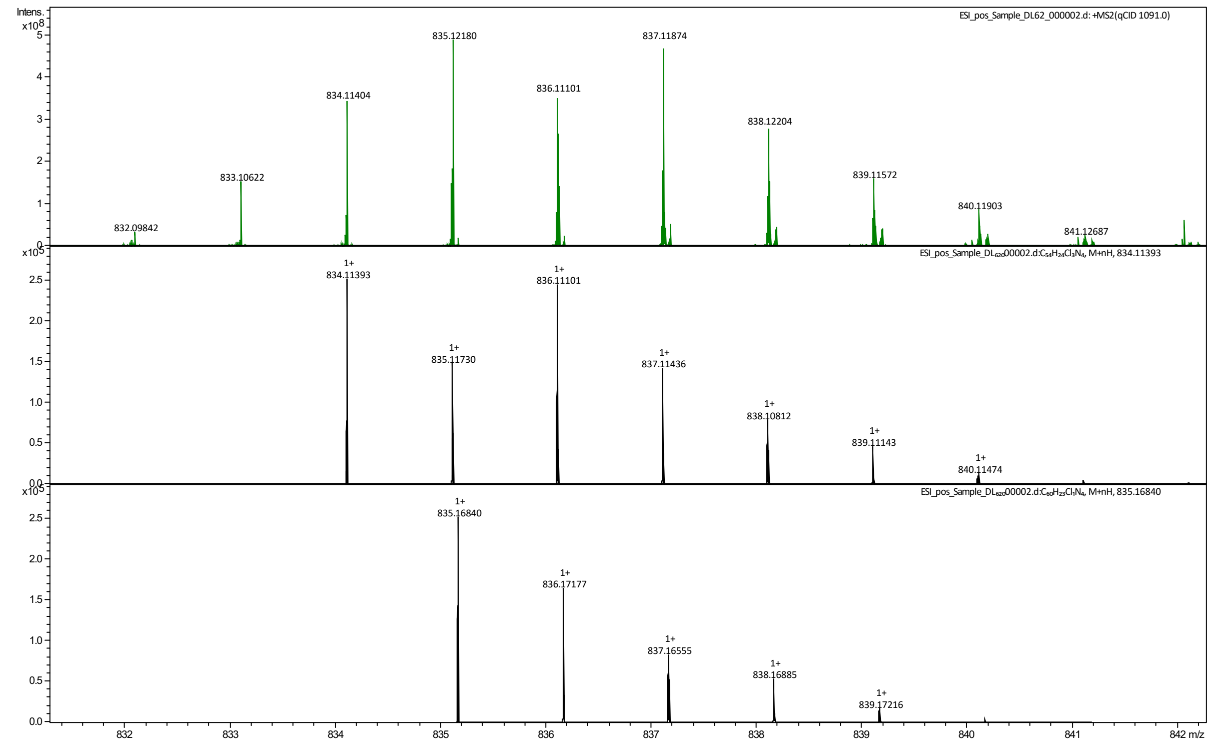

Figure S42. HRMS spectrum (top), simulated spectrum of [**8**+H-7HCl]$^+$ (middle), and simulated spectrum of [**8**+H-$C_6H_3Cl_2$-4HCl]$^+$ (bottom).

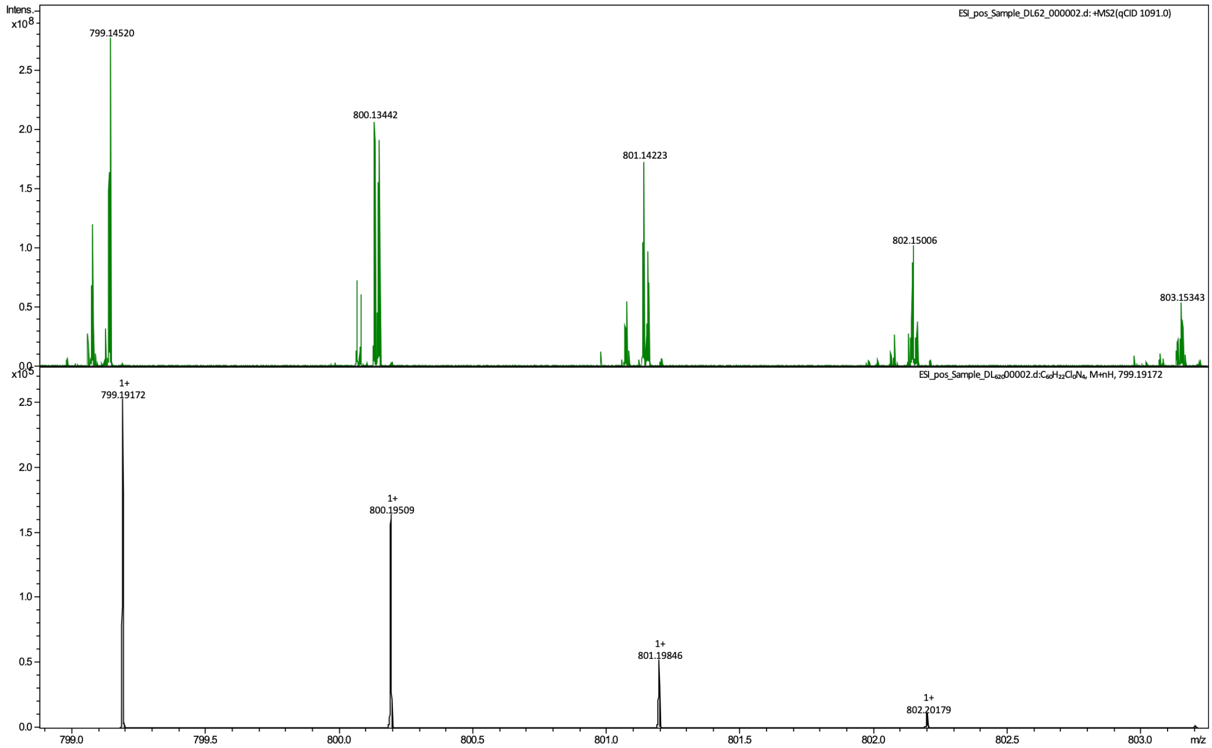

Figure S43. HRMS spectrum (top) and simulated spectrum [**8**+H-8HCl]$^+$ (bottom); no matching signal at loss of 8 HCl.

FT-ICR experiments with 8Pd

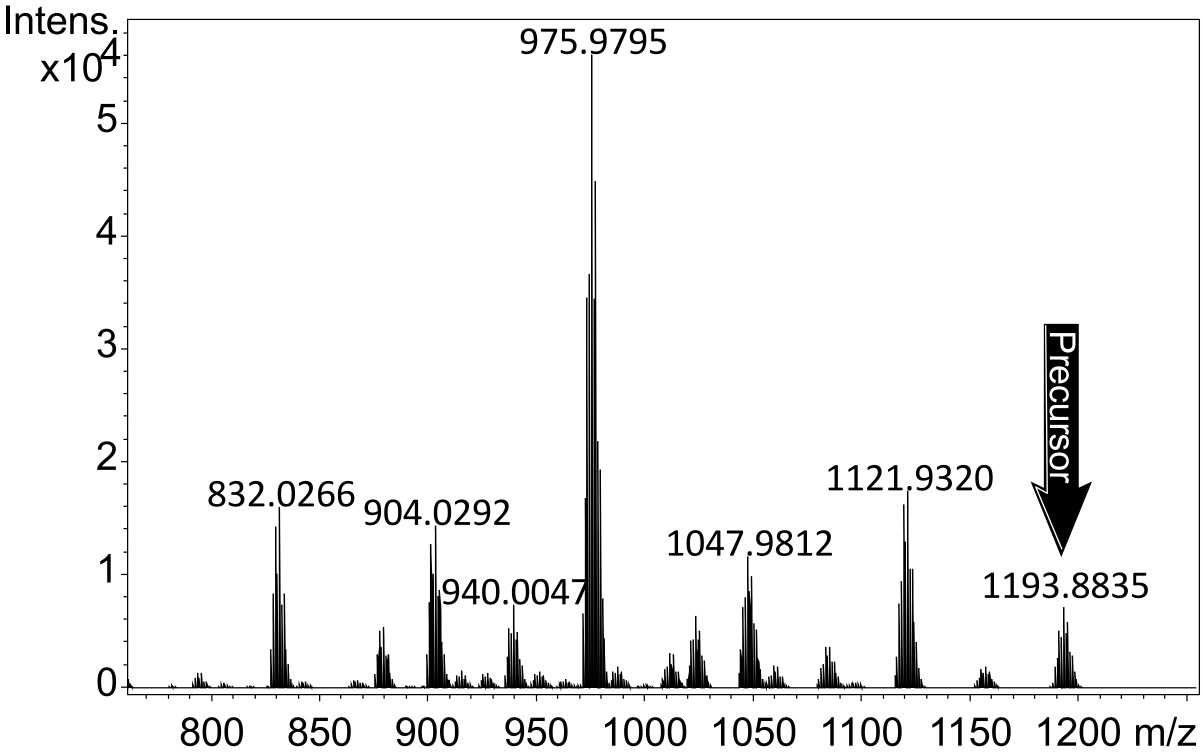

Figure S44. Overview on monomer[**8Pd**]$^{+\bullet}$ MS^2 experiments with FT-ICR.

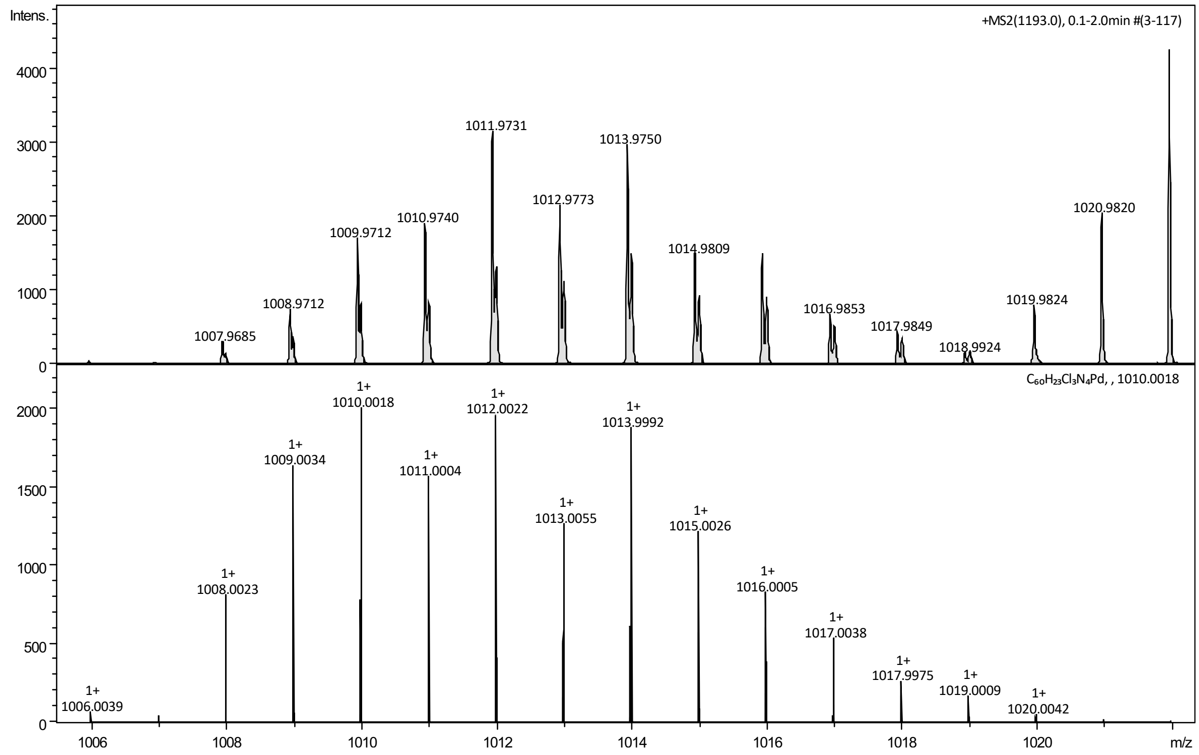

Figure S45. HRMS spectrum (top) and simulated spectrum [**8Pd**-5HCl]$^{+\bullet}$ (bottom).

2.3 Chapter S3 – Dissociation Experiments of 1, 1Co, 1Cu, 1Zn, 2, 7, and Ref

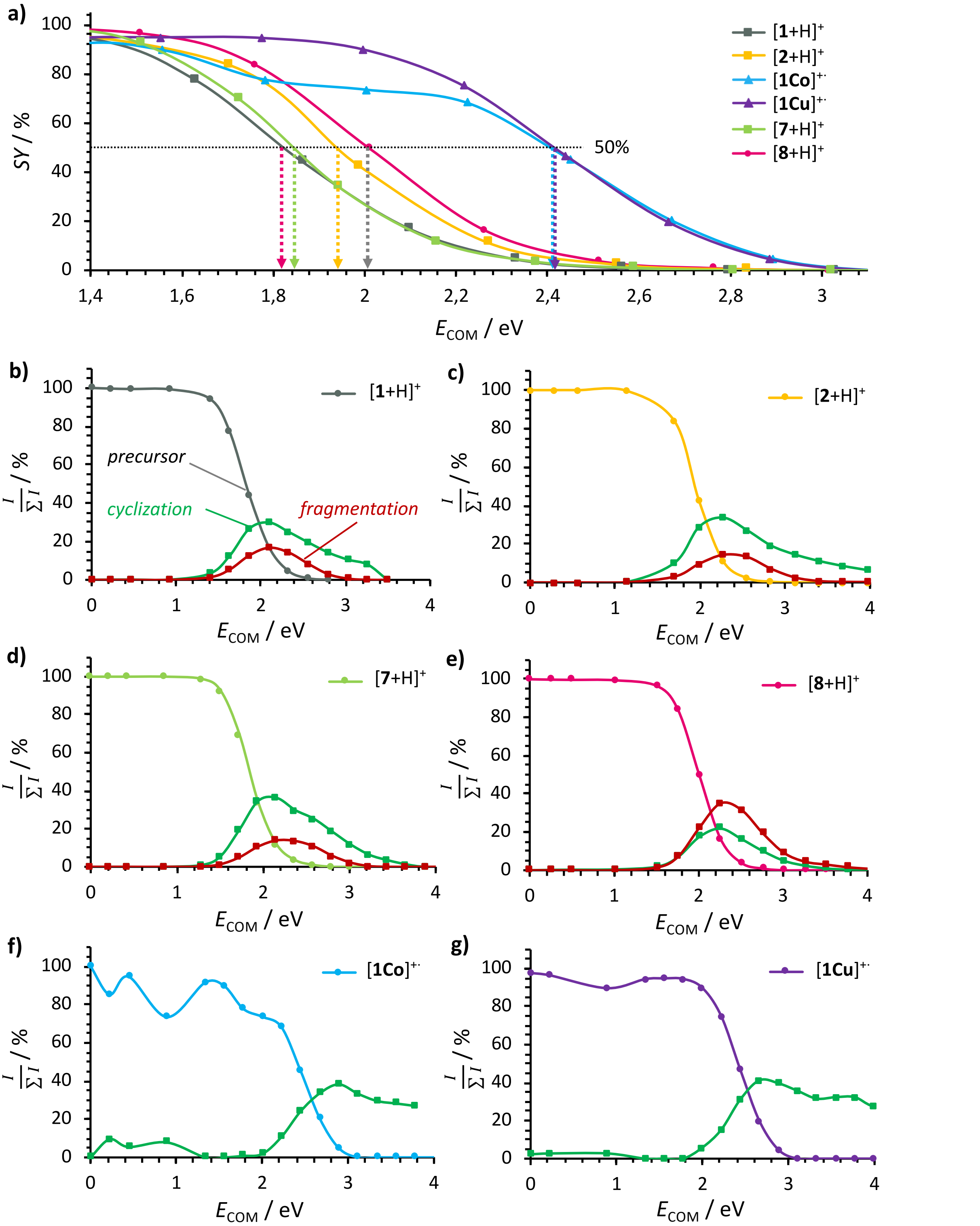

Figure S46. Energy-dependent CID breakdown graphs: a) zoom-in in the precursor ion region (survival yield, *SY*); b) [**1**+H]$^+$; c) [**2**+H]$^+$; d) [**7**+H]$^+$; e) [**8**+H]$^+$; f) [**1Co**]$^{+\bullet}$; g) [**1Cu**]$^{+\bullet}$. The precursor in b-e is depicted in black, the prodcuts of 1-8 cyclization steps in green and the side-reaction of the aryl-radical fragmentation in red; x-axis: center-of-mass collision energy (E_{COM}) and y-axis: ratio of precursor or fragment ion intensity over the sum of intensities of all signals ($I/\sum I$).

FT-ICR experiments with 2

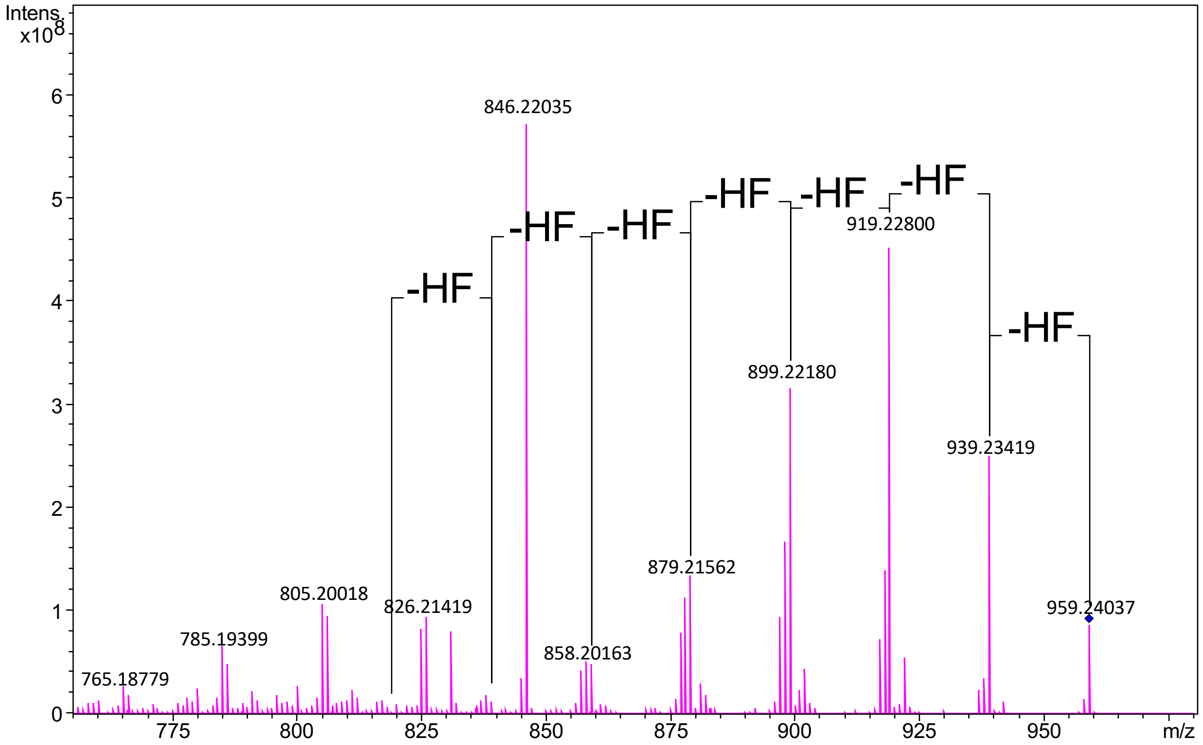

Figure S47. Overview on monomer [**2**+H]$^+$ MS^2 experiments with FT-ICR.

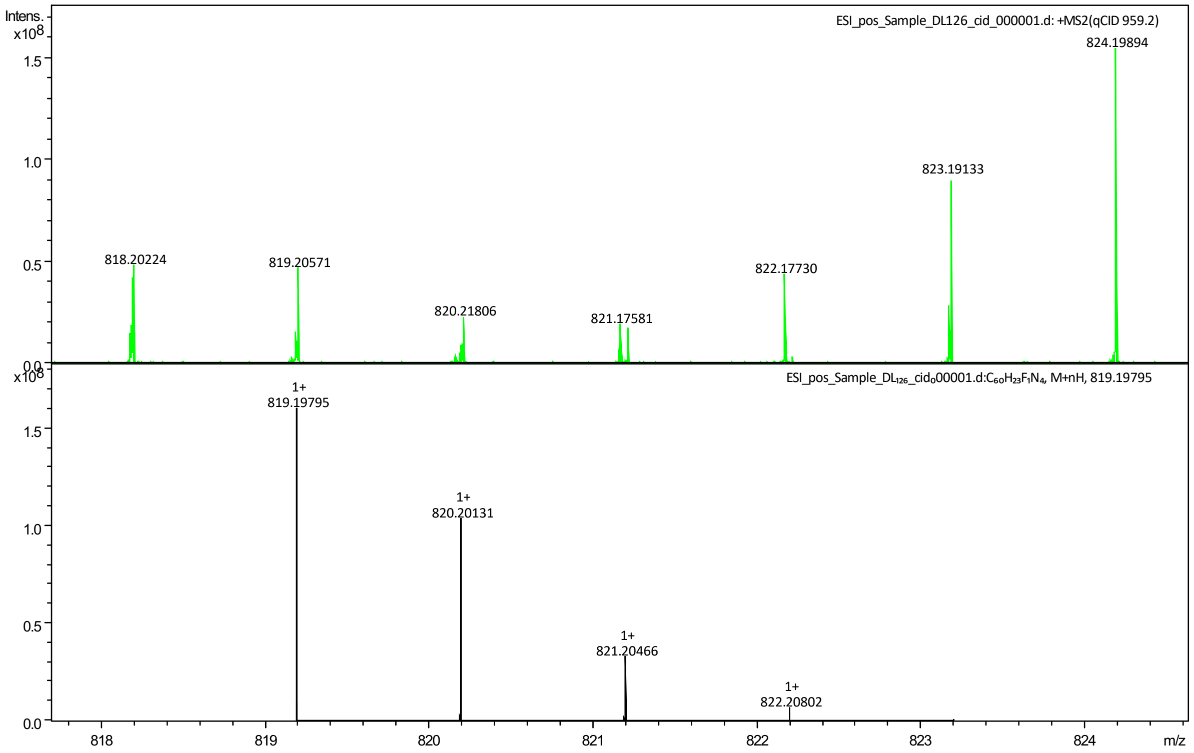

Figure S48. HRMS spectrum (top) and simulated spectrum [**2**+H-7HF]$^+$ (bottom).

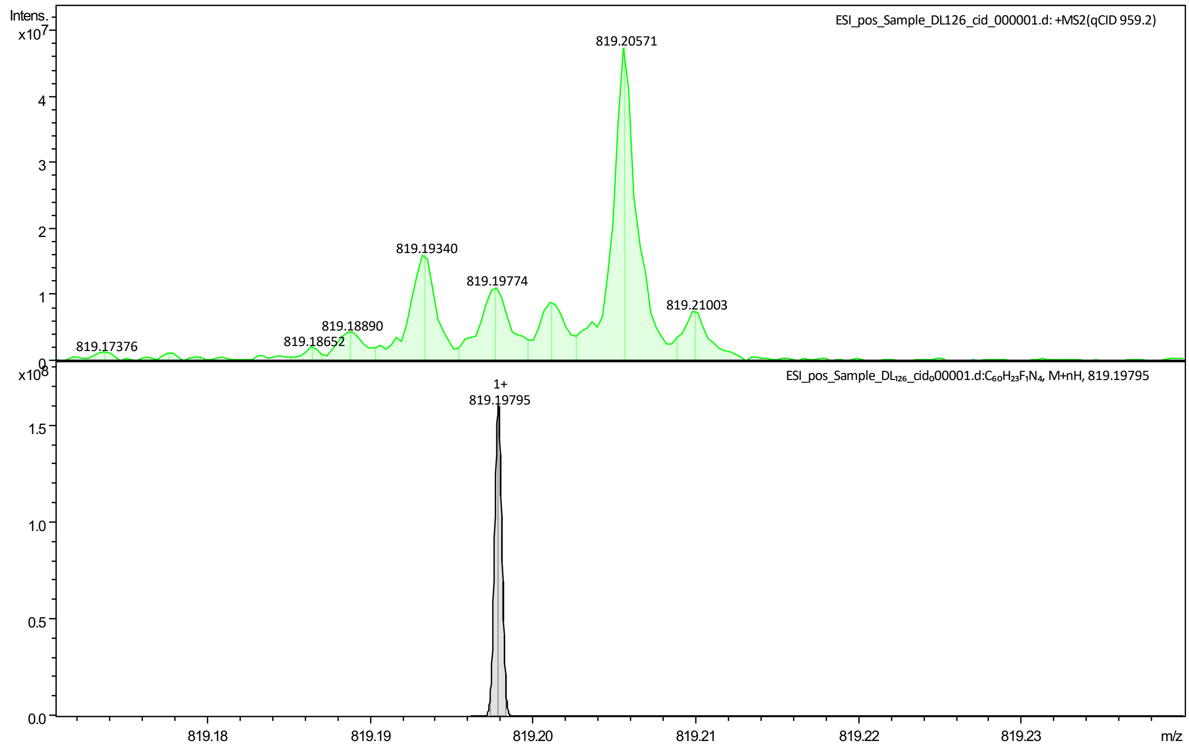

Figure S49. High resolution analysis of observed peak at 819 m/z (top) and simulation (bottom) of [**2**+H-7HF]$^{+}$.

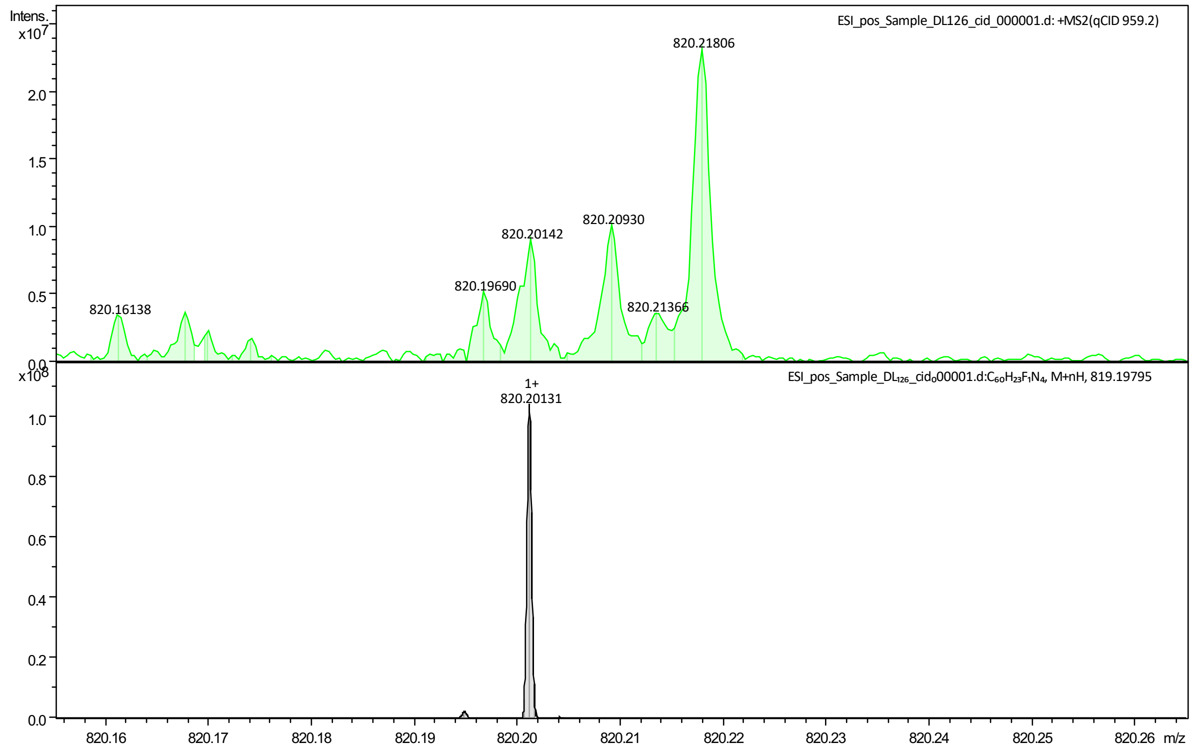

Figure S50. High resolution analysis of observed peak at 820 m/z (top) and simulation (bottom) of [**2**+H-7HF]$^{+}$.

QTOF experiments with 1

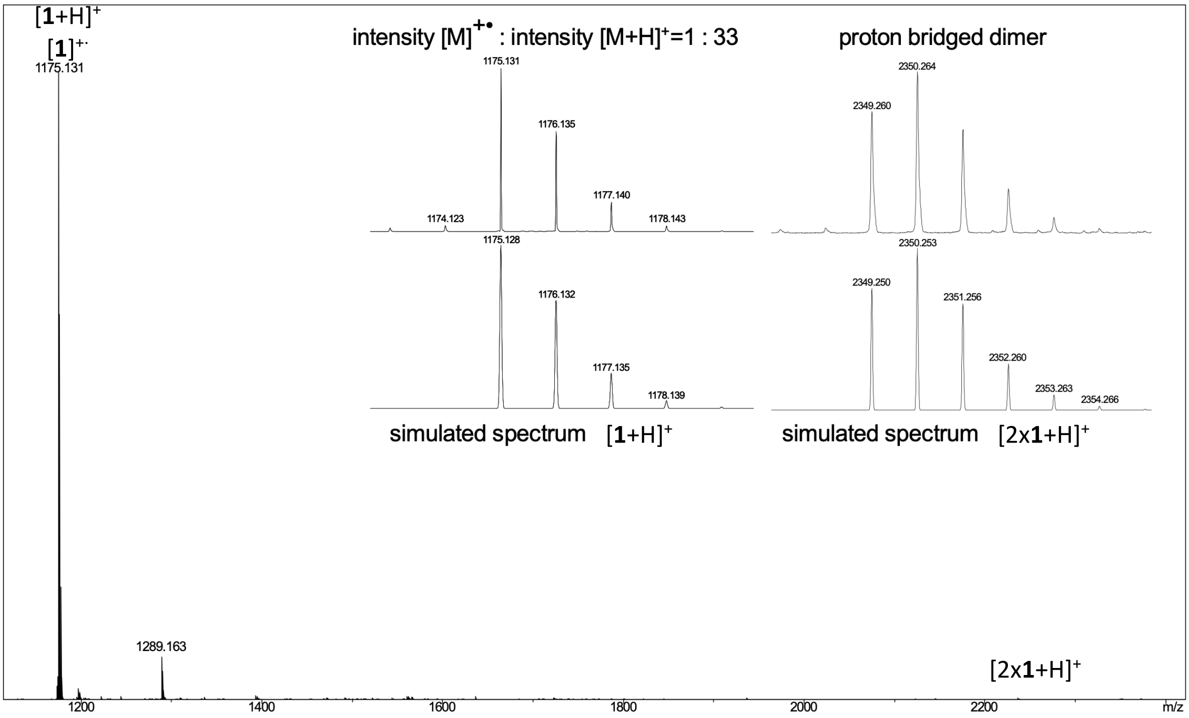

Figure S51. MS^2 of proton bridged dimer.

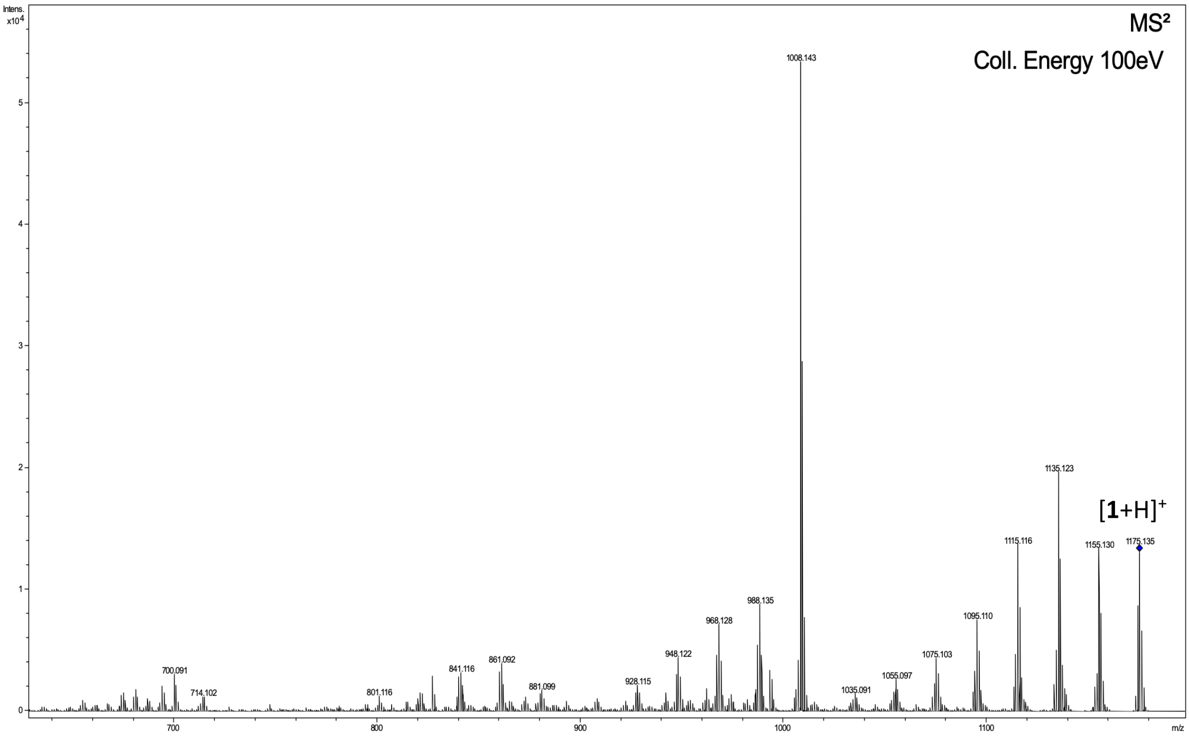

Figure S52. Overview on monomer [**1**+H]+ MS^2 experiments with QTOF.

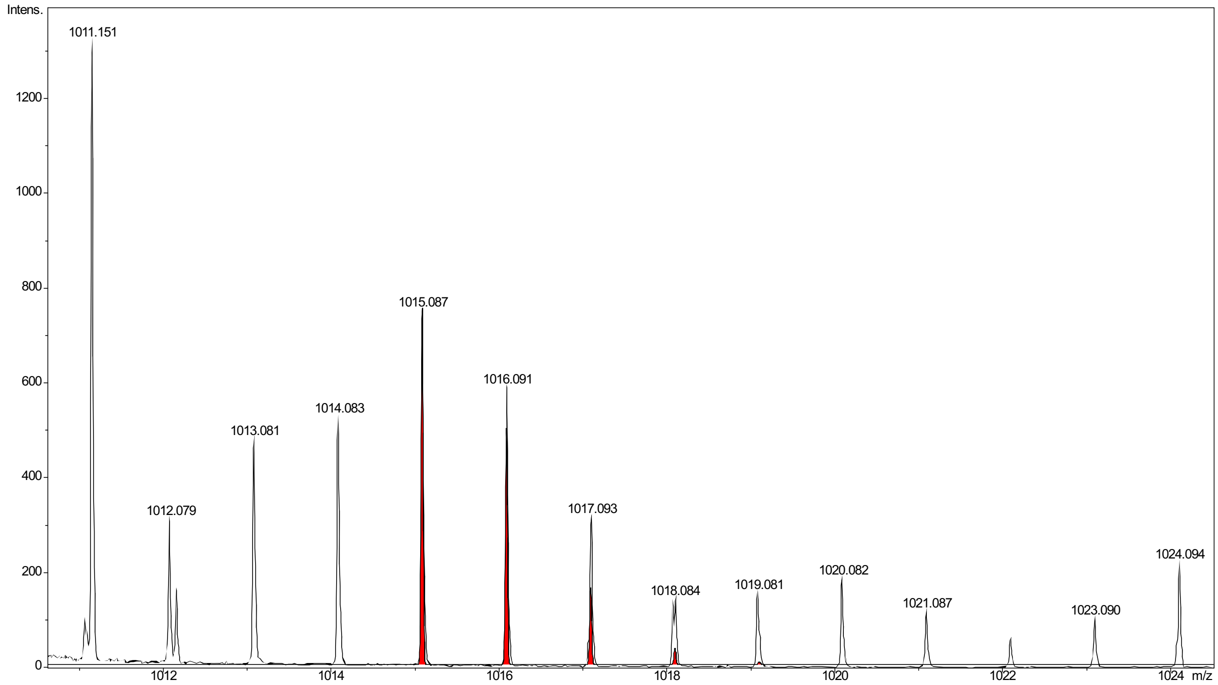

Figure S53. HRMS spectrum (black), and simulated spectrum [**1**+H-8HF]$^+$ (red).

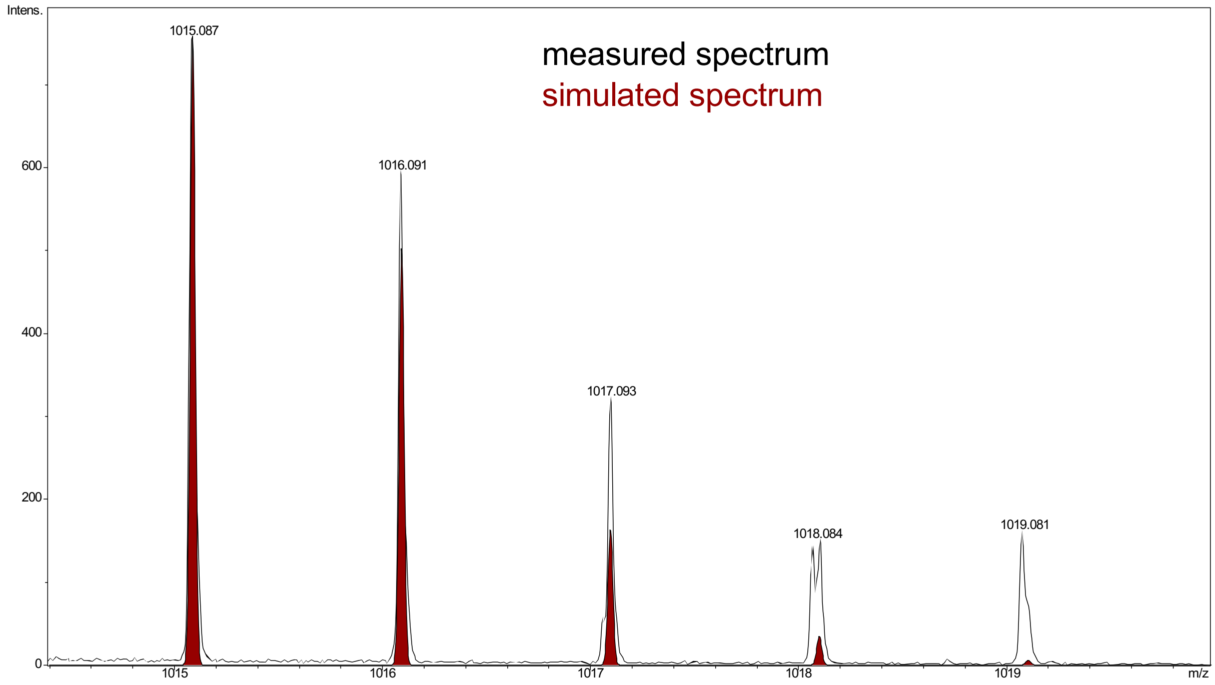

Figure S54. Zoom-in HRMS spectrum (black), and simulated spectrum [**1**+H-8HF]$^+$ (red).

FT-ICR experiments with 1

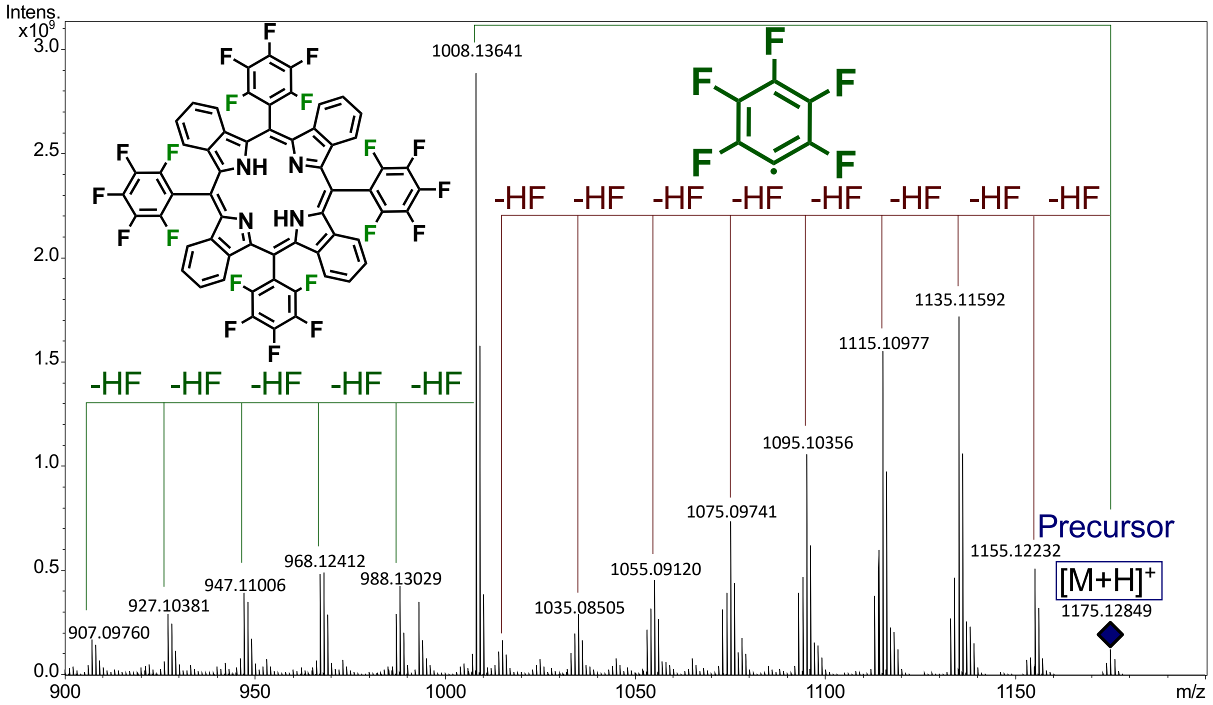

Figure S55. Overview on monomer [**1**+H]$^+$ MS^2 experiments with FT-ICR.

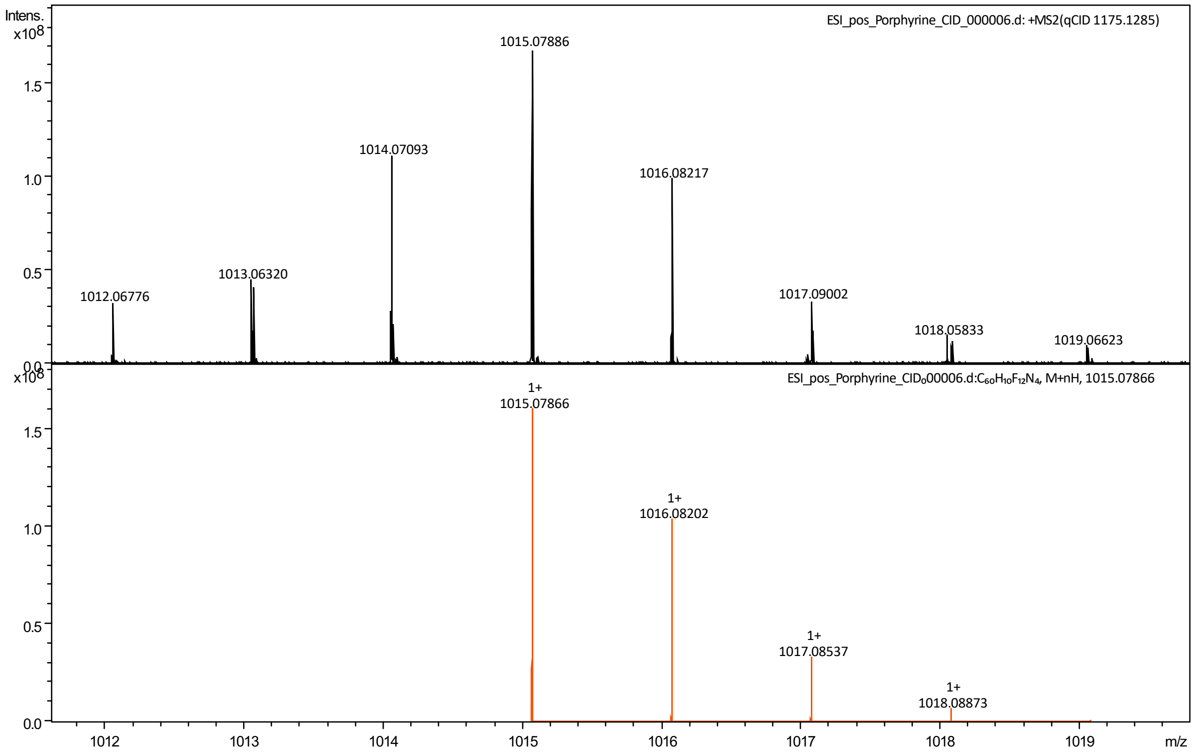

Figure S56. HRMS spectrum (top), and simulated spectrum [**1**+H-8HF]$^+$ (bottom).

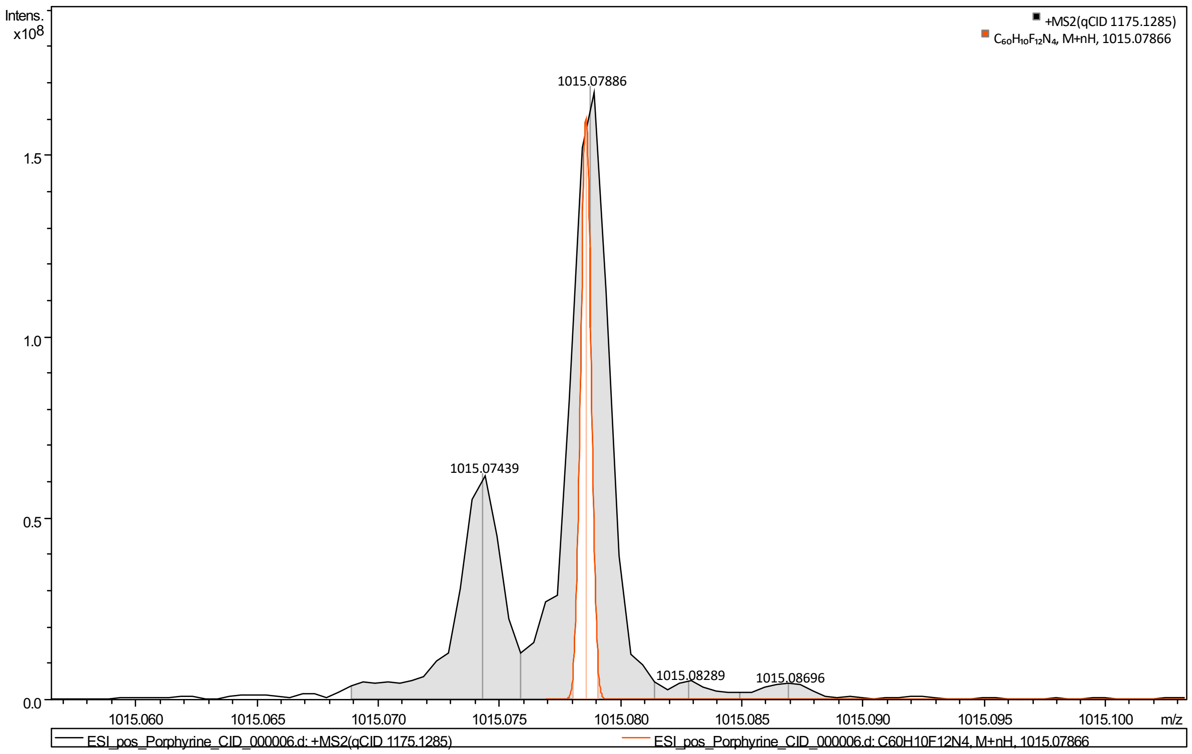

Figure S57. High resolution analysis of observed peak at 1015 m/z and simulation (red) of [**1**+H-8HF]$^{+}$.

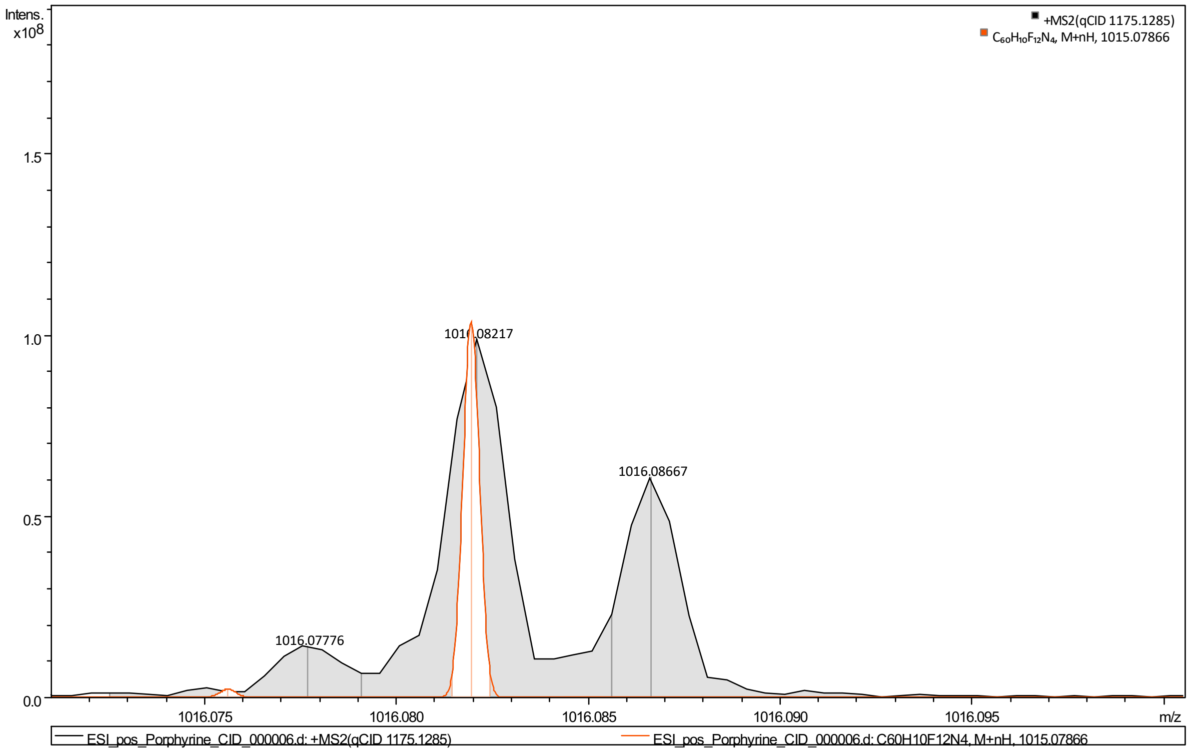

Figure S58. High resolution analysis of observed peak at 1016 m/z and simulation (red) of [**1**+H-8HF]$^{+}$.

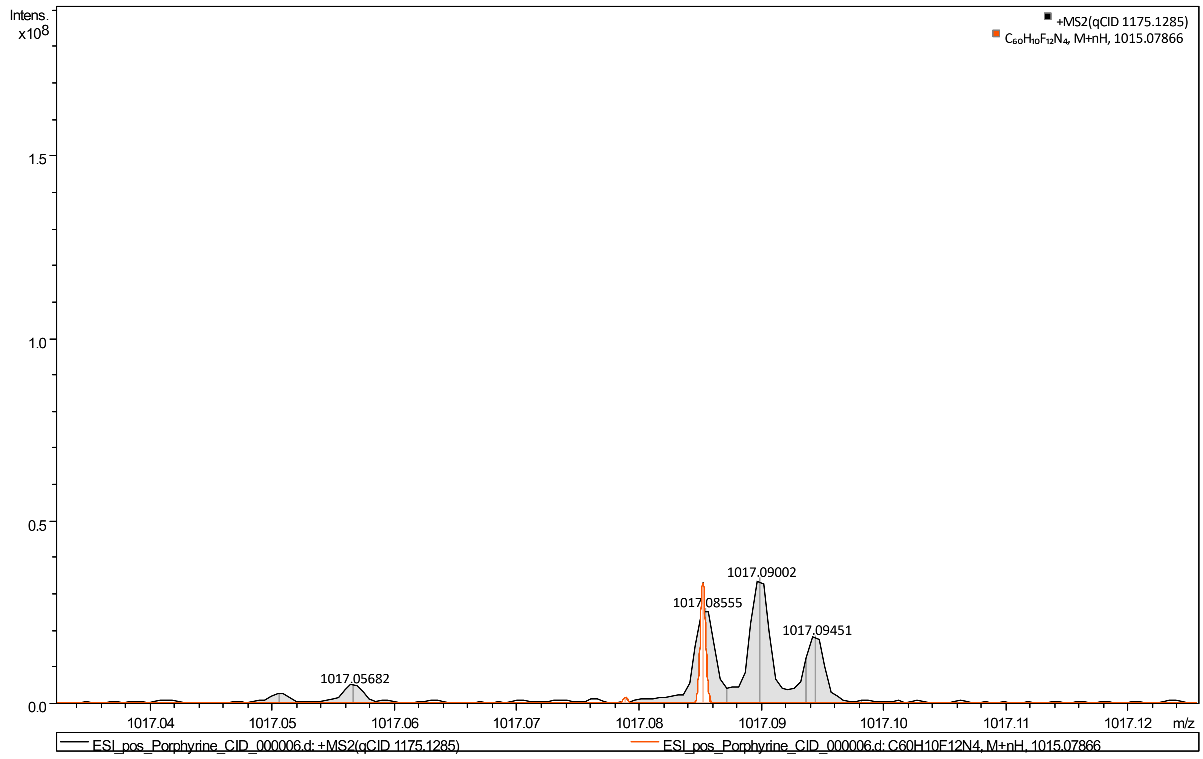

Figure S59. High resolution analysis of observed peak at 1017 m/z and simulation (red) of [**1**+H-8HF]$^{+}$.

QTOF experiments with 1Co

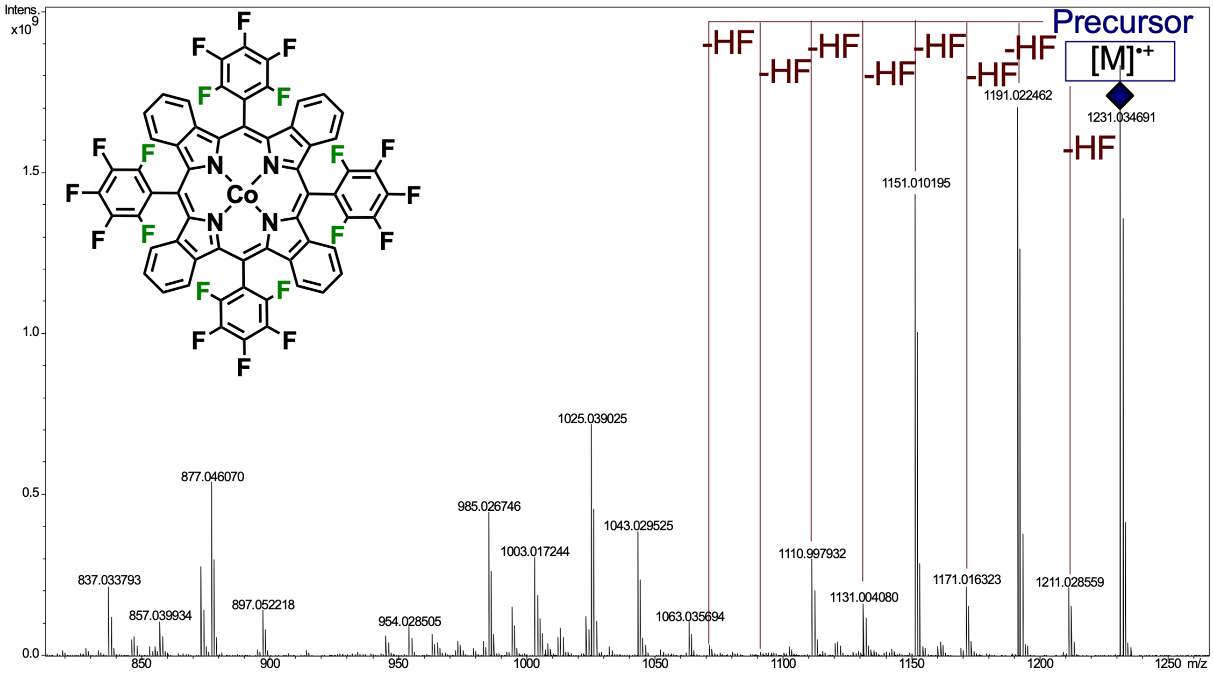

Figure S60. Overview on monomer [**1Co**]$^{+\bullet}$ MS^{2} experiments with QTOF.

FT-ICR experiments with 1Co

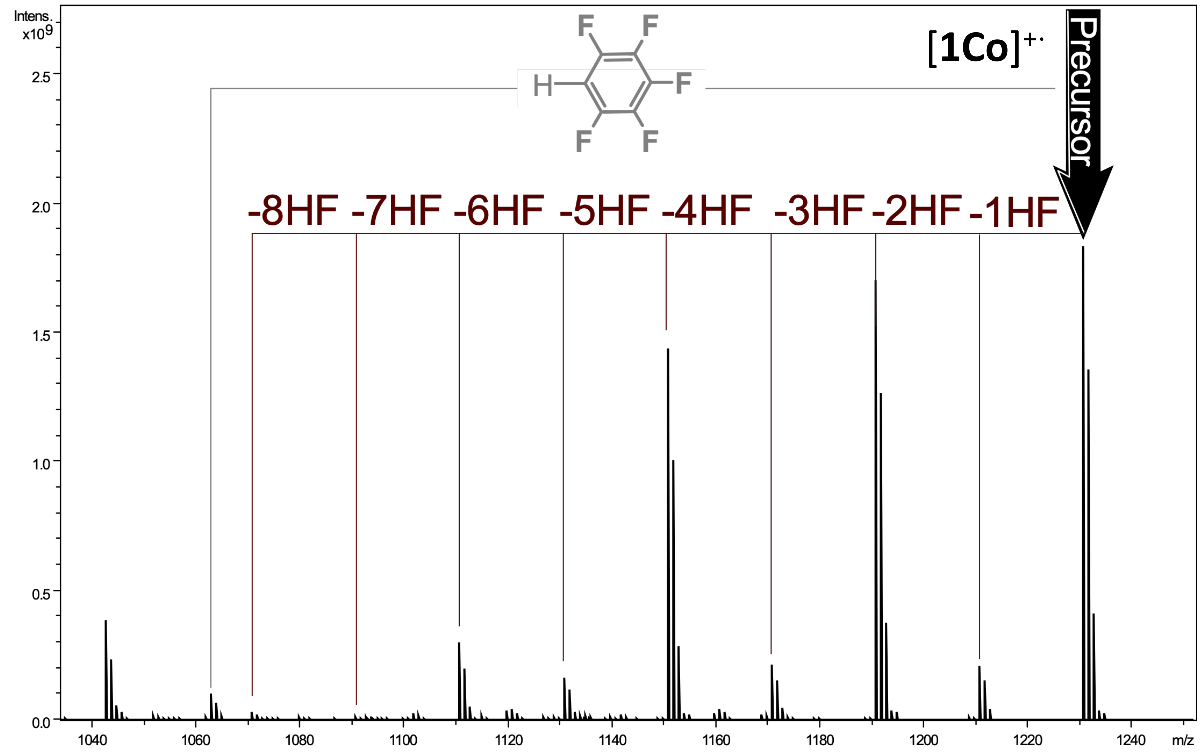

Figure S61. Overview on monomer [**1Co**]$^{+\bullet}$ MS^2 experiments with FT-ICR.

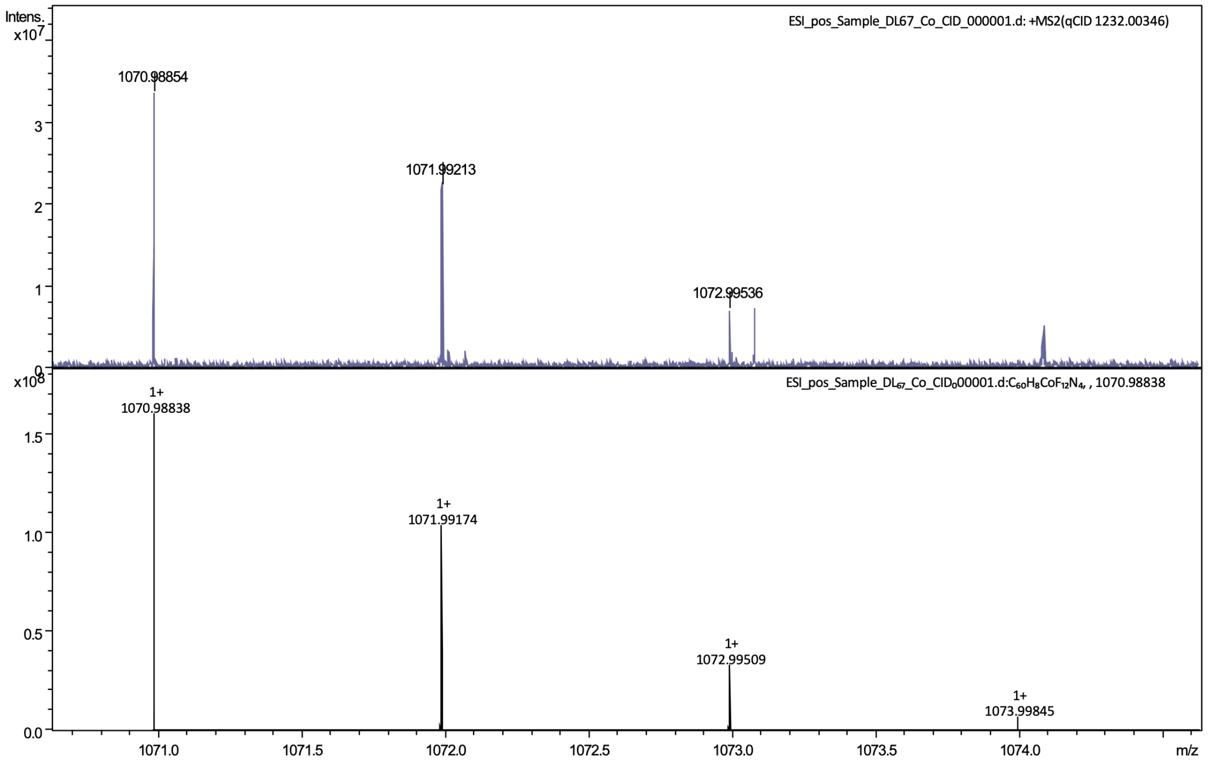

Figure S62. HRMS spectrum (top), and simulated spectrum [**1Co**-8HF]$^{+\bullet}$ (bottom).

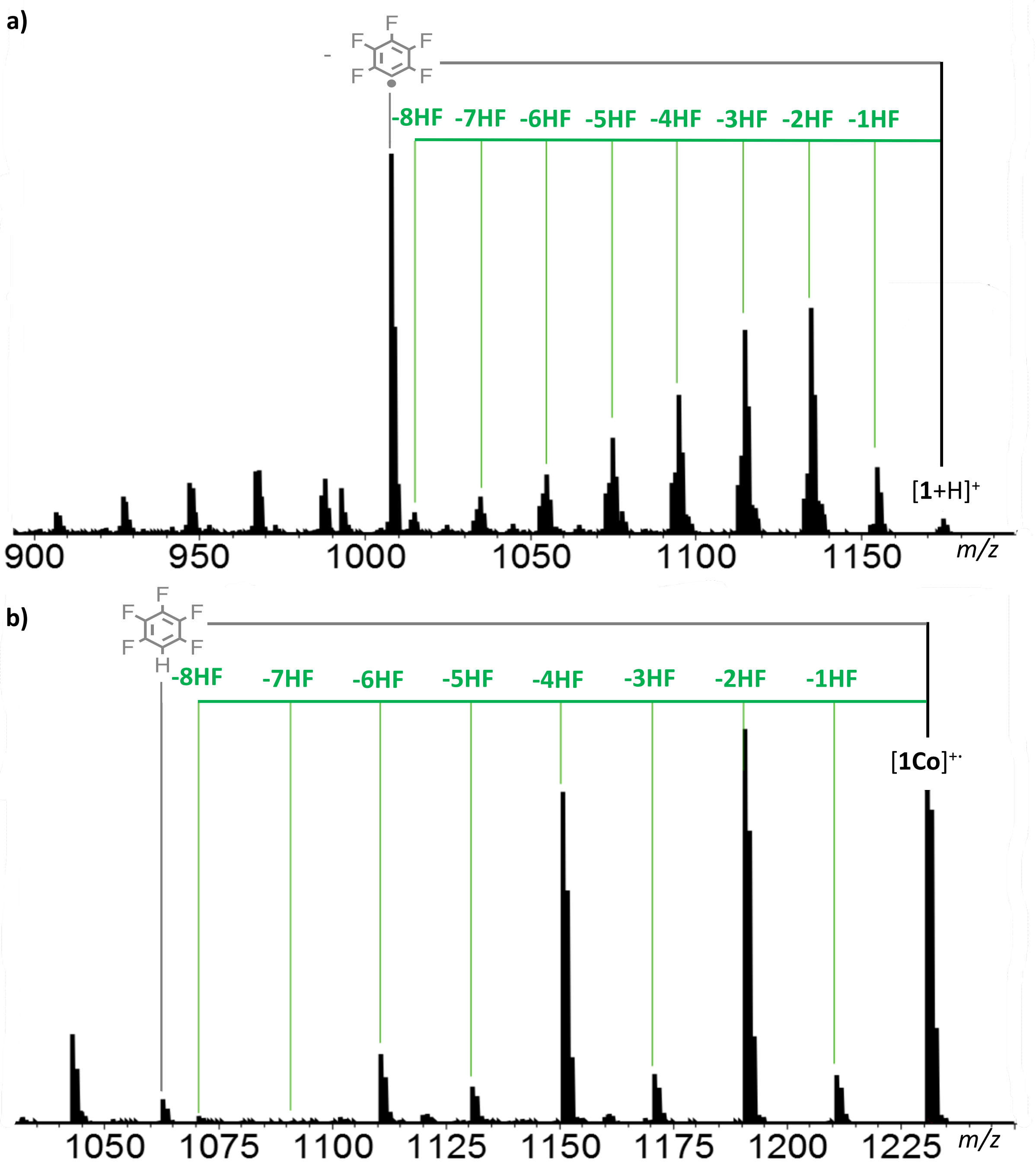

Figure S63. Enlarged comparative overview on MS^2 experiments of monomer $[\mathbf{1}+H]^+$ (top) and $[\mathbf{1Co}]^{+\bullet}$ (bottom) with FT-ICR.

QTOF and FT-ICR experiments with 1Cu and 1Zn

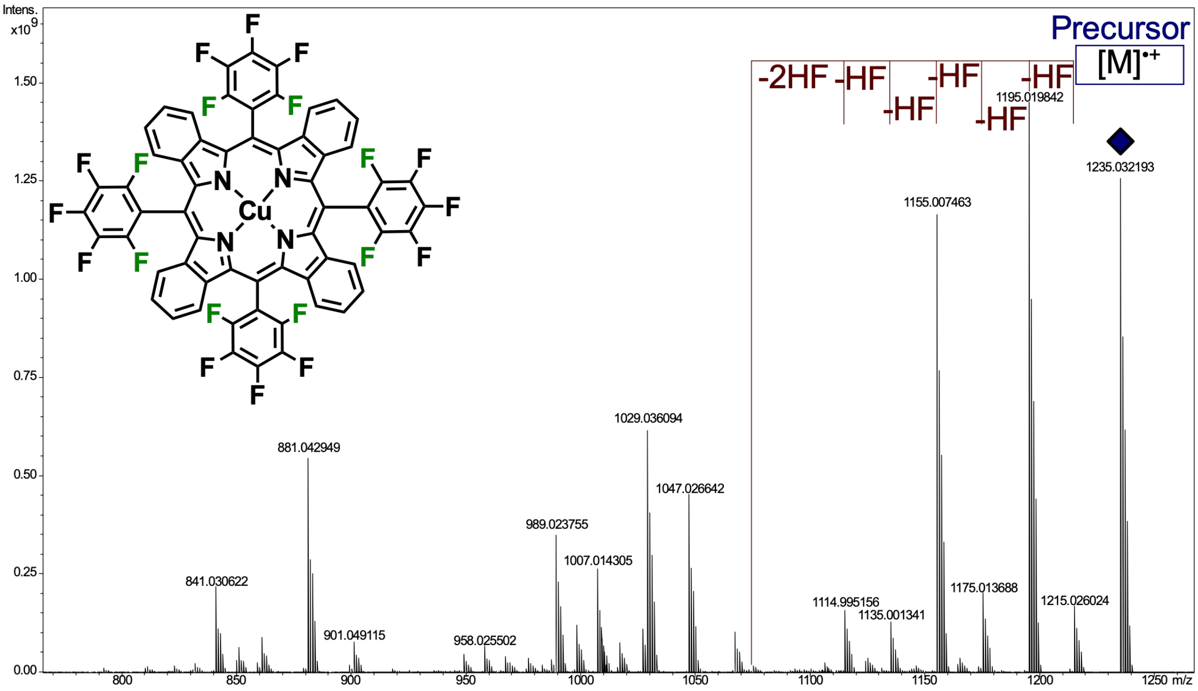

Figure S64. Overview on monomer [**1Cu**]$^{+\bullet}$ MS^2 experiments with QTOF.

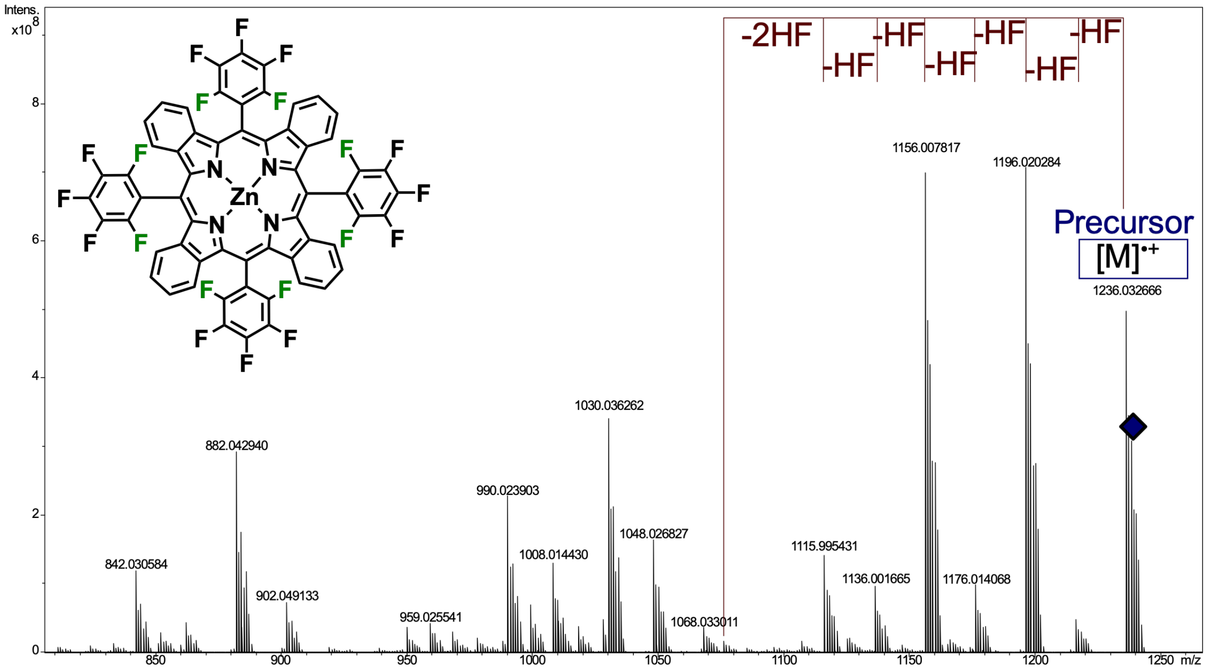

Figure S65. Overview on monomer [**1Zn**]$^{+\bullet}$ MS^2 experiments with QTOF.

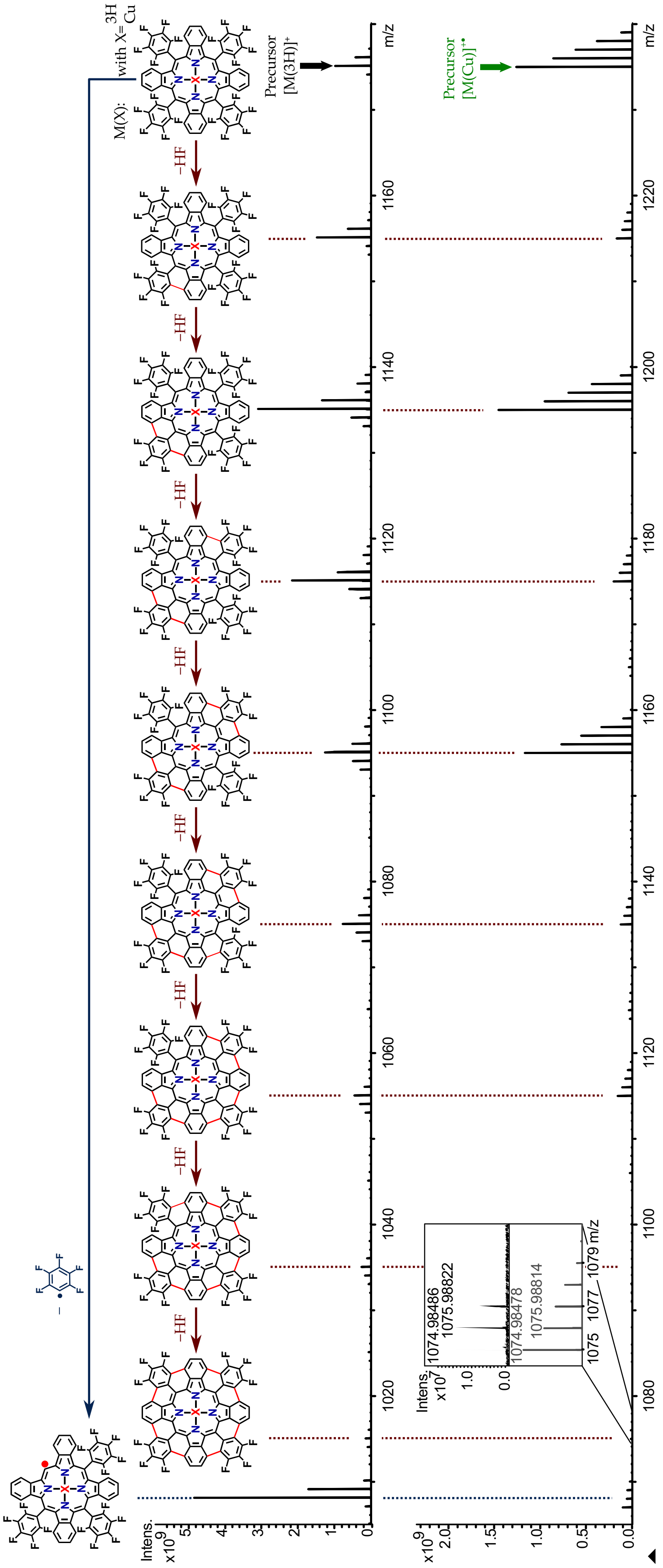

Figure S66. Enlarged comparative overview on MS^2 experiments of monomer [**1**+H]$^+$ (left) and [**1Cu**]$^{+\bullet}$ (right) with FT-ICR.

QTOF Experiments with 7

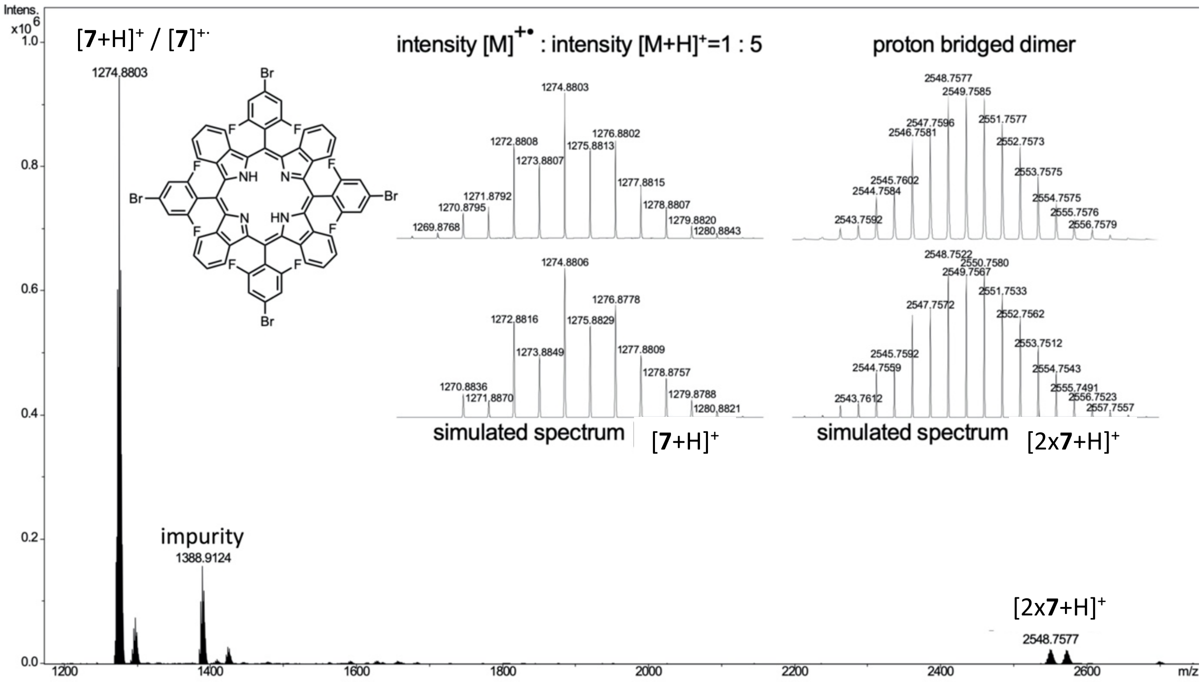

Figure S67. Analysis of precursor ion nature.

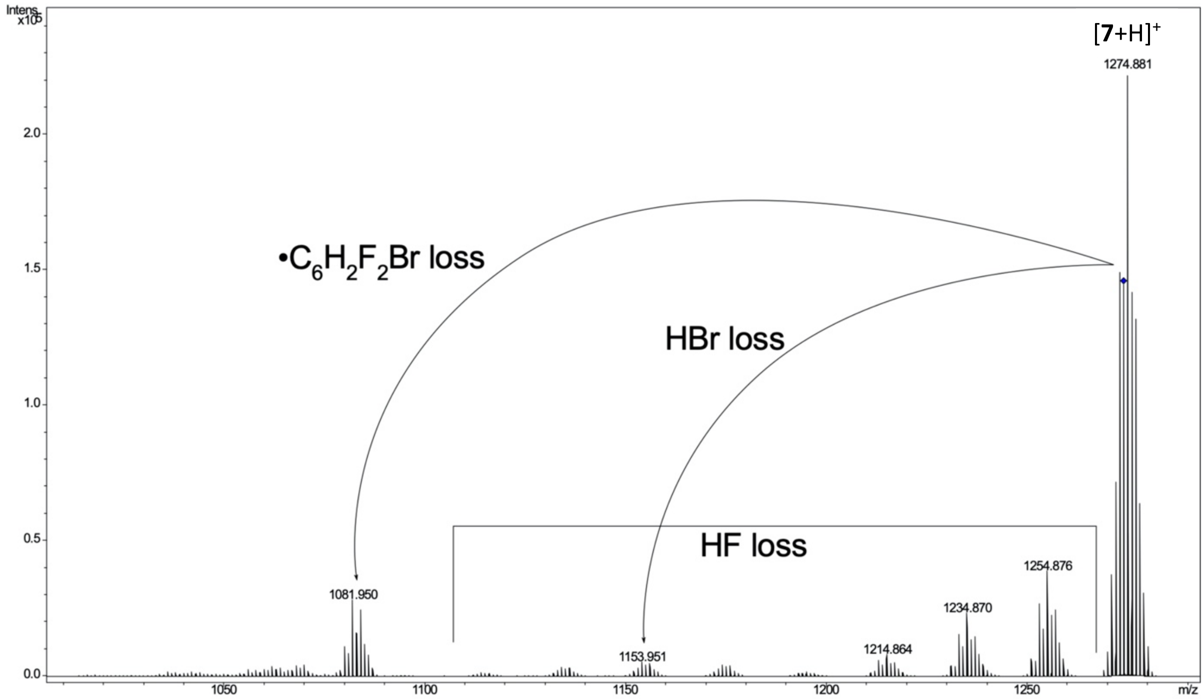

Figure S68. Overview on monomer and [7+H]+ MS^2 experiments with QTOF.

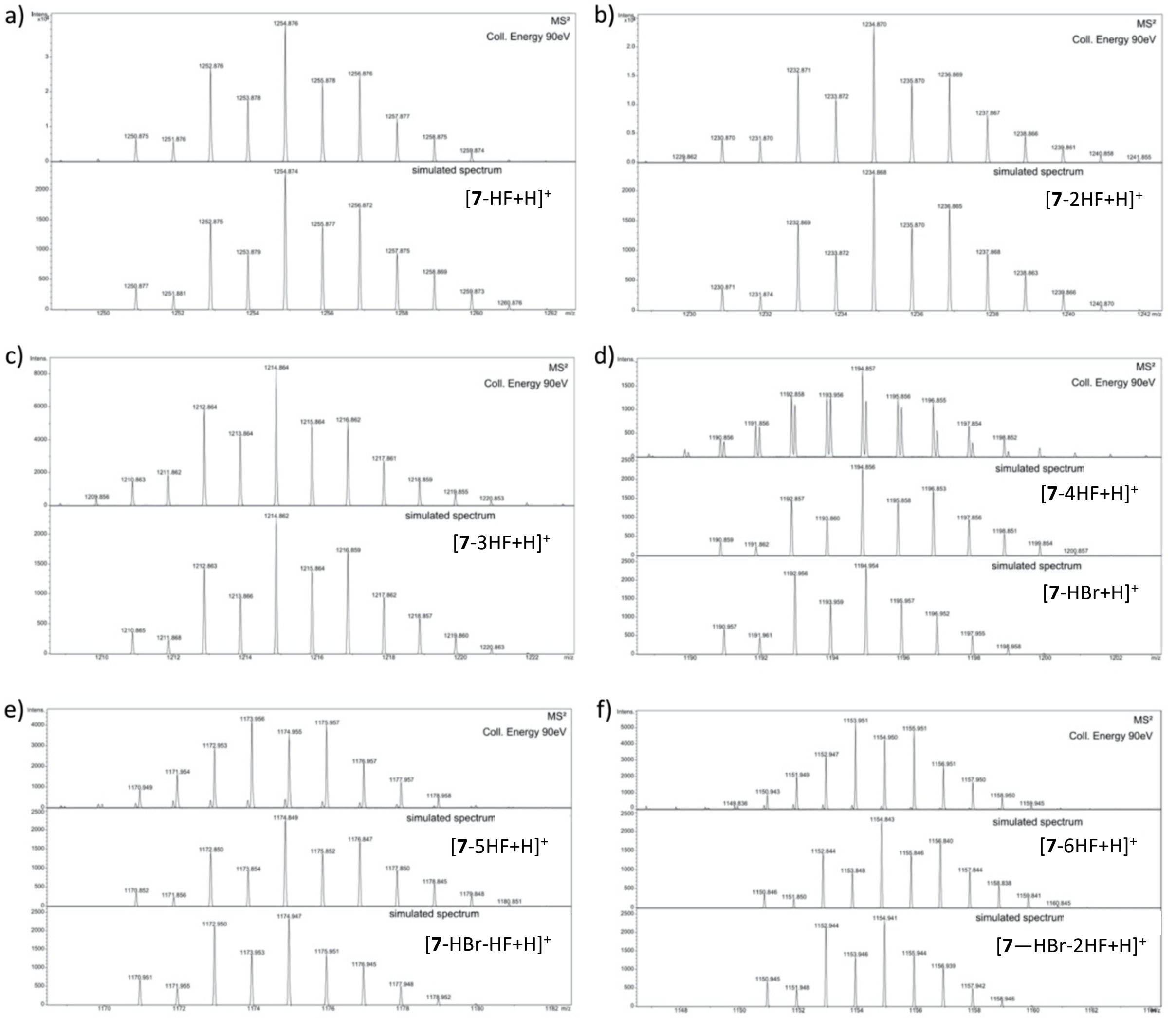

Figure S69. HRMS spectrum (top) and simulated spectrum (bootom of: a) [**7**-HF+H]⁺; b) [**7**-2HF+H]⁺; c) [**7**-3HF+H]⁺; d) [**7**-4HF+H]⁺ (simulation middle) and [**7**-HBr+H]⁺ (simulation bottom); e) [**7**-5HF+H]⁺ (simulation middle) and [**7**-HBr-HF+H]⁺ (simulation bottom); f) [**7**-6HF+H]⁺ (simulation middle) and [**7**-HBr-2HF+H]⁺ (simulation bottom).

FT-ICR experiments with Ref (5,10,15,20-tetrakis(pentafluorophenyl)-porphyrin)

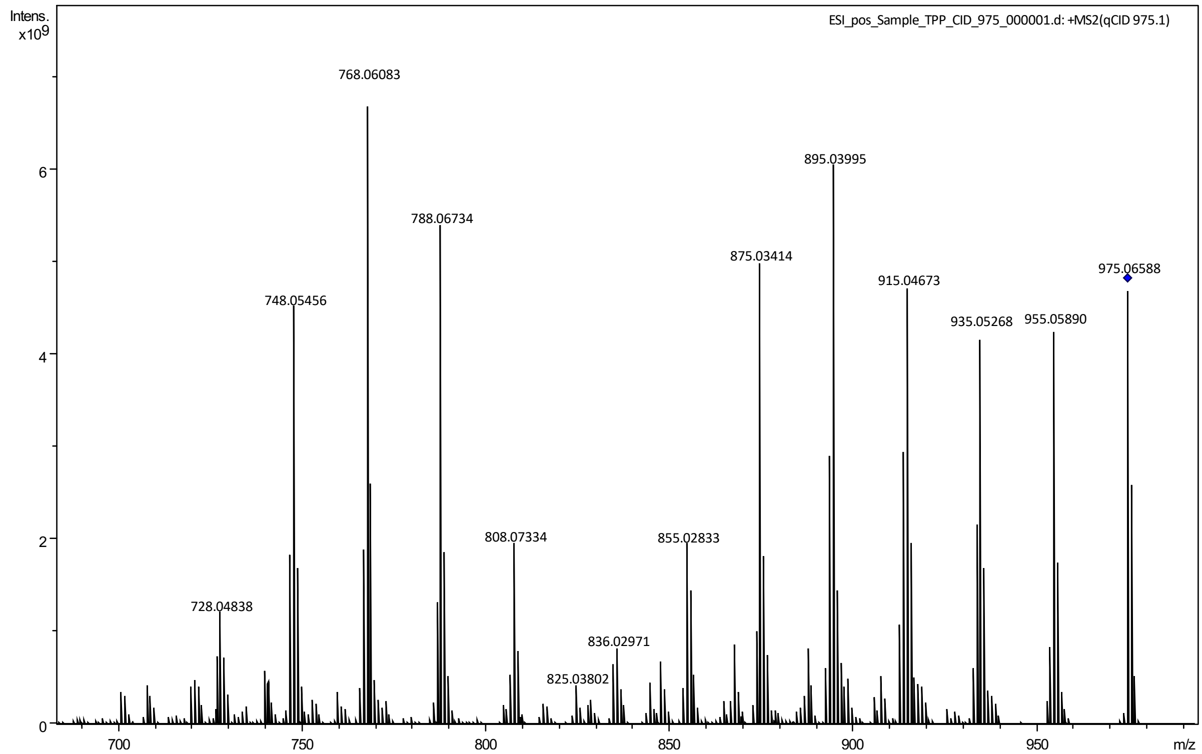

Figure S70. Overview on MS^2 experiments of monomer [**Ref**+H]$^+$ with FT-ICR.

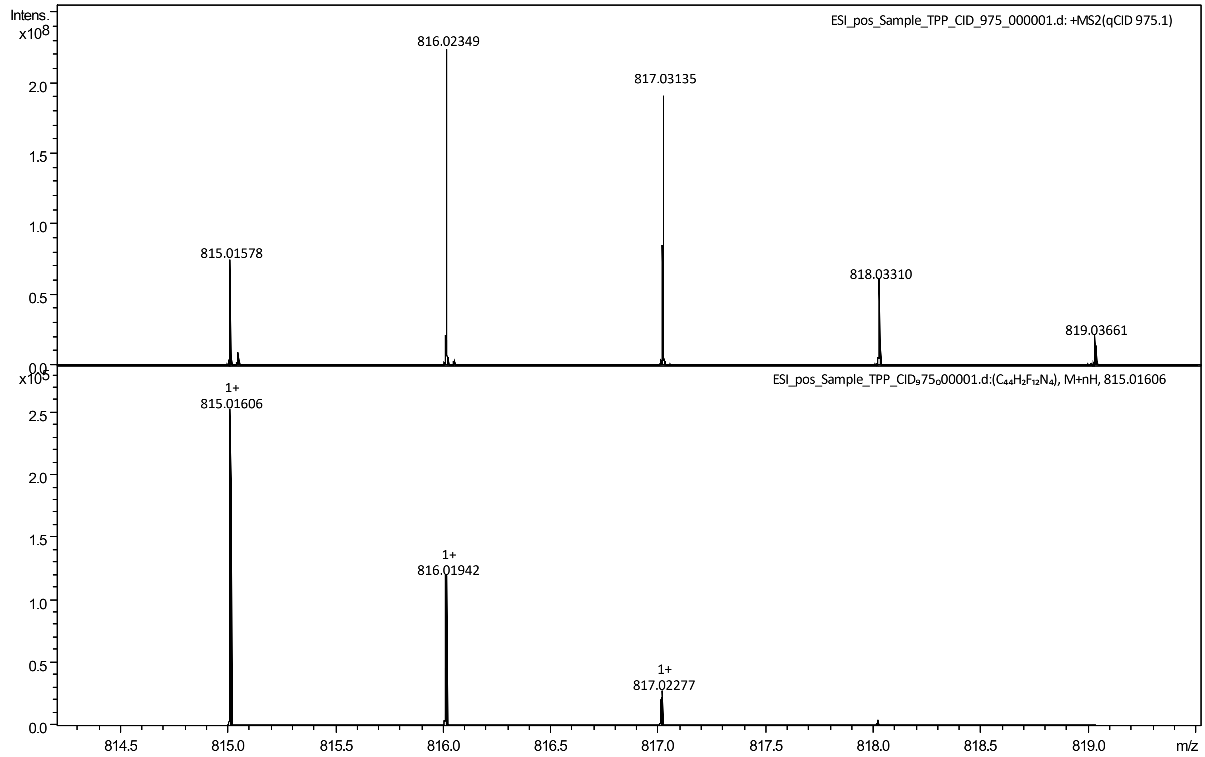

Figure S71. HRMS spectrum (top), and simulated spectrum [**Ref**+H-8HF]$^+$ (bottom).

2.4 Chapter S4 – Dissociation Experiments of 3, 4, 5, 6, and 6Pd

QTOF experiments with 3

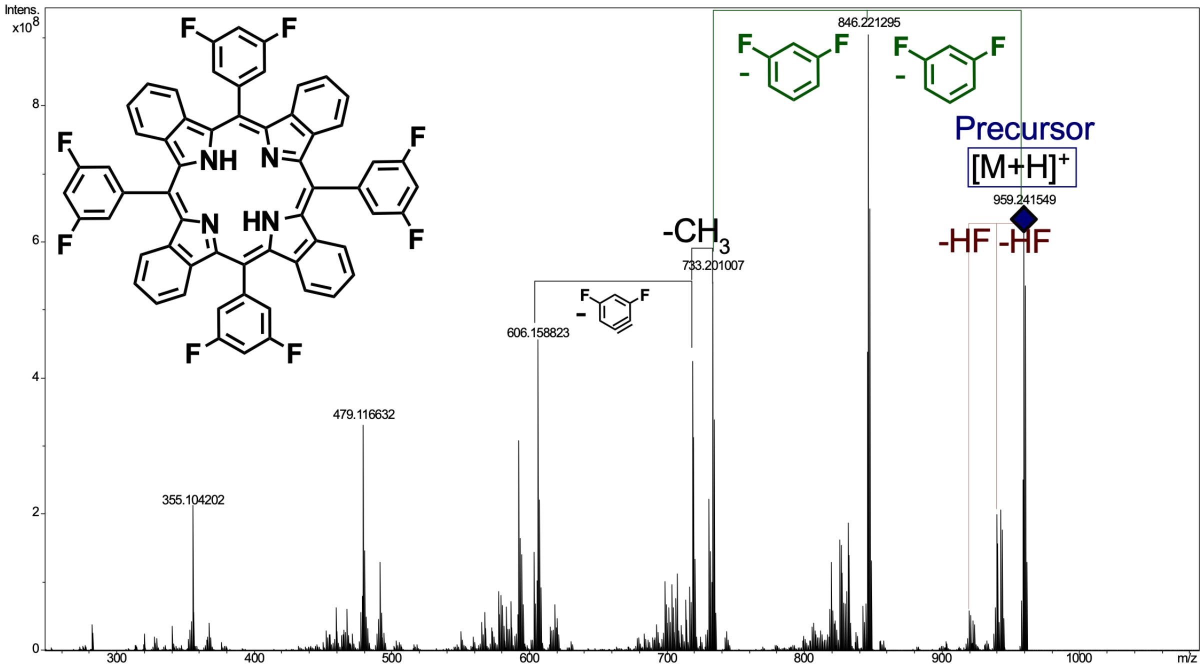

Figure S72. Overview on MS^2 experiments of monomer $[\mathbf{3}+H]^+$ with QTOF.

FT-ICR experiments with 3

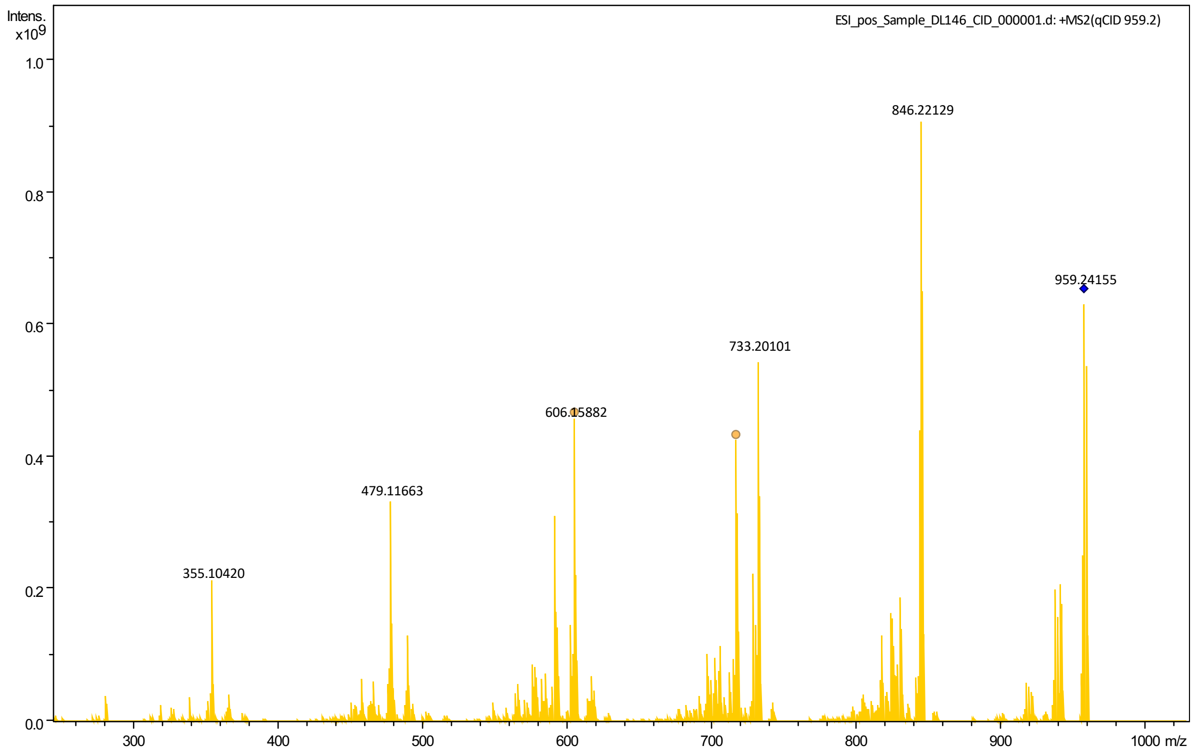

Figure S73. Overview on MS^2 experiments of monomer $[\mathbf{3}+H]^+$ with FT-ICR.

QTOF experiments with 4

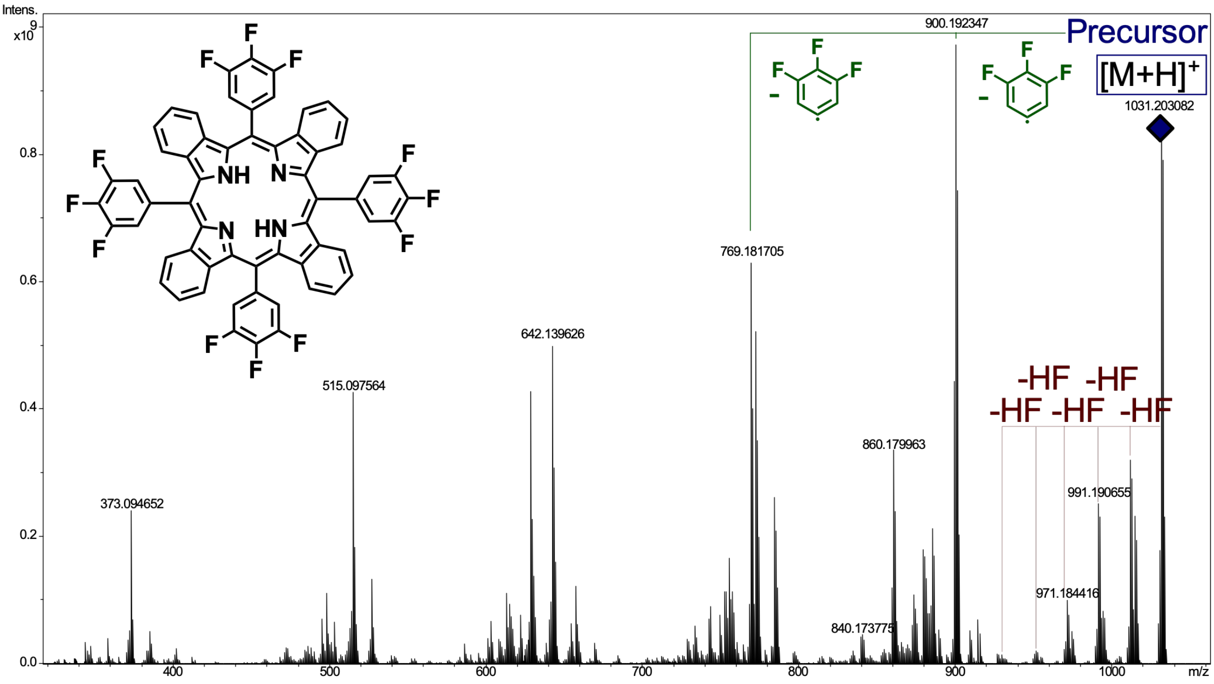

Figure S74. Overview on MS^2 experiments of monomer [**4**+H]$^+$ with QTOF.

FT-ICR experiments with 4

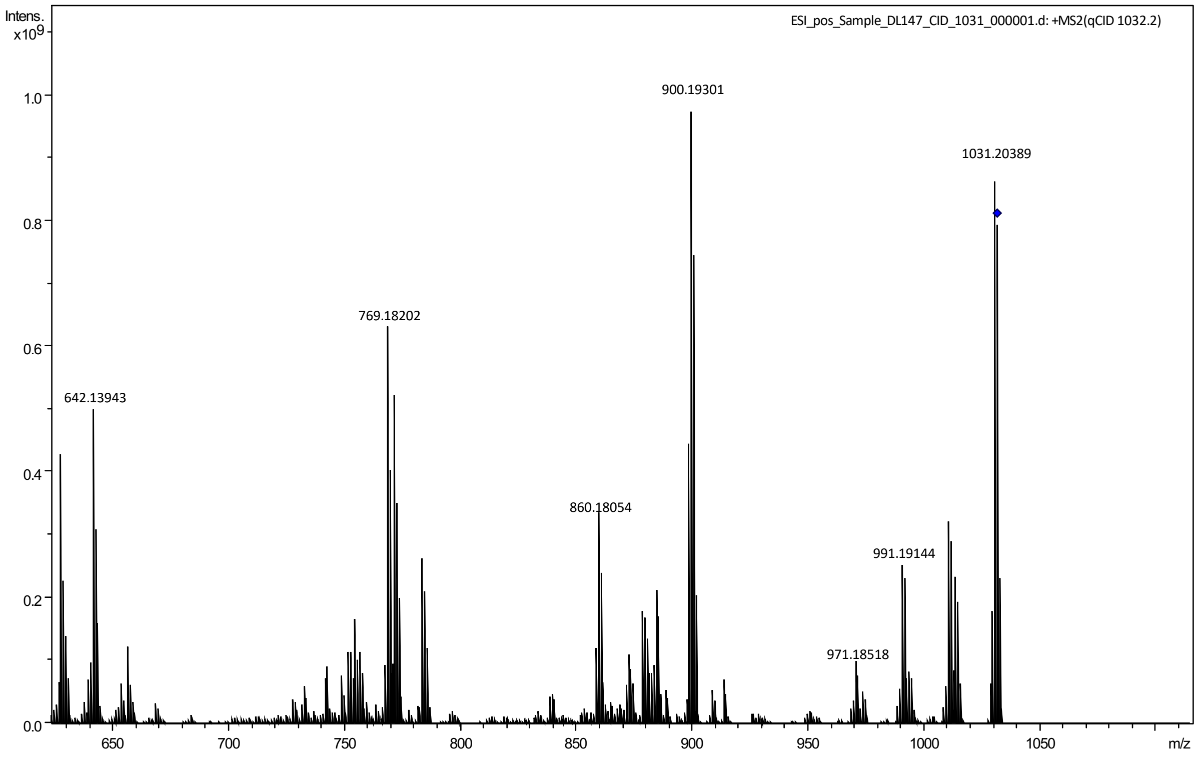

Figure S75. Overview on MS^2 experiments of monomer [**4**+H]$^+$ with FT-ICR.

QTOF experiments with 5

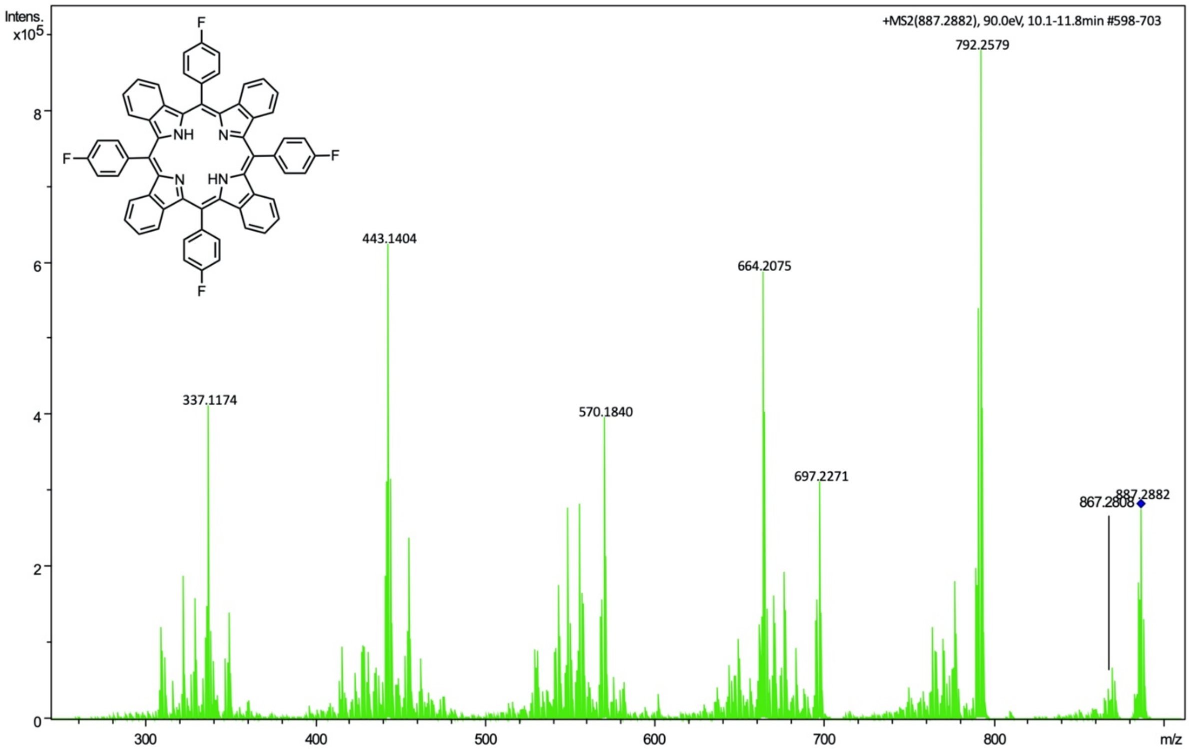

Figure S76. Overview on MS^2 experiments of monomer [**5**+H]$^+$ with QTOF.

QTOF experiments with 6

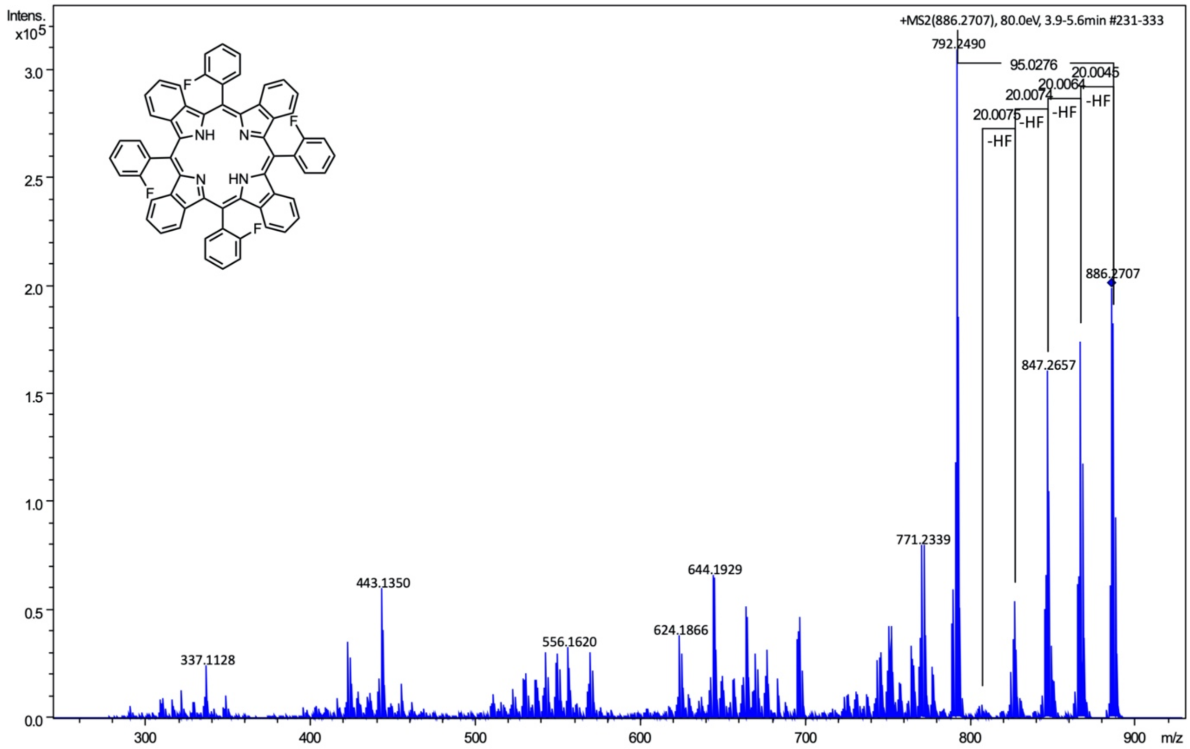

Figure S77. Overview on MS^2 experiments of monomer [**6**+H]$^+$ with QTOF.

QTOF experiments with 6Pd

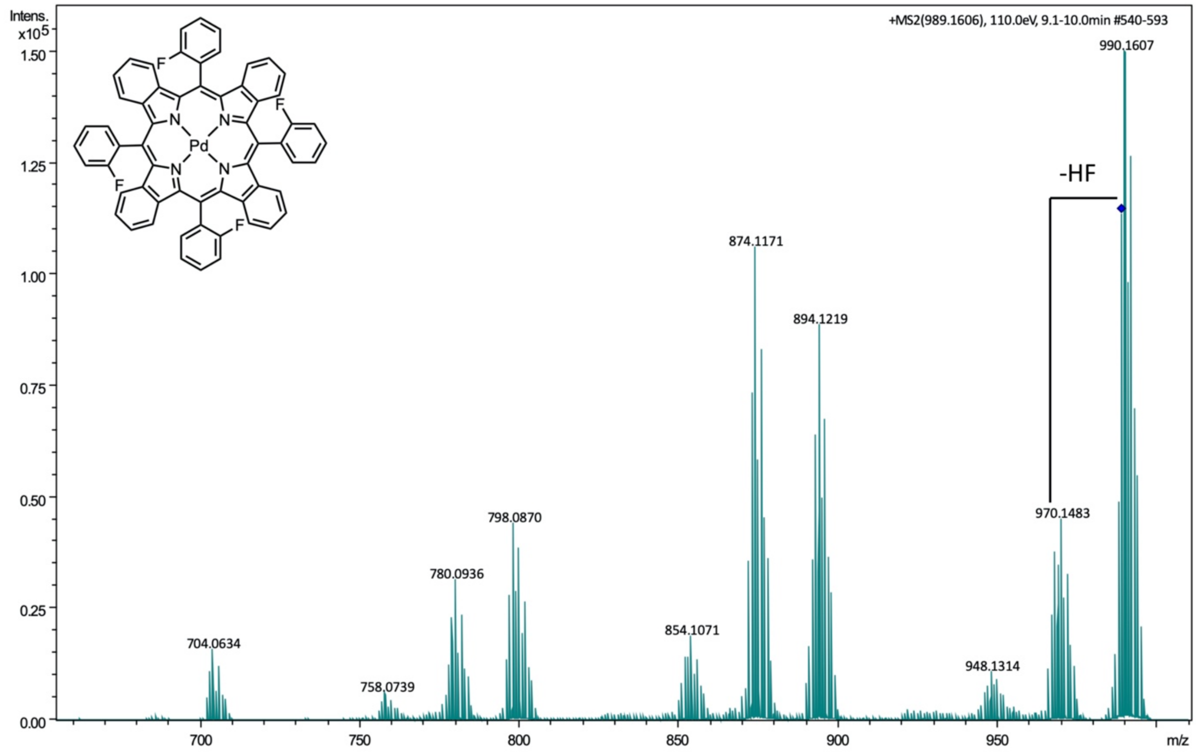

Figure S78. Overview on MS^2 experiments of monomer [**6Pd**]$^{+\bullet}$ with QTOF.

3. COMPUTATIONAL ANALYSIS

3.1 General information

For comparison of our mass spectra with DFT data we performed geometry optimizations for the detected cations. Starting with the intact benzoporphyrin cation we stepwise eliminated HF under consideration of all possible pathways, following the path of the most stable intermediate.

For the quantum chemical calculations in the manuscript, we employed the range-separated hybrid functional ωB97X-D,[5] which employs the Grimme D2 correction, in combination with the 6-311G(d,p) Pople basis set, using the Spartan ’16 work package.[6]

Comparable results were obtained from the exchange-correlation functional of Perdew, Burke, and Ernzerhof (PBE)[7] in combination with the def2-TZVP basis set of Weigend and Ahlrichs[8] using the RI-J[9] and MARI-J[10] approximation. Additionally the Grimme D3 dispersion correction[11] was employed. All quantum chemical calculations were performed using the Turbomole suite.[12]

The following numbering for cyclized bonds is used in the tables below.

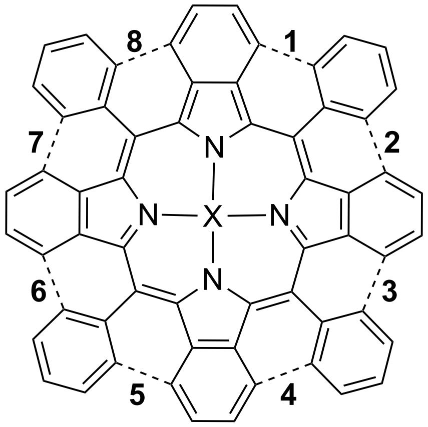

3.2 Energy data on cyclization of [1+H]$^+$ and [1Co]$^{+\bullet}$

Table S4. Energy values of geometry-optimized cyclized intermediates, starting from [**1Co**]$^{+\bullet}$, calculated at the ωB97X-D/6-311G(d,p) level of theory.

-xHF	Closed bonds	E_{total} [eV]	E_{total} + E_{HF} [eV]	E_{rel} to 0 [eV]	ΔE_{step} [eV]
0	0	-160386.1830	-160386.1830	0	0
1	1	-157653.6575	-160386.8789	-0.695877132	-0.695877132
2	12	-154921.2401	-160387.6829	-1.499892368	-0.804015236
3	125	-152188.7548	-160388.4191	-2.236069584	-0.736177216
4	1256	-149456.5757	-160389.4614	-3.278375049	-1.042305466
5	12563	-146723.5143	-160389.6214	-3.43835087	-0.159975821
6	125634	-143990.8248	-160390.1533	-3.970279317	-0.531928447
7	1256347	-141257.3714	-160389.9213	-3.738302132	0.231977185
8	12563478	-138524.8562	-160390.6276	-4.44460123	-0.706299098

Table S5. Energy values of geometry-optimized cyclized intermediates, starting from [**1**+H]$^+$, calculated at the ωB97X-D/6-311G(d,p) level of theory.

-xHF	Closed bonds	E_{total} [eV]	E_{total} + E_{HF} [eV]	E_{rel} to 0 [eV]	ΔE_{step} [eV]
0	0	-122806.0471	-122806.0471	0	0
1	1	-120073.4300	-122806.6514	-0.604256348	-0.604256348
2	12	-117341.1085	-122807.5513	-1.504218981	-0.899962632
3	125	-114608.5916	-122808.2559	-2.208776549	-0.704557569
4	1256	-111876.2704	-122809.1561	-3.109011296	-0.900234746
5	12563	-109143.2965	-122809.4036	-3.356471767	-0.247460472
6	125634	-106410.3367	-122809.6652	-3.618082167	-0.2616104
7	1256347	-103677.3445	-122809.8945	-3.847392635	-0.229310468
8	12563478	-100944.5571	-122810.3285	-4.28133283	-0.433940196

3.3 Energy data on cyclization of [1+H]$^+$ and [1Cu]$^{+\bullet}$

Table S6. Energy values of geometry-optimized cyclized intermediates, starting from [**1Cu**]$^{+\bullet}$, calculated at the PBE/def2-TZVP D3 level of theory.

-xHF	Closed bonds	E_{total} [eV]	E_{total} + E_{HF} [eV]	E_{rel} to 0 [eV]	ΔE_{step} [eV]
0	0	-167319.969	-167319.969	0	0
1	1	-164588.899	-167320.688	-0.7189835	-0.7189835
2	12	-161858.145	-167321.723	-1.7540195	-1.035036
3	125	-159126.992	-167322.359	-2.3902931	-0.6362736
4	1256	-156396.305	-167323.461	-3.4920937	-1.1018006
5	12563	-153664.867	-167323.812	-3.8431134	-0.3510197
6	125634	-150933.584	-167324.317	-4.3483955	-0.5052821
7	1256347	-148202.096	-167324.618	-4.6491816	-0.3007861
8	12563478	-145470.901	-167325.212	-5.2433116	-0.59413

Table S7. Energy values of geometry-optimized cyclized intermediates, starting from [**1**+H]$^+$, calculated at the PBE/def2-TZVP D3 level of theory.

-xHF	Closed bonds	E_{total} [eV]	E_{total} + E_{HF} [eV]	E_{rel} to 0 [eV]	ΔE_{step} [eV]
0	0	-122732.806	-122732.806	0	0
1	1	-120001.708	-122733.497	-0.6903124	-0.6903124
2	12	-117270.864	-122734.441	-1.635041	-0.9447286
3	126	-114539.873	-122735.240	-2.4331287	-0.7980877
4	1265	-111809.051	-122736.207	-3.4003781	-0.9672494
5	12653	-109077.635	-122736.579	-3.7725678	-0.3721897
6	126534	-106346.205	-122736.939	-4.132385	-0.3598172
7	1265347	-103614.804	-122737.326	-4.5200164	-0.3876314
8	12653478	-100883.523	-122737.834	-5.0276159	-0.5075995

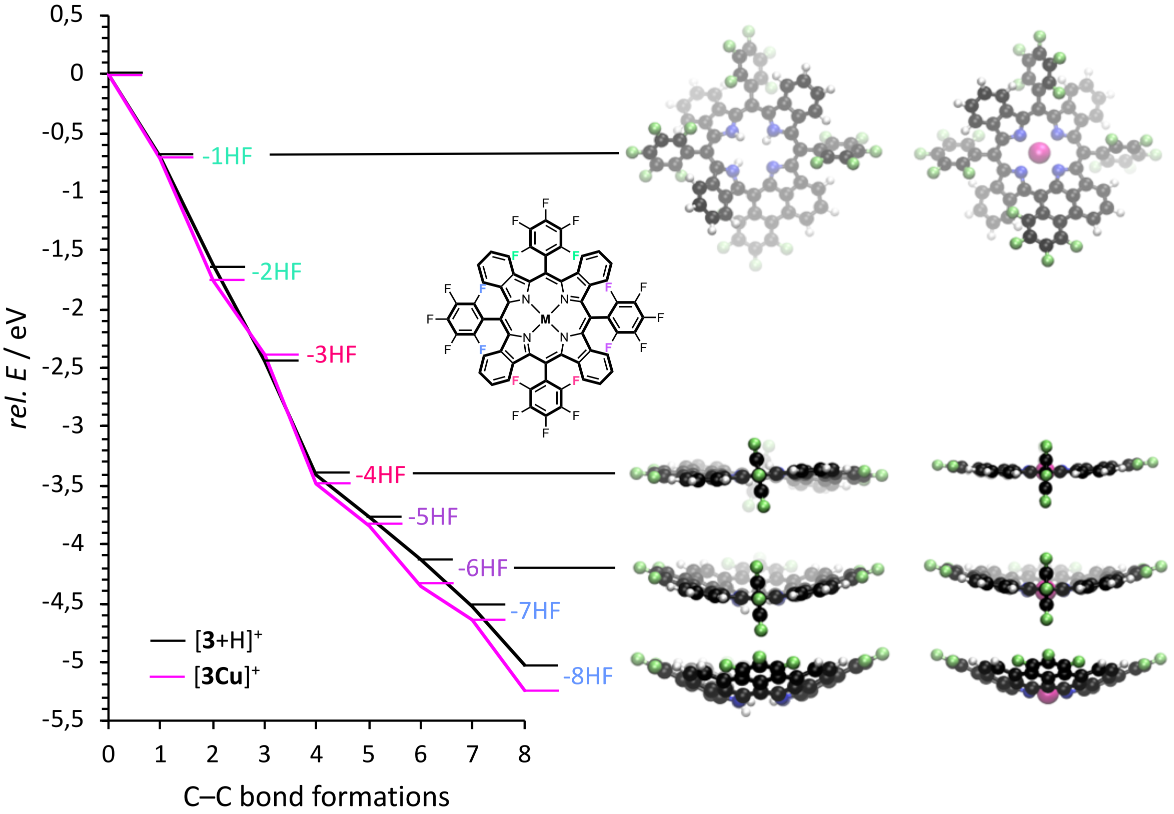

Figure S79. Summary of the computational analysis of the stepwise C–C bond formation through HF elimination of [**1**+H]$^{+}$ and [**1Cu**]$^{+\bullet}$ at the DFT PBE/def2-TZVP D3 level of theory.

3.4 Normal-coordinate structural decomposition analysis

For Normal-coordinate Structural Decomposition (NSD) analyses, we used the NSD engine program as provided by J. A. Shelnutt,[13] which was kindly handed to us by the Senge group from Dublin. The theory and development of this method have been described by Shelnutt and co-workers.[14-16]

NSD employs the decomposition of the conformation of the porphyrin macrocycle by a basis set composed of its various normal modes of vibration. The result affords the quantitative separation of the contributing distortions to the macrocycle conformation. The summaries of the NSD are given in Å:

The normal-modes for the out-of-plane distortions of the minimum basis consist of the lowest energy vibration from each symmetry and comprise the saddle (B_{2u}), ruffle (B_{1u}), domed (A_{2u}), propellered (A_{1u}) and the degenerate wave modes [E_g(x) and E_g(y)]. The in-plane modes that compose the minimum basis are the *meso*-stretching (B_{2g}), N-stretching (B_{1g}), pyrrole-translation [E_u(x) and E_u(y)], breathing (A_{1g}) and pyrrole-rotation (A_{2g}).[17,18]

[**1**+H]$^+$ -0HF:

NSD result generated from file F5P-TBP+H_cation_wB97X-D-6-311Gss.pdb

**basis**	**Dip**	**dip**	**B2g**	**B1g**	**Eu(x)**	**Eu(y)**	**A1g**	**A2g**
min.	0.4015	0.0950	-0.0019	0.0624	-0.0015	0.0347	-0.3951	-0.0005
ext.	0.4836	0.0771	-0.0019	0.0633	-0.0015	0.0341	-0.3959	-0.0004
			0.0004	0.0454	0.0005	-0.0469	-0.2613	0.0104
comp.	0.6584	0.0000	0.0020	0.0789	0.0025	0.1677	0.6317	0.0104

**basis**	**Doop**	**doop**	**B2u**	**B1u**	**A2u**	**Eg(x)**	**Eg(y)**	**A1u**
min.	3.2577	0.0468	3.2542	0.0375	0.0321	0.0013	-0.1427	-0.0003
ext.	3.2712	0.0041	3.2489	0.0375	0.0315	0.0013	-0.1470	-0.0003
			-0.2736	-0.0019	-0.0145	-0.0013	-0.1148	-0.0003
comp.	3.2713	0.0000	3.2657	0.0375	0.0364	0.0020	0.1843	0.0004

[**1**+H]$^+$ -1HF:

NSD result generated from file -1HFd_F5P-TBP+H_cation_wB97X-D-6311Gss.pdb

**basis**	**Dip**	**dip**	**B2g**	**B1g**	**Eu(x)**	**Eu(y)**	**A1g**	**A2g**
min.	0.4477	0.0997	-0.1431	0.0795	-0.2131	0.1208	-0.3370	-0.0091
ext.	0.5503	0.0773	-0.1439	0.0804	-0.2129	0.1202	-0.3375	-0.0070
			-0.1207	0.0471	0.0176	-0.0550	-0.1641	0.2352
comp.	0.7321	0.0001	0.1891	0.1464	0.2811	0.2432	0.5327	0.2382

**basis**	**Doop**	**doop**	**B2u**	**B1u**	**A2u**	**Eg(x)**	**Eg(y)**	**A1u**
min.	2.8923	0.0594	2.6396	1.0090	-0.2088	0.3309	-0.4763	0.0066
ext.	2.9135	0.0183	2.6334	1.0090	-0.2046	0.3341	-0.4776	0.0066
			-0.3220	0.0320	0.0979	0.0860	-0.0353	-0.0127
comp.	2.9158	0.0000	2.6593	1.0097	0.2308	0.3505	0.4839	0.0144

[**1**+H]$^+$ -2HF:

NSD result generated from file -2HFa_F5P-TBP+H_cation_wB97X-D-6311Gss.pdb

**basis**	**Dip**	**dip**	**B2g**	**B1g**	**Eu(x)**	**Eu(y)**	**A1g**	**A2g**
min.	0.2982	0.0633	-0.2478	0.0823	-0.0871	0.1105	-0.0290	0.0104
ext.	0.3568	0.0507	-0.2483	0.0830	-0.0868	0.1097	-0.0295	0.0105
			-0.0859	0.0376	0.0251	-0.0697	-0.1553	0.0040
comp.	0.4775	0.0000	0.2671	0.0928	0.1012	0.2245	0.2953	0.0116

**basis**	**Doop**	**doop**	**B2u**	**B1u**	**A2u**	**Eg(x)**	**Eg(y)**	**A1u**
min.	2.1353	0.0365	2.0661	-0.0034	0.0335	-0.2192	-0.4893	-0.0448
ext.	2.1483	0.0093	2.0622	-0.0034	0.0326	-0.2167	-0.4929	-0.0448
			-0.2044	0.0081	-0.0203	0.0686	-0.0943	0.0134
comp.	2.1491	0.0000	2.0763	0.0104	0.0407	0.2314	0.5001	0.0468

[**1**+H]$^+$ -3HF:

NSD result generated from file -3HFd_F5P-TBP+H_cation_wB97X-D-6311Gss.pdb

**basis**	**Dip**	**dip**	**B2g**	**B1g**	**Eu(x)**	**Eu(y)**	**A1g**	**A2g**
min.	0.3347	0.0742	-0.3188	0.0594	0.0659	-0.0184	-0.0362	0.0288
ext.	0.4284	0.0539	-0.3203	0.0596	0.0658	-0.0186	-0.0365	0.0278
			-0.2191	0.0099	-0.0113	-0.0231	-0.1048	-0.1084
comp.	0.5693	0.0000	0.3904	0.1164	0.1095	0.2563	0.2586	0.1164

**basis**	**Doop**	**doop**	**B2u**	**B1u**	**A2u**	**Eg(x)**	**Eg(y)**	**A1u**
min.	1.7839	0.0507	1.3513	-0.8205	0.2666	0.2183	-0.7510	-0.0146
ext.	1.8092	0.0164	1.3466	-0.8205	0.2608	0.2185	-0.7549	-0.0146
			-0.2471	-0.0316	-0.1340	0.0045	-0.1045	0.0026
comp.	1.8123	0.0000	1.3740	0.8214	0.2997	0.2298	0.7608	0.0149

[**1**+H]$^+$ -4HF:

NSD result generated from file -4HFa_F5P-TBP+H_cation_wB97X-D-6311Gss.pdb

**basis**	**Dip**	**dip**	**B2g**	**B1g**	**Eu(x)**	**Eu(y)**	**A1g**	**A2g**
min.	0.4374	0.0493	-0.3868	0.0397	-0.0003	0.0448	0.1949	0.0093
ext.	0.4855	0.0316	-0.3880	0.0404	-0.0004	0.0450	0.1946	0.0092
			-0.1817	0.0363	-0.0150	0.0123	-0.0982	-0.0092
comp.	0.5180	0.0000	0.4365	0.0590	0.0238	0.0993	0.2525	0.0136

**basis**	**Doop**	**doop**	**B2u**	**B1u**	**A2u**	**Eg(x)**	**Eg(y)**	**A1u**
min.	0.6834	0.0299	0.6337	-0.0687	0.0300	0.1374	-0.1868	-0.0776
ext.	0.7143	0.0031	0.6310	-0.0687	0.0300	0.1404	-0.1915	-0.0776
			-0.1439	0.0058	0.0005	0.0793	-0.1265	0.0124
comp.	0.7145	0.0000	0.6499	0.0689	0.0300	0.1592	0.2258	0.0786

[**1**+H]$^+$ -5HF:

NSD result generated from file -5HFb_F5P-TBP+H_cation_wB97X-D-6311Gss.pdb

**basis**	**Dip**	**dip**	**B2g**	**B1g**	**Eu(x)**	**Eu(y)**	**A1g**	**A2g**
min.	0.5879	0.0570	-0.5134	-0.2203	-0.1667	0.0189	0.0611	-0.0414
ext.	0.6234	0.0414	-0.5142	-0.2206	-0.1676	0.0194	0.0608	-0.0410
			-0.1294	-0.0162	-0.0849	0.0435	-0.1243	0.0387
comp.	0.6792	0.0000	0.5339	0.2295	0.1908	0.1926	0.2154	0.0604

**basis**	**Doop**	**doop**	**B2u**	**B1u**	**A2u**	**Eg(x)**	**Eg(y)**	**A1u**
min.	1.0106	0.0555	0.5945	0.5666	0.3760	0.0205	-0.4460	-0.0777
ext.	1.0636	0.0160	0.5906	0.5666	0.3724	0.0247	-0.4540	-0.0777
			-0.2037	0.0495	-0.0841	0.1126	-0.2146	0.0144
comp.	1.0681	0.0000	0.6285	0.5692	0.3857	0.1384	0.4978	0.0790

[**1**+H]+ -6HF:

NSD result generated from file -6HFa_F5P-TBP+H_cation_wB97X-D-6311Gss.pdb

**basis**	**Dip**	**dip**	**B2g**	**B1g**	**Eu(x)**	**Eu(y)**	**A1g**	**A2g**
min.	0.2907	0.0568	-0.2314	0.0240	-0.1502	-0.0873	0.0129	0.0048
ext.	0.3366	0.0465	-0.2320	0.0245	-0.1497	-0.0865	0.0125	0.0048
			-0.0856	0.0304	0.0441	0.0728	-0.1153	0.0030
comp.	0.4374	0.0000	0.2532	0.0412	0.1715	0.1987	0.2379	0.0080

**basis**	**Doop**	**doop**	**B2u**	**B1u**	**A2u**	**Eg(x)**	**Eg(y)**	**A1u**
min.	0.9459	0.0671	0.1205	0.2806	0.8769	-0.0781	-0.1624	0.0032
ext.	1.0260	0.0050	0.1191	0.2806	0.8811	-0.0866	-0.1737	0.0032
			-0.0723	-0.0051	0.0984	-0.2280	-0.3022	-0.0007
comp.	1.0264	0.0000	0.1405	0.2807	0.8826	0.2414	0.3432	0.0032

[**1**+H]+ -7HF:

NSD result generated from file -7HFa_F5P-TBP+H_cation_wB97X-D-6311Gss.pdb

**basis**	**Dip**	**dip**	**B2g**	**B1g**	**Eu(x)**	**Eu(y)**	**A1g**	**A2g**
min.	0.4070	0.0715	-0.3160	-0.1567	-0.0293	0.1653	-0.1044	-0.0461
ext.	0.4606	0.0587	-0.3160	-0.1570	-0.0307	0.1653	-0.1049	-0.0459
			0.0034	-0.0201	-0.1204	0.0025	-0.1769	0.0181
comp.	0.5831	0.0000	0.3201	0.1807	0.1346	0.2296	0.3626	0.0512

**basis**	**Doop**	**doop**	**B2u**	**B1u**	**A2u**	**Eg(x)**	**Eg(y)**	**A1u**
min.	1.3964	0.0362	0.4165	0.3917	1.2333	0.0295	-0.3132	-0.0557
ext.	1.4131	0.0158	0.4144	0.3917	1.2351	0.0354	-0.3104	-0.0557
			-0.1097	0.0505	0.0430	0.1585	0.0732	0.0081
comp.	1.4164	0.0000	0.4308	0.3954	1.2341	0.1793	0.3256	0.0563

[**1**+H]+ -8HF:

NSD result generated from file -8HF_F5P-TBP+H_cation_wB97X-D-6311Gss.pdb

**basis**	**Dip**	**dip**	**B2g**	**B1g**	**Eu(x)**	**Eu(y)**	**A1g**	**A2g**
min.	0.1854	0.0733	0.0200	0.0969	0.0024	0.0913	-0.1273	-0.0051
ext.	0.2582	0.0647	0.0200	0.0975	0.0024	0.0916	-0.1278	-0.0051
			0.0013	0.0324	0.0049	0.0291	-0.1743	0.0007
comp.	0.4559	0.0000	0.0205	0.1057	0.0090	0.1603	0.4128	0.0057

**basis**	**Doop**	**doop**	**B2u**	**B1u**	**A2u**	**Eg(x)**	**Eg(y)**	**A1u**
min.	1.4244	0.0313	0.1839	-0.0041	1.4081	0.0033	-0.1101	-0.0040
ext.	1.4392	0.0079	0.1833	-0.0041	1.4159	0.0033	-0.1137	-0.0040
			-0.0321	0.0024	0.1794	0.0013	-0.0970	-0.0001
comp.	1.4399	0.0000	0.1868	0.0047	1.4201	0.0065	0.1468	0.0040

[**1Co**]+• -0HF:

NSD result generated from file Co-F5P-TBP-cation_wB97X-D-6311Gss.pdb

**basis**	**Dip**	**dip**	**B2g**	**B1g**	**Eu(x)**	**Eu(y)**	**A1g**	**A2g**
min.	0.7929	0.0704	-0.0510	0.0648	0.0003	-0.0009	-0.7884	0.0171
ext.	0.8314	0.0479	-0.0512	0.0652	0.0003	-0.0009	-0.7889	0.0189
			-0.0295	0.0238	0.0005	0.0001	-0.1558	0.1917
comp.	0.8697	0.0001	0.0697	0.0700	0.0013	0.0014	0.8416	0.1958

**basis**	**Doop**	**doop**	**B2u**	**B1u**	**A2u**	**Eg(x)**	**Eg(y)**	**A1u**
min.	3.4485	0.0177	3.3426	0.8432	0.0216	-0.0007	-0.0005	0.0872
ext.	3.4499	0.0055	3.3408	0.8432	0.0204	-0.0007	-0.0005	0.0872
			-0.0935	-0.0144	-0.0287	0.0011	-0.0001	0.0036
comp.	3.4500	0.0000	3.3439	0.8438	0.0359	0.0014	0.0011	0.0873

[**1Co**]$^{+\bullet}$ -1HF:

NSD result generated from file -1HF_Co-F5P-TBP-cation_wB97X-D-6311Gss.pdb

**basis**	**Dip**	**dip**	**B2g**	**B1g**	**Eu(x)**	**Eu(y)**	**A1g**	**A2g**
min.	0.7272	0.0753	-0.0368	0.0605	-0.0984	-0.1983	-0.6890	-0.0113
ext.	0.8064	0.0370	-0.0361	0.0611	-0.0984	-0.1974	-0.6888	-0.0084
			0.1109	0.0336	0.0005	0.0837	0.0554	0.3130
comp.	0.8380	0.0001	0.1256	0.0933	0.1275	0.2294	0.7144	0.3141

**basis**	**Doop**	**doop**	**B2u**	**B1u**	**A2u**	**Eg(x)**	**Eg(y)**	**A1u**
min.	2.9760	0.0395	2.4336	1.6733	0.1861	-0.2490	-0.1713	0.0923
ext.	2.9861	0.0164	2.4304	1.6732	0.1801	-0.2448	-0.1727	0.0923
			-0.1632	0.0187	-0.1387	0.1104	-0.0391	0.0137
comp.	2.9878	0.0000	2.4391	1.6743	0.2325	0.2796	0.1830	0.0933

[**1Co**]$^{+\bullet}$ -2HF:

NSD result generated from file -2HFa_Co-F5P-TBP-cation_wB97X-D-6311Gss.pdb

**basis**	**Dip**	**dip**	**B2g**	**B1g**	**Eu(x)**	**Eu(y)**	**A1g**	**A2g**
min.	0.4123	0.0383	0.1184	-0.0511	-0.0278	-0.0727	-0.3836	0.0095
ext.	0.4389	0.0279	0.1191	-0.0513	-0.0276	-0.0723	-0.3839	0.0099
			0.1106	-0.0075	0.0170	0.0403	-0.0833	0.0396
comp.	0.4629	0.0000	0.1738	0.0524	0.0675	0.1068	0.4028	0.0558

**basis**	**Doop**	**doop**	**B2u**	**B1u**	**A2u**	**Eg(x)**	**Eg(y)**	**A1u**
min.	2.2666	0.0280	2.1886	0.4010	-0.0350	-0.2866	0.3204	-0.0300
ext.	2.2712	0.0109	2.1884	0.4010	-0.0340	-0.2833	0.3162	-0.0300
			-0.0111	-0.0076	0.0234	0.0871	-0.1103	-0.0167
comp.	2.2721	0.0000	2.1886	0.4013	0.0421	0.3026	0.3421	0.0343

[**1Co**]$^{+\bullet}$ -3HF:

NSD result generated from file -3HFd_Co-F5P-TBP-cation_wB97X-D-6311Gss.pdb

**basis**	**Dip**	**dip**	**B2g**	**B1g**	**Eu(x)**	**Eu(y)**	**A1g**	**A2g**
min.	0.3728	0.0483	0.0763	-0.0036	0.0679	0.0635	-0.3520	-0.0238
ext.	0.4423	0.0278	0.0777	-0.0034	0.0681	0.0633	-0.3518	-0.0229
			0.2099	0.0118	0.0181	-0.0173	0.0459	0.0988
comp.	0.4685	0.0000	0.2275	0.0350	0.1255	0.1036	0.3584	0.1076

**basis**	**Doop**	**doop**	**B2u**	**B1u**	**A2u**	**Eg(x)**	**Eg(y)**	**A1u**
min.	1.8199	0.0357	1.1878	1.2559	0.1528	0.1591	0.5134	0.1081
ext.	1.8355	0.0134	1.1858	1.2559	0.1468	0.1542	0.5099	0.1081
			-0.1069	0.0246	-0.1375	-0.1323	-0.0932	0.0108
comp.	1.8375	0.0000	1.1926	1.2567	0.2057	0.2159	0.5235	0.1086

[**1Co**]$^{+\bullet}$ -4HF:

NSD result generated from file -4HFa_Co-F5P-TBP-cation_wB97X-D-6311Gss.pdb

**basis**	**Dip**	**dip**	**B2g**	**B1g**	**Eu(x)**	**Eu(y)**	**A1g**	**A2g**
min.	0.1345	0.0370	0.1142	-0.0137	-0.0002	0.0001	-0.0696	0.0055
ext.	0.2319	0.0205	0.1154	-0.0136	-0.0002	0.0001	-0.0697	0.0053
			0.1833	0.0041	-0.0000	0.0002	-0.0393	-0.0235
comp.	0.2577	0.0000	0.2323	0.0154	0.0005	0.0008	0.1013	0.0442

**basis**	**Doop**	**doop**	**B2u**	**B1u**	**A2u**	**Eg(x)**	**Eg(y)**	**A1u**
min.	0.2794	0.0050	-0.2573	0.0169	-0.0034	0.0015	0.0019	-0.1076
ext.	0.2809	0.0005	-0.2568	0.0169	-0.0027	0.0015	0.0019	-0.1076
			0.0244	0.0007	0.0140	-0.0006	-0.0009	0.0022
comp.	0.2809	0.0000	0.2585	0.0170	0.0146	0.0016	0.0024	0.1076

[**1Co**]$^{+•}$ -5HF:

NSD result generated from file -5HFa_Co-F5P-TBP-cation_wB97X-D-6311Gss.pdb

**basis**	**Dip**	**dip**	**B2g**	**B1g**	**Eu(x)**	**Eu(y)**	**A1g**	**A2g**
min.	0.3644	0.0352	0.1978	0.1328	-0.0437	0.1534	-0.2209	-0.0426
ext.	0.4018	0.0183	0.1987	0.1337	-0.0432	0.1540	-0.2211	-0.0427
			0.1408	0.0452	0.0441	0.0573	-0.0391	-0.0037
comp.	0.4149	0.0000	0.2516	0.1418	0.0790	0.1657	0.2273	0.0583

**basis**	**Doop**	**doop**	**B2u**	**B1u**	**A2u**	**Eg(x)**	**Eg(y)**	**A1u**
min.	0.9463	0.0247	0.3994	0.7643	-0.2827	0.2236	-0.0735	0.1289
ext.	0.9583	0.0172	0.3980	0.7643	-0.2789	0.2248	-0.0707	0.1289
			-0.0761	0.0477	0.0884	0.0333	0.0747	-0.0153
comp.	0.9640	0.0000	0.4066	0.7661	0.2963	0.2352	0.1306	0.1298

[**1Co**]$^{+•}$ -6HF:

NSD result generated from file -6HFa_Co-F5P-TBP-cation_wB97X-D-6311Gss.pdb

**basis**	**Dip**	**dip**	**B2g**	**B1g**	**Eu(x)**	**Eu(y)**	**A1g**	**A2g**
min.	0.3728	0.0295	0.0846	-0.0072	-0.1721	0.1782	-0.2653	-0.0068
ext.	0.3966	0.0162	0.0853	-0.0073	-0.1716	0.1777	-0.2655	-0.0067
			0.1024	-0.0025	0.0448	-0.0417	-0.0636	0.0056
comp.	0.4089	0.0000	0.1415	0.0081	0.1850	0.1893	0.2775	0.0089

**basis**	**Doop**	**doop**	**B2u**	**B1u**	**A2u**	**Eg(x)**	**Eg(y)**	**A1u**
min.	0.5511	0.0378	0.0012	0.1889	-0.5127	-0.0119	0.0340	-0.0622
ext.	0.5927	0.0065	0.0014	0.1889	-0.5161	-0.0066	0.0285	-0.0622
			0.0114	-0.0060	-0.0776	0.1398	-0.1478	-0.0076
comp.	0.5940	0.0000	0.0115	0.1892	0.5193	0.1412	0.1527	0.0626

[**1Co**]$^{+•}$ -7HF:

NSD result generated from file -7HF_Co-F5P-TBP-cation_wB97X-D-6311Gss.pdb

**basis**	**Dip**	**dip**	**B2g**	**B1g**	**Eu(x)**	**Eu(y)**	**A1g**	**A2g**
min.	0.4307	0.0345	0.0726	0.1669	0.1611	-0.1052	-0.3334	-0.0645
ext.	0.4626	0.0157	0.0733	0.1675	0.1611	-0.1041	-0.3337	-0.0645
			0.0976	0.0327	-0.0002	0.1039	-0.0841	-0.0011
comp.	0.4705	0.0000	0.1248	0.1707	0.1655	0.1524	0.3480	0.0700

**basis**	**Doop**	**doop**	**B2u**	**B1u**	**A2u**	**Eg(x)**	**Eg(y)**	**A1u**
min.	0.6835	0.0491	0.2430	0.3548	0.4346	0.2812	-0.0503	0.1085
ext.	0.7323	0.0195	0.2417	0.3548	0.4430	0.2869	-0.0509	0.1085
			-0.0658	0.0627	0.1944	0.1505	-0.0162	-0.0122
comp.	0.7411	0.0000	0.2518	0.3603	0.4781	0.3225	0.1070	0.1092

[**1Co**]$^{+•}$ -8HF:

NSD result generated from file -8HF_Co_F5P-TBP+H_cation_wB97X-D-6311Gss.pdb

**basis**	**Dip**	**dip**	**B2g**	**B1g**	**Eu(x)**	**Eu(y)**	**A1g**	**A2g**
min.	0.3305	0.0340	0.0016	0.0133	-0.0000	-0.0000	-0.3301	0.0068
ext.	0.3386	0.0308	0.0016	0.0135	-0.0000	-0.0000	-0.3303	0.0065
			0.0003	0.0104	0.0000	-0.0000	-0.0667	-0.0297
comp.	0.3814	0.0000	0.0033	0.0344	0.0000	0.0000	0.3763	0.0514

**basis**	**Doop**	**doop**	**B2u**	**B1u**	**A2u**	**Eg(x)**	**Eg(y)**	**A1u**
min.	1.1698	0.0265	0.0032	0.0000	1.1698	0.0000	0.0000	-0.0060
ext.	1.1823	0.0083	0.0036	0.0000	1.1771	0.0000	0.0000	-0.0060
			0.0174	-0.0000	0.1709	-0.0000	0.0000	-0.0001
comp.	1.1833	0.0000	0.0220	0.0000	1.1830	0.0000	0.0000	0.0060

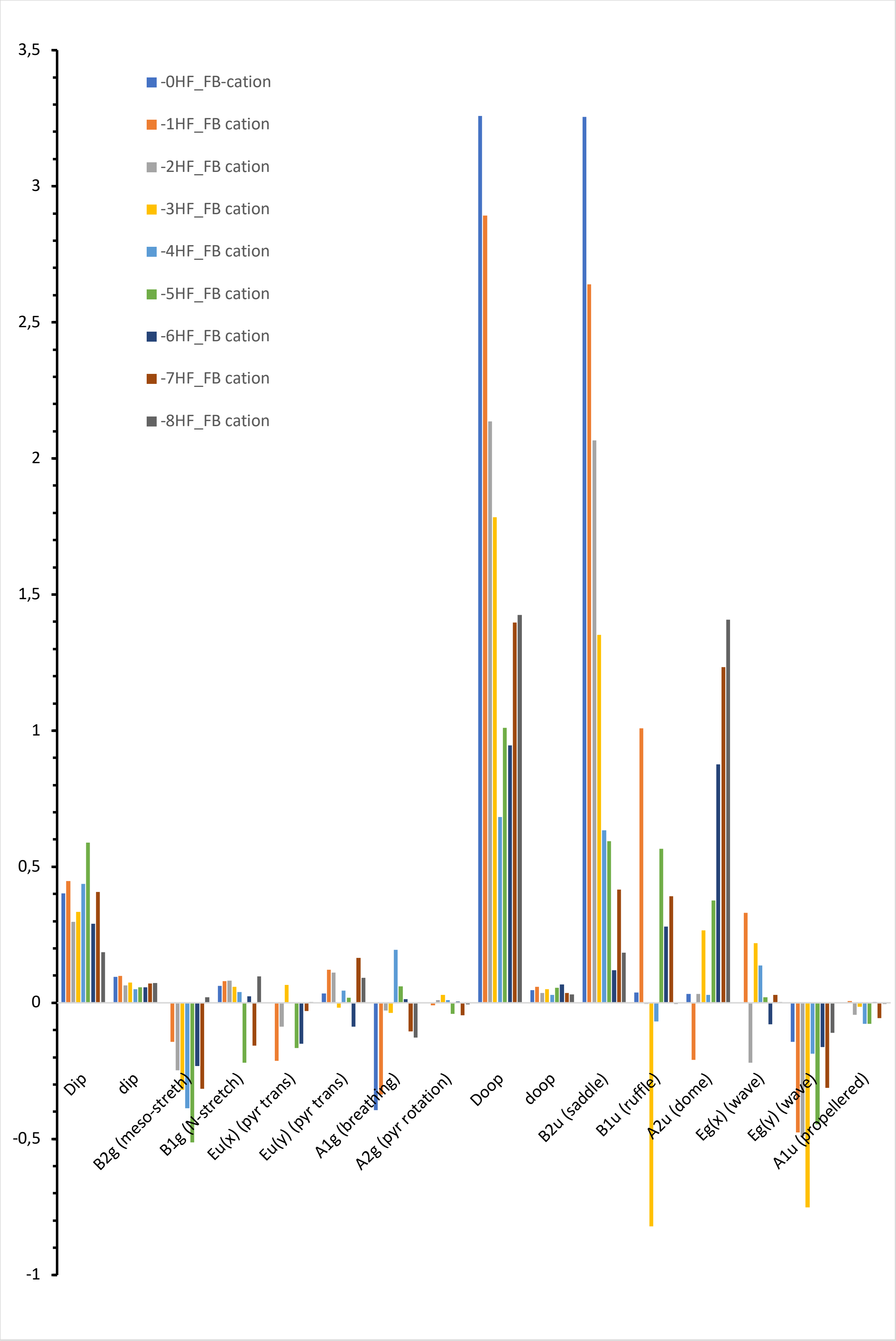

Figure S80. NSD of [**1**+H]$^{+}$ and its HF-elimination intermediates, calculated at the ωB97X-D/6-311G(d,p) level of theory.

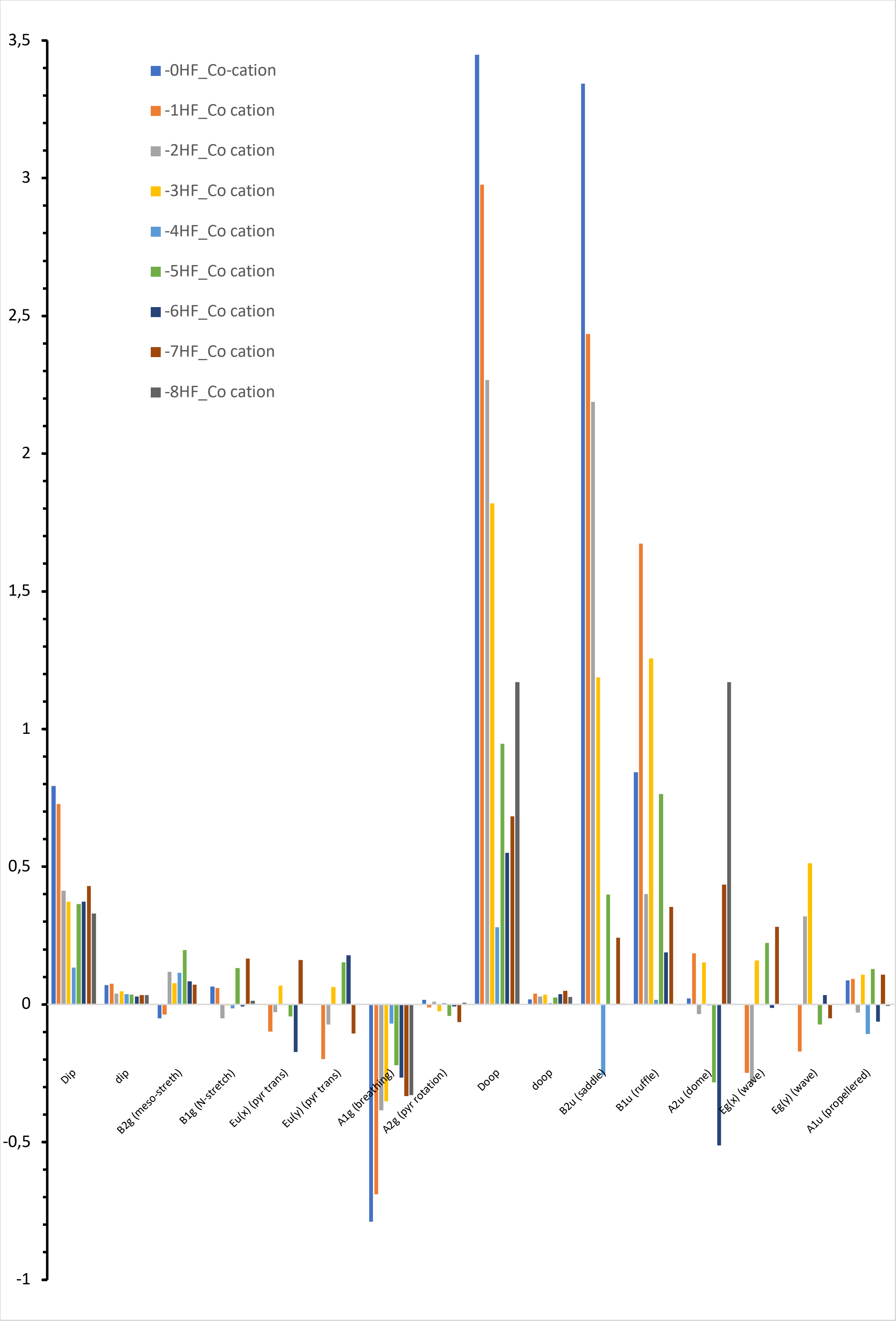

Figure S81. NSD of [**1Co**]$^{+\bullet}$ and its HF-elimination intermediates, calculated at the ωB97X-D/6-311G(d,p) level of theory.

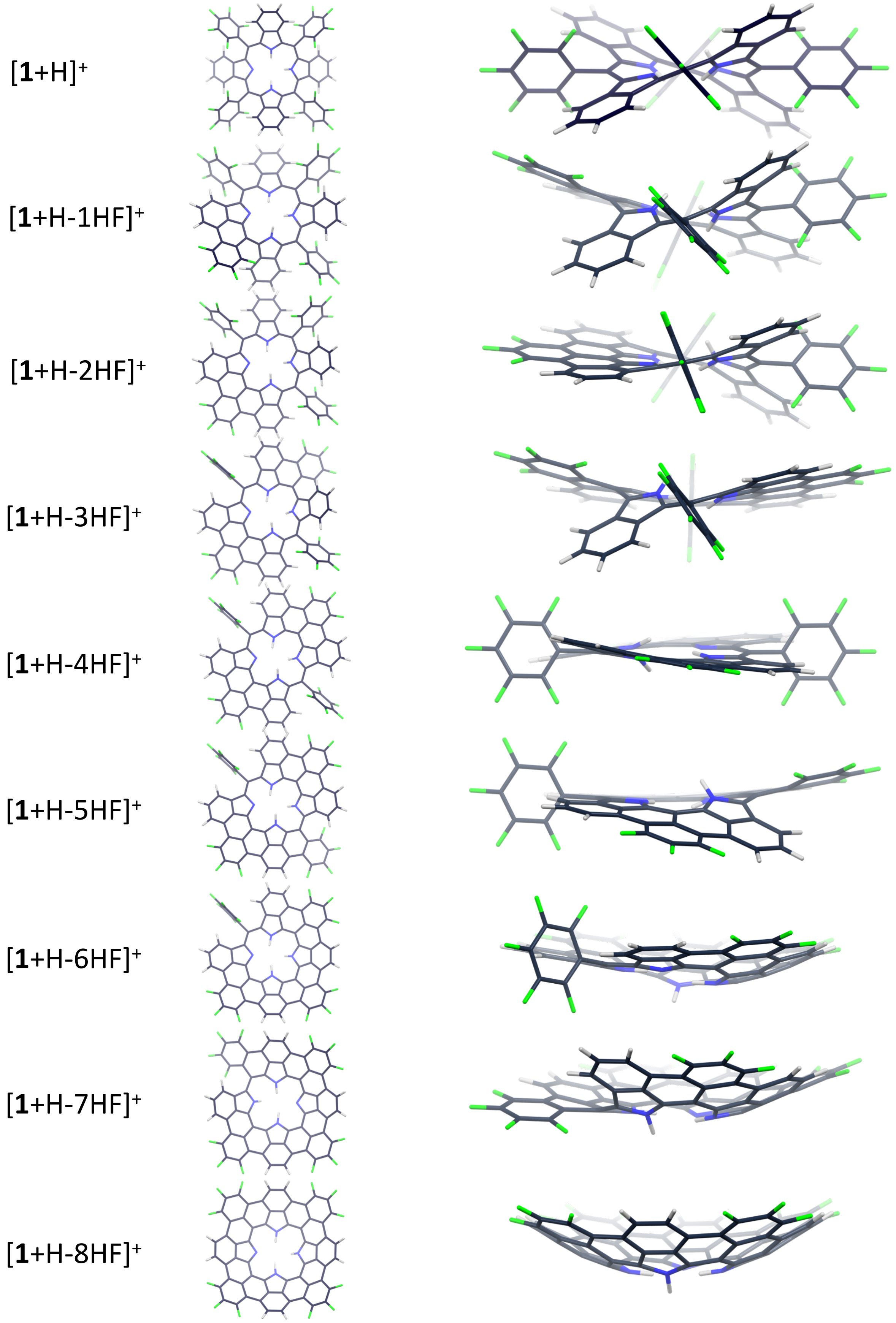

Figure S82. Enlarged geometry optimized structures from Figure 4 of [**1**+H-nHF]$^+$, in top and sideview.

[**1Co**]$^{+\cdot}$

[**1Co**-1HF]$^{+\cdot}$

[**1Co**-2HF]$^{+\cdot}$

[**1Co**-3HF]$^{+\cdot}$

[**1Co**-4HF]$^{+\cdot}$

[**1Co**-5HF]$^{+\cdot}$

[**1Co**-6HF]$^{+\cdot}$

[**1Co**-7HF]$^{+\cdot}$

[**1Co**-8HF]$^{+\cdot}$

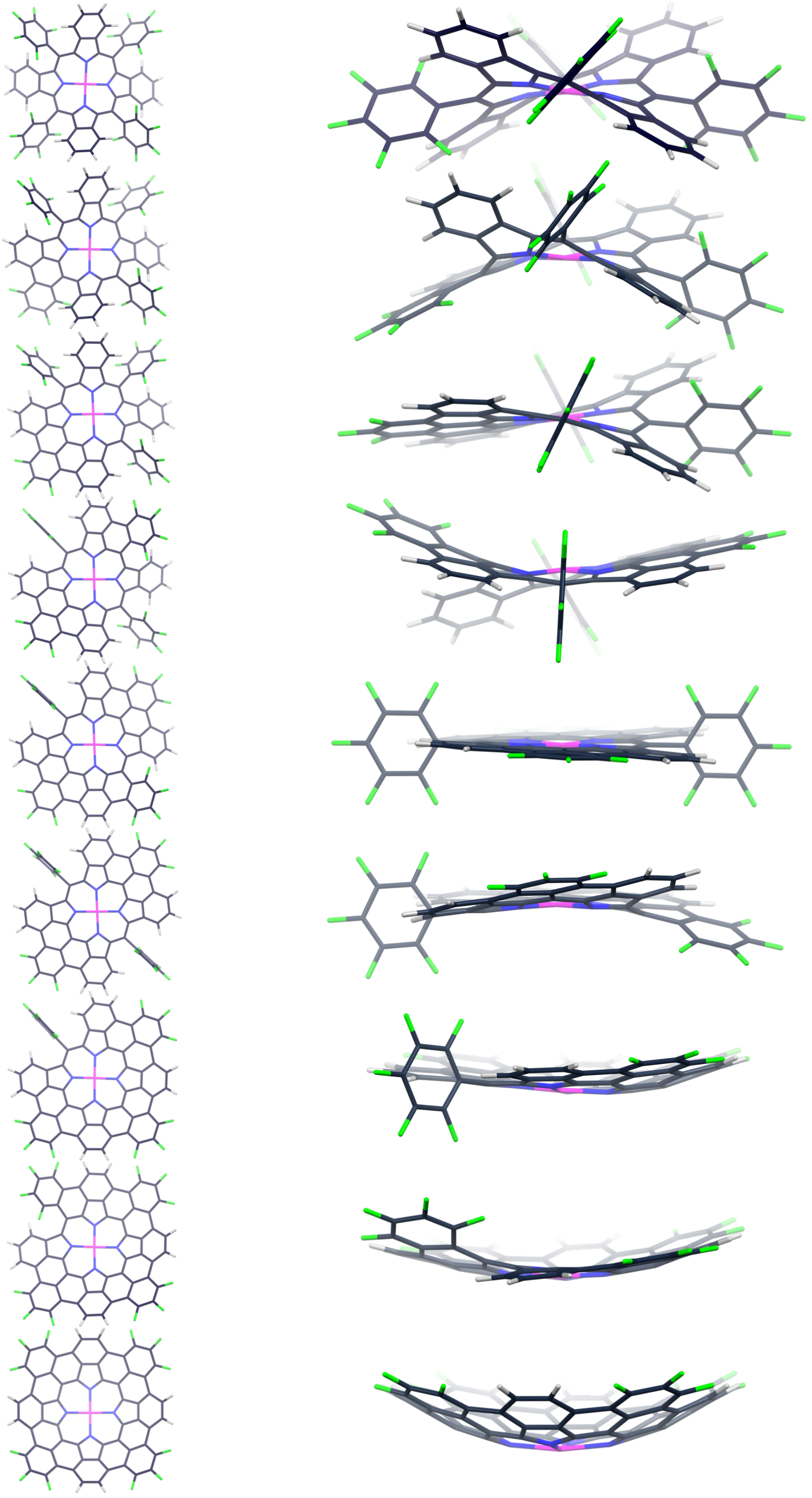

Figure S83. Enlarged geometry optimized structures from Figure 4 of [**1Co**-nHF]$^{+\cdot}$, in top and sideview.

3.5 XYZ Coordinates of geometry optimized structures

[**1**+H]$^+$

E_{abs} = -4513.036710 hartrees

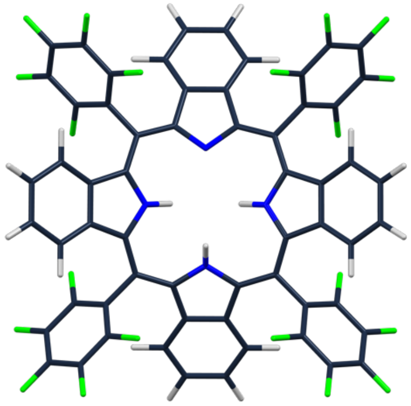

Atom	X	Y	Z
C1	-2.811977	0.356982	1.122067
C2	-3.340693	1.100246	2.225594
C3	-2.358945	1.112076	3.242364
C4	-1.230230	0.372813	2.762511
C5	-0.064703	-0.025837	3.433867
C6	1.115417	-0.411237	2.815459
C7	2.281017	-1.058239	3.379218
C8	3.288616	-1.051131	2.394964
C9	2.743589	-0.395248	1.223369
C10	3.379795	0.026824	0.056642
N11	1.439997	-0.124980	1.509889
C12	2.725357	0.466356	-1.104232
C13	3.380363	1.073641	-2.274948
C14	2.408575	1.085097	-3.280583
C15	1.210496	0.480765	-2.674929
C16	0.074862	0.051230	-3.376589
C17	-1.113859	-0.383488	-2.789646
C18	-2.257817	-1.044743	-3.384228
C19	-3.277253	-1.075007	-2.412413
C20	-2.764867	-0.430567	-1.222383
C21	-3.433048	-0.053277	-0.066768
N22	-1.451068	-0.128223	-1.495825
H23	0.885320	0.428383	0.872166
H24	-0.841958	0.426763	-0.911843
N25	1.412692	0.241045	-1.355341
N26	-1.527353	0.005630	1.469849
H27	-1.671030	-1.681417	-5.375846
C28	-2.449365	-1.660345	-4.627486
H29	-5.268546	-1.787732	-1.926609
C30	-4.490412	-1.722318	-2.673666
H31	-5.602394	-2.812009	-4.130608
C32	-4.670536	-2.306190	-3.909707
H33	-3.822009	-2.756076	-5.835237
C34	-3.657910	-2.274509	-4.879152
H35	-5.293155	1.838603	1.614746
C36	-4.544736	1.807719	2.393598
H37	-1.824554	1.879661	5.205807
C38	-2.576762	1.831519	4.431336
H39	-3.956528	3.053396	5.494958
C40	-3.768909	2.495329	4.585654
H41	-5.669516	3.033836	3.720618
C42	-4.748084	2.483457	3.571724
H43	1.719509	-1.737932	5.362349
C44	2.493111	-1.691466	4.609229
H45	5.290237	-1.718689	1.883639
C46	4.516740	-1.679150	2.636380
H47	5.662942	-2.771802	4.063538
C48	4.719312	-2.280611	3.860527
H49	3.898523	-2.781223	5.785434
C50	3.715646	-2.286367	4.839537
H51	5.381147	1.696866	-1.714962
C52	4.626134	1.657812	-2.486503
H53	1.919864	1.739220	-5.289369
C54	2.667060	1.682385	-4.511147
H55	4.131868	2.724679	-5.670660
C56	3.915314	2.249937	-4.721584
H57	5.845974	2.701590	-3.900108
C58	4.884193	2.237071	-3.720547
C59	-4.915748	-0.053617	-0.085597
C60	-7.708794	-0.038952	-0.095456
C61	-5.624869	0.811086	-0.913340
C62	-5.639870	-0.908131	0.738628
C63	-7.024699	-0.909513	0.742709
C64	-7.009268	0.825450	-0.927858
C65	4.863394	-0.027022	0.050753
C66	7.654182	-0.128842	0.040954
C67	5.614581	0.838575	0.837146
C68	5.542953	-0.946693	-0.739334
C69	6.926806	-1.005171	-0.753850
C70	6.998836	0.797910	0.841389

C71	0.133812	0.018456	-4.859676
C72	0.240218	-0.040812	-7.649362
C73	-0.627414	0.894566	-5.624350
C74	0.947163	-0.891141	-5.525117
C75	1.009847	-0.928140	-6.908334
C76	-0.583478	0.875372	-7.008372
C77	-0.102948	-0.009669	4.916102
C78	-0.196943	0.035383	7.706972
C79	-0.951741	-0.858598	5.618259
C80	0.696287	0.864673	5.645930
C81	0.659220	0.894330	7.029640
C82	-1.007384	-0.844781	7.001823
F83	-4.999061	-1.750044	1.541629
F84	-7.695584	-1.731253	1.532060
F85	-9.027247	-0.032447	-0.101116
F86	-7.666105	1.654408	-1.721501
F87	-4.970912	1.650657	-1.707499
F88	-1.418490	1.777204	-5.022871
F89	-1.315108	1.717908	-7.719459
F90	0.291476	-0.068728	-8.967629
F91	1.791469	-1.798980	-7.525383
F92	1.685614	-1.747856	-4.830152
F93	4.861053	-1.792939	-1.501741
F94	7.557062	-1.885947	-1.513588
F95	8.972863	-0.177055	0.035882
F96	7.696872	1.630174	1.597007
F97	4.999736	1.731196	1.606433
F98	1.512979	1.698632	5.012694
F99	1.426302	1.732305	7.706511
F100	-0.240498	0.056172	9.024497
F101	-1.819521	-1.661119	7.652002
F102	-1.728308	-1.709762	4.957164
H103	-1.054187	-0.768365	1.021283

[**1**-HF+H]$^+$

E_{abs} = -4412.614932 hartrees

Atom	X	Y	Z
C1	-0.238635	-2.845826	1.011023
C2	-0.984914	-3.426068	2.077177
C3	-1.209414	-2.413306	3.040191
C4	-0.544602	-1.229611	2.585089
C5	-0.259345	-0.033717	3.297598
C6	0.179050	1.149985	2.749245
C7	0.749380	2.311157	3.429565
C8	0.952944	3.314120	2.466806
C9	0.492749	2.789613	1.184588
C10	0.334784	3.433506	-0.023538
N11	0.133757	1.486631	1.412375
C12	-0.099366	2.800114	-1.219684
C13	-0.432258	3.505468	-2.481923
C14	-0.764745	2.478288	-3.361246
C15	-0.595070	1.243979	-2.665515
C16	-0.633842	0.074140	-3.400515
C17	-0.043291	-1.126673	-2.887331
C18	0.831609	-2.070226	-3.513797
C19	1.145614	-3.055599	-2.554288
C20	0.460234	-2.705404	-1.340636

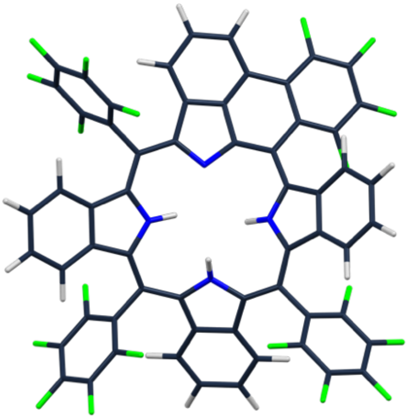

C21	0.253653	-3.415967	-0.168266
N22	-0.173696	-1.500923	-1.606404
H23	-0.311002	0.971135	0.658610
H24	-0.819678	-1.046169	-0.978687
N25	-0.223414	1.472816	-1.357822
N26	-0.051373	-1.513888	1.345456
H27	1.179272	-1.328649	-5.521399
C28	1.402902	-2.098985	-4.793659
H29	2.320500	-4.836245	-2.144773
C30	2.040860	-4.086525	-2.872984
H31	3.271585	-4.901880	-4.407559
C32	2.578895	-4.112874	-4.140362
H33	2.719857	-3.176942	-6.077199
C34	2.264625	-3.126662	-5.095848

H35	-1.398760	-5.481597	1.503023
C36	-1.546311	-4.705851	2.242027
H37	-2.237388	-1.911398	4.893536
C38	-2.013497	-2.679105	4.166982
H39	-3.156703	-4.159539	5.178664
C40	-2.537128	-3.937899	4.318437
H41	-2.735386	-5.929411	3.512132
C42	-2.299443	-4.949027	3.362693
H43	1.047956	1.740448	5.500164
C44	1.160937	2.507232	4.749001
H45	1.752513	5.294364	2.093444
C46	1.559751	4.520316	2.820065
H47	2.415906	5.642207	4.422588
C48	1.943067	4.711183	4.135020
H49	2.069763	3.877229	6.111110
C50	1.746932	3.712459	5.090484
H51	-0.219975	5.665621	-2.347871
C52	-0.454567	4.807485	-2.959980
C53	-1.059268	2.633644	-4.705158
H54	-1.290668	4.185804	-6.204389
C55	-1.068517	3.960519	-5.172907
H56	-0.804463	6.017747	-4.691183
C57	-0.784750	5.005888	-4.304900
C58	0.524296	-4.871572	-0.165312
C59	1.028612	-7.617841	-0.127059
C60	-0.166457	-5.738472	-1.008074
C61	1.471270	-5.414743	0.697986
C62	1.730878	-6.774673	0.723751
C63	0.076352	-7.101345	-0.996692
C64	0.614431	4.890549	-0.076853
C65	1.137431	7.630423	-0.192874
C66	-0.316202	5.815409	0.379287
C67	1.811630	5.368560	-0.594250
C68	2.083767	6.725596	-0.657231
C69	-0.068183	7.177749	0.327844
C70	-1.152289	0.151127	-4.772155
C71	-2.196138	0.228113	-7.386131
C72	-1.605866	-1.004901	-5.424711
C73	-1.337431	1.401325	-5.436226
C74	-1.835688	1.392971	-6.741442
C75	-2.091482	-0.982115	-6.714325
C76	-0.424662	-0.106011	4.770690
C77	-0.767015	-0.291497	7.536309
C78	0.402406	-0.913838	5.542808
C79	-1.428550	0.606612	5.416857
C80	-1.606168	0.524169	6.787990
C81	0.241934	-1.015505	6.914394
F82	2.157841	-4.621002	1.511677
F83	2.639651	-7.271347	1.545568
F84	1.266453	-8.914189	-0.110054
F85	-0.592998	-7.911123	-1.799465
F86	-1.092868	-5.265156	-1.832358
F87	-1.614512	-2.187576	-4.807483
F88	-2.487519	-2.098969	-7.299017
F89	-2.668276	0.260225	-8.618237
F90	-2.012698	2.529485	-7.413916
F91	2.721594	4.511856	-1.044552
F92	3.229256	7.163630	-1.154177
F93	1.385191	8.925599	-0.246521
F94	-0.964115	8.045248	0.769457
F95	-1.470518	5.394690	0.883176
F96	-2.243401	1.385607	4.714547
F97	-2.566273	1.209824	7.386067
F98	-0.928958	-0.378707	8.842318
F99	1.038625	-1.789899	7.631872
F100	1.373574	-1.608423	4.960077
H101	0.685456	-0.966994	0.919164

[**1**-2HF+H]$^+$

E_{abs} = -4312.204021 hartrees

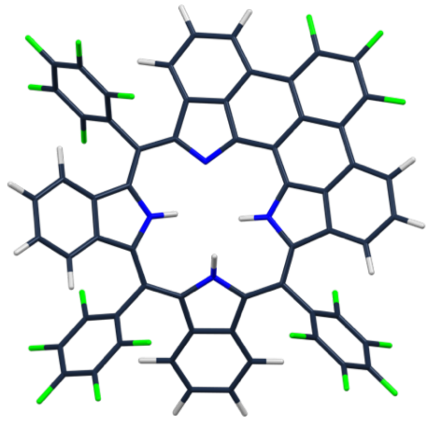

Atom	X	Y	Z
C1	-2.905035	0.224532	0.989645
C2	-3.447828	0.883703	2.133822
C3	-2.438737	0.921419	3.125898
C4	-1.283518	0.262755	2.596618
C5	-0.078939	-0.070627	3.244407
C6	1.144346	-0.363816	2.658895
C7	2.345075	-0.873741	3.295920
C8	3.399506	-0.771330	2.367203
C9	2.840584	-0.246010	1.138225
C10	3.481776	0.078126	-0.065245
N11	1.508398	-0.084690	1.357338
C12	2.833757	0.342301	-1.271657
C13	3.509920	0.582827	-2.571985
C14	2.482059	0.472356	-3.502054
C15	1.275496	0.272531	-2.781271
C16	0.097273	0.084817	-3.511300
C17	-1.134490	-0.099739	-2.906564
C18	-2.281739	-0.419464	-3.642330
C19	-3.363991	-0.565240	-2.765926
C20	-2.847389	-0.249308	-1.437486
C21	-3.519368	-0.061606	-0.248856
N22	-1.485648	-0.029342	-1.600646
H23	0.936771	0.336677	0.635485
H24	-0.856853	0.353504	-0.911120
N25	1.480909	0.237422	-1.447590
N26	-1.597643	-0.085060	1.299447
C27	-2.305122	-0.565442	-5.025571
H28	-5.465031	-1.130525	-2.708522
C29	-4.583897	-0.958858	-3.311044
H30	-5.579898	-1.445955	-5.132690
C31	-4.639481	-1.139400	-4.690298
H32	-3.693532	-1.090249	-6.603382
C33	-3.549392	-0.940428	-5.545031
H34	-5.468750	1.506314	1.612115
C35	-4.691482	1.507182	2.361516
H36	-1.902570	1.683065	5.094452
C37	-2.669423	1.601941	4.338682
H38	-4.085062	2.708448	5.474309
C39	-3.892921	2.185932	4.545163
H40	-5.855260	2.612734	3.755269
C41	-4.903058	2.133846	3.562242
H42	1.760528	-1.604390	5.255847
C43	2.563412	-1.461233	4.548264
H44	5.510212	-1.149120	2.014804
C45	4.685743	-1.211824	2.707641
H46	5.880229	-2.100035	4.233220
C47	4.891351	-1.754935	3.957541
H48	4.016252	-2.347194	5.832056
C49	3.834627	-1.889552	4.867124
H50	5.637336	1.006183	-2.399566
C51	4.783202	0.863371	-3.046300
C52	2.604261	0.537900	-4.878974
H53	4.119575	0.898175	-6.392759
C54	3.904816	0.811414	-5.339301
H55	5.930867	1.208269	-4.818936
C56	4.945052	0.981499	-4.430931
C57	-5.002329	-0.095916	-0.282190
C58	-7.793195	-0.159892	-0.301897
C59	-5.736396	0.899422	-0.918956
C60	-5.698534	-1.121895	0.344972
C61	-7.083572	-1.164661	0.341874
C62	-7.120475	0.877455	-0.936315
C63	4.967503	0.066206	-0.069691
C64	7.758166	0.055519	-0.089948
C65	5.688389	1.143737	0.426104
C66	5.674336	-1.018399	-0.574996
C67	7.058814	-1.035537	-0.591284
C68	7.074677	1.150571	0.421468
C69	0.146501	0.024537	-4.974718
C70	0.241972	-0.063271	-7.766753
C71	-1.036278	-0.293651	-5.714995
C72	1.379794	0.279926	-5.648457
C73	1.379090	0.231649	-7.039802

C74	-0.940332	-0.325752	-7.104152
C75	-0.131439	-0.066247	4.729200
C76	-0.263384	-0.041465	7.517454
C77	-0.901906	-0.996124	5.417060
C78	0.569136	0.879766	5.470020
C79	0.512394	0.900437	6.853167
C80	-0.975152	-0.994067	6.800223
F81	-5.030738	-2.086767	0.966771
F82	-7.728799	-2.149724	0.942690
F83	-9.111399	-0.189365	-0.311953
F84	-7.802824	1.832796	-1.545577
F85	-5.106571	1.902968	-1.519456
F86	-1.997056	-0.608484	-7.860236
F87	0.287229	-0.099921	-9.087299
F88	2.485655	0.462368	-7.739014
F89	5.015345	-2.067603	-1.053172
F90	7.716824	-2.075632	-1.077076
F91	9.077711	0.049962	-0.099497
F92	7.746304	2.185084	0.899100
F93	5.044916	2.194134	0.922259
F94	1.305275	1.793993	4.849158
F95	1.183731	1.807651	7.542399
F96	-0.324988	-0.029777	8.834555
F97	-1.711116	-1.888454	7.437950
F98	-1.582715	-1.916840	4.743565
H99	-1.111800	-0.838867	0.831634

[**1**-3HF+H]$^+$

E_{abs} = -4211.785929 hartrees

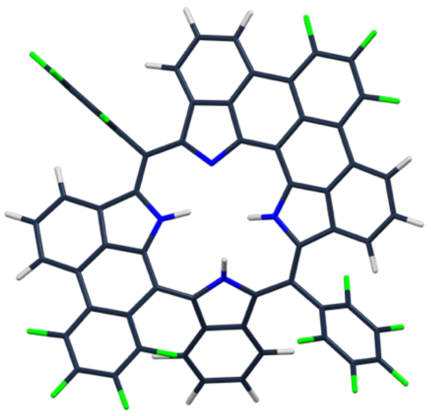

Atom	X	Y	Z
C1	-2.879958	0.280104	1.080805
C2	-3.297985	0.948517	2.265197
C3	-2.306187	0.724953	3.251897
C4	-1.305126	-0.094594	2.676100
C5	-0.077058	-0.558217	3.259856
C6	1.138490	-0.430402	2.627216
C7	2.341236	-0.449853	3.380080
C8	3.411526	-0.080004	2.557143
C9	2.846123	0.094910	1.221745
C10	3.490960	0.326430	0.015035
N11	1.494988	-0.085770	1.339849
C12	2.842312	0.312086	-1.233960
C13	3.523009	0.354338	-2.553062
C14	2.494017	0.128815	-3.464154
C15	1.285672	0.025073	-2.730945
C16	0.105106	-0.186759	-3.427491
C17	-1.148312	-0.240528	-2.806318
C18	-2.315015	-0.521883	-3.525352
C19	-3.414424	-0.475697	-2.656608
C20	-2.879982	-0.125247	-1.354625
C21	-3.522240	0.143564	-0.164990
N22	-1.493757	-0.033497	-1.519169
H23	0.935118	-0.059985	0.491265
H24	-0.856723	0.395446	-0.863199
N25	1.506418	0.146524	-1.389124
N26	-1.649414	-0.289005	1.374380
C27	-2.351779	-0.781447	-4.894667
H28	-5.568036	-0.791707	-2.583170
C29	-4.670293	-0.777479	-3.185700
H30	-5.702324	-1.299794	-4.974054
C31	-4.736434	-1.066081	-4.542002
H32	-3.780854	-1.281347	-6.440267
C33	-3.623239	-1.062223	-5.396396
H34	-5.157775	1.992400	1.816614
C35	-4.397274	1.783869	2.556123
H36	-1.623489	1.189633	5.262733
C37	-2.396860	1.335629	4.519211
H38	-3.581090	2.601336	5.751923
C39	-3.483116	2.125075	4.784202
H40	-5.322432	2.981923	4.046431
C41	-4.480993	2.343863	3.804320

C42	2.424054	-0.648748	4.752956
H43	5.532739	0.385704	2.564991
C44	4.665665	0.083406	3.134364
H45	5.750525	-0.001319	4.973939
C46	4.781495	-0.129077	4.506632
H47	3.863807	-0.613595	6.368635
C48	3.700476	-0.481921	5.310470
H49	5.661407	0.727673	-2.426624
C50	4.803069	0.529150	-3.052211
C51	2.618301	0.023964	-4.837423
H52	4.147431	0.150563	-6.378599
C53	3.929659	0.202151	-5.323617
H54	5.966170	0.592885	-4.849842
C55	4.972458	0.452945	-4.441688
C56	-4.993054	0.326875	-0.192149
C57	-7.762020	0.688620	-0.191092
C58	-5.574861	1.401946	-0.856808
C59	-5.831087	-0.562198	0.472162
C60	-7.205794	-0.393133	0.478639
C61	-6.946415	1.591084	-0.862210
C62	4.966459	0.488729	0.047704
C63	7.741584	0.784922	0.102815
C64	5.547982	1.746122	0.142593
C65	5.805173	-0.617235	-0.017795
C66	7.183622	-0.483926	0.008297
C67	6.924816	1.906499	0.170305
C68	0.142869	-0.382599	-4.880676
C69	0.232789	-0.770817	-7.650310
C70	-1.060372	-0.689683	-5.594399
C71	1.386666	-0.270287	-5.573562
C72	1.383427	-0.470567	-6.954622
C73	-0.966077	-0.878148	-6.968678
C74	-0.032629	-1.022007	4.654662
C75	-0.031899	-1.924466	7.320793
C76	-1.175014	-1.579710	5.247913
C77	1.177206	-1.025613	5.411980
C78	1.128915	-1.459049	6.739730
C79	-1.191847	-1.997330	6.561468
F80	-5.313895	-1.602339	1.114869
F81	-7.986868	-1.251001	1.112597
F82	-9.069200	0.859937	-0.191150
F83	-7.481719	2.621240	-1.496194
F84	-4.809031	2.279725	-1.494771
F85	-2.036656	-1.169161	-7.700637
F86	0.271223	-0.956137	-8.958440
F87	2.503293	-0.384203	-7.665670
F88	5.281137	-1.834246	-0.108828
F89	7.967713	-1.547599	-0.056392
F90	9.053480	0.925092	0.129177
F91	7.463102	3.111661	0.261338
F92	4.776622	2.825015	0.209432
F93	2.224941	-1.468584	7.496900
F94	-0.038288	-2.330867	8.575999
F95	-2.295212	-2.495714	7.089189
F96	-2.299546	-1.754231	4.554914
H97	-1.271603	-1.053362	0.832050

[**1**-4HF+H]$^+$

E_{abs} = -4111.375028 hartrees

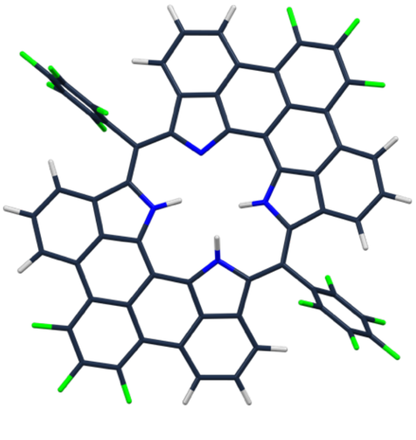

Atom	X	Y	Z
C1	1.175334	-0.008619	-2.955063
C2	2.472749	-0.251047	-3.532152
C3	3.392985	-0.156940	-2.474365
C4	2.712396	0.095953	-1.285542
C5	3.372780	0.130708	-0.058066
C6	2.742025	0.158124	1.180932
C7	3.492040	0.217452	2.367202
C8	2.625296	0.199660	3.469142
C9	1.288606	0.094054	2.910979
C10	0.056794	-0.040724	3.543103
N11	1.426268	0.100834	1.545722

C12	-1.176225	-0.144336	2.884755
C13	-2.490936	-0.240046	3.549817
C14	-3.401788	-0.199146	2.497583
C15	-2.662028	-0.125095	1.287888
C16	-3.372672	-0.052840	0.080327
C17	-2.769003	0.007485	-1.171739
C18	-3.504499	0.217477	-2.342918
C19	-2.630555	0.282297	-3.438083
C20	-1.300003	0.074149	-2.900267
C21	-0.080071	0.030057	-3.563972
N22	-1.456627	-0.073123	-1.527786
H23	0.580595	-0.013991	0.989953
H24	-0.774043	-0.477912	-0.905448
N25	-1.327347	-0.106580	1.525922
N26	1.376989	0.184573	-1.590793
C27	-4.890583	0.346161	-2.388010
H28	-2.586331	0.628438	-5.590083
C29	-3.184098	0.540187	-4.694381
H30	-5.010798	0.879604	-5.736071
C31	-4.562578	0.684150	-4.769270
H32	-6.473863	0.702029	-3.819333
C33	-5.413928	0.585052	-3.660795
H34	2.338087	-0.687169	-5.666331
C35	2.974723	-0.569991	-4.801206
C36	4.778375	-0.307559	-2.575007
H37	6.294379	-0.739520	-4.051650
C38	5.243311	-0.601659	-3.853896
H39	4.749928	-0.976323	-5.899943
C40	4.342866	-0.735016	-4.925383
C41	4.882338	0.269540	2.425284
H42	2.590814	0.275899	5.645016
C43	3.186607	0.276390	4.743810
H44	5.025048	0.427568	5.812938
C45	4.570259	0.360547	4.832089
H46	6.478105	0.406338	3.880283
C47	5.413839	0.353034	3.715886
H48	-2.362021	-0.395069	5.718021
C49	-2.991414	-0.345814	4.841576
C50	-4.781531	-0.222897	2.612209
H51	-6.323184	-0.370237	4.133858
C52	-5.265869	-0.334051	3.926446
H53	-4.786001	-0.487594	5.995923
C54	-4.378118	-0.397977	4.996192
C55	-0.109173	0.015595	-5.048019
C56	-0.154190	-0.013042	-7.837608
C57	-0.452497	-1.136926	-5.744092
C58	0.212127	1.152893	-5.778231
C59	0.192967	1.151034	-7.163578
C60	-0.478654	-1.163037	-7.129000
C61	0.075804	-0.068509	5.028284
C62	0.121944	-0.125653	7.817066
C63	0.232375	-1.265528	5.714149
C64	-0.052444	1.100063	5.767088
C65	-0.032537	1.084627	7.152718
C66	0.256291	-1.307188	7.099106
C67	-4.842407	0.015617	0.112314
C68	-7.643129	0.107254	0.175643
C69	-5.580201	0.201500	-1.101364
C70	-5.532105	-0.099339	1.358893
C71	-6.924085	-0.055508	1.342060
C72	-6.970090	0.239522	-1.021179
C73	4.842324	0.064492	-0.081659
C74	7.639506	0.010283	-0.137125
C75	5.525528	-0.127043	-1.323997
C76	5.578988	0.181112	1.137384
C77	6.970449	0.161284	1.059847
C78	6.917349	-0.140516	-1.304095
F79	0.548627	2.269544	-5.143257
F80	0.501783	2.241824	-7.843951
F81	-0.175689	-0.026671	-9.156000
F82	-0.808900	-2.267421	-7.776662
F83	-0.766539	-2.240592	-5.076372
F84	-7.719855	0.402391	-2.107485
F85	-8.963489	0.143361	0.205470
F86	-7.634174	-0.164469	2.460485
F87	-0.200326	2.261417	5.140417
F88	-0.158974	2.206164	7.842815
F89	0.142882	-0.152702	9.136219
F90	0.405720	-2.455776	7.738164
F91	0.361780	-2.400010	5.036130
F92	7.723904	0.280884	2.148180
F93	8.959816	-0.001706	-0.165509
F94	7.621045	-0.296875	-2.420772
H95	0.740839	0.751165	-1.047782

[**1**-5HF+H]$^+$

E_{abs} = -4010.940138 hartrees

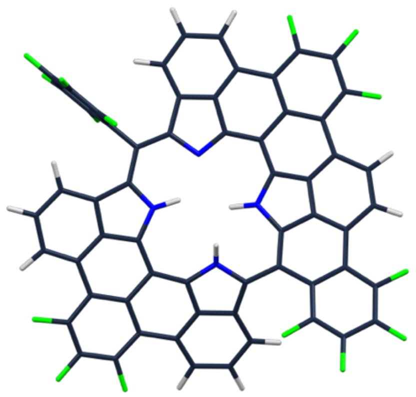

Atom	X	Y	Z
C1	3.150811	-1.250556	0.279606
C2	3.654367	-2.464038	-0.265485
C3	2.572337	-3.364084	-0.288480
C4	1.421970	-2.724330	0.152834
C5	0.166584	-3.343262	0.093525
C6	-1.069553	-2.714905	0.213244
C7	-2.254928	-3.461850	0.258891
C8	-3.353977	-2.595124	0.369476
C9	-2.802073	-1.260510	0.316896
C10	-3.461123	-0.014275	0.210387
N11	-1.445607	-1.390794	0.270737
C12	-2.819993	1.194460	0.006103
C13	-3.457352	2.526000	-0.160229
C14	-2.381180	3.396805	-0.312518
C15	-1.194839	2.621331	-0.241744
C16	0.039373	3.289543	-0.310970
C17	1.239562	2.629870	-0.167243
C18	2.433953	3.350775	-0.086370
C19	3.453802	2.454079	0.170407
C20	2.902514	1.158195	0.230323
C21	3.711617	0.061484	0.444156
N22	1.539211	1.304498	-0.003641
H23	-0.903866	-0.537492	0.142300
H24	0.877040	0.580613	-0.221204
N25	-1.446684	1.318953	-0.065030
N26	1.796675	-1.442454	0.491355
C27	2.593323	4.711399	-0.230566
C28	4.775361	2.819469	0.274473
H29	5.990684	4.617033	0.158060
C30	4.989818	4.215062	0.101806
H31	4.216325	6.160865	-0.238555
C32	3.951281	5.119272	-0.132441
H33	5.741700	-2.248540	-0.868238
C34	4.877406	-2.896946	-0.809867
C35	2.627082	-4.691172	-0.726458
H36	4.013015	-6.095231	-1.594455
C37	3.857754	-5.093206	-1.226165
H38	5.877490	-4.548263	-1.696196
C39	4.944827	-4.190687	-1.277167
C40	-2.325761	-4.851254	0.178830
H41	-5.523009	-2.563340	0.579686
C42	-4.628800	-3.160876	0.474265
H43	-5.699341	-5.001947	0.537712
C44	-4.722276	-4.543188	0.448119
H45	-3.779701	-6.448712	0.256909
C46	-3.611051	-5.384287	0.293320
H47	-5.631110	2.477244	-0.079541
C48	-4.733209	3.068792	-0.192044
C49	-2.436846	4.769540	-0.484727
H50	-3.917070	6.351370	-0.655365
C51	-3.740996	5.295500	-0.522760
H52	-5.828192	4.894001	-0.411138
C53	-4.839422	4.452050	-0.379578
C54	5.119564	0.383198	0.743173
C55	7.845459	0.906919	1.313341
C56	5.663539	1.710609	0.596062
C57	5.966401	-0.580145	1.312070
C58	7.302464	-0.347452	1.560864
C59	7.022563	1.911255	0.854418
C60	-4.944592	-0.031530	0.279721
C61	-7.731202	-0.064564	0.415621
C62	-5.713114	-0.174301	-0.868494
C63	-5.600523	0.092624	1.497208
C64	-6.984098	0.078688	1.577652
C65	-7.097536	-0.192034	-0.814102
C66	0.084147	4.746498	-0.443831
C67	0.150364	7.527073	-0.768446
C68	1.343548	5.440620	-0.442660
C69	-1.143108	5.471889	-0.570173
C70	-1.059429	6.849604	-0.737007
C71	1.324787	6.825511	-0.612733
C72	0.163112	-4.791076	-0.185506
C73	0.171524	-7.552214	-0.648472
C74	1.376558	-5.446433	-0.573050

C75	-1.055082	-5.533618	-0.085120
C76	-0.999403	-6.908303	-0.307767
C77	1.334272	-6.819627	-0.787546
F78	5.499254	-1.775101	1.673123
F79	8.058880	-1.304027	2.067860
F80	9.122202	1.134341	1.554223
F81	7.560981	3.118132	0.692204
F82	2.459362	7.517045	-0.626997
F83	0.169205	8.838746	-0.931796
F84	-2.150422	7.594683	-0.874056
F85	-4.894778	0.226899	2.614209
F86	-7.594113	0.199464	2.745298
F87	-9.049182	-0.079971	0.479836
F88	-7.815561	-0.329280	-1.916799
F89	-5.115835	-0.298107	-2.048101
F90	-2.087882	-7.666792	-0.214635
F91	0.179496	-8.856730	-0.851865
F92	2.426169	-7.495953	-1.131483
H93	1.284086	-0.883427	1.158512

[**1**-6HF+H]$^+$

E_{abs} = -3910,505768 hartrees

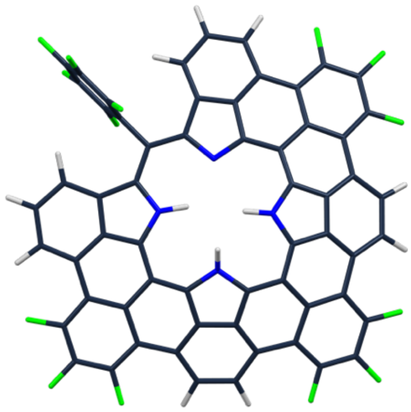

Atom	X	Y	Z
C1	-1.150462	-1.125272	3.007825
C2	-0.728319	-2.282616	3.645884
C3	-0.625919	-3.280265	2.684940
C4	-0.957352	-2.725040	1.451561
C5	-0.615439	-3.369814	0.281050
C6	-0.552913	-2.748675	-0.968065
C7	-0.300816	-3.496754	-2.118267
C8	-0.305622	-2.642030	-3.234760
C9	-0.495487	-1.302779	-2.704430
C10	-0.400654	-0.049002	-3.329254
N11	-0.674054	-1.443563	-1.349613
C12	-0.408462	1.195324	-2.689667
C13	-0.108627	2.502228	-3.324250
C14	-0.054868	3.395080	-2.254353
C15	-0.377036	2.671266	-1.082926
C16	-0.418469	3.381270	0.133339
C17	-0.791732	2.798331	1.320406
C18	-0.579820	3.436682	2.546208
C19	-0.726866	2.484246	3.543695
C20	-1.065588	1.278732	2.924627
C21	-1.017977	0.098818	3.638789
N22	-1.159226	1.505518	1.574928
H23	-0.669768	-0.602924	-0.779006
H24	-1.153153	0.832675	0.824594
N25	-0.590368	1.365181	-1.334521
N26	-1.332203	-1.407178	1.666427
C27	-0.012337	4.679162	2.730222
C28	-0.291135	2.645398	4.846085
H29	0.564103	4.217679	6.076399
C30	0.207494	3.950801	5.091522
H31	0.775355	5.868935	4.368473
C32	0.336169	4.923234	4.083699
C33	-0.221926	-2.378641	4.934451
C34	-0.035469	-4.511750	2.907774
H35	0.854865	-5.607563	4.549952
C36	0.393172	-4.679915	4.242005
H37	0.718024	-3.874503	6.188882
C38	0.308256	-3.656579	5.212777
C39	-0.050692	-4.872841	-2.121880
H40	-0.126282	-2.661157	-5.408381
C41	-0.116310	-3.231702	-4.490221
H42	0.225685	-5.075608	-5.497156
C43	0.083673	-4.603305	-4.532480
H44	0.325765	-6.474139	-3.523823
C45	0.133351	-5.420851	-3.389971
H46	0.091982	2.416829	-5.492892
C47	0.128332	3.017212	-4.595039
C48	0.257157	4.747596	-2.307470
H49	0.732228	6.274912	-3.773623

C50	0.490664	5.237890	-3.600547
H51	0.591856	4.796431	-5.682798
C52	0.413870	4.379994	-4.698506
C53	-0.519559	0.132349	5.010045
C54	0.407384	0.191867	7.642017
C55	-0.243674	1.399743	5.632883
C56	-0.202357	-1.103746	5.675817
C57	0.235375	-1.020080	6.994605
C58	0.191492	1.374227	6.956280
C59	-0.185853	-0.077614	-4.800365
C60	0.213984	-0.145089	-7.560352
C61	1.094284	-0.152967	-5.333903
C62	-1.261563	-0.040355	-5.676512
C63	-1.075609	-0.072377	-7.050010
C64	1.306049	-0.186031	-6.702520
C65	0.041065	4.767552	0.203029
C66	0.913460	7.418320	0.308967
C67	0.219855	5.412048	1.479449
C68	0.342207	5.454512	-1.013963
C69	0.763859	6.775821	-0.911410
C70	0.653578	6.735566	1.478375
C71	-0.159841	-4.757319	0.397108
C72	0.679123	-7.410716	0.600009
C73	0.096389	-5.326923	1.693141
C74	0.063239	-5.512619	-0.796193
C75	0.466277	-6.836260	-0.643243
C76	0.509966	-6.654923	1.742122
F77	0.523008	-2.121864	7.676075
F78	0.817977	0.219444	8.897768
F79	0.436811	2.507177	7.603100
F80	0.827074	7.384369	2.625596
F81	1.316331	8.676521	0.345731
F82	1.056463	7.492489	-1.991123
F83	-2.498660	0.028443	-5.198626
F84	-2.111090	-0.035091	-7.872474
F85	0.403135	-0.176718	-8.866052
F86	2.532103	-0.256940	-7.195303
F87	2.143647	-0.194582	-4.520846
F88	0.681715	-7.614937	-1.697876
F89	1.062054	-8.672093	0.685804
F90	0.751212	-7.237749	2.911158
H91	-2.007410	-0.940414	1.079078

[**1**-7HF+H]$^{+}$

E_{abs} = -3810.070211 hartrees

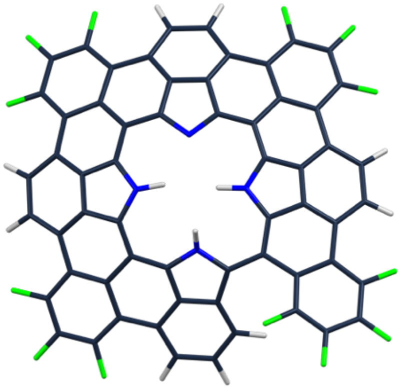

Atom	X	Y	Z
C1	-1.143154	0.663452	-2.978251
C2	-0.598619	-0.059659	-4.078183
C3	0.804868	-0.012585	-3.935481
C4	1.133562	0.673155	-2.776796
C5	2.456455	0.672688	-2.280749
C6	2.832460	1.048606	-1.002711
C7	4.140435	0.800663	-0.545429
C8	4.149597	0.915547	0.838827
C9	2.853613	1.238608	1.228249
C10	2.463845	1.103089	2.555444
N11	2.087325	1.394868	0.116405
C12	1.125149	1.155239	2.892270
C13	0.754033	0.762029	4.201276
C14	-0.617107	0.616850	4.168539
C15	-0.995551	0.914836	2.835093
C16	-2.284966	0.583463	2.445414
C17	-2.665882	0.658372	1.120468
C18	-3.961787	0.324193	0.741785
C19	-4.033141	0.442972	-0.636210
C20	-2.751876	0.804522	-1.122895
C21	-2.486796	0.764732	-2.480754
N22	-1.948238	0.948796	0.000548
H23	1.087469	1.491070	0.227719
H24	-0.949665	1.058539	0.058538

N25	0.051121	1.286545	2.063076	C58	5.306832	-0.316095	5.375738
N26	-0.058795	1.086287	-2.213536	C59	4.791903	0.374214	3.099730
C27	-4.941490	-0.198784	1.565068	C60	3.004404	0.261730	4.848994
C28	-5.133116	0.036675	-1.357420	C61	3.979668	-0.176877	5.741175
H29	-7.102655	-0.806122	-1.014571	C62	5.690457	-0.063707	4.068632
C30	-6.187976	-0.466451	-0.550309	C63	-3.215661	0.004009	3.403467
H31	-6.954096	-0.998214	1.359752	C64	-4.988139	-1.134422	5.235642
C32	-6.102559	-0.578285	0.844200	C65	-4.541993	-0.351360	2.975099
C33	-1.116148	-0.827127	-5.140330	C66	-2.766321	-0.276140	4.742078
C34	1.734798	-0.624187	-4.790823	C67	-3.690761	-0.834373	5.618734
H35	1.804455	-1.828085	-6.575636	C68	-5.391968	-0.902694	3.932755
C36	1.183482	-1.324261	-5.851648	C69	3.500019	0.055690	-3.120190
H37	-0.599862	-2.006116	-6.827636	C70	5.498370	-0.976931	-4.798433
C38	-0.220038	-1.423889	-5.996665	C71	3.146801	-0.493333	-4.395530
C39	5.179496	0.235895	-1.254589	C72	4.853662	-0.062415	-2.647843
C40	5.156200	0.456924	1.672340	C73	5.816746	-0.553906	-3.527710
H41	7.150735	-0.381651	1.480665	C74	4.174113	-0.962516	-5.205880
C42	6.279581	-0.017768	0.955193	F75	-4.661504	1.281910	-6.767431
H43	7.168071	-0.551999	-0.897915	F76	-6.990272	0.275266	-5.818001
C44	6.287654	-0.118554	-0.445321	F77	-7.231484	-0.330396	-3.229600
C45	1.569906	0.263986	5.186382	F78	-6.636015	-1.238992	3.613452
C46	-1.361537	-0.030921	5.122117	F79	-5.832124	-1.658523	6.108407
H47	-1.029025	-0.955214	7.070896	F80	-3.348211	-1.105712	6.871762
C48	-0.565636	-0.456414	6.231065	F81	3.653174	-0.492114	6.988902
H49	1.342164	-0.723446	7.119751	F82	6.199935	-0.726597	6.260106
C50	0.826031	-0.318308	6.260477	F83	6.963984	-0.275707	3.759285
C51	-3.700818	0.641422	-3.318346	F84	7.088979	-0.638650	-3.150376
C52	-5.951389	0.404216	-5.015686	F85	6.436639	-1.426022	-5.611876
C53	-4.974600	0.217337	-2.796120	F86	3.927056	-1.439307	-6.421901
C54	-3.671224	1.048320	-4.658515	H87	-0.125971	1.913316	-1.638625
C55	-4.753320	0.909033	-5.503825	H88	-2.178782	-0.957039	-5.293075
C56	-6.048003	0.082893	-3.678549	F89	-2.583823	1.618916	-5.175022
C57	3.436854	0.610992	3.520876				

[**1**-8HF+H]$^{+}$

E_{abs} = -3709.642174 hartrees

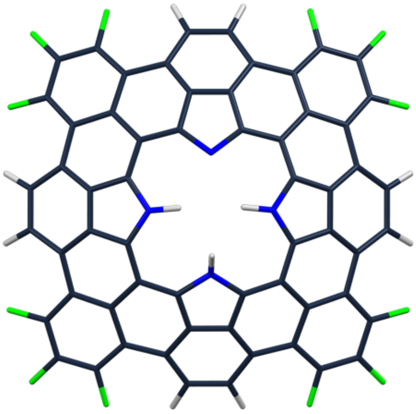

Atom	X	Y	Z
C1	-1.258463	-1.121990	-2.848447
C2	-0.685439	-0.693409	-4.038292
C3	-0.685434	0.696818	-4.043257
C4	-1.262977	1.133318	-2.856576
C5	-1.059890	2.443794	-2.422398
C6	-1.252888	2.846812	-1.108484
C7	-0.807854	4.106931	-0.684145
C8	-0.789266	4.106388	0.707243
C9	-1.213299	2.846143	1.127632
C10	-0.982449	2.412274	2.419279
N11	-1.561698	2.125005	0.023375
C12	-1.130329	1.075085	2.777724
C13	-0.658919	0.690475	4.058709
C14	-0.667839	-0.684641	4.059638
C15	-1.133378	-1.069810	2.779421
C16	-0.980545	-2.414996	2.428427
C17	-1.212519	-2.850658	1.136814
C18	-0.793673	-4.110898	0.710697
C19	-0.812227	-4.104279	-0.677878
C20	-1.252998	-2.839130	-1.098744
C21	-1.056330	-2.436786	-2.413551
N22	-1.560951	-2.116834	0.041907
H23	-1.692232	1.127652	0.114650
H24	-1.682354	-1.118497	0.142362
N25	-1.457444	-0.000318	2.002951
N26	-1.659793	0.005505	-2.145122
C27	-0.140675	-5.049747	1.487603
C28	-0.174747	-5.054017	-1.455438
H29	0.907013	-6.914992	-1.182304
C30	0.394836	-6.101205	-0.688813
H31	0.934003	-6.915244	1.200982
C32	0.412211	-6.101704	0.717213
C33	0.003673	-1.471549	-4.960298
C34	0.005356	1.472028	-4.965699
H35	1.170418	1.190804	-6.766611
C36	0.621086	0.706136	-5.972006
H37	1.167397	-1.198149	-6.762935
C38	0.619427	-0.709802	-5.969666
C39	-0.169179	5.056549	-1.461024
C40	-0.135810	5.047288	1.481335
H41	0.934659	6.914563	1.197105
C42	0.414848	6.100633	0.712311
H43	0.910332	6.918214	-1.185556
C44	0.398934	6.102820	-0.693935
C45	-0.041552	1.469468	5.007403
C46	-0.052837	-1.466110	5.010131
H47	0.988791	-1.192766	6.903640
C48	0.502009	-0.699207	6.074349
H49	0.997524	1.191744	6.902223
C50	0.507530	0.700773	6.073389
C51	-0.360784	-3.357967	-3.316872
C52	0.860960	-5.150790	-5.086903
C53	-0.023734	-4.683540	-2.870304
C54	0.070483	-2.900857	-4.612709
C55	0.645962	-3.836481	-5.465724
C56	0.552079	-5.547935	-3.799685
C57	-0.321900	3.341293	3.339464
C58	0.901815	5.131060	5.114098
C59	0.020551	4.667786	2.895244
C60	0.072564	2.892570	4.648451
C61	0.654373	3.825436	5.501394
C62	0.605303	5.526770	3.822694
C63	-0.327470	-3.342711	3.348376
C64	0.870099	-5.135771	5.130447
C65	0.012178	-4.670127	2.902953
C66	0.058572	-2.892159	4.658459
C67	0.626669	-3.828296	5.516711
C68	0.585322	-5.531005	3.835931
C69	-0.358217	3.361886	-3.324565
C70	0.882022	5.146608	-5.088939
C71	0.075376	2.901743	-4.618757
C72	-0.016260	4.685721	-2.876312
C73	0.569740	5.545906	-3.802930

C74	0.659766	3.834101	-5.469372
F75	1.029115	-3.484144	-6.687584
F76	1.397164	-6.007446	-5.937389
F77	0.841648	-6.799152	-3.460859
F78	0.893030	-6.777993	3.496851
F79	1.405290	-5.991420	5.984084
F80	0.973900	-3.485524	6.751644
F81	1.012791	3.479035	6.732211
F82	1.452136	5.983183	5.961377
F83	0.916267	6.772133	3.480599
F84	0.867969	6.794795	-3.462784
F85	1.429475	5.999525	-5.936042
F86	1.046362	3.479150	-6.689477
H87	-2.444673	0.004134	-1.511115

[1Co]$^{+\bullet}$

E_{abs} = -5894.080534 hartrees

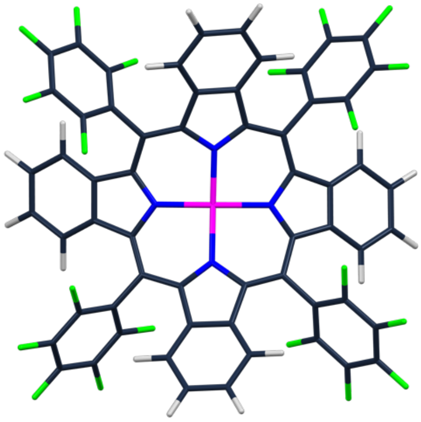

Atom	X	Y	Z
C1	1.065921	-2.718418	0.262436
C2	2.168337	-3.408804	0.948831
C3	3.112188	-2.421615	1.242757
C4	2.600187	-1.188339	0.642572
C5	3.317067	-0.085253	0.291903
C6	2.714641	1.060458	-0.306327
C7	3.422180	2.161403	-0.966814
C8	2.465715	3.159175	-1.175447
C9	1.219096	2.646906	-0.597657
C10	0.083607	3.340670	-0.309232
N11	1.411747	1.324042	-0.206411
C12	-1.066389	2.717525	0.261311
C13	-2.169032	3.408277	0.947007
C14	-3.113176	2.421308	1.240690
C15	-2.600903	1.187640	0.641464
C16	-3.317789	0.084240	0.291480
C17	-2.715430	-1.062197	-0.305324
C18	-3.422900	-2.163595	-0.965273
C19	-2.466194	-3.161152	-1.174039
C20	-1.219602	-2.648583	-0.596384
C21	-0.083857	-3.341914	-0.308360
N22	-1.412502	-1.325634	-0.205219
N23	-1.288039	1.406985	0.211584
N24	1.287427	-1.407990	0.212279
H25	-5.469775	-1.540713	-1.312985
C26	-4.721049	-2.308355	-1.443133
H27	-2.052009	-5.077314	-2.092614
C28	-2.785469	-4.312503	-1.881798
H29	-4.350709	-5.343115	-2.916152
C30	-4.084156	-4.455526	-2.355386
H31	-6.043837	-3.598297	-2.524403
C32	-5.041340	-3.468699	-2.135539
H33	1.626765	-5.492984	1.202362
C34	2.346379	-4.712302	1.398476
H35	4.950346	-1.944607	2.278368
C36	4.236075	-2.708600	2.005131
H37	5.275785	-4.258720	3.055640
C38	4.411311	-4.012999	2.451096
H39	3.634829	-6.011460	2.509155
C40	3.481708	-5.002794	2.145588
H41	5.468698	1.537660	-1.314891
C42	4.720259	2.305638	-1.444996
H43	2.051902	5.075500	-2.093854
C44	2.785145	4.310404	-1.883344
H45	4.350592	5.340441	-2.918059
C46	4.083850	4.452955	-2.357238
H47	6.043155	3.595088	-2.526778
C48	5.040762	3.465811	-2.137622
H49	-1.627493	5.492518	1.200362
C50	-2.347279	4.711942	1.396090
H51	-4.952062	1.944813	2.275271
C52	-4.237528	2.708634	2.002241
H53	-5.277836	4.259104	3.051545

C54	-4.412981	4.013127	2.447637
H55	-3.636338	6.011578	2.505338
C56	-3.483073	5.002721	2.142341
C57	-0.027430	-4.802364	-0.556846
C58	0.126013	-7.550991	-1.018787
C59	-0.775094	-5.697109	0.200705
C60	0.801219	-5.316380	-1.545818
C61	0.886259	-6.678357	-1.785598
C62	-0.709213	-7.062199	-0.021607
C63	0.029216	4.801698	-0.554863
C64	-0.120807	7.551972	-1.007914
C65	0.778234	5.693085	0.205340
C66	-0.798741	5.319873	-1.542223
C67	-0.882167	6.682780	-1.777536
C68	0.714106	7.058920	-0.012566
C69	-4.781225	0.054544	0.529591
C70	-7.535335	-0.038037	0.971155
C71	-5.324183	-0.796803	1.483272
C72	-5.649626	0.855682	-0.203309
C73	-7.017297	0.820766	0.009701
C74	-6.689341	-0.852058	1.712532
C75	4.780109	-0.053919	0.532244
C76	7.533188	0.042612	0.979587
C77	5.650912	-0.855376	-0.197327

C78	5.320232	0.799796	1.485464
C79	6.684826	0.857195	1.717482
C80	7.018073	-0.818592	0.018660
F81	-4.521740	-1.583643	2.191849
F82	-7.188406	-1.668853	2.624891
F83	-8.837210	-0.080056	1.179753
F84	-7.829932	1.591975	-0.693834
F85	-5.168365	1.673725	-1.132284
F86	-1.571190	-5.244279	1.162185
F87	-1.429601	-7.899935	0.705855
F88	0.196408	-8.850082	-1.237161
F89	1.680777	-7.148897	-2.732079
F90	1.538141	-4.488801	-2.278765
F91	5.172611	-1.675630	-1.125947
F92	7.833015	-1.590298	-0.681638
F93	8.834569	0.086545	1.190946
F94	7.181046	1.676744	2.628972
F95	4.515470	1.586886	2.190984
F96	1.573708	5.236154	1.165420
F97	1.435615	7.893366	0.717561
F98	-0.189885	8.851867	-1.221656
F99	-1.676531	7.157432	-2.722138
F100	-1.536977	4.495644	-2.277578
Co101	-0.000380	-0.000688	-0.082356

[**1Co**-HF]$^{+\bullet}$

E_{abs} = -5793.662123 hartrees

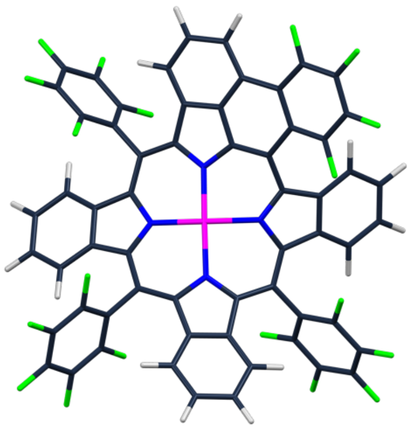

Atom	X	Y	Z
C1	-2.821777	-1.094762	-0.152324
C2	-3.598748	-2.079957	0.603091
C3	-2.667965	-2.958800	1.160883
C4	-1.362835	-2.529539	0.647765
C5	-0.228355	-3.262980	0.527118
C6	0.953888	-2.742914	-0.100632
C7	2.042245	-3.532633	-0.677207
C8	3.012398	-2.608659	-1.084994
C9	2.520082	-1.296574	-0.652782
C10	3.213032	-0.130575	-0.537585
N11	1.219189	-1.443736	-0.174525
C12	2.654853	1.058289	0.037582
C13	3.426223	2.168084	0.614847
C14	2.481755	3.088939	1.080533
C15	1.175588	2.571830	0.670193
C16	0.016080	3.286057	0.567696
C17	-1.187136	2.736145	0.052094

C18	-2.395174	3.480476	-0.361305	C59	-5.223248	-0.933674	-2.055506
C19	-3.266194	2.492791	-0.816457	C60	-6.438193	-0.876876	-2.700734
C20	-2.642808	1.241778	-0.619472	C61	-6.487256	1.490266	-2.347080
C21	-3.339539	0.087090	-0.802860	C62	4.639234	-0.093621	-0.945475
N22	-1.364433	1.421795	-0.106628	C63	7.316341	0.018729	-1.726954
N23	1.348070	1.280307	0.157056	C64	5.626724	-0.734628	-0.206011
N24	-1.526471	-1.289503	0.003418	C65	5.023220	0.608605	-2.079565
H25	-2.235427	5.627267	-0.059761	C66	6.349043	0.671354	-2.478841
C26	-2.837998	4.792964	-0.390158	C67	6.957286	-0.688820	-0.585752
C27	-4.558473	2.680447	-1.271920	C68	-0.003050	4.724992	0.930153
H28	-5.979269	4.285103	-1.633032	C69	-0.076977	7.432129	1.599077
C29	-4.989531	4.022435	-1.292142	C70	-0.578968	5.150093	2.119539
H30	-4.495009	6.053154	-0.893938	C71	0.527472	5.684714	0.077184
C31	-4.136241	5.031339	-0.865512	C72	0.498915	7.031486	0.400006
H32	-5.670422	-1.465037	0.512336	C73	-0.619614	6.491459	2.465034
C33	-4.947455	-2.161971	0.916524	C74	-0.201310	-4.664889	1.009252
H34	-2.363999	-4.612857	2.521863	C75	-0.111229	-7.301064	1.924262
C35	-3.071569	-3.946273	2.048174	C76	-0.937134	-5.665133	0.382676
H36	-4.762659	-4.791914	3.053553	C77	0.586067	-5.017772	2.097457
C37	-4.425420	-4.036436	2.354252	C78	0.638717	-6.322077	2.561970
H38	-6.398191	-3.245472	2.061527	C79	-0.902753	-6.975005	0.829743
C39	-5.352006	-3.158675	1.795456	F80	-1.106863	4.256352	2.948701
H40	1.455493	-5.619362	-0.641195	F81	-1.170084	6.879776	3.603278
C41	2.194471	-4.890860	-0.940603	F82	-0.108869	8.712316	1.916482
H42	4.862620	-2.326593	-2.173486	F83	1.013280	7.933334	-0.419497
C43	4.128145	-3.025328	-1.800230	F84	1.080241	5.312620	-1.070722
H44	5.132895	-4.723500	-2.628327	F85	-7.125110	2.640986	-2.552546
C45	4.272678	-4.381423	-2.065737	F86	-8.244589	0.410961	-3.460178
H46	3.459418	-6.356218	-1.853458	F87	-6.978002	-1.967143	-3.213863
C47	3.324292	-5.304589	-1.631822	F88	-4.606311	-2.114770	-2.012519
H48	5.540782	1.694903	0.493217	F89	-1.686091	-5.371046	-0.674022
C49	4.784325	2.383267	0.835919	F90	-1.611046	-7.913501	0.223410
H50	2.153969	4.906493	2.210942	F91	-0.070807	-8.546278	2.357148
C51	2.872507	4.212071	1.800866	F92	1.392646	-6.638060	3.601158
H52	4.550879	5.281939	2.587852	F93	1.312136	-4.088348	2.708457
C53	4.227357	4.417083	2.021590	F94	5.298101	-1.404943	0.892189
H54	6.224720	3.696097	1.716107	F95	7.884417	-1.307305	0.127161
C55	5.171891	3.518127	1.534578	F96	8.581759	0.069390	-2.096143
C56	-4.628611	0.196569	-1.480385	F97	6.695728	1.344179	-3.562907
C57	-7.083275	0.347662	-2.837351	F98	4.105356	1.242581	-2.800623
C58	-5.253204	1.466649	-1.690092	Co99	-0.068830	0.008621	-0.136510

[**1Co**-2HF]$^{+\bullet}$

E_{abs} = -5693.247686 hartrees

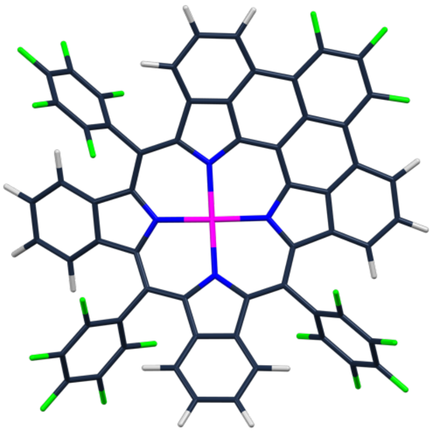

Atom	X	Y	Z
C1	1.069625	-2.865293	0.268352
C2	2.208837	-3.646086	0.575121
C3	3.269394	-2.774195	0.780208
C4	2.703443	-1.439249	0.458438
C5	3.398723	-0.288803	0.247549
C6	2.778150	0.908289	-0.215203
C7	3.498778	2.047118	-0.806642
C8	2.520708	2.991488	-1.124501
C9	1.264118	2.455915	-0.598341
C10	0.148267	3.180310	-0.287950
N11	1.459386	1.137081	-0.200723
C12	-1.048476	2.605613	0.207332
C13	-2.205666	3.360011	0.692250
C14	-3.269333	2.455081	0.714418
C15	-2.709625	1.166446	0.292245
C16	-3.412540	0.012030	0.054491
C17	-2.790975	-1.221542	-0.205981
C18	-3.493093	-2.497180	-0.493244
C19	-2.487716	-3.456302	-0.429435
C20	-1.276575	-2.788953	-0.189230
C21	-0.142978	-3.518718	0.021574
N22	-1.459969	-1.420077	-0.103579
N23	-1.343750	1.299708	0.094347
N24	1.332389	-1.557819	0.226845
H25	-5.618862	-2.267484	-0.887302
C26	-4.773436	-2.932120	-0.781276
C27	-2.631436	-4.829857	-0.538320
H28	-4.188889	-6.297613	-0.940437
C29	-3.949470	-5.252251	-0.820671
H30	-5.961400	-4.670926	-1.179661
C31	-4.963997	-4.315619	-0.950159
C32	2.233109	-5.027163	0.658841
H33	5.347559	-2.717137	1.419256
C34	4.476806	-3.314732	1.188793
H35	5.477176	-5.150901	1.645470
C36	4.542178	-4.709034	1.321622
H37	3.617734	-6.622730	1.166550
C38	3.474240	-5.559415	1.053846
H39	5.613856	1.568994	-0.926565
C40	4.827940	2.269477	-1.158692
H41	2.080627	4.847707	-2.147543
C42	2.836187	4.139017	-1.840752
H43	4.427445	5.232760	-2.765868
C44	4.161315	4.348950	-2.198902
H45	6.176263	3.615460	-2.130432
C46	5.146955	3.431816	-1.847877
H47	-1.546775	5.366545	1.185981
C48	-2.367443	4.665680	1.147006
H49	-5.362375	2.176105	1.215782
C50	-4.523174	2.853301	1.169525
H51	-5.654983	4.485082	1.964098
C52	-4.686214	4.162399	1.602204
H53	-3.763365	6.066412	1.964347
C54	-3.619472	5.057166	1.598347
C55	-0.195739	-4.973049	0.010898
C56	-0.300143	-7.756764	-0.041847
C57	-1.426816	-5.622409	-0.299449
C58	0.978960	-5.722891	0.324274
C59	0.882903	-7.106485	0.281980
C60	-1.428551	-7.020710	-0.326381
C61	0.172796	4.650363	-0.494203
C62	0.200574	7.414128	-0.880465
C63	0.927764	5.478847	0.327012
C64	-0.569264	5.237200	-1.510486
C65	-0.565132	6.607662	-1.712015
C66	0.951417	6.851096	0.143672
C67	-4.895850	0.034657	0.090421
C68	-7.685203	0.054610	0.148697
C69	-5.590821	-0.455550	1.188843
C70	-5.627105	0.530987	-0.980442
C71	-7.013076	0.547567	-0.962175
C72	-6.975038	-0.450796	1.230789
C73	4.869006	-0.304805	0.448955

C74	7.632061	-0.320296	0.846975
C75	5.721605	-0.897202	-0.475770
C76	5.431026	0.278555	1.576030
C77	6.801687	0.278274	1.784947
C78	7.093244	-0.912510	-0.289407
F79	-4.918347	-0.943035	2.225831
F80	-7.621846	-0.921990	2.284281
F81	-9.004250	0.065400	0.176970
F82	-7.695739	1.027554	-1.988081
F83	-4.995230	1.008195	-2.045572
F84	-2.532274	-7.698126	-0.621278
F85	-0.338498	-9.077273	-0.072349
F86	1.928116	-7.880031	0.549947
F87	5.217808	-1.460080	-1.568622
F88	7.889931	-1.482326	-1.179082
F89	8.937684	-0.328287	1.034265
F90	7.319444	0.841357	2.863357
F91	4.645978	0.862108	2.473933
F92	1.645061	4.953217	1.313927
F93	1.677222	7.625540	0.933258
F94	0.213771	8.720867	-1.062642
F95	-1.280543	7.149915	-2.683479
F96	-1.306763	4.473732	-2.309154
Co97	-0.011052	-0.121978	0.078082

[**1Co**-3HF]$^{+\bullet}$

E_{abs} = -5592.830756 hartrees

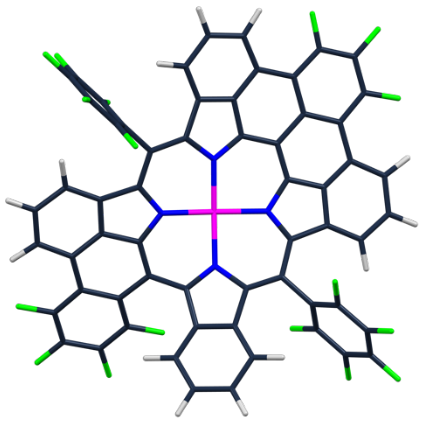

Atom	X	Y	Z
C1	-2.788733	-1.057138	0.000414
C2	-3.597330	-2.199447	0.207609
C3	-2.755352	-3.253079	0.539680
C4	-1.392636	-2.678526	0.433871
C5	-0.215527	-3.368499	0.475745
C6	1.033866	-2.779816	0.152197
C7	2.284842	-3.523433	-0.104591
C8	3.157659	-2.563160	-0.607318
C9	2.506709	-1.310286	-0.539923
C10	3.196105	-0.169758	-0.828517
N11	1.212752	-1.465948	-0.061984
C12	2.701805	1.073242	-0.296722
C13	3.526031	2.136583	0.280793
C14	2.635206	3.105255	0.742576
C15	1.299577	2.621227	0.384473
C16	0.157624	3.359800	0.308449
C17	-1.099638	2.803613	-0.050495
C18	-2.350464	3.555422	-0.287216
C19	-3.325124	2.565469	-0.374547
C20	-2.685278	1.325435	-0.235593
C21	-3.419484	0.177866	-0.219145
N22	-1.326463	1.480299	-0.074547
N23	1.411782	1.305945	-0.102249
N24	-1.487780	-1.316136	0.135350
H25	-2.078480	5.710335	-0.412167
C26	-2.758595	4.870165	-0.442762
C27	-4.684377	2.737155	-0.566601
H28	-6.109363	4.351457	-0.883540
C29	-5.076198	4.084623	-0.721397
H30	-4.462677	6.119703	-0.794242
C31	-4.126525	5.098124	-0.664445
C32	-4.980411	-2.223160	0.136352
H33	-2.777718	-5.345402	1.141000
C34	-3.342241	-4.472660	0.845140
H35	-5.214952	-5.491385	1.013839
C36	-4.738155	-4.547596	0.777376
H37	-6.621454	-3.617883	0.408288
C38	-5.552564	-3.471593	0.430553
H39	2.173961	-5.621194	0.458659
C40	2.765368	-4.814929	0.049713

C41	4.470859	-2.769590	-0.991785
H42	5.947360	-4.358520	-1.102956
C43	4.936827	-4.089344	-0.836255
H44	4.481127	-6.070132	-0.205058
C45	4.092700	-5.065970	-0.324249
H46	5.583169	1.469395	0.204072
C47	4.889076	2.234754	0.525239
H48	2.425343	4.957810	1.846773
C49	3.096393	4.209802	1.449053
H50	4.842572	5.165766	2.234712
C51	4.462981	4.318046	1.677389
H52	6.407425	3.444644	1.429007
C53	5.348993	3.343306	1.223131
C54	-4.868773	0.248515	-0.325905
C55	-7.634266	0.380483	-0.609911
C56	-5.490665	1.515166	-0.546047
C57	-5.641195	-0.946682	-0.196213
C58	-7.014455	-0.836323	-0.355773
C59	-6.880700	1.530455	-0.690305
C60	4.484137	-0.334934	-1.504602
C61	6.939481	-0.598197	-2.846281
C62	5.145720	-1.600660	-1.546474
C63	5.038163	0.719770	-2.240857
C64	6.253567	0.610687	-2.879942
C65	6.379217	-1.679389	-2.199087
C66	0.199522	4.813979	0.600488
C67	0.249743	7.549579	1.146387
C68	-0.325091	5.315535	1.784741
C69	0.744960	5.712281	-0.308206
C70	0.776958	7.072643	-0.047043
C71	-0.305496	6.671147	2.068339
C72	-0.253431	-4.826000	0.753662
C73	-0.324039	-7.565724	1.275910
C74	-0.455956	-5.742712	-0.270214
C75	-0.085288	-5.309402	2.043615
C76	-0.119187	-6.668546	2.316127
C77	-0.493554	-7.105093	-0.024072
F78	-0.862738	4.481265	2.668062
F79	-0.809557	7.132136	3.200985
F80	0.275437	8.842853	1.405224
F81	1.304271	7.914948	-0.919640
F82	1.254062	5.267470	-1.450565
F83	-7.532390	2.667927	-0.909329
F84	-8.946597	0.431210	-0.755982
F85	-7.808801	-1.897384	-0.268830
F86	-0.617946	-5.310178	-1.515246
F87	-0.688066	-7.965194	-1.010173
F88	-0.357989	-8.861354	1.522836
F89	0.042736	-7.113668	3.551034
F90	0.116247	-4.459565	3.043591
F91	7.048419	-2.829723	-2.251344
F92	8.101018	-0.711974	-3.461952
F93	6.755092	1.634990	-3.545765
F94	4.385778	1.875856	-2.362377
Co95	-0.058942	-0.006846	-0.122013

[**1Co**-4HF]$^{+\bullet}$

E_{abs} = -5492.425076 hartrees

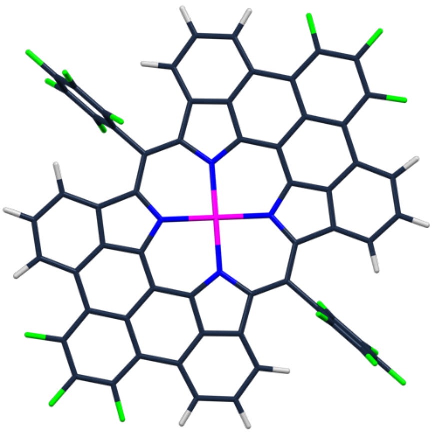

Atom	X	Y	Z
C1	-2.772647	-1.045222	0.030020
C2	-3.585399	-2.204548	-0.013054
C3	-2.743263	-3.305411	-0.079586
C4	-1.382109	-2.724472	-0.038350
C5	-0.214845	-3.437893	0.006962
C6	1.058285	-2.839906	0.038896
C7	2.335808	-3.587899	0.069523
C8	3.308700	-2.598149	-0.005615

C9	2.652057	-1.353721	-0.044459
C10	3.398674	-0.205438	-0.058792
N11	1.283022	-1.505652	-0.019011
C12	2.772022	1.045128	-0.054513
C13	3.582994	2.204665	-0.119772
C14	2.739255	3.305602	-0.156956
C15	1.380000	2.724445	-0.075342
C16	0.214662	3.437860	0.006960
C17	-1.056902	2.840075	0.076747
C18	-2.332878	3.587906	0.147471
C19	-3.307478	2.597933	0.102912
C20	-2.652431	1.353632	0.041987
C21	-3.399089	0.205353	0.048642
N22	-1.283349	1.505843	0.024361
N23	1.461336	1.334511	-0.037884
N24	-1.462105	-1.334620	0.005356
H25	-2.082922	5.744886	0.306834
C26	-2.756296	4.902932	0.251455
C27	-4.678613	2.779145	0.156965
H28	-6.131474	4.391846	0.299912
C29	-5.086895	4.126371	0.253350
H30	-4.484375	6.161206	0.380261
C31	-4.139021	5.137682	0.298527
C32	-4.969615	-2.218670	-0.027701
H33	-2.758443	-5.477390	-0.236739
C34	-3.325046	-4.561145	-0.169114
H35	-5.195198	-5.596132	-0.254740
C36	-4.723450	-4.623472	-0.182135
H37	-6.610003	-3.637970	-0.132803
C38	-5.540401	-3.501054	-0.113456
H39	2.090549	-5.744738	0.238625
C40	2.761976	-4.902992	0.161695
C41	4.680737	-2.779402	0.006593
H42	6.136881	-4.392528	0.106782
C43	5.091605	-4.126638	0.091558
H44	4.492927	-6.161295	0.239426
C45	4.145395	-5.137817	0.166861
C46	4.966060	2.218799	-0.178503
H47	2.749480	5.478279	-0.308173
C48	3.317958	4.561695	-0.261494
H49	5.184374	5.596927	-0.404695
C50	4.715200	4.624101	-0.319296
H51	6.602290	3.638288	-0.333438
C52	5.533875	3.501374	-0.279720
C53	-4.857412	0.286729	0.068906
C54	-7.645470	0.451545	0.112474
C55	-5.488934	1.564516	0.121746
C56	-5.634826	-0.910463	0.036774
C57	-7.017686	-0.782949	0.062375
C58	-6.885624	1.599623	0.138350
C59	4.856846	-0.286755	-0.083897
C60	7.644851	-0.451594	-0.128554
C61	5.489594	-1.564671	-0.053039
C62	5.632869	0.910486	-0.138532
C63	7.015829	0.783024	-0.156422
C64	6.886126	-1.599687	-0.080644
C65	0.290655	4.920197	0.014698
C66	0.434186	7.705930	0.032557
C67	0.462521	5.617032	1.203141
C68	0.191679	5.646174	-1.164657
C69	0.261618	7.030236	-1.169337
C70	0.535802	7.000793	1.225492
C71	-0.289956	-4.920238	0.011144
C72	-0.431132	-7.706184	0.019598
C73	-0.223361	-5.640578	-1.173824
C74	-0.428100	-5.622890	1.200516
C75	-0.499941	-7.006815	1.218199
C76	-0.292518	-7.024597	-1.183294
F77	0.560219	4.950678	2.347558
F78	0.700362	7.650757	2.365571
F79	0.501869	9.023485	0.040946
F80	0.164221	7.708408	-2.300674
F81	0.023329	5.007744	-2.316530
F82	-7.543280	2.753472	0.180821
F83	-8.964721	0.522110	0.132691
F84	-7.812232	-1.847179	0.039893
F85	-0.087076	-4.996575	-2.326876
F86	-0.226200	-7.697090	-2.320240
F87	-0.497865	-9.023803	0.023496
F88	-0.632663	-7.662419	2.359196
F89	-0.494317	-4.962270	2.350382
F90	7.544769	-2.753513	-0.061222
F91	8.964197	-0.522293	-0.150001
F92	7.809250	1.847282	-0.201886
Co93	0.000645	0.000031	0.049765

[**1Co**-5HF]$^{+\bullet}$

E_{abs} = -5391.986971 hartrees

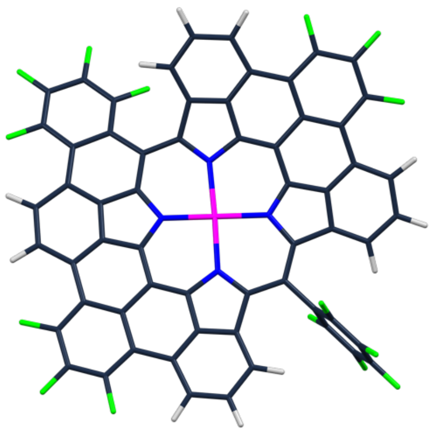

Atom	X	Y	Z
C1	-2.747204	0.032455	0.915225
C2	-3.577408	0.085492	2.059879
C3	-2.759813	0.305365	3.162645
C4	-1.385871	0.284239	2.610373
C5	-0.222808	0.228591	3.338438
C6	1.048267	0.034145	2.761569
C7	2.325582	-0.144613	3.501525
C8	3.280677	-0.302364	2.499982
C9	2.617061	-0.219099	1.270072
C10	3.337837	-0.347029	0.117021
N11	1.264414	-0.012179	1.426126
C12	2.691538	-0.209870	-1.102368
C13	3.483084	-0.192916	-2.268192
C14	2.629348	0.118199	-3.294513
C15	1.334709	0.249688	-2.724197
C16	0.266978	0.475778	-3.548059
C17	-1.034445	0.220059	-2.997396
C18	-2.264816	-0.135001	-3.727724
C19	-3.242172	-0.213847	-2.743017
C20	-2.612845	-0.017692	-1.501463
C21	-3.349400	-0.099817	-0.352028
N22	-1.256833	0.201573	-1.660981
N23	1.398578	0.024968	-1.354110
N24	-1.452680	0.165664	1.225463
H25	-1.911273	-0.485016	-5.840436
C26	-2.618816	-0.468254	-5.021570
C27	-4.573867	-0.539109	-2.921093
H28	-5.933946	-1.114285	-4.520116
C29	-4.923585	-0.842006	-4.256403
H30	-4.256344	-1.079770	-6.262135
C31	-3.958530	-0.816989	-5.254416
C32	-4.957700	-0.024778	2.057452
H33	-2.824426	0.660081	5.308345
C34	-3.370337	0.459608	4.397786
H35	-5.261856	0.490478	5.395995
C36	-4.766503	0.366161	4.440352
H37	-6.625825	0.063413	3.444406
C38	-5.556134	0.124060	3.319910
H39	2.125842	-0.083796	5.668775
C40	2.773886	-0.197382	4.811508
C41	4.643641	-0.519537	2.646318
H42	6.105089	-0.739208	4.243393
C43	5.068773	-0.575546	3.990011
H44	4.509008	-0.460046	6.037808
C45	4.146819	-0.414104	5.017706
C46	4.834637	-0.378899	-2.377869
C47	3.017550	0.220288	-4.601978
H48	4.843262	0.035989	-5.779541
C49	4.415580	-0.015319	-4.788645
H50	6.328555	-0.432898	-3.967342
C51	5.283498	-0.290874	-3.730510
C52	-4.793097	-0.306554	-0.438617
C53	-7.548192	-0.728757	-0.613061
C54	-5.393899	-0.539772	-1.712627
C55	-5.586875	-0.276412	0.750188
C56	-6.950338	-0.495524	0.617755
C57	-6.776358	-0.745591	-1.751754
C58	4.778918	-0.531762	0.141446
C59	7.532685	-0.926849	0.226475
C60	5.424544	-0.644215	1.408160
C61	5.521245	-0.586988	-1.089501
C62	6.889999	-0.789914	-0.996079
C63	6.808099	-0.845670	1.399184
C64	0.616321	0.844503	-4.937436
C65	1.205634	1.575937	-7.609994
C66	-0.283958	1.562734	-5.732579
C67	1.935818	0.643668	-5.485816
C68	2.166402	0.980417	-6.820701
C69	-0.024759	1.889914	-7.048430

C70	-0.304107	0.314458	4.818654
C71	-0.444281	0.478009	7.599760
C72	-0.504377	-0.822662	5.590625
C73	-0.172639	1.534489	5.467594
C74	-0.241086	1.628813	6.849007
C75	-0.576774	-0.754687	6.972401
F76	-1.438614	1.998356	-5.226869
F77	-0.930791	2.534123	-7.762167
F78	1.460200	1.890838	-8.865797
F79	3.364505	0.769075	-7.361505
F80	-7.402681	-0.960998	-2.903845
F81	-8.852283	-0.927573	-0.684857
F82	-7.757921	-0.493435	1.672019
F83	-0.631995	-2.004483	4.998229
F84	-0.770012	-1.846467	7.694115
F85	-0.511572	0.555466	8.915324
F86	-0.113924	2.798417	7.453389
F87	0.024804	2.639005	4.758017
F88	7.487739	-0.967536	2.533644
F89	8.839781	-1.120494	0.266033
F90	7.639803	-0.855860	-2.089427
Co91	-0.014741	0.059929	-0.072938

[**1Co**-6HF]$^{+\bullet}$

E_{abs} = -5291.562535 hartrees

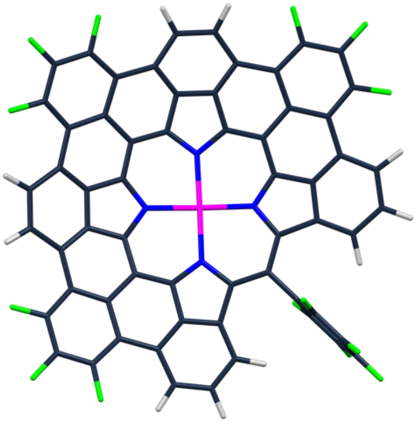

Atom	X	Y	Z
C1	-2.762705	0.468884	0.816670
C2	-3.589657	0.284738	1.936876
C3	-2.770890	0.279508	3.065088
C4	-1.391527	0.429765	2.523679
C5	-0.207445	0.395886	3.255390
C6	1.066679	0.398450	2.686058
C7	2.353414	0.182790	3.405134
C8	3.302108	0.062930	2.390767
C9	2.641209	0.272918	1.169064
C10	3.400091	0.287228	0.007788
N11	1.302999	0.472192	1.338596
C12	2.792651	0.557291	-1.190104
C13	3.538223	0.397196	-2.384261
C14	2.621553	0.459594	-3.409502
C15	1.377673	0.671410	-2.789122
C16	0.254473	0.632875	-3.575903
C17	-0.958327	0.758765	-2.948007
C18	-2.119410	0.606508	-3.727960
C19	-3.160773	0.561579	-2.828954
C20	-2.570982	0.674454	-1.547318
C21	-3.352524	0.480460	-0.439437
N22	-1.223142	0.819202	-1.611425
N23	1.471400	0.748769	-1.429733
N24	-1.447181	0.560719	1.157984
C25	-2.185908	0.231434	-5.046874
C26	-4.438988	0.180147	-3.124265
H27	-5.562056	-0.410819	-4.906833
C28	-4.594562	-0.116299	-4.523536
H29	-3.748987	-0.382905	-6.449579
C30	-3.531856	-0.098323	-5.428986
C31	-4.966266	0.101659	1.882602
H32	-2.859635	0.122983	5.234026
C33	-3.392620	0.128176	4.293665
H34	-5.287489	-0.140274	5.252039
C35	-4.788482	-0.024292	4.296959
H36	-6.635035	-0.189303	3.246131
C37	-5.569475	-0.049673	3.146040
H38	2.167399	0.169437	5.573159
C39	2.808875	0.072230	4.708621
C40	4.664675	-0.175495	2.521933

H41	6.132139	-0.473549	4.098445
C42	5.096133	-0.290470	3.857180
H43	4.550292	-0.244844	5.911961
C44	4.180672	-0.161524	4.896681
C45	4.816542	-0.070140	-2.508267
C46	2.841090	0.074792	-4.709051
H47	4.540271	-0.630628	-5.894381
C48	4.206035	-0.325592	-4.911971
H49	6.127932	-0.746991	-4.122786
C50	5.136153	-0.394453	-3.872901
C51	-4.767709	0.206832	-0.620670
C52	-7.478330	-0.344897	-0.932476
C53	-5.311623	0.060961	-1.946309
C54	-5.578407	0.043902	0.541946
C55	-6.931174	-0.220384	0.335582
C56	-6.672245	-0.214466	-2.047842
C57	4.808161	-0.070964	0.015555
C58	7.495351	-0.794867	0.064363
C59	5.443153	-0.287554	1.274371
C60	5.516539	-0.241980	-1.226122
C61	6.858409	-0.603171	-1.147552
C62	6.790150	-0.643634	1.248818
C63	0.337851	0.285345	-4.976309
C64	0.494234	-0.456700	-7.650035
C65	-0.879666	0.111980	-5.722897
C66	1.630886	0.028941	-5.552607
C67	1.652873	-0.331533	-6.898361
C68	-0.744202	-0.250513	-7.061635
C69	-0.305705	0.265562	4.732072
C70	-0.489801	0.016570	7.505291
C71	-0.338481	-0.984913	5.336106
C72	-0.366770	1.388648	5.545693
C73	-0.458152	1.277996	6.924869
C74	-0.429848	-1.122108	6.711488
F75	-1.817423	-0.419602	-7.821170
F76	0.569750	-0.795091	-8.926044
F77	2.804777	-0.576000	-7.507180
F78	-7.238858	-0.356387	-3.240283
F79	-8.768784	-0.598198	-1.070928
F80	-7.763818	-0.376379	1.357437
F81	-0.279999	-2.079037	4.585145
F82	-0.459331	-2.320707	7.271616
F83	-0.577072	-0.101375	8.816952
F84	-0.516311	2.357826	7.686934
F85	-0.338708	2.598887	5.000687
F86	7.463066	-0.863794	2.371614
F87	8.773247	-1.132921	0.097203
F88	7.571273	-0.772720	-2.254944
Co89	0.027940	0.697050	-0.111757

[**1Co**-7HF]$^{+\bullet}$

E_{abs} = -5191.110026 hartrees

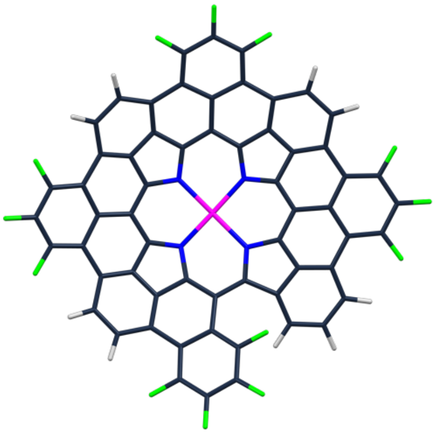

Atom	X	Y	Z
C1	-1.259616	2.665717	-0.675082
C2	-2.495714	3.323793	-0.461123
C3	-3.447099	2.331296	-0.423816
C4	-2.754569	1.135902	-0.661471
C5	-3.454411	-0.032025	-0.634310
C6	-2.755229	-1.197197	-0.870313
C7	-3.442825	-2.420204	-0.723090
C8	-2.468448	-3.394727	-0.776987
C9	-1.247890	-2.707053	-0.947979
C10	-0.076851	-3.390507	-0.817013
N11	-1.422740	-1.350840	-1.019518
C12	1.115289	-2.669942	-0.785357
C13	2.294992	-3.391735	-0.537575
C14	3.271642	-2.444475	-0.323380
C15	2.648751	-1.166318	-0.428706

C16	3.366325	-0.021722	-0.133640
C17	2.754988	1.256837	-0.388819
C18	3.369499	2.607105	-0.477133
C19	2.292056	3.488849	-0.437685
C20	1.122646	2.721667	-0.499942
C21	-0.087885	3.378732	-0.532627
N22	1.402086	1.371685	-0.526510
N23	1.319552	-1.335465	-0.766571
N24	-1.405554	1.338320	-0.810133
C25	4.628296	3.159537	-0.633635
C26	2.325618	4.877936	-0.433439
H27	3.791914	6.478731	-0.571941
C28	3.624179	5.412083	-0.554651
H29	5.699028	5.006461	-0.796797
C30	4.719198	4.560225	-0.675234
C31	-2.701566	4.622546	-0.097883
C32	-4.751090	2.469396	-0.021581
H33	-6.032900	4.120360	0.631626
C34	-5.039141	3.839077	0.309363
H35	-4.391296	5.844098	0.564690
C36	-4.072877	4.852684	0.271222
C37	-4.714672	-2.587223	-0.235229
C38	-2.625686	-4.694940	-0.375904
H39	-4.265821	-5.949788	0.349030
C40	-3.981085	-4.955511	0.031356
H41	-5.936525	-4.259869	0.470011
C42	-4.963649	-3.962357	0.102278
C43	2.414283	-4.740430	-0.297182
C44	4.544736	-2.752612	0.064198
H45	5.740904	-4.527976	0.484274
C46	4.765695	-4.160516	0.199144
H47	4.004993	-6.140536	0.227174
C48	3.751248	-5.105672	0.045165
C49	-0.180718	4.813206	-0.315627
C50	-0.307414	7.558279	0.110168
C51	1.030136	5.565337	-0.294713
C52	-1.466077	5.434949	-0.102089
C53	-1.473342	6.806373	0.108662
C54	0.915390	6.941752	-0.084947
C55	-0.101480	-4.806288	-0.502875
C56	-0.144407	-7.515441	0.136925
C57	-1.369484	-5.463101	-0.309992
C58	1.143238	-5.496048	-0.302999
C59	1.068200	-6.851214	-0.002753
C60	-1.332749	-6.822168	0.003631
C61	4.700111	-0.282107	0.471429
C62	7.287444	-0.688940	1.582696
C63	5.340663	0.675131	1.271546
C64	5.330273	-1.583323	0.447562
C65	6.624658	-1.719484	0.952357
C66	6.617142	0.509754	1.773292
C67	-4.837420	-0.054158	-0.222388
C68	-7.465888	-0.106898	0.657241
C69	-5.493920	1.193953	0.072229
C70	-5.477539	-1.330565	-0.033900
C71	-6.797346	-1.298875	0.402469
C72	-6.818185	1.107551	0.502421
F73	7.179813	1.478044	2.475679
F74	8.507871	-0.853912	2.057208
F75	7.246036	-2.895640	0.878403
F76	1.992022	7.717618	-0.050723
F77	-0.365386	8.863910	0.310342
F78	-2.618332	7.446147	0.314303
F79	-7.498854	2.209267	0.788186
F80	-8.723363	-0.136133	1.066288
F81	-7.467177	-2.426545	0.598388
F82	-2.463166	-7.493419	0.186640
F83	-0.157205	-8.806190	0.424592
F84	2.171335	-7.564428	0.181491
Co85	-0.008769	0.018247	-0.865915
H86	5.525782	2.560947	-0.729828
F87	4.715323	1.791013	1.647329

[**1Co**-8HF]$^{+\bullet}$

E_{abs} = -5090.691998 hartrees

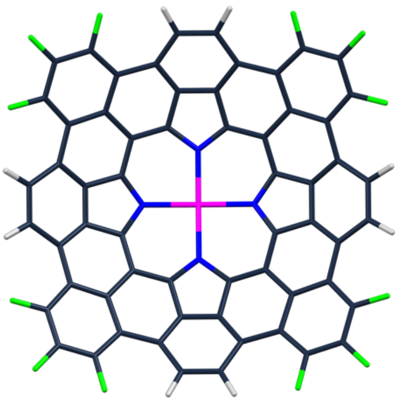

Atom	X	Y	Z
C1	2.939869	0.342063	-1.160454
C2	3.935904	1.284597	-0.763596
C3	3.299588	2.511099	-0.766979
C4	1.963633	2.245727	-1.163455
C5	1.023763	3.228060	-0.987446
C6	-0.342056	2.939868	-1.160454
C7	-1.284590	3.935903	-0.763596
C8	-2.511093	3.299587	-0.766979
C9	-2.245720	1.963632	-1.163455
C10	-3.228053	1.023761	-0.987446
N11	-0.922200	1.785514	-1.460050
C12	-2.939862	-0.342058	-1.160454
C13	-3.935896	-1.284592	-0.763596
C14	-3.299580	-2.511094	-0.766979
C15	-1.963626	-2.245722	-1.163455
C16	-1.023755	-3.228054	-0.987446
C17	0.342064	-2.939863	-1.160454
C18	1.284598	-3.935898	-0.763596
C19	2.511101	-3.299581	-0.766979
C20	2.245728	-1.963627	-1.163455
C21	3.228061	-1.023756	-0.987446
N22	0.922208	-1.785509	-1.460050
N23	-1.754012	-0.908936	-1.437916
N24	1.754009	0.909046	-1.437856
C25	1.027166	-5.133217	-0.153979
C26	3.643118	-3.776282	-0.158552
H27	4.277575	-5.613808	0.841905
C28	3.453355	-5.095662	0.372328
H29	2.165829	-6.709765	0.842690
C30	2.215975	-5.737389	0.374010
C31	5.133041	1.026046	-0.154493
C32	3.775245	3.643631	-0.158960
H33	5.613403	4.277000	0.841285
C34	5.094869	3.452962	0.371925
H35	6.708858	2.165722	0.842056
C36	5.736389	2.215609	0.373603
C37	-1.027201	5.133249	-0.154013
C38	-3.643130	3.776256	-0.158575
H39	-4.277586	5.613787	0.841912
C40	-3.453400	5.095645	0.372286
H41	-2.165855	6.709745	0.842660
C42	-2.216035	5.737371	0.374003
C43	-5.133012	-1.026011	-0.154456
C44	-3.775201	-3.643640	-0.158925
H45	-5.613413	-4.277039	0.841284
C46	-5.094839	-3.452971	0.371974
H47	-6.708891	-2.165773	0.842062
C48	-5.736372	-2.215594	0.373656
C49	4.494687	-1.413732	-0.370430
C50	6.921115	-2.169197	0.812550
C51	4.721331	-2.790546	-0.019337
C52	5.463463	-0.405288	-0.019401
C53	6.657483	-0.832637	0.547104
C54	5.954961	-3.117879	0.548807
C55	-4.494638	1.413760	-0.370443
C56	-6.921128	2.169143	0.812559
C57	-4.721325	2.790544	-0.019363
C58	-5.463415	0.405321	-0.019378
C59	-6.657477	0.832569	0.547135
C60	-5.954983	3.117841	0.548789
C61	-1.412591	-4.495078	-0.370815
C62	-2.169149	-6.921118	0.812616
C63	-0.404499	-5.464057	-0.019316
C64	-2.789485	-4.721257	-0.019497
C65	-3.117419	-5.954916	0.548821
C66	-0.832264	-6.657680	0.547196
C67	1.412625	4.495103	-0.370831
C68	2.169124	6.921143	0.812597
C69	2.789540	4.721279	-0.019518
C70	0.404479	5.464071	-0.019336

C71	0.832248	6.657702	0.547160
C72	3.117410	5.954954	0.548789
F73	0.043661	-7.599489	0.873890
F74	-2.521873	-8.078015	1.345777
F75	-4.376909	-6.228462	0.866094
F76	6.228973	-4.377125	0.866500
F77	8.077910	-2.522124	1.345801
F78	7.599124	0.043739	0.873370
F79	4.376892	6.228491	0.866063
F80	2.521893	8.078029	1.345770
F81	-0.043652	7.599522	0.873865
F82	-6.229027	4.377084	0.866496
F83	-8.077923	2.522090	1.345813
F84	-7.599133	-0.043826	0.873413
Co85	0.000004	0.000003	-1.643400

4. REFERENCES

1 M. Ruppel, D. Lungerich, S. Sturm, R. Lippert, F. Hampel, N. Jux, *Chem. Eur. J.* **2020**, *26*, 3287–3296.

2 B. D. Steinberg, E. A. Jackson, A. S. Filatov, A. Wakamiya, M. A. Petrukhina, L. T. Scott, *J. Am. Chem. Soc.* **2009**, *131*, 10537-10545.

3 J. P. Lewtak, D. T. Gryko, *Chem. Commun.* **2012**, *48*, 10069-10086.

4 M. Stepien, E. Gonka, M. Zyla, N. Sprutta, *Chem. Rev.* **2017**, *117*, 3479-3716.

5 Y. Minenkov, Å. Singstad, G. Occhipinti, V. R. Jensen, *Dalton Trans.,* **2012**, *41*, 5526–5541.

6 Y. Shao, L.F. Molnar, Y. Jung, J. Kussmann, C. Ochsenfeld, S.T. Brown, A.T.B. Gilbert, L.V. Slipchenko, S.V. Levchenko, D.P. O'Neill, R.A. DiStasio Jr., R.C. Lochan, T. Wang, G.J.O. Beran, N.A. Besley, J.M. Herbert, C.Y. Lin, T. Van Voorhis, S.H. Chien, A.Sodt, R.P. Steele, V.A. Rassolov, P.E. Maslen, P.P. Korambath, R.D. Adamson, B. Austin, J. Baker, E.F.C. Byrd, H. Dachsel, R.J. Doerksen, A. Dreuw, B.D. Dunietz, A.D. Dutoi, T.R. Furlani, S.R. Gwaltney, A. Heyden, S. Hirata, C-P. Hsu, G. Kedziora, R.Z. Khalliulin, P. Klunzinger, A.M. Lee, M.S. Lee, W.Z. Liang, I. Lotan, N. Nair, B. Peters, E.I. Proynov, P.A. Pieniazek, Y.M. Rhee, J. Ritchie, E. Rosta, C.D. Sherrill, A.C. Simmonett, J.E. Subotnik, H.L. Woodcock III, W. Zhang, A.T. Bell, A.K. Chakraborty, D.M. Chipman, F.J. Keil, A.Warshel, W.J. Hehre, H.F. Schaefer, J. Kong, A.I. Krylov, P.M.W. Gill and M. Head-Gordon, *Phys. Chem. Chem. Phys.* **2006**, *8*, 3172–3191.

7 J. P. Perdew, K. Burke, M. Ernzerhof, *Phys. Rev. Lett.* **1996**, *77*, 3865-3868.

8 F. Weigend, R. Ahlrichs, *Phys. Chem. Chem. Phys.* **2005**, *7*, 3297-3305.

9 F. Weigend, *Phys. Chem. Chem. Phys.* **2006**, *8*, 1057-1065.

10 M. Sierka, A. Hogekamp, R. Ahlrichs, *J. Chem. Phys.* **2003**, *118*, 9136-9148.

11 S. Grimme, J. Antony, S. Ehrlich, H. Krieg, *J. Chem. Phys.* **2010**, *132*, 154104.

12 TURBOMOLE V6.5 **2013**, University of Karlsruhe and Forschungszentrum Karlsruhe GmbH, 1989-2007**,** http://www.turbomole.com.

13 *NSDGUI*, Version 1.3 alpha version; Sandia National Laboratory: New Mexico, **2001**.

14 W. Jentzen, X.-Z. Song, J. A. Shelnutt, *J. Phys. Chem. B,* **1997**, *101*, 1684–1699.

15 W. Jentzen, J.-G. Ma, J. A. Shelnutt, *Biophys. J.* **1998**, *74*, 753–763.

16 J. A. Shelnutt, *J. Porphyrins Phthalocyanines,* **2001**, *5*, 300–311.

17 J. Schindler, S. Kupfer, A. A. Ryan, K. J. Flanagan, M. O. Senge, B. Dietzek, *Coord. Chem. Rev.,* **2018**, *360*, 1–16.

18 M. O. Senge, S. A. MacGowan, J. M. O'Brien, *Chem. Commun.,* **2015**, *51*, 17031–17063.
